# Tumor Microenvironment Characterization in Breast Cancer Identifies Prognostic Pathway Signatures

**DOI:** 10.3390/genes13111976

**Published:** 2022-10-29

**Authors:** Ji Li, Jiayue Qiu, Junwei Han, Xiangmei Li, Ying Jiang

**Affiliations:** 1 College of Bioinformatics Science and Technology, Harbin Medical University, Harbin 150081, China; 2 College of Basic Medical Science, Heilongjiang University of Chinese Medicine, Harbin 150040, China

**Keywords:** breast cancer, pathway analysis, prognostic marker, tumor microenvironment

## Abstract

Breast cancer is one of the most common female malignancies worldwide. Due to its early metastases formation and a high degree of malignancy, the 10 year-survival rate of metastatic breast cancer does not exceed 30%. Thus, more precise biomarkers are urgently needed. In our study, we first estimated the tumor microenvironment (TME) infiltration using the xCell algorithm. Based on TME infiltration, the three main TME clusters were identified using consensus clustering. Our results showed that the three main TME clusters cause significant differences in survival rates and TME infiltration patterns (log-rank test, *p* = 0.006). Then, multiple machine learning algorithms were used to develop a nine-pathway-based TME-related risk model to predict the prognosis of breast cancer (BRCA) patients (the immune-related pathway-based risk score, defined as IPRS). Based on the IPRS, BRCA patients were divided into two subgroups, and patients in the IPRS-low group presented significantly better overall survival (OS) rates than the IPRS-high group (log-rank test, *p* < 0.0001). Correlation analysis revealed that the IPRS-low group was characterized by increases in immune-related scores (cytolytic activity (CYT), major histocompatibility complex (MHC), T cell-inflamed immune gene expression profile (GEP), ESTIMATE, immune, and stromal scores) while exhibiting decreases in tumor purity, suggesting IPRS-low patients may have a strong immune response. Additionally, the gene-set enrichment analysis (GSEA) result confirmed that the IPRS-low patients were significantly enriched in several immune-associated signaling pathways. Furthermore, multivariate Cox analysis revealed that the IPRS was an independent prognostic biomarker after adjustment by clinicopathologic characteristics. The prognostic value of the IPRS model was further validated in three external validation cohorts. Altogether, our findings demonstrated that the IPRS was a powerful predictor to screen out certain populations with better prognosis in breast cancer and may serve as a potential biomarker guiding clinical treatment decisions.

## 1. Introduction

Breast cancer is one of the most common female malignancies worldwide and also a heterogeneous disease with varied morphological appearances, molecular features, behavior, and response to therapy [1]. In recent years, various therapies have emerged that have enormous potential to improve the clinical outcomes of breast cancer. However, due to early metastases formation and a high degree of malignancy, the prognosis is poor, and the 10 year-survival rate of metastatic breast cancer does not exceed 30% [2]. Hence, prospective identification for “high-risk” breast cancer patients is important to improve clinical outcomes. Recently, in clinical practice, the American Joint Committee on Cancer (AJCC) classification system has been used to assess the risk of a specific patient based on the clinical stage. However, the AJCC classification system is primarily determined by clinicopathological staging without regard to the molecular biological characteristics, hampering its ability to provide optimal clinical care to patients [3]. Thus, there is an urgent need to identify a reliable biomarker for breast cancer prognostic prediction.

Breast cancer has long been thought of as a nonimmunogenic malignancy, but accumulating evidence indicates that this might not always be the case. Researchers have proven that tumors are not insular masses of proliferating cells [4]; instead, a variety of cell types make up the tumor microenvironment (TME), including tumor cells, inflammatory cells, and cancer-associated fibroblasts [5]. The TME is increasingly recognized to play a critical role in breast cancer progression and therapeutic responses [6,7]. For example, tumor-associated macrophages, a key component of the cancer microenvironment, produce growth factors to stimulate the proliferation of breast carcinoma cells [8,9,10,11], and the variety of the TME components acts in concert with genetic changes in tumor cells to allow for a dynamic response to a novel environment during tumor progression and after exposure to drugs [12]. An in-depth understanding of the role of the TME components in modulating the therapeutic response to breast cancer may provide important insights into prognosis prediction.

Nowadays, a growing number of studies report that many molecular biomarkers, such as single-gene or multi-gene signatures, can be used to predict breast cancer prognosis, unfortunately, drawbacks remain in using these biomarkers. For example, the non-standardized cutoff values for risk classification, unimpressive predictive ability, and high cost-effectiveness have limited the clinical application of existing biomarkers. Biological pathways reflect the key cellular mechanisms that dictate disease states, drug response, and altered cellular function. Increasingly, studies indicate that dysfunction of key pathways is associated with breast cancer initiation, progression, and outcomes [13,14]. For example, Xu et al. have demonstrated that Wnt signaling is involved mainly in the processes of breast cancer proliferation and metastasis [15]. Aberrant activation of the Hedgehog signaling pathway has been reported as an association between the basal-like subtype of breast cancer phenotype and a poor prognosis in terms of metastasis and breast cancer-related death [16]. Therefore, precise identification and understanding of aberrant pathways may provide insight into breast cancer therapeutic strategies and the development of novel agents.

In this study, a proposed gene signature-based algorithm, xCell, was first applied to evaluate the infiltration of 64 immune and stromal cells in TME based on clinically annotated breast cancer gene expression profiles. Based on TME infiltration, three main TME clusters were defined (TME cluster-A, TME cluster-B, and TME cluster-C) using consensus clustering, and systematically correlated the TME phenotype with biological pathways and pathological features of breast cancer. A TME-related pathway-based risk model was then constructed to predict the prognosis of breast cancer using multiple machine learning algorithms. Our results indicate that the IPRS signature could well distinguish the patients with a distinct prognosis in training and validation cohorts. Furthermore, the IPRS signature was also found to be an independent prognostic factor for breast cancer after adjustment by clinicopathologic factors (subtype, ER status, HER2 status, PR status, grade, tumor size, and tumor stage). Altogether, our findings demonstrate that the IPRS is a powerful predictor to screen out certain populations with better prognosis in breast cancer and may serve as a potential biomarker guiding clinical treatment decisions.

## 2. Materials and Methods

### 2.1. Breast Cancer Datasets and Preprocessing

We comprehensively searched for breast cancer gene expression datasets that were publicly available and had clinical annotation. Patients without survival information were excluded from our further analysis, and those with overall survival (OS) of 30 days or less were excluded because they were likely to have died due to infection or other factors instead of cancer itself. The overall survival of a sample was measured from the start of the sample’s diagnosis to the date of death or the end of follow-up. In total, we obtained four cohorts of patients with breast cancer for this study: (1) the Bernard cohort [17], consisting of 1901 breast cancer patients, which was used to construct the risk model; (2) GSE1456, consisting of 159 patients with breast cancer; (3) GSE20685, consisting of 327 patients; (4) and the Christina Yau cohort [18], consisting of 682 tumors. The raw data from the microarray datasets and the clinical information were downloaded from the Gene Expression Omnibus database (GEO; https://www.ncbi.nlm.nih.gov/geo/ (accessed on 3 October 2022)). The detailed information on cohorts used in our study is listed in Supplementary Table S1.

### 2.2. KEGG Pathway Information

Biological pathways reflect the key cellular mechanisms that dictate disease states, drug response, and altered cellular function, which helps to understand the pathogenesis and progression of cancer. An increasing number of studies indicate that dysfunction of key pathways is associated with breast cancer initiation, progression, and outcomes [13,19,20,21]. Therefore, precise identification and understanding of aberrant pathways may provide insight into breast cancer therapeutic strategies and the development of novel agents. We then downloaded the information on pathways from the Kyoto Encyclopedia of Genes and Genomes database (KEGG; http://www.kegg.jp/kegg/pathway.html (accessed on 1 October 2022)). KEGG pathways were grouped into two categories: metabolic and non-metabolic pathways. Considering the complexity of the metabolic pathways, only the non-metabolic pathways were used in our further analysis. Thus, 238 non-metabolic pathways were acquired from the KEGG pathways database. Detailed information on the non-metabolic pathways used in our study is listed in Supplementary Table S3.

### 2.3. Identification of Breast Cancer TME Phenotype Based on the Infiltration of Immune Cells

Previous research suggested that the infiltration of immune cells in the TME is critical to tumor progression, treatment, and prognosis [22]. To investigate the possible associations between distinct classes of immune cell infiltration and breast patient prognosis, the composition of immune cells in the TME of each tumor was first estimated using the xCell algorithm [23]. The xCell algorithm is a gene signature-based method, which can accurately infer the abundance of 64 human immune and stromal cell types, including B cells, T cells, NK cells, macrophages, DCs, myeloid subsets, and stromal cells. In this study, the xCell algorithm was run for 1000 permutations and quantile normalization was applied. Then, an unsupervised clustering, based on the hierarchical clustering machine learning algorithm, was used to explore a novel TME phenotype of breast cancer patients based on the infiltration of immune cells. A consensus clustering algorithm was applied to determine the number of clusters in the training cohort, via the ConsensusClusterPlus R package [24]. A clustering procedure was conducted with 1000 iterations, sampling 80% of the data each time to ensure the stability of classification. The optimal number of clusters was determined by the proportion of the ambiguous clustering algorithm and the consensus heatmap [25]. We then compared the clinicopathological factors with different TME patterns, and the Kaplan–Meier survival analysis was carried out to further estimate the prognosis of patients in different TME cluster groups. The log-rank test evaluated the statistically significant differences in different TME cluster groups.

### 2.4. Construction of the Pathway-Activity-Based Classification for Breast Cancer Patients

To identify disorder pathways associated with TME patterns, we first grouped breast cancer patients into TMEcluster-A, TMEcluster-B, and TMEcluster-C groups. Then differential activity pathways among the three TME clusters were identified using the limma R package [26], which implements an empirical Bayesian approach to estimate the pathway activity changes using the *t*-test. The pathways with a Benjamini–Hochberg false discovery rate adjusted *p* value < 0.01 were identified as the significant differential activity pathways among the three TME clusters. Then, a univariable Cox proportional hazards regression was applied to evaluate the relationships between the activities of significant differential pathways and patient prognosis, and pathways with FDR-corrected *p* values less than 0.01 remained. Next, the random forest algorithm was used to reduce noise or redundant pathways, and the activities of candidate pathways were selected as the input variables, while the survival status of patients was selected as the outcome. We implemented an iteration procedure in the algorithm to narrow down the pathway set in which the least essential pathways were discarded at each iteration step. In detail, we constructed ten thousand trees at each iteration step and set the square root of the number of input pathways to the size of randomly chosen pathways at each node of the single classification tree. Permutation testing estimated the important score for each pathway on out-of-bag samples, and one-third of the least important pathways were discarded at each step. After each discard, we re-estimated the generalization error of the classification on out-of-bag samples. We found that the generalization error changed slightly at first, but it increased sharply when less than 12 pathways were retained. Therefore, 12 pathways associated with prognostic classification were selected for subsequent analysis (Supplementary Figure S2). The least absolute shrinkage and selection operator (LASSO) regression was then used to further narrow the scope of the candidate pathways by selecting the pathways with optimal prediction, and 10-fold cross-validation was used to determine the regression shrinkage parameter. The multivariable Cox proportional hazards regression analysis was then performed on the potential predictive pathways to obtain the pathway-activity-based classification for breast cancer patients. The pathway-activity-based classification, termed the immune-related pathway-based risk score (IPRS), was calculated by the formula: IPRS = Σβi ×Pi, where the βi is the coefficient of pathway i in the multivariable Cox proportional hazards regression and the Pi represents the activities of pathway i. Then, maximally selected rank statistics analysis based on the IPRS and OS, which was performed by the survminer R package, was used to determine the optimal threshold value to stratify patients into high-risk and low-risk groups. The survival analysis was performed using Kaplan–Meier curves, with a *p* value calculated by the log-rank test, to estimate the two subgroups’ survival distributions. The robust performance of the IPRS model in predicting the prognosis of patients was validated in three external validation cohorts.

### 2.5. The Correlation between the IPRS and Immune-Related Features

To explore the potential mechanisms of the IPRS model, a gene-set enrichment analysis (GSEA) was carried out. Gene sets were downloaded from the MSigDB database of Broad Institute and included broad hallmarks and KEGG pathways. Then a ranked gene list was constructed using the gene different expression levels of high-risk and low-risk groups. GSEA was performed with 1000 permutations using the clusterProfiler R package [27]. Subsequently, the Benjamini–Hochberg procedure was used to adjust for multiple testing to control the FDR.

Moreover, we calculated several immune-related scores, including the tumor purity, estimate score, immune score, stromal score, cytolytic activity (CYT) score, major histocompatibility complex (MHC) score, and T cell-inflamed immune gene expression profile (GEP) score, and then Pearson correlation analysis was performed to assess the correlation between those scores and the IPRS model. The tumor purity, estimate, immune, and stromal scores were calculated for each patient by the Estimation of STromal and Immune cells in MAlignant Tumours using Expression data (ESTIMATE) algorithm, which was based on the expression of related molecular biomarkers in immune and stromal cells [28]. The CYT, MHC, and GEP scores have been demonstrated to be efficient biomarkers for predicting the immune response [29,30,31,32]. The CYT score and MHC score were calculated by taking the mean expression of their signature genes. The GEP score was estimated by performing ssGSEA using the GEP gene list, which is listed in Supplementary Table S2.

## 3. Results

### 3.1. The Landscape of TME in Breast Cancer and Clinicopathological Characteristics of TME Phenotypes

**Figure 1:**
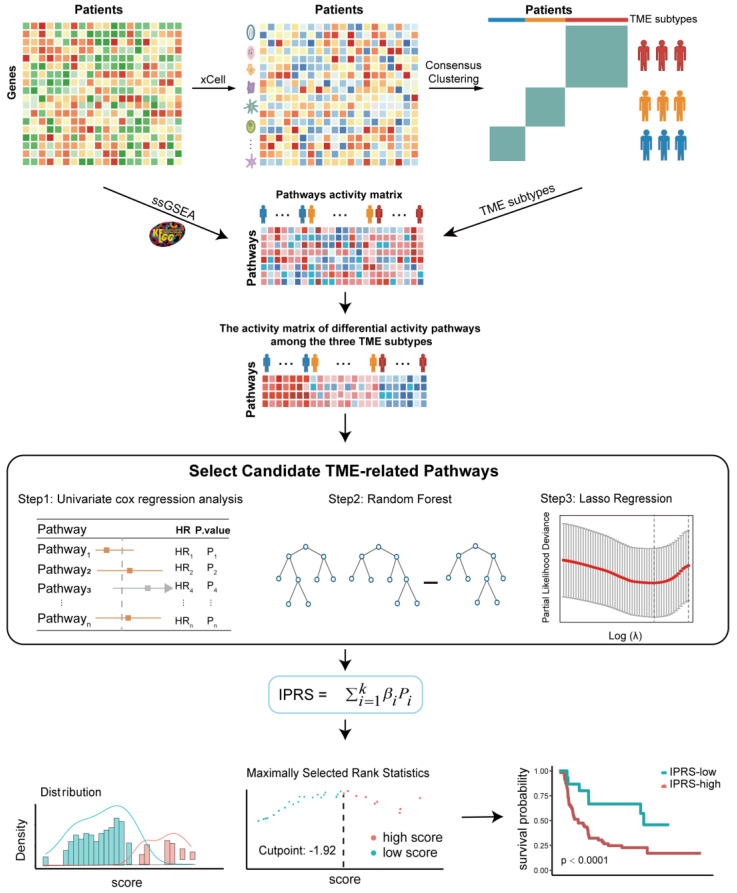
Flowchart for constructing IPRS based on pathway activity in breast cancer patients.

**Figure 2:**
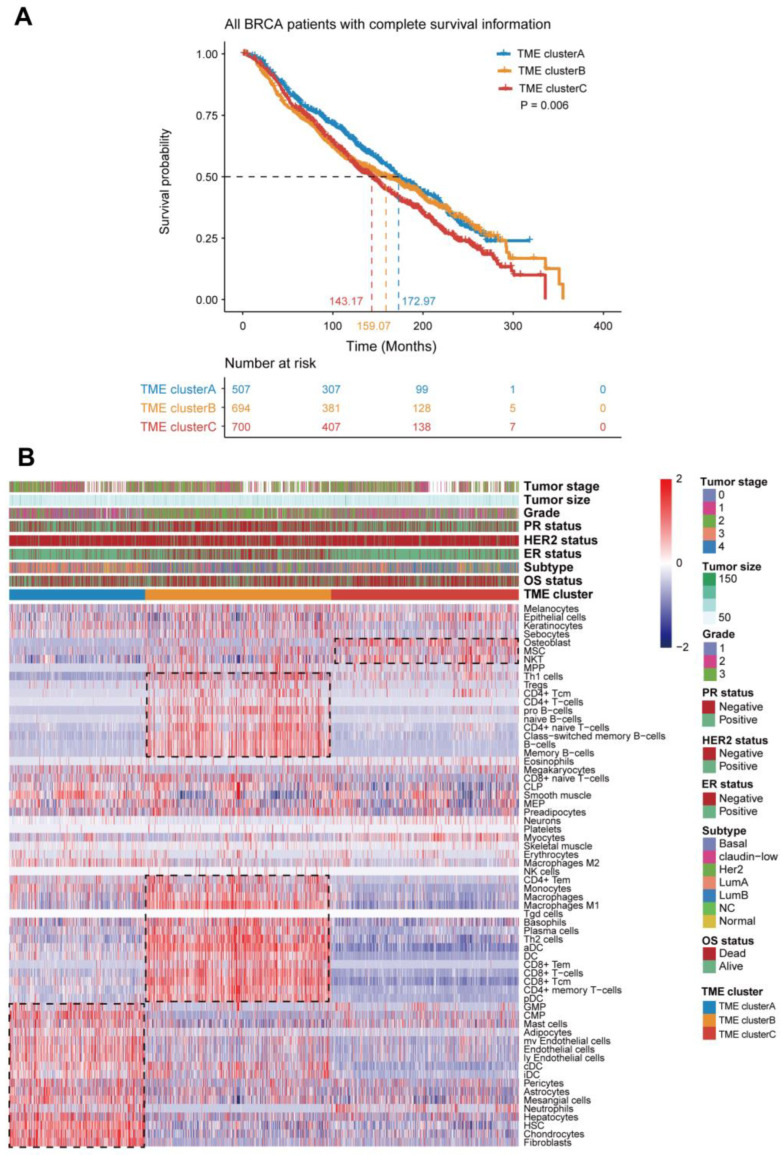
The landscape of the TME in breast cancer and characteristics of TME subtypes. (**A**) Kaplan–Meier curves for overall survival (OS) of all BRCA patients with three TME subtypes. The log-rank test showed an overall *p* = 0.006. (**B**) Consensus clustering of tumor-infiltrating cells in Bernard cohorts. Rows represent tumor-infiltrating cells, and columns represent samples.

The overall flowchart of our study is displayed in Figure 1. First, we used the xCell, a gene signature-based algorithm, to estimate the infiltration of 64 immune and stromal cell types in breast tumor tissue. Based on 1901 breast tumors with matched microenvironment infiltration profiles from the Bernard cohort, unsupervised clustering was performed to stratify the BRCA patients into distinct TME subtypes. The consensus heatmap showed that the optimal number of clusters was three, and the BRCA patients were then stratified into three groups: TME cluster-A, TME cluster-B, and TME cluster-C (Supplementary Figure S1A). The Kaplan–Meier survival analysis indicated that three TME subtypes showed significant differences in survival (log-rank test, *p* = 0.006; Figure 2A).

**Figure 3:**
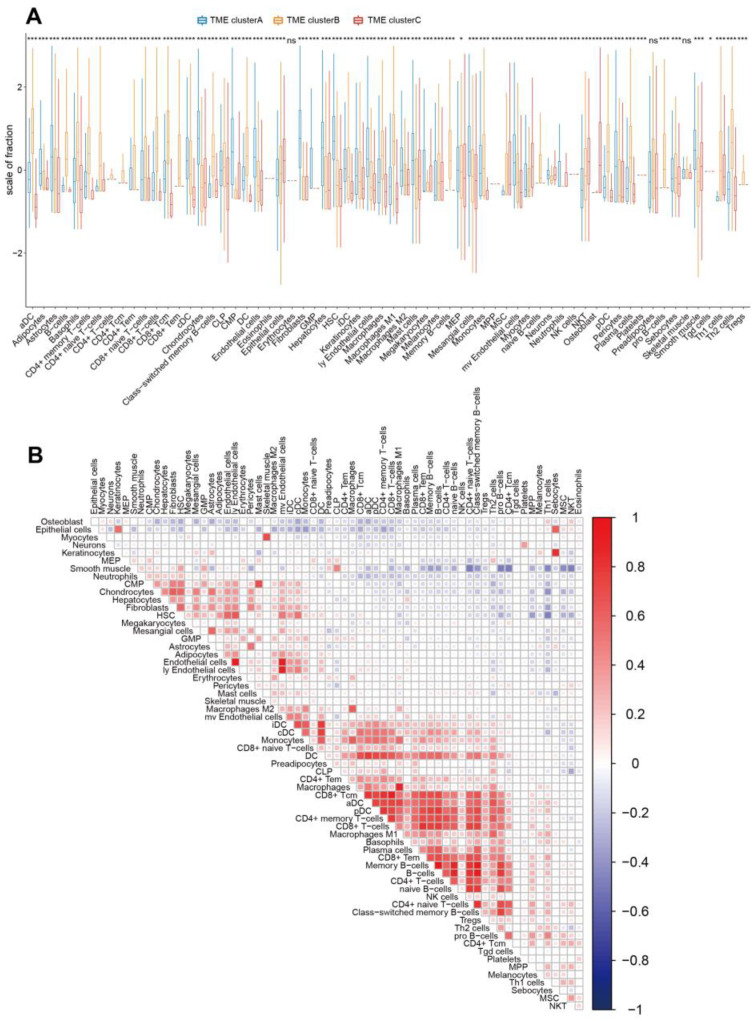
(**A**) The fraction of tumor-infiltrating cells in three TME subtypes. The statistical difference between the three TME subtypes was compared through the Kruskal–Wallis test. * *p* < 0.05; *** *p* < 0.001; ns no significance. (**B**) Cellular interaction of the TME subtypes.

To further explore the intrinsic biological differences that result in various clinical phenotypes, we then analyzed the immune cell composition of the TME among the three distinct TME subtypes. The results revealed that TME cluster-A was characterized by an increase in the infiltration of cancer-associated fibroblasts, mast cells, cDC, iDC, and neutrophils while having decreased infiltration of other TME cell subtypes. TME cluster-B was found to have a high infiltration of CD8 T cells, CD4 T cells, B cells, and plasma cells, while the TME cluster-C showed significant increases in the infiltration of NKT cells and osteoblasts (Figure 2B). Moreover, the significant differences in TME cell infiltration among the three distinct TME phenotypes were confirmed by the Kruskal–Wallis tests, in which 62 of 64 cell types showed significantly different infiltration (Figure 3A). Henriques et al. found that mast cells were associated with the prognosis of mastocytosis [33]. The CD8 and CD4 T cells are the key components of the cancer microenvironment, which is strongly related to the occurrence and development of cancer [34,35]. An increasing number of studies has found that not only the immune cells but also the stromal cells play important roles in the progression and prognosis of tumors [36,37,38,39]. For example, the osteoblasts, the first bone-resident cells identified to regulate hematopoiesis, were posited as the remote regulators of lung cancer [40]. Heichler et al. observed that the STAT3 activation in fibroblasts promotes colorectal tumor development and correlates with poor prognosis [41]. Mesenchymal stem cells within tumor stroma promote breast cancer metastasis [42]. These findings confirmed that TME infiltration is associated with patients’ prognosis, indicating that distinct TME infiltration patterns result in various clinical phenotypes.

In order to visualize the correlations between the infiltration of the different cell types in TME, a correlation coefficient heatmap was generated. The heatmap showed that a highly positive association was exhibited between the infiltration of B or T cell subtypes (such as B-cells and memory B-cells, B-cells and class-switched memory B-cells) (Figure 3B), suggesting those immune cells may interact with each other to perform specific functions, thereby affecting the prognosis of breast cancer patients. Conversely, a highly negative association was found between the infiltration of immune and stromal cells (such as CD4+ naive T-cells and smooth muscle, CD4+ Tcm, and smooth muscle), which indicates immune and stromal cells may play an opposite function in the initiation and progression of tumors. For instance, a negative association was observed between the mesenchymal stem cells and DC infiltration. However, Karnoub et al. found mesenchymal stem cells within tumor stroma promote breast cancer metastasis [42], while Michea et al. and Banchereau et al. revealed that the DCs are specialized in triggering adaptive immune response through the activation of T cells [43,44]. These findings indicate that different components in TME play distinct roles in the prognosis of patients, and our body will regulate the infiltration degree of different components to affect the prognosis. A deep understanding of the molecular mechanism may provide new insights into breast cancer therapeutic strategies and the development of novel agents.

### 3.2. Construction of the Pathway-Activity-Based Classification for BRCA Patients

**Figure 4:**
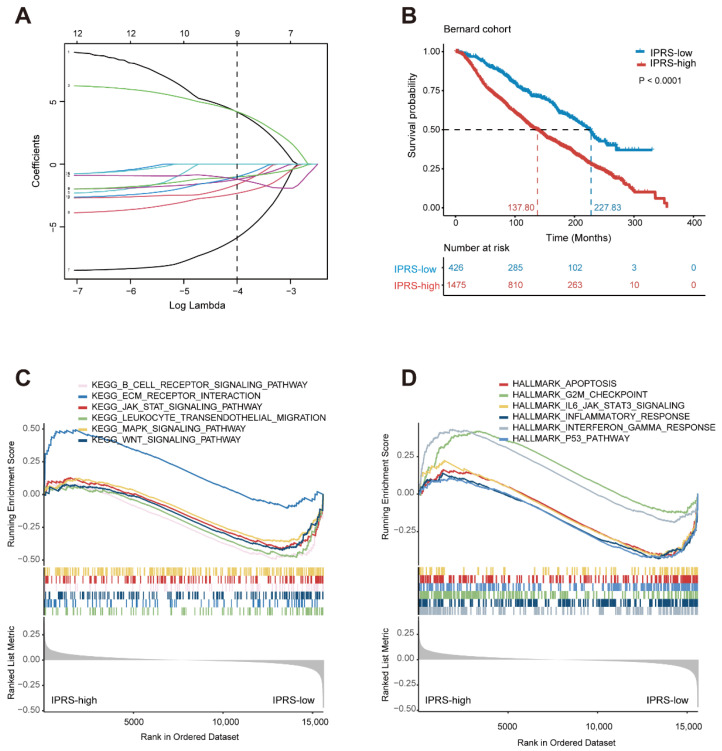
Identification of a 9-pathway signature for overall survival by LASSO regression analysis in Bernard cohort. (**A**) LASSO coefficient spectrum of 12 pathways in breast cancer; the dashed line represents the number of pathways corresponding to the covariates used for multivariate Cox analysis. (**B**) The Kaplan–Meier curves of the IPRS-high and IPRS-low groups in Bernard cohorts. (**C**,**D**) GSEA plot of significant hallmark pathways (**C**) and KEGG pathways (**D**) in comparison between the IPRS-high and IPRS-low groups.

Biological pathways reflect the key cellular mechanisms that dictate disease states, drug response, and altered cellular function, which helps us to understand the pathogenesis and progression of cancer. To identify the underlying disorder pathways that are associated with TME patterns, the different activity pathways among the three TME clusters were first identified using the “limma” R package. Next, a univariable Cox proportional hazards regression was performed to estimate the association between each different activity pathway and patient survival, and 29 pathways with FDR-corrected *p* values less than 0.01 remained. Then, random forest and LASSO regression were employed to further narrow the scope of the signature pathways and to prevent overfitting (Figure 4A). Finally, nine dysregulated pathways were identified, including progesterone-mediated oocyte maturation, neuroactive ligand-receptor interaction, apoptosis, ovarian steroidogenesis, estrogen signaling pathway, acute myeloid leukemia, bladder cancer, longevity regulating pathway, amoebiasis (Table 1). Apoptosis is an ordered and orchestrated physiological and pathological cellular process, which has been reported to play a pivotal role in the pathogenesis of diseases [45], and tumor resistance to apoptosis is the major contributor to poor therapeutic responses during cancer intervention [46].

An apparent dysfunction in ovarian steroidogenesis has been shown to be associated with polycystic ovarian syndrome and plays a crucial role in its prognosis [47,48]. Researchers found that approximately 70% of invasive breast cancers have some degree of independence from the estrogen hormone for cell proliferation and growth [49], and Keenan et al. found that estrogen signaling was independently associated with resistance in hormone-positive breast cancer [50,51]. These studies suggest that the dysfunction pathways identified by our study are partly associated with the development and progression of tumors.

To further explore whether the signature pathways can jointly use to predict the prognosis of BRCA patients, a risk model termed TME-related pathway-based risk score (IPRS) was calculated using a formula derived from the activity of the nine signature pathways weighted by their multivariable Cox proportional hazards regression coefficient (Table 1). An optimal cutoff value of −1.92 was used to classify patients into IPRS-high and IPRS-low groups. The Kaplan-Meier survival analysis demonstrated that the IPRS-high group was associated with poor prognosis (median OS, 137.80 months vs. 227.83 months; log-rank test *p* < 0.0001; Figure 4B). Moreover, the multivariable Cox regression showed that the IPRS was an independent prognostic factor after adjustment by clinicopathologic factors, including subtype, ER status, HER2 status, PR status, grade, tumor size, and tumor stage (Table 2).

### 3.3. The Correlation between the IPRS and Immune-Related Features

To explore the potential mechanisms which lead to the distinct outcomes of the IPRS-high and IPRS-low groups, a gene-set enrichment analysis (GSEA) was performed using the clusterProfiler R package [27]. The IPRS-low patients were significantly enriched in several immune-associated signaling aspects, including the B cell receptor signaling pathway, JAK-STA signaling pathway, inflammatory response, and P53 pathway, while the IPRS-high patients were significantly enriched in ECM receptor interaction, G2M checkpoint, and interferon-γ response (Figure 4C,D and Table 3).

**Figure 5:**
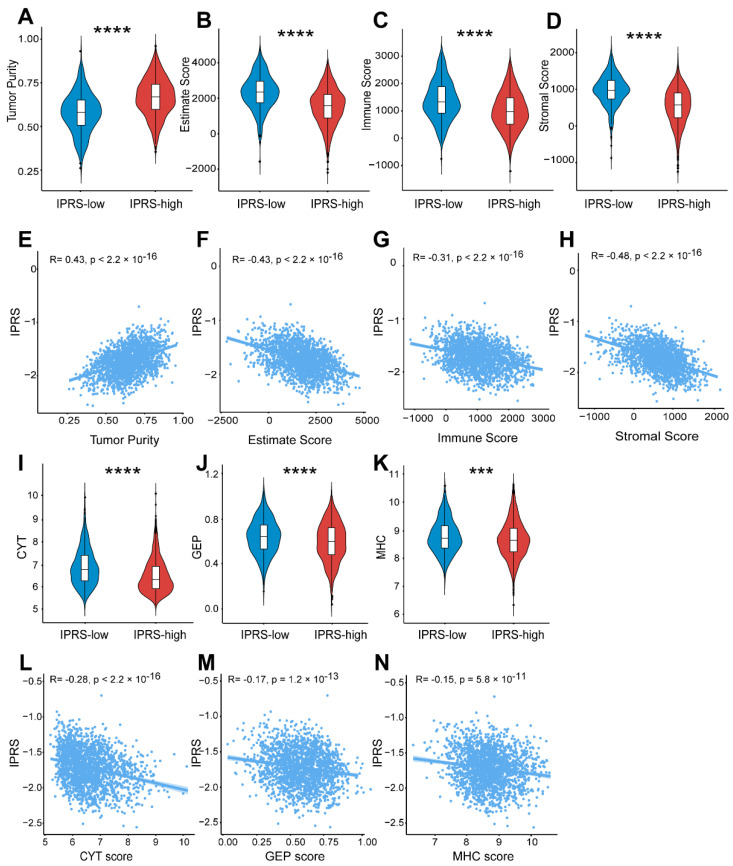
Correlation between the IPRS and immune-related features. (**A**–**D**) Comparison of the tumor purity score (**A**), estimate score (**B**), immune score (**C**), and stromal score (**D**) between the IPRS-high and IPRS-low groups. (**E**–**H**) Scatter plots of the linear fit of IPRS with the tumor purity score (**E**), estimate score (**F**), immune score (**G**), and stromal score (**H**). (**I**–**K**) Comparison of the CYT score (**I**), GEP score (**J**), and MHC score (**K**) between the IPRS-high and IPRS-low groups. (**L**–**N**) Scatter plots of the linear fit of IPRS with the CYT score (**L**), GEP score (**M**), and MHC score (**N**). *** *p* < 0.001; **** *p* < 0.0001.

We next calculated tumor purity, estimate score, immune score, and stromal score using the ESTIMATE algorithm. With relatively low tumor purity, the IPRS-low group had higher estimate, immune, and stromal scores when compared with the IPRS-high group (Wilcoxon test *p* < 0.01, Figure 5A–D). The correlation analysis indicated that the IPRS negatively correlated with estimate, immune, and stromal scores, while the opposite trend was observed in tumor purity (Pearson correlation test *p* < 0.01, Figure 5E–H). Moreover, we investigated the distribution of the immune cytolytic activity (CYT), T cell-inflamed immune gene expression profile (GEP), and major histocompatibility complex (MHC) scores among different risk patients, which have been demonstrated to be efficient biomarkers for predicting the immune response [29,30,31,32]. We found that IPRS-low patients had significantly higher CYT, GEP, and MHC scores than others (Wilcoxon test *p* < 0.01, Figure 5I–K); meanwhile, extremely significant negative associations were observed between the IPRS and three immune-related scores (Pearson correlation test *p* < 0.01, Figure 5L–N). Taken together, these findings strongly suggested that the IPRS signature is associated with immune-related features.

### 3.4. Validation of the Prognostic Value of the IPRS Model

**Figure 6:**
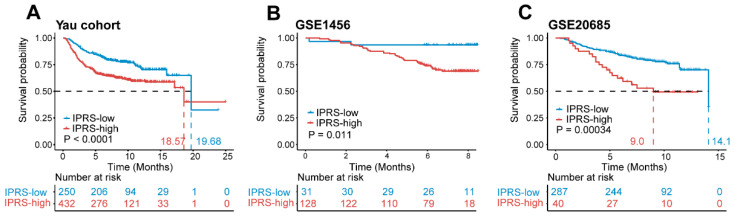
Comparisons of OS between the IPRS-high and IPRS-low groups across different cohorts. (**A**–**C**) Kaplan–Meier survival curves of OS comparing the IPRS-high and IPRS-low groups among patients with breast cancer from the Yau cohort (**A**), the GSE1456 (**B**), and the GSE20685 (**C**).

To evaluate the robust performance of the IPRS model to predict the prognosis of BRCA patients, we validated the predictive value of the IPRS model in three external validation cohorts. Using the optimal cutoff value obtained from the training cohort, patients were separated into IPRS-high and IPRS-low groups. In the first validation cohort (Yau cohort), the IPRS model discriminated 250 patients out of 682 into the IPRS-low group and 432 patients into the IPRS-high group. Patients with lower IPRS scores had more favorable OS rates than those in the IPRS-high group (median OS, 19.68 months vs. 18.57 months, log-rank test *p* < 0.0001; Figure 6A). In the second validation cohort (GSE1456), the Kaplan–Meier survival analysis showed that patients with lower IPRS obtained significantly longer OS (median OS, not reached, log-rank test *p* = 0.011; Figure 6B). In the third validation cohort (GSE20685), the median OS rate of IPRS-low patients was significantly superior to that of IPRS-high patients (median OS, 14.1 months vs. 9.0 months, log-rank test *p* = 0.00034; Figure 6C). These findings suggest that there is a strong predictive value of the IPRS model for the prognosis of breast cancer.

## 4. Discussion

Breast cancer is one of the most common female malignancies worldwide and also a heterogeneous disease with varied morphological appearances, molecular features, behaviors, and responses to therapy [1]. Due to early metastases formation and a high degree of malignancy, the prognosis is poor, and the 10 year-survival rate of metastatic breast cancer does not exceed 30% [2]. Thus, more precise biomarkers are urgently needed. Numerous studies have reported many molecular biomarkers, such as single-gene or multi-gene signatures, can be used to predict breast cancer prognosis; unfortunately, drawbacks remain in the use of these biomarkers. In recent years, there has been growing evidence that biological pathways reflect key cellular mechanisms and biological functions whose dysfunction plays an important role in tumorigenesis, progression, and prognosis [52,53,54,55,56,57,58].

Accumulating evidence shows that the TME signature is a robust biomarker for predicting survival in breast cancer and guiding more effective immunotherapy strategies. In our study, we first estimate the TME infiltration using the xCell algorithm. Based on the TME infiltration, three main TME clusters were identified using consensus clustering. Our results showed that the three main TME clusters are associated with significant differences in survival and TME infiltration patterns (Figure 2). The cancer-associated fibroblasts, mast cells, cDC, iDC, and neutrophils showed significantly higher infiltration in TME cluster-A patients while having decreased infiltration of other TME cell subtypes. The TME cluster-B was characterized by an increase in the infiltration of cancer-associated immune cells, such as Th1 cells, CD4 T cells, CD8 T cells, B cells, macrophages, and plasma cells. The TME cluster-C showed significant increases in the infiltration of osteoblasts, MSC cells, and NKT cells. These findings suggested that patients with different TME infiltration patterns have different prognoses.

To further explore the intrinsic biological differences that result in various clinical phenotypes, a multiple machine learning algorithm was used to develop a nine-pathway-based TME-related risk model. Based on the IPRS score, BRCA patients were divided into two subgroups, and patients in the IPRS-low group presented significantly better OS than the IPRS-high group (Figure 4B). Correlation analysis revealed that the IPRS-low group was characterized by increases in immune-related scores (CYT, MHC, GEP, estimate, immune, and stromal scores) while exhibiting decreases in tumor purity (Figure 5), suggesting IPRS-low patients may have a strong immune response. Additionally, the GSEA result confirmed that the IPRS-low patients were significantly enriched in several immune-associated signaling pathways. The prognostic value of the IPRS model was further validated in three external validation cohorts. Furthermore, multivariate Cox analysis revealed that the IPRS was an independent prognostic biomarker after adjustment by clinicopathologic characteristics.

Altogether, our findings demonstrated that the IPRS is a powerful predictor to screen out certain populations with better prognosis in breast cancer and may serve as a potential biomarker guiding clinical treatment decisions.

## Figures and Tables

**Table 1:** The identified significant signaling pathways and their coefficients were calculated by the multivariate Cox regression model.

Pathways	Coef	HR	95%CI	*p* Value
Progesterone-mediated oocyte maturation	6.27	530.25	6.21–45,308.78	<0.01
Acute myeloid leukemia	−3.20	0.04	0.0003–4.96	0.19
Bladder cancer	5.72	304.04	9.93–9311.16	<0.01
Neuroactive ligand-receptor interaction	−1.90	0.15	0.001–12.03	0.40
Apoptosis	−8.67	0.0001	0.0001–0.03	<0.01
Longevity regulating pathway—multiple species	−3.15	0.043	0.0003–6.28	0.22
Ovarian steroidogenesis	−1.33	0.27	0.02–4.34	0.35
Estrogen signaling pathway	−2.17	0.11	0.0005–24.11	0.43
Amoebiasis	−0.54	0.58	0.02–19.24	0.76

**Table 2:** Univariable and multivariable Cox analysis of IPRS and clinic-pathological factors (subtype, ER status, HER2 status, grade, PR status, tumor size, tumor stage) for OS in the Bernard cohort.

Training Cohort	Univariable Analysis			Multivariable Analysis		
	HR	95%CI	*p* Value	HR	95%CI	*p* Value
IPRS	2.70	2.20–3.40	<0.01	1.67	1.178–2.37	<0.01
Subtype	0.86	0.64–1.20	0.31			
Claudin-low				0.98	0.69–1.40	0.91
Her2				1.32	0.95–1.83	0.10
LumA				1.18	0.82–1.70	0.38
LumB				1.31	0.92–1.88	0.13
NC				1.37	0.33–5.71	0.67
Normal				1.39	0.90–2.16	0.14
ER status	0.85	0.74–0.97	0.021	0.95	0.71–1.25	0.69
HER2 status	1.50	1.20–1.70	<0.01	1.22	0.96–1.56	0.10
Grade	1.30	1.20–1.40	<0.01	1.04	0.91–1.18	0.61
PR status	0.79	0.70–0.89	<0.01	0.89	0.745–1.06	0.17
Tumor size	1.00	1–1	<0.01	1.01	1.00–1.01	<0.01
Tumor stage	1.80	1.60–2.00	<0.01	1.50	1.31–1.72	<0.01

**Table 3:** The gene-set enrichment analysis (GSEA) of IPRS.

Pathways	NES	*p* Value	*p* Adjust
**Hallmark Pathways**			
Hallmark interferon γ response	1.75	0.0000153	0.0001091
Hallmark G2M checkpoint	1.71	0.0000597	0.0002987
Hallmark inflammatory response	−1.73	0.0000195	0.0001217
Hallmark P53 pathway	−1.70	0.0000593	0.0002987
Hallmark apoptosis	−1.60	0.0007173	0.0032604
Hallmark IL6 JAK STAT3 signaling	−1.73	0.0090204	0.0214772
**KEGG C2 Pathways**			
KEGG ECM receptor interaction	1.76	0.0002955	0.0108816
KEGG leukocyte transendothelial migration	−1.77	0.0002973	0.0108816
KEGG WNT signaling pathway	−1.64	0.0003632	0.0110781
KEGG B cell receptor signaling Pathway	−1.69	0.0016411	0.0250264
KEGG JAK STAT signaling pathway	−1.59	0.0014223	0.0250264
KEGG MAPK signaling pathway	−1.49	0.0009607	0.0250264

## Data Availability

The Bernard cohort was downloaded from the cBioportal (http://www.cbioportal.org/ (accessed on 1 October 2022)). Three external validation cohorts, GES1456, GSE20685, and the Christina Yau cohort, were downloaded from the GEO database (https://www.ncbi.nlm.nih.gov/geo/ (accessed on 3 October 2022)) and UCSC Xena (https://xena.ucsc.edu/ (accessed on 1 October 2022)).

## Supplementary Materials

The following supporting information can be downloaded at: https://www.mdpi.com/article/10.3390/genes13111976/s1, Supplementary Figure S1. Consensus matrix of all BRCA samples displaying the clustering stability using 1000 iterations of hierarchical clustering. Supplementary Figure S2. The generalization error of random forest classification algorithm estimated by the retained pathways after each deletion. Supplementary Table S1. Data source. Supplementary Table S2. Gene list of immune-related gene signature. Supplementary Table S3. The KEGG pathways involved in this study.
