# Supplementary material for: Tumor Microenvironment Characterization in Breast Cancer Identifies Prognostic Pathway Signatures
Source: Genes (Basel). 2022 Oct 29;13(11):1976. doi: 10.3390/genes13111976 (PMC9690299; doi:10.3390/genes13111976)
Supplement: Supplementary file 1 [file genes-13-01976-s001.zip › genes-1993460-supplementary.pdf]

Supplementary materials

Tumor microenvironment characterization in breast cancer identifies prognostic pathway signatures

Contents:

Supplementary Figure S1-S2

Supplementary Table S1-S3

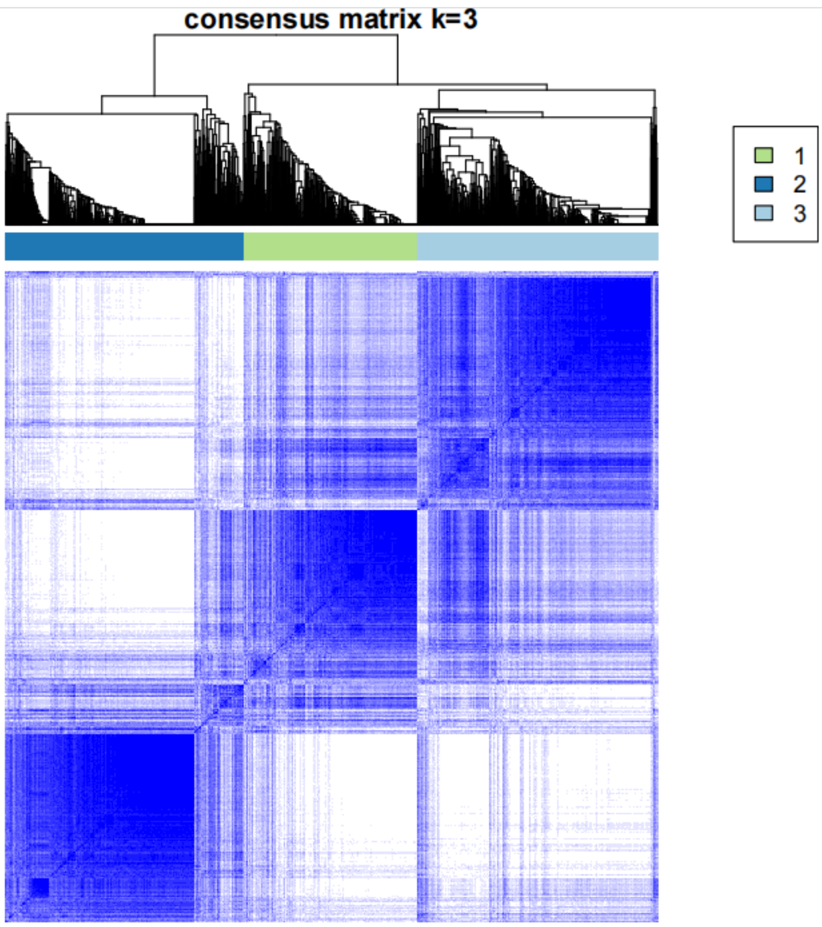

Supplementary Figure S1. Consensus matrix of all BRCA samples displaying the clustering stability using 1000 iterations of hierarchical clustering.

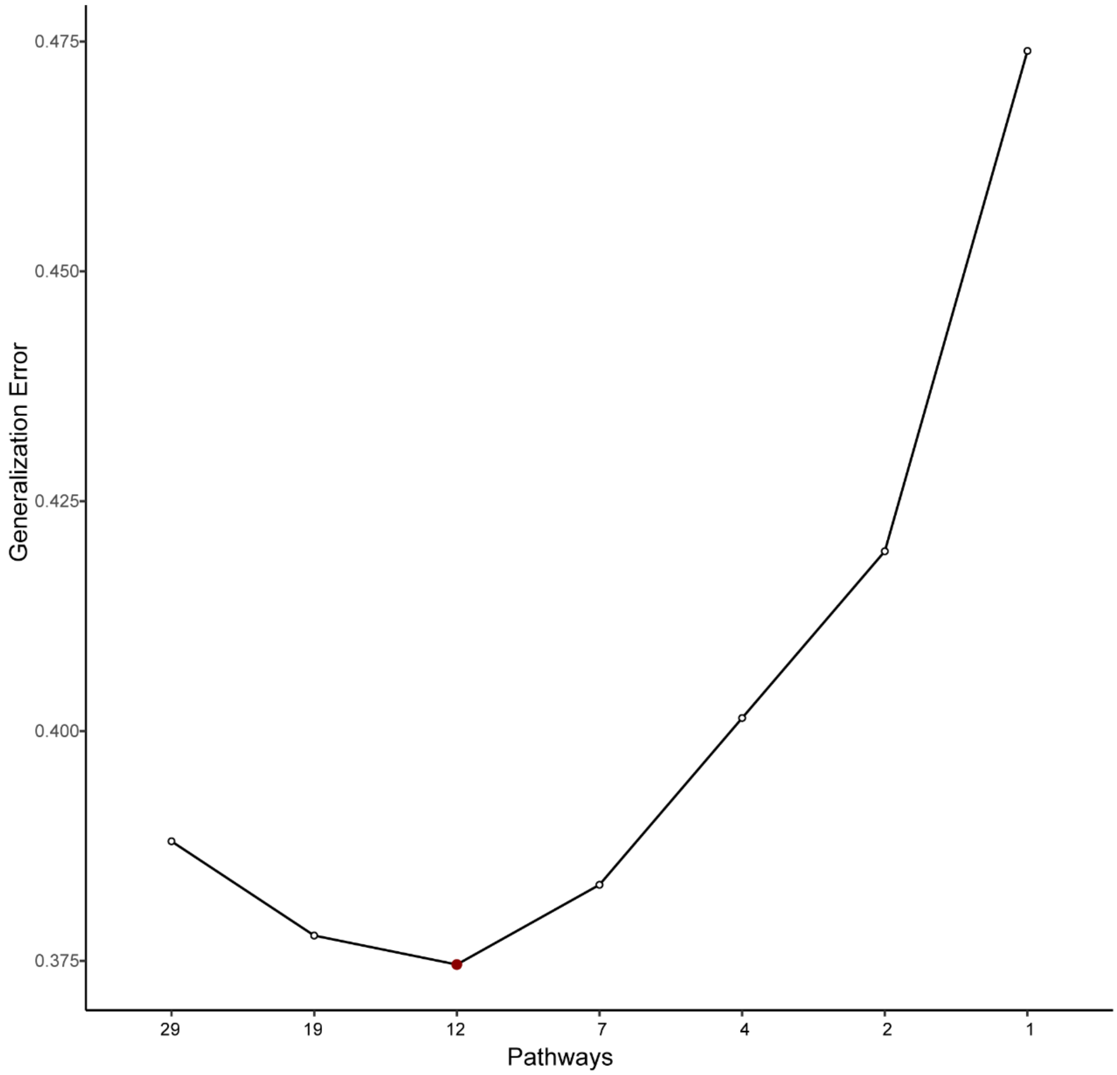

Supplementary Figure S2. The generalization error of random forest classification algorithm estimated by the retained pathways after each deletion.

Supplementary Table S1. Data source.

	Training Set	Validation set		
**Cohort**	**Bernard cohort**	**GSE1456**	**GSE20685**	**Christina Yau cohort**
**No. of samples**	1901	159	327	682
**Outcome**	OS	OS	OS	OS
**Tumor Size (cm)**	1-182 (mm)	NA	NA	NA
**Subtype**	Basal (n = 199)<br>claudin-low (n = 197)<br>Her2 (n = 220)<br>LumA (n = 678)<br>LumB (n = 461)<br>NC (n = 6)<br>Normal (n = 140)	Basal (n = 25)<br>ERBB2 (n = 15)<br>LumA (n = 39)<br>LumB (n = 23)<br>Normal (n = 37)<br>No subtype (n = 37)	NA	Basal (n = 180)<br>Her2 (n = 79)<br>LumA (n = 240)<br>LumB (n = 121)<br>Normal (n = 62)

Supplementary Table S2. Gene list of immune-related gene signature.

Classification	Genes
Cytolytic activity (CYT)	GZMA, PRF1
Major Histocompatibility complex (MHC)	HLA-A, HLA-B, HLA-C, TAP1, TAP2, NLRC5, PSMB9, PSMB8, B2M
Gene expression profile (GEP)	CCL5, CD27, CD274, CD276, CD8A, CMKLR1, CXCL9, CXCR6, HLA-DQA1, HLA-DRB1, HLA-E, IDO1, LAG3, NKG7, PDCD1LG2, PSMB10, STAT1, TIGIT

Supplementary Table S3. The KEGG pathways involved in this study.

KEGG Pathways	Genes
mRNA surveillance pathway	RBM8A; MAGOH; MAGOHB; EIF4A3; CASC3; ACIN1; RNPS1; SAP18; SRRM1; NXF1; NXF5; NXF3; NXF2; NXF2B; PNN; ALYREF; NXT1; NXT2; NCBP1; UPF3B; UPF3A; DDX39B; PYM1; UPF1; UPF2; SMG1; SMG5; SMG7; SMG6; ETF1; GSPT2; GSPT1; RNGTT; RNMT; NUDT21; PAPOLA; PAPOLB; PAPOLG; PCF11; CLP1; CPSF6; CPSF7; FIP1L1; CPSF3; CPSF2; CPSF1; CPSF4; CSTF1; CSTF2; CSTF2T; CSTF3; SYMPK; NCBP2; NCBP2L; BCL2L2-PABPN1; PABPN1L; PABPN1; PABPC4L; PABPC5; PABPC1; PABPC1L2A; PABPC3; PABPC1L2B; PABPC1L; PABPC4; PPP2R3B; PPP2R3C; PPP2CA; PPP2CB; PPP2R1A; PPP2R1B; PPP2R2A; PPP2R2B; PPP2R2C; PPP2R3A; PPP2R5A; PPP2R5B; PPP2R5C; PPP2R5D; PPP2R5E; PPP2R2D; HBS1L; PELO; MSI2; DAZAP1; MSI1; WDR33; WDR82; PPP1CA; PPP1CB; PPP1CC; SSU72; DDX19B; DDX19A; GLE1; FUS; TARDBP
RNA polymerase	POLR2B; POLR2A; POLR2C; POLR2D; POLR2E; POLR2F; POLR2G; POLR2H; POLR2I; POLR2L; POLR2J2; POLR2J; POLR2J3; POLR2K; POLR3B; POLR3A; POLR3C; POLR3D; POLR1C; POLR3K; POLR1D; POLR3H; POLR3G; POLR3GL; POLR3F; POLR1B; POLR1A; POLR1H; POLR1F; POLR1E; POLR3E
Spliceosome	DDX46; DDX39B; SNRNP70; SNRPA; SNRPC; DHX16; DHX38; CDC40; PRPF40B; PRPF40A; RBM25; DHX8; DHX15; SNRPB; SNRPD1; SNRPD2; SNRPD3; SNRPE; SNRPF; SNRPG; DDX5; TCERG1; SNRPA1; SNRPB2; SF3A1; SF3A3; SF3A2; SF3B4; SF3B2; DDX42; SF3B3; SF3B1; SF3B6; SF3B5; PHF5A; U2AF1L5; U2AF2; U2AF1L4; U2AF1; PUF60; SMNDC1; RBM17; CHERP; U2SURP; NCBP2; NCBP2L; NCBP1; RBMXL3; HNRNPA1L2; HNRNPA3; RBMXL2; RBMX; HNRNPA1; HNRNPC; HNRNPK; HNRNPU; HNRNPCL1; HNRNPM; RBMXL1; PCBP1; SRSF10; SRSF8; TRA2A; SRSF1; SRSF2; SRSF3; SRSF4; SRSF5; SRSF6; SRSF7; TRA2B; SRSF9; PRPF19; CDC5L; BCAS2; PLRG1; CWC15; CTNNBL1; HSPA1A; HSPA1B; HSPA1L; HSPA2; HSPA6; HSPA8; PQBP1; SYF2; CRNKL1; XAB2; WBP11; SNW1; ISY1-RAB43; ISY1; PPIL1; PPIE; CCDC12; RBM22; AQR; LSM6; LSM5; LSM4; LSM3; LSM7; LSM8; LSM2; PRPF31; PPIH; PRPF4; PRPF3; SNU13; EFTUD2; SNRNP200; PRPF6; PRPF8; SNRNP40; DDX23; TXNL4A; SNRNP27; USP39; SART1; ACIN1; EIF4A3; RBM8A; MAGOH; MAGOHB; ALYREF; THOC2; THOC3; THOC1; SLU7; ZMAT2; PRPF38B; PRPF38A; PRPF18; BUD31; RNVU1-4; RNVU1-20; RNVU1-1; RNVU1-18; RNVU1-7; RNU1-3; RNU1-2; RNU1-1; RNU1-4; RNU2-1; RNU5A-1; RNU4-2; RNU4-1; RNU6-9; RNU6-1; RP9; FUS
Proteasome	IFNG; PSMD3; PSMD12; PSMD11; PSMD6; PSMD7; PSMD13; PSMD14; PSMD8; SEM1; PSMD4; PSMD2; PSMD1; ADRM1; PSMC2; PSMC1; PSMC4; PSMC6; PSMC3; PSMC5; PSME1; PSME2; PSME3; PSME4; PSMA6; PSMA2; PSMA4; PSMA8; PSMA7; PSMA5; PSMA1; PSMA3; PSMB6; PSMB7; PSMB3; PSMB2; PSMB5; PSMB1; PSMB4; PSMB9; PSMB10; PSMB8; PSMB11; PSMF1; POMP; PSMD9
Base excision repair	APEX2; APEX1; FEN1; PARP2; PARP3; PARP1; PARP4; LIG1; LIG3; POLE3; POLE; POLE2; POLE4; POLD3; POLD1; POLD2; POLD4; POLB; PCNA; MUTYH; UNG; NTHL1; XRCC1; HMGB1; POLL; NEIL2; OGG1; MBD4; MPG; SMUG1; NEIL1; NEIL3; TDG

Nucleotide excision repair	GTF2H4; GTF2H3; GTF2H2; GTF2H2C; GTF2H2C_2; GTF2H1; ERCC2; GTF2H5; ERCC3; CCNH; MNAT1; CDK7; DDB1; CUL4B; CUL4A; RBX1; LIG1; RFC1; RFC2; RFC3; RFC4; RFC5; PCNA; POLE3; POLE; POLE2; POLE4; ERCC4; BIVM-ERCC5; ERCC5; POLD3; POLD1; POLD2; POLD4; RPA4; RPA1; RPA2; RPA3; ERCC1; XPA; DDB2; CETN2; RAD23A; RAD23B; ERCC8; ERCC6; XPC
Calcium signaling pathway	ITPKA; ITPKB; ITPKC; ADCY1; ADCY3; ADCY8; PDE1A; PDE1C; PDE1B; NOS1; NOS2; NOS3; VDAC1; VDAC2; VDAC3; PPIF; SLC8A2; SLC8A1; SLC8A3; CAMK1D; CAMK1G; CAMK4; CAMK2A; CAMK2B; CAMK2D; CAMK2G; CAMK1; PPP3CA; PPP3CB; PPP3CC; PPP3R1; PPP3R2; PRKCA; PRKCB; PRKCG; PTK2B; TPCN2; TPCN1; CD38; PLCE1; LOC102723407; EGFR; ERBB2; ERBB3; ERBB4; PDGFRA; PDGFRB; GNA11; GNA15; GNAQ; GNA14; CHRNA7; P2RX2; GRIN1; GRIN2A; GRIN2C; GRIN2D; P2RX1; P2RX3; P2RX4; P2RX5; P2RX7; P2RX6; PLCG1; PLCG2; SPHK2; SPHK1; PLCZ1; MYLK4; MYLK; MYLK2; MYLK3; PLCD3; PLCD1; PLCD4; ITPR1; ITPR2; ITPR3; PHKA1; PHKA2; PHKB; PHKG1; PHKG2; RYR1; RYR2; RYR3; PLCB1; PLCB2; PLCB3; PLCB4; ORAI2; ORAI1; ORAI3; CACNA1C; CACNA1D; CACNA1F; CACNA1S; CACNA1A; CACNA1B; CACNA1E; CYSLTR1; CHRM1; CHRM2; CHRM3; ADRA1D; ADRA1B; ADRA1A; AGTR1; EDNRA; EDNRB; F2R; GRM1; GRM5; GRPR; HRH1; HTR2A; HTR2B; HTR2C; LHCGR; NTSR1; OXTR; AVPR1A; AVPR1B; LTB4R2; CYSLTR2; PTAFR; PTGER1; PTGER3; PTGFR; BDKRB1; BDKRB2; TACR2; TACR1; TACR3; TBXA2R; TRHR; CXCR4; CCKAR; CCKBR; CALML6; CALML5; CALM1; CALM2; CALM3; CALML3; CALML4; TNNC2; TNNC1; SLC25A4; SLC25A5; SLC25A6; SLC25A31; MCU; ADCY2; ADCY7; ADCY9; ADCY4; ATP2A1; ATP2A2; ATP2A3; CHRM5; ADORA2A; ADORA2B; ADRB1; ADRB2; ADRB3; DRD1; DRD5; HRH2; HTR4; HTR5A; HTR6; HTR7; PLN; PRKACA; PRKACB; PRKACG; GNAL; GNAS; CACNA1I; CACNA1H; CACNA1G; ATP2B1; ATP2B2; ATP2B3; ATP2B4; STIM2; STIM1; CASQ1; CASQ2; TRDN; ASPH; HRC; MCOLN2; MCOLN3; MCOLN1
Sphingolipid signaling pathway	MAPK1; MAPK3; MAP2K1; MAP2K2; RAF1; AKT3; AKT1; AKT2; PIK3CA; PIK3CB; PIK3CD; PIK3R1; PIK3R2; PIK3R3; PDPK1; PRKCA; PRKCB; PRKCG; S1PR1; S1PR2; S1PR3; S1PR4; S1PR5; GNAI1; GNAI2; GNAI3; GNAQ; GNA13; GNA12; HRAS; KRAS; NRAS; PLCB1; PLCB2; PLCB3; PLCB4; RHOA; ROCK1; ROCK2; PTEN; TNF; SPTLC1; SPTLC3; SPTLC2; CERS1; CERS3; CERS6; CERS2; CERS4; CERS5; DEGS2; DEGS1; NSMAF; SMPD2; ACER1; ACER2; ASAH1; ASAH2; SPHK2; SPHK1; ABCC1; CTSD; MAP3K5; PRKCZ; PPP2R3B; PPP2R3C; PPP2CA; PPP2CB; PPP2R1A; PPP2R1B; PPP2R2A; PPP2R2B; PPP2R2C; PPP2R3A; PPP2R5A; PPP2R5B; PPP2R5C; PPP2R5D; PPP2R5E; PPP2R2D; SMPD1; SGPP2; SGPP1; MAPK8; MAPK9; MAPK10; BID; MAPK14; MAPK11; MAPK13; MAPK12; BAX; TP53; BCL2; SGPL1; SGMS2; SGMS1; NOS3; NFKB1; RELA; RAC1; RAC2; RAC3; TNFRSF1A; ADORA1; ADORA3; OPRD1; BDKRB2; FCER1A; MS4A2; FCER1G; PLD1; PLD2; FYN; GAB2; TRADD; TRAF2; PRKCE; KNG1
SNARE interactions in vesicular transport	BNIP1; USE1; STX18; STX17; BET1; BET1L; YKT6; GOSR1; STX5; SEC22B; GOSR2; SNAP29; STX6; STX16; STX19; STX11; VTI1B; VTI1A; STX8; STX7; VAMP4; VAMP5; SNAP23; STX1B; STX2; STX1A; STX3; STX4; VAMP8; VAMP1; VAMP2; VAMP3; VAMP7

Autophagy - other	RPTOR; MTOR; MLST8; ULK2; ATG13; ATG101; IGBP1; PPP2CA; PPP2CB; BECN2; BECN1; PIK3R4; PIK3C3; ATG9B; ATG9A; ATG2A; ATG2B; WIPI2; WIPI1; ATG3; GABARAP; GABARAPL2; GABARAPL1; ATG4A; ATG4B; ATG4C; ATG4D; ATG16L1; ATG12; ATG10; ATG7; ATG5
Phagosome	SEC22B; STX18; VAMP3; FCAR; FCGR1A; FCGR2A; FCGR2B; FCGR3A; FCGR3B; FCGR2C; C1R; ITGAM; ITGB2; ITGAV; ITGB3; ITGB1; PLA2R1; MRC1; MRC2; MSR1; ITGB5; ITGA2; ITGA5; ACTB; ACTG1; PIK3C3; EEA1; RAB5A; RAB5B; RAB5C; RAB7B; RAB7A; CYBB; RILP; CYBA; NCF4; NCF1; NCF2; RAC1; DYNC1H1; DYNC1I1; DYNC1I2; DYNC1LI2; DYNC1LI1; DYNC2H1; TUBA1B; TUBA3E; TUBA3D; TUBA8; TUBA4A; TUBA3C; TUBA1A; TUBAL3; TUBA1C; TUBB3; TUBB4A; TUBB4B; TUBB; TUBB8; TUBB2B; TUBB2A; TUBB1; TUBB6; HGS; M6PR; TCIRG1; ATP6V1G3; ATP6V0E2; ATP6V0A2; ATP6V0D2; ATP6V1C2; ATP6V0A4; ATP6V1D; ATP6V1H; ATP6V1A; ATP6V1B1; ATP6V1B2; ATP6V0C; ATP6V1C1; ATP6V1E1; ATP6V0B; ATP6V1G2; ATP6V0A1; ATP6AP1; ATP6V0E1; ATP6V1E2; ATP6V0D1; ATP6V1F; ATP6V1G1; LAMP1; LAMP2; MPO; PIKFYVE; STX12; STX7; CD36; MARCO; OLR1; CD14; TLR2; TLR6; TLR4; NOS1; SCARB1; LOC102723407; C3; COMP; THBS1; THBS2; THBS3; THBS4; MBL2; SFTPD; SFTPA1; SFTPA2; COLEC11; COLEC12; SEC61B; SEC61G; SEC61A1; SEC61A2; CALR; CANX; HLA-DMA; HLA-DMB; HLA-DOA; HLA-DOB; HLA-DPA1; HLA-DPB1; HLA-DQA1; HLA-DQA2; HLA-DQB1; HLA-DRA; HLA-DRB1; HLA-DRB3; HLA-DRB4; HLA-DRB5; CLEC4M; CD209; CLEC7A; TAP1; TAP2; CTSL; CTSS; HLA-A; HLA-B; HLA-C; HLA-E; HLA-F; HLA-G; TFRC; CORO1A
mTOR signaling pathway	IGF1R; INSR; IRS1; PIK3CA; PIK3CB; PIK3CD; PIK3R1; PIK3R2; PIK3R3; RAF1; BRAF; MAPK1; MAPK3; HRAS; KRAS; NRAS; MAP2K1; MAP2K2; PTEN; PDPK1; AKT3; AKT1; AKT2; TSC1; TSC2; RHEB; EIF4EBP1; LPIN1; LPIN3; LPIN2; RPS6KB1; RPS6KB2; RHOA; PRKCA; PRKCB; PRKCG; SGK1; TBC1D7-LOC100130357; TBC1D7; RRAGB; RRAGA; RRAGD; RRAGC; RPS6KA6; RPS6KA1; RPS6KA2; RPS6KA3; PRKAA1; PRKAA2; GRB2; DDIT4; EIF4E; EIF4E1B; EIF4E2; ULK1; ULK2; RPS6; TNF; IKBKB; TNFRSF1A; SLC7A5; SLC3A2; SOS1; SOS2; NPRL2; NPRL3; DEPDC5; RNF152; SKP2; MIOS; SEC13; WDR59; SEH1L; WDR24; SESN2; ATP6V1G3; ATP6V1C2; ATP6V1D; ATP6V1H; ATP6V1A; ATP6V1B1; ATP6V1B2; ATP6V1C1; ATP6V1E1; ATP6V1G2; ATP6V1E2; ATP6V1F; ATP6V1G1; SLC38A9; LAMTOR5; LAMTOR2; LAMTOR4; LAMTOR1; LAMTOR3; CHUK; IGF1; INS; GRB10; FLCN; FNIP2; FNIP1; EIF4B; MTOR; DEPTOR; MLST8; RPTOR; AKT1S1; TTI1; TELO2; PRR5; RICTOR; MAPKAP1; GSK3B; WNT16; WNT4; WNT1; WNT2; WNT3; WNT5A; WNT6; WNT7A; WNT7B; WNT8A; WNT8B; WNT10B; WNT11; WNT2B; WNT9A; WNT9B; WNT10A; WNT5B; WNT3A; FZD10; FZD2; FZD5; FZD3; FZD1; FZD4; FZD6; FZD7; FZD8; FZD9; LRP6; LRP5; DVL1; DVL2; DVL3; CLIP1; STK11; STRADB; STRADA; CAB39; CAB39L; CASTOR2; CASTOR1
PI3K-Akt signaling pathway	EIF4E; EIF4E1B; EIF4E2; EIF4EBP1; RPS6; EIF4B; RPS6KB1; RPS6KB2; RPTOR; MTOR; MLST8; RHEB; TSC2; TSC1; AKT3; AKT1; AKT2; PDPK1; PIK3CA; PIK3CB; PIK3CD; PIK3R1; PIK3R2; PIK3R3; PTEN; IRS1; CSF1R; EGFR; EPHA2; ERBB2; ERBB3; ERBB4; FGFR1; FGFR3; FGFR2; FGFR4; FLT1; FLT3; FLT4; IGF1R;

	INSR; KDR; KIT; MET; NGFR; NTRK1; NTRK2; PDGFRA; PDGFRB; TEK; LPAR6; CHRM1; CHRM2; LPAR1; F2R; LPAR3; LPAR4; LPAR5; LPAR2; GSK3B; MYC; CDKN1B; RBL2; FASLG; BCL2L11; FOXO3; BAD; MDM2; BCL2L1; TP53; GYS1; GYS2; CCND1; PCK1; PCK2; G6PC; G6PC2; G6PC3; CHUK; IKBKB; IKBKG; NFKB1; RELA; YWHAQ; YWHAB; YWHAE; YWHAG; YWHAH; YWHAZ; CSF1; EFNA1; EFNA2; EFNA3; EFNA4; EFNA5; EGF; EREG; FGF1; FGF2; FGF3; FGF4; FGF5; FGF6; FGF7; FGF8; FGF9; FGF10; VEGFD; FLT3LG; FGF20; FGF21; FGF22; ANGPT1; ANGPT2; HGF; IGF1; IGF2; INS; AREG; KITLG; NGF; NTF3; NTF4; ANGPT4; PDGFA; PDGFB; PGF; PDGFC; BDNF; TGFA; VEGFA; VEGFB; VEGFC; PDGFD; FGF23; FGF18; FGF17; FGF16; FGF19; GRB2; SOS1; SOS2; HRAS; KRAS; NRAS; THEM4; PPP2R3B; PPP2R3C; PPP2CA; PPP2CB; PPP2R1A; PPP2R1B; PPP2R2A; PPP2R2B; PPP2R2C; PPP2R3A; PPP2R5A; PPP2R5B; PPP2R5C; PPP2R5D; PPP2R5E; PPP2R2D; HSP90AA1; HSP90AB1; HSP90B1; CDC37; NOS3; PRKAA1; PRKAA2; DDIT4; STK11; CRTC2; PHLPP2; PHLPP1; CDKN1A; CDK2; CDK4; CDK6; CASP9; PIK3R6; PIK3R5; PIK3CG; RAF1; MAP2K1; MAP2K2; MAPK1; MAPK3; LOC102723407; CD19; SYK; GNB5; GNB1; GNB2; GNB3; GNG3; GNG4; GNG5; GNG7; GNG10; GNG11; GNGT1; GNGT2; GNG13; GNG2; GNG12; GNB4; GNG8; ITGA11; ITGA6; ITGA1; ITGA2; ITGA2B; ITGA3; ITGA4; ITGA5; ITGA7; ITGA9; ITGAV; ITGA10; ITGA8; ITGB1; ITGB3; ITGB4; ITGB5; ITGB6; ITGB7; ITGB8; LAMC3; CHAD; COL1A1; COL1A2; COL2A1; COL4A1; COL4A2; COL4A3; COL4A4; COL4A5; COL4A6; COL6A1; COL6A2; COL6A3; COL9A1; COL9A2; COL9A3; COMP; COL6A6; LAMB4; FN1; COL6A5; LAMA1; TNC; IBSP; LAMA2; LAMA3; LAMA4; LAMA5; LAMB1; LAMB2; LAMB3; LAMC1; LAMC2; RELN; TNN; SPP1; THBS1; THBS2; THBS3; THBS4; TNR; TNXB; VTN; VWF; PTK2; TLR2; TLR4; RAC1; CSF3R; EPOR; GHR; IFNAR1; IFNAR2; IL2RA; IL2RB; IL2RG; IL3RA; IL4R; IL6R; IL7R; PRLR; OSMR; JAK1; JAK2; JAK3; CSF3; CSH1; CSH2; EPO; GH1; GH2; IFNA1; IFNA2; IFNA4; IFNA5; IFNA6; IFNA7; IFNA8; IFNA10; IFNA13; IFNA14; IFNA16; IFNA17; IFNA21; IFNB1; IL2; IL3; IL4; IL6; IL7; OSM; PRL; BRCA1; CREB3; CREB1; ATF2; ATF6B; CREB3L4; ATF4; CREB3L2; CREB3L3; CREB3L1; CREB5; BCL2; MCL1; MYB; PRKCA; PKN3; PKN1; PKN2; C8orf44-SGK3; SGK2; SGK3; SGK1; PIK3AP1; CCND2; CCND3; CCNE1; CCNE2; MTCP1; TCL1A; TCL1B; NR4A1; RXRA
Notch signaling pathway	NCSTN; APH1A; APH1B; PSEN1; PSEN2; HDAC1; HDAC2; CIR1; CTBP1; CTBP2; NCOR2; PTCRA; HES1; HES5; RBPJL; RBPJ; SNW1; CREBBP; EP300; KAT2A; KAT2B; MAML3; MAML2; MAML1; ADAM17; PSENEN; DTX2; DTX3L; DTX1; DTX3; DTX4; NUMB; NUMBL; JAG1; JAG2; NOTCH1; NOTCH2; NOTCH3; NOTCH4; DVL1; DVL2; DVL3; LFNG; MFNG; RFNG; DLL3; DLL1; DLL4; ATXN1L; ATXN1
Hedgehog signaling pathway	SMO; GPR161; TPTEP2-CSNK1E; CSNK1A1L; CSNK1A1; CSNK1D; CSNK1E; CSNK1G2; CSNK1G3; CSNK1G1; GSK3B; PRKACA; PRKACB; PRKACG; GLI1; GLI2; GLI3; KIF7; CUL1; FBXW11; BTRC; PTCH1; PTCH2; HHIP; SUFU; GAS1; LRP2; CDON; BOC; SMURF1; SMURF2; IHH; DHH; SHH; EVC2; EVC; GRK2; GRK3; ARRB1; ARRB2; KIF3A; SPOPL; SPOP; CUL3; CCND1; CCND2; BCL2; MEGF8; MGRN1; MOSMO

TGF-beta signaling pathway	LTBP1; THBS1; CUL1; RBX1; SMAD4; ACVR2A; ACVR2B; ACVR1C; ACVR1B; SMAD2; SMAD3; NODAL; SMAD6; SMAD7; PITX2; SP1; CREBBP; EP300; CDKN2B; MYC; TFDP1; E2F4; E2F5; RBL1; ID1; ID2; ID3; ID4; ACVR1; INHBA; INHBB; INHBC; INHBE; FST; LEFTY1; LEFTY2; RPS6KB1; RPS6KB2; PPP2CA; PPP2CB; PPP2R1A; PPP2R1B; ROCK1; RHOA; ZFYVE9; ZFYVE16; SKP1; MAPK1; MAPK3; SMURF1; SMURF2; TGFBR2; TGFBR1; TGFB1; TGFB2; TGFB3; DCN; TNF; IFNG; AMHR2; BMPR2; BMPR1A; BMPR1B; GDF7; AMH; BMP8A; GDF6; BMP2; BMP4; BMP5; BMP6; BMP7; BMP8B; GDF5; NOG; MICOS10-NBL1; NBL1; SMAD1; SMAD5; SMAD9; CHRD; BAMBI; TGIF2; TGIF1; FMOD; HJV; NEO1; RGMB; RGMA; HAMP; GREM1; GREM2; THSD4; FBN1
Gap junction	CSNK1D; PRKCA; PRKCB; PRKCG; GNAI1; GNAI2; GNAI3; GJA1; GJD2; MAPK7; MAP3K2; MAP2K5; PRKG1; PRKG2; HTR2A; HTR2B; HTR2C; CDK1; LPAR1; PRKACA; PRKACB; PRKACG; TJP1; TUBB3; TUBB4A; TUBB4B; TUBB; TUBB8; TUBB2B; TUBB2A; TUBB1; TUBB6; ADCY1; ADCY2; ADCY3; ADCY5; ADCY6; ADCY7; ADCY8; ADCY9; ADCY4; SRC; TUBA1B; TUBA3E; TUBA3D; TUBA8; TUBA4A; TUBA3C; TUBA1A; TUBAL3; TUBA1C; GUCY1A2; GUCY1A1; GUCY1B1; MAPK1; MAPK3; MAP2K1; MAP2K2; RAF1; HRAS; KRAS; NRAS; SOS1; SOS2; GRB2; EGFR; PDGFRA; PDGFRB; EGF; PDGFA; PDGFB; PDGFC; PDGFD; ADRB1; GNAS; GNA11; GNAQ; GRM1; GRM5; DRD2; PLCB1; PLCB2; PLCB3; PLCB4; DRD1
Antigen processing and presentation	CD74; HLA-DMA; HLA-DMB; HLA-DOA; HLA-DOB; HLA-DPA1; HLA-DPB1; HLA-DQA1; HLA-DQA2; HLA-DQB1; HLA-DRA; HLA-DRB1; HLA-DRB3; HLA-DRB4; HLA-DRB5; NFYA; NFYB; NFYC; RFX5; RFXAP; RFXANK; CREB1; CIITA; CTSB; CTSL; CTSS; LGMN; IFI30; CD4; KIR2DS2; KIR3DL3; KIR2DL1; KIR2DL2; KIR2DL3; KIR2DL4; KIR2DS1; KIR2DS3; KIR2DS4; KIR2DS5; KIR3DL1; KIR3DL2; KLRC1; KLRC2; KLRC3; KLRD1; KIR2DL5A; KLRC4; CD8A; CD8B; CD8B2; TAPBP; CALR; PDIA3; B2M; HLA-A; HLA-B; HLA-C; HLA-E; HLA-F; HLA-G; TAP1; TAP2; CANX; HSPA5; HSP90AA1; HSP90AB1; HSPA1A; HSPA1B; HSPA1L; HSPA2; HSPA4; HSPA6; HSPA8; PSME3; PSME1; PSME2; TNF; IFNG
Toll-like receptor signaling pathway	IFNB1; CHUK; TRAF6; IRAK1; IRAK4; MYD88; MAPK1; MAPK3; MAP3K8; NFKB1; SPP1; TRAF3; RIPK1; IRF7; IRF5; CXCL11; CXCL10; IFNA1; IFNA2; IFNA4; IFNA5; IFNA6; IFNA7; IFNA8; IFNA10; IFNA13; IFNA14; IFNA16; IFNA17; IFNA21; CD86; CD40; IKBKB; MAP3K7; MAPK14; MAPK11; MAPK13; MAPK12; CXCL8; TIRAP; RAC1; MAPK8; MAPK9; MAPK10; IL1B; TBK1; IKBKE; FADD; IL6; CCL5; TAB1; AKT3; AKT1; AKT2; TAB2; STAT1; NFKBIA; TNF; IRF3; CD80; TLR2; LY96; TLR5; TLR3; TLR1; TLR6; LBP; TLR9; TLR4; TICAM2; TOLLIP; TICAM1; CD14; IKBKG; CASP8; CCL3L3; CCL3; CCL3L1; CXCL9; PIK3CA; PIK3CB; PIK3CD; PIK3R1; PIK3R2; PIK3R3; IFNAR1; IFNAR2; TLR7; TLR8; MAP2K7; MAP2K4; RELA; MAP2K3; MAP2K6; MAP2K1; MAP2K2; FOS; JUN; IL12A; IL12B; CCL4L1; CCL4; CCL4L2; CTSK
JAK-STAT signaling pathway	MYC; AKT3; AKT1; AKT2; PIK3CA; PIK3CB; PIK3CD; PIK3R1; PIK3R2; PIK3R3; SOS1; SOS2; GRB2; CISH; BCL2; SOCS4; SOCS7; SOCS1; SOCS2; SOCS3; SOCS6; SOCS5; CREBBP; EP300; IRF9; STAT1; STAT2; STAT3; STAT4; STAT5A; STAT5B; STAT6; PIAS3; PIAS4; PIAS1; PIAS2; PTPN6; STAM2; STAM; JAK1; JAK2; JAK3;

	TYK2; FHL1; IL22RA2; CNTFR; CSF2RA; CSF2RB; CSF3R; IL23R; IFNLR1; EGFR; EPOR; GHR; IFNAR1; IFNAR2; IFNGR1; IFNGR2; IL2RA; IL2RB; IL2RG; IL3RA; IL4R; IL5RA; IL6R; IL6ST; IL7R; IL9R; IL10RA; IL10RB; IL11RA; IL12RB1; IL12RB2; IL13RA1; IL13RA2; IL15RA; LEPR; LIFR; MPL; IL21R; PDGFRA; PDGFRB; IL20RA; IL20RB; PRLR; IL22RA1; CRLF2; OSMR; IL27RA; PTPN2; MCL1; PIM1; CCND1; CCND2; CCND3; PTPN11; CDKN1A; BCL2L1; HRAS; RAF1; AOX1; MTOR; GFAP; IL24; CSF2; CTF1; IFNL2; IFNL3; IFNL1; IL19; IFNE; IFNA1; IFNA2; IFNA4; IFNA5; IFNA6; IFNA7; IFNA8; IFNA10; IFNA13; IFNA14; IFNA16; IFNA17; IFNA21; IFNB1; IFNG; IFNW1; IL2; IL3; IL4; IL5; IL6; IL7; IL9; IL10; IL11; IL12A; IL12B; IL13; IL15; LIF; OSM; IL20; IL22; IL23A; IL17D; IFNK; IL21; TSLP; CNTF; CSF3; CSH1; CSH2; EPO; GH1; GH2; LEP; PRL; THPO
Th1 and Th2 cell differentiation	IFNG; IL5; NFKBIA; NFKBIB; NFKBIE; NFKB1; RELA; CHUK; IKBKB; IKBKG; PRKCQ; NFATC1; NFATC2; NFATC3; PPP3CA; PPP3CB; PPP3CC; PPP3R1; PPP3R2; ZAP70; CD3E; CD3D; CD247; CD3G; MAPK1; MAPK3; MAPK14; MAPK11; MAPK13; MAPK12; MAPK8; MAPK9; MAPK10; JUN; LAT; FOS; PLCG1; CD4; LCK; IFNGR1; IFNGR2; STAT1; IL12A; IL12B; IL12RB1; IL12RB2; STAT4; IL4; IL4R; STAT6; GATA3; TBX21; IL13; IL2; IL2RA; IL2RB; IL2RG; STAT5A; STAT5B; MAF; JAG1; JAG2; NOTCH1; NOTCH2; MAML3; MAML2; MAML1; RBPJL; RBPJ; DLL3; DLL1; DLL4; NOTCH3; HLA-DMA; HLA-DMB; HLA-DOA; HLA-DOB; HLA-DPA1; HLA-DPB1; HLA-DQA1; HLA-DQA2; HLA-DQB1; HLA-DRA; HLA-DRB1; HLA-DRB3; HLA-DRB4; HLA-DRB5; JAK2; TYK2; JAK1; JAK3; RUNX3
Th17 cell differentiation	NFKBIA; NFKBIB; NFKBIE; NFKB1; RELA; CHUK; IKBKB; IKBKG; PRKCQ; NFATC1; NFATC2; NFATC3; PPP3CA; PPP3CB; PPP3CC; PPP3R1; PPP3R2; ZAP70; MAPK1; MAPK3; MAPK14; MAPK11; MAPK13; MAPK12; MAPK8; MAPK9; MAPK10; JUN; LAT; FOS; PLCG1; CD4; LCK; IFNG; IFNGR1; IFNGR2; STAT1; IL4; IL2RG; IL4R; STAT6; IL2; IL2RA; IL2RB; STAT5A; STAT5B; HLA-DMA; HLA-DMB; HLA-DOA; HLA-DOB; HLA-DPA1; HLA-DPB1; HLA-DQA1; HLA-DQA2; HLA-DQB1; HLA-DRA; HLA-DRB1; HLA-DRB3; HLA-DRB4; HLA-DRB5; IL1R1; IL1RAP; IL21R; IL6R; IL6ST; IL23R; IL12RB1; STAT3; MTOR; TGFBR1; TGFBR2; IL21; IL17A; IL17F; IL22; RORC; RORA; IRF4; TBX21; RUNX1; GATA3; RARA; RXRA; RXRB; RXRG; FOXP3; TGFB1; SMAD2; SMAD3; SMAD4; IL27RA; IL1B; IL17D; IL6; IL23A; HSP90AA1; HSP90AB1; AHR; CD3E; CD3G; CD3D; CD247; HIF1A; JAK1; JAK3; JAK2; TYK2
Progesterone-mediated oocyte maturation	IGF1R; IGF1; INS; MOS; CPEB2; CPEB3; CPEB1; CPEB4; ADCY1; ADCY2; ADCY3; ADCY5; ADCY6; ADCY7; ADCY8; ADCY9; ADCY4; CCNB1; CDK1; KRAS; PIK3CA; PIK3CB; PIK3CD; PIK3R1; PIK3R2; PIK3R3; AKT3; AKT1; AKT2; PDE3B; ANAPC10; CDC26; ANAPC13; ANAPC2; ANAPC4; ANAPC5; ANAPC7; ANAPC11; ANAPC1; CDC23; CDC16; CDC27; FZR1; MAD2L2; MAD2L1; MAD1L1; BUB1; CDC25A; CDC25B; CDC25C; PLK1; CCNB3; CCNB2; HSP90AA1; HSP90AB1; PRKACA; PRKACB; PRKACG; PKMYT1; RPS6KA6; RPS6KA1; RPS6KA2; RPS6KA3; MAPK1; MAPK3; MAP2K1; GNAI1; GNAI2; GNAI3; CDK2; CCNA2; CCNA1; SPDYE2B; SPDYE18; SPDYE11; SPDYE16; SPDYE17; SPDYE15; SPDYA; SPDYE1; SPDYC; SPDYE4; SPDYE3; SPDYE2; SPDYE5; SPDYE6; AURKA; MAPK14; MAPK8; MAPK11; MAPK9; MAPK10; MAPK13; MAPK12; PGR; ARAF;

	RAF1; BRAF; STK10; KIF22
Prolactin signaling pathway	AKT3; AKT1; AKT2; PIK3CA; PIK3CB; PIK3CD; PIK3R1; PIK3R2; PIK3R3; MAPK1; MAPK3; MAP2K1; MAP2K2; RAF1; HRAS; KRAS; NRAS; PRL; PRLR; JAK2; STAT1; STAT3; STAT5A; STAT5B; SHC2; SHC4; SHC3; SHC1; SRC; GRB2; SOS1; SOS2; FOXO3; MAPK8; MAPK9; MAPK10; MAPK14; MAPK11; MAPK13; MAPK12; GALT; SOCS4; SOCS7; SOCS1; SOCS2; SOCS3; SOCS6; SOCS5; CSN2; ELF5; CISH; TNFSF11; IRF1; TH; SLC2A2; GCK; INS; CCND2; LHCGR; ESR1; ESR2; CGA; LHB; CYP17A1; CCND1; GSK3B; FOS; TNFRSF11A; NFKB1; RELA
Growth hormone synthesis	secretion and action
Thyroid hormone signaling pathway	ITGB3; ITGAV; AKT3; AKT1; AKT2; PDPK1; PIK3CA; PIK3CB; PIK3CD; PIK3R1; PIK3R2; PIK3R3; PRKCA; PRKCB; PRKCG; CTNNB1; GSK3B; PLCD3; PLCB1; PLCE1; PLCB2; PLCB3; PLCB4; PLCD1; PLCG1; PLCG2; PLCD4; PLCZ1; HRAS; KRAS; NRAS; RAF1; MAP2K1; MAP2K2; STAT1; THRA; THRB; MTOR; GATA4; RCAN1; HIF1A; MYH6; MYH7; ATP2A1; ATP2A2; ATP2A3; PLN; PRKACA; PRKACB; PRKACG; MAPK1; MAPK3; TP53; ESR1; RCAN2; SLC2A1; TBC1D4; PFKFB2; SLC9A1; ATP1B4; ATP1A1; ATP1A2; ATP1A3; ATP1A4; ATP1B1; ATP1B2; ATP1B3; FXYD2; ACTB; ACTG1; DIO1; DIO2; DIO3; SLC16A10; SLC16A2; SLCO1C1; RXRA; RXRB; RXRG; NCOR1; SIN3A; HDAC1; HDAC2; HDAC3; KAT2A; KAT2B; NCOA2; NCOA3; NCOA1; CREBBP; EP300; MED16; MED12L; MED13L; MED4; MED30; MED14; MED17; MED27; MED24; MED12; MED13; MED1; MDM2; TSC2; RHEB; BAD; FOXO1; CASP9; PFKL; PFKM; PFKP; SRC; WNT4; NOTCH1; NOTCH2; NOTCH3; NOTCH4; BMP4; CCND1; MYC
Melanogenesis	DVL1; DVL2; DVL3; DCT; TYRP1; TYR; MITF; PRKACA; PRKACB; PRKACG; CAMK2A; CAMK2B; CAMK2D; CAMK2G; CALML6; CALML5; CALM1; CALM2; CALM3; CALML3; CALML4; GNAI1; GNAI2; GNAI3; GNAO1; EDNRB; KIT; KITLG; FZD10; FZD2; FZD5; FZD3; FZD1; FZD4; FZD6; FZD7; FZD8; FZD9; PLCB1; PLCB2; PLCB3; PLCB4; ASIP; CREBBP; EP300; LEF1; TCF7; TCF7L2; TCF7L1; GNAS; PRKCB; CREB3; CREB1; CREB3L4; CREB3L2; CREB3L3; CREB3L1; ADCY1; ADCY2; ADCY3; ADCY5; ADCY6; ADCY7; ADCY8; ADCY9; ADCY4; PRKCA; PRKCG; GSK3B; CTNNB1; POMC; GNAQ; MC1R; WNT16; WNT4; WNT1; WNT2; WNT3; WNT5A; WNT6; WNT7A; WNT7B; WNT8A; WNT8B; WNT10B; WNT11; WNT2B; WNT9A; WNT9B; WNT10A; WNT5B; WNT3A; MAPK1; MAPK3; MAP2K1; MAP2K2; RAF1; HRAS; KRAS; NRAS; EDN1
Renin-angiotensin system	ACE2; ACE; AGTR1; AGTR2; NLN; THOP1; MME; ANPEP; ENPEP; CTSA; CPA3; MAS1; LNPEP; CMA1; CTSG; AGT; REN; ATP6AP2; KLK1; KLK2; MRGPRD; PREP; PRCP
Aldosterone synthesis and secretion	ITPR1; ITPR2; ITPR3; KCNK3; KCNK9; PLCB1; PLCB2; PLCB3; PLCB4; CACNA1C; CACNA1D; CACNA1F; CACNA1S; GNA11; GNAQ; CACNA1I; CACNA1H; CACNA1G; CREB3; CREB1; ATF2; ATF6B; CREB3L4; ATF4; CREB3L2; CREB3L3; CREB3L1; CREB5; ADCY1; ADCY2; ADCY3; ADCY5; ADCY6; ADCY7; ADCY8; ADCY9; ADCY4; PDE2A; CALML6; CALML5; CALM1; CALM2; CALM3; CALML3; CALML4; AGTR1; MC2R; GNAS; PRKCA; PRKCB; PRKCE; PRKCG; PRKD3; PRKD2; PRKD1; CAMK1D; CAMK1G; CAMK4; CAMK2A; CAMK2B; CAMK2D; CAMK2G; CAMK1; NR4A2; NR4A1; CYP11B2; STAR; NPR1; HSD3B1;

	HSD3B2; PRKACA; PRKACB; PRKACG; ORAI1; DAGLB; DAGLA; LDLR; SCARB1; LIPE; ATF1; CYP11A1; CYP21A2; KCNJ5; ATP1B4; ATP1A1; ATP1A2; ATP1A3; ATP1A4; ATP1B1; ATP1B2; ATP1B3; ATP2B1; ATP2B2; ATP2B3; ATP2B4; POMC; AGT; NPPA
Adrenergic signaling in cardiomyocytes	CACNG3; CACNG2; CACNG5; CACNG4; CACNA2D3; CACNG8; CACNG7; CACNG6; CACNA1C; CACNA1D; CACNA1F; CACNA1S; CACNA2D1; CACNB1; CACNB2; CACNB3; CACNB4; CACNG1; CACNA2D2; CACNA2D4; RYR2; SLC8A2; SLC8A1; SLC8A3; ATP2A1; ATP2A2; ATP2A3; TNNC1; TNNT2; TNNI3; ACTC1; TPM1; TPM2; TPM3; TPM4; MYH6; MYH7; MYL2; MYL3; MYL4; ATP1B4; ATP1A1; ATP1A2; ATP1A3; ATP1A4; ATP1B1; ATP1B2; ATP1B3; FXYD2; SLC9A1; ADRB1; ADRB2; GNAS; GNAI1; GNAI2; GNAI3; ADCY1; ADCY2; ADCY3; ADCY5; ADCY6; ADCY7; ADCY8; ADCY9; ADCY4; PRKACA; PRKACB; PRKACG; PIK3R6; PIK3R5; PIK3CG; AKT3; AKT1; AKT2; MAPK1; MAPK3; CREB3; CREB1; ATF2; ATF6B; CREB3L4; ATF4; CREB3L2; CREB3L3; CREB3L1; CREB5; PLN; SCN1B; SCN4B; SCN5A; SCN7A; PPP1R1A; PPP1CA; PPP1CB; PPP1CC; PPP2R3B; PPP2R3C; PPP2CA; PPP2CB; PPP2R1A; PPP2R1B; PPP2R2A; PPP2R2B; PPP2R2C; PPP2R3A; PPP2R5A; PPP2R5B; PPP2R5C; PPP2R5D; PPP2R5E; PPP2R2D; CALML6; CALML5; CALM1; CALM2; CALM3; CALML3; CALML4; CAMK2A; CAMK2B; CAMK2D; CAMK2G; AGTR1; AGTR2; GNAQ; PLCB1; PLCB2; PLCB3; PLCB4; PRKCA; RAPGEF3; RAPGEF4; MAPK14; MAPK11; MAPK13; MAPK12; ADRA1D; ADRA1B; ADRA1A; CREM; BCL2; RPS6KA5; ATP2B1; ATP2B2; ATP2B3; ATP2B4; KCNE1B; KCNE1; KCNQ1; AGT
Carbohydrate digestion and absorption	SLC5A1; SLC2A5; SLC2A2; ATP1B4; ATP1A1; ATP1A2; ATP1A3; ATP1A4; ATP1B1; ATP1B2; ATP1B3; FXYD2; SLC37A4; LCT; SI; MGAM; MGAM2; AMY1A; AMY1B; AMY1C; AMY2A; AMY2B; CACNA1D; HK1; HK2; HK3; HKDC1; G6PC; G6PC2; G6PC3; PIK3CA; PIK3CB; PIK3CD; PIK3R1; PIK3R2; PIK3R3; AKT3; AKT1; AKT2; TAS1R2; TAS1R3; GNAT3; PLCB1; PLCB2; PLCB3; PLCB4; PRKCB
Fat digestion and absorption	ABCG5; ABCG8; NPC1L1; CD36; APOB; CEL; PLA2G2D; PLA2G2E; PLA2G2C; PLA2G3; PLA2G1B; PLA2G2A; PLA2G5; PNLIP; PNLIPRP1; PNLIPRP2; PLA2G2F; PLA2G12A; PLA2G10; PLA2G12B; LIPF; AGPAT1; AGPAT2; MOGAT3; MOGAT2; DGAT2; DGAT1; ACAT1; ACAT2; MTTP; APOA4; APOA1; ABCA1; CLPS; PLPP1; PLPP2; PLPP3; SLC27A4; SLC27A1; SCARB1; FABP2; FABP1; GOT2
Dopaminergic synapse	DRD5; SLC6A3; SLC18A1; SLC18A2; GNAL; GNAS; ADCY5; PRKACA; PRKACB; PRKACG; GNAQ; PLCB1; PLCB2; PLCB3; PLCB4; ITPR1; ITPR2; ITPR3; PRKCA; PRKCB; PRKCG; DDC; MAOA; MAOB; CACNA1A; CACNA1B; DRD2; GNB5; GNAI1; GNAI2; GNAI3; GNAO1; GNB1; GNB2; GNB3; GNG3; GNG4; GNG5; GNG7; GNG10; GNG11; GNGT1; GNGT2; GNG13; GNG2; GNG12; GNB4; GNG8; AKT3; AKT1; AKT2; GSK3A; GSK3B; CREB3; CREB1; ATF2; ATF6B; CREB3L4; ATF4; CREB3L2; CREB3L3; CREB3L1; CREB5; PPP1R1B; MAPK14; MAPK8; MAPK11; MAPK9; MAPK10; MAPK13; MAPK12; TH; DRD1; DRD3; DRD4; COMT; LRTOMT; FOS; PPP3CA; PPP3CB; PPP3CC; CAMK2A; CAMK2B; CAMK2D; CAMK2G; ARRB1; ARRB2; PPP2R3B; PPP2R3C; PPP2CA; PPP2CB; PPP2R1A; PPP2R1B; PPP2R2A; PPP2R2B; PPP2R2C; PPP2R3A; PPP2R5A; PPP2R5B; PPP2R5C; PPP2R5D; PPP2R5E; PPP2R2D; PPP1CA; PPP1CB; PPP1CC; KCNJ3; KCNJ5; KCNJ6;

	KCNJ9; SCN1A; CALML6; CALML5; CALM1; CALM2; CALM3; CALML3; CALML4; CACNA1C; CACNA1D; KIF5A; KIF5B; KIF5C; CLOCK; ARNTL; GRIA1; GRIA2; GRIA3; GRIA4; GRIN2A; GRIN2B; CALY
Serotonergic synapse	HTR4; HTR6; HTR7; HTR2A; HTR2B; HTR2C; SLC18A1; SLC18A2; GNAS; ADCY5; PRKACA; PRKACB; PRKACG; GNAQ; PLCB1; PLCB2; PLCB3; PLCB4; ITPR1; ITPR2; ITPR3; PRKCA; PRKCB; PRKCG; DDC; SLC6A4; CACNA1A; CACNA1B; HTR1A; HTR1B; HTR1D; HTR1E; HTR1F; GNB5; GNAI1; GNAI2; GNAI3; GNAO1; GNB1; GNB2; GNB3; GNG3; GNG4; GNG5; GNG7; GNG10; GNG11; GNGT1; GNGT2; GNG13; GNG2; GNG12; GNB4; GNG8; RAPGEF3; TPH2; TPH1; HTR5A; MAOA; MAOB; PLA2G4B; PLA2G4E; PLA2G4F; PLA2G4D; PLA2G4A; PLA2G4C; JMJD7-PLA2G4B; CACNA1C; CACNA1D; CACNA1F; CACNA1S; HTR3C; HTR3D; HTR3E; HTR3A; HTR3B; TRPC1; LOC107987478; LOC107987479; CYP2C19; CYP2C8; CYP2C9; CYP2C18; CYP2D7; CYP2D6; CYP2J2; CYP4X1; ALOX12; ALOX5; ALOX12B; ALOX15; ALOX15B; PTGS1; PTGS2; CASP3; MAPK1; MAPK3; DUSP1; KCNJ3; KCNJ5; KCNJ6; KCNJ9; HRAS; KRAS; NRAS; ARAF; RAF1; BRAF; MAP2K1; APP; KCND2; KCNN2; GABRB1; GABRB2; GABRB3
Retrograde endocannabinoid signaling	SLC17A8; SLC17A7; SLC17A6; GNAQ; PLCB1; PLCB2; PLCB3; PLCB4; ITPR1; ITPR2; ITPR3; PRKCA; PRKCB; PRKCG; CACNA1A; CACNA1B; CACNA1C; CACNA1D; CACNA1F; CACNA1S; PTGS2; GABRR3; GABRA1; GABRA2; GABRA3; GABRA4; GABRA5; GABRA6; GABRB1; GABRB2; GABRB3; GABRD; GABRE; GABRG1; GABRG2; GABRG3; GABRP; GABRR1; GABRR2; GABRQ; CNR1; GNB5; GNAI1; GNAI2; GNAI3; GNAO1; GNB1; GNB2; GNB3; GNG3; GNG4; GNG5; GNG7; GNG10; GNG11; GNGT1; GNGT2; GNG13; GNG2; GNG12; GNB4; GNG8; GRM1; GRM5; DAGLB; DAGLA; MGLL; ABHD6; SLC32A1; GRIA1; GRIA2; GRIA3; GRIA4; KCNJ3; KCNJ5; KCNJ6; KCNJ9; ADCY1; ADCY2; ADCY3; ADCY5; ADCY6; ADCY7; ADCY8; ADCY9; ADCY4; PRKACA; PRKACB; PRKACG; FAAH; NAPEPLD; MAPK14; MAPK1; MAPK3; MAPK8; MAPK11; MAPK9; MAPK10; MAPK13; MAPK12; RIMS1; NDUFC2-KCTD14; NDUFA11; NDUFS7; ND1; ND2; ND3; ND4; ND4L; ND5; ND6; NDUFA1; NDUFA2; NDUFA3; NDUFA4; NDUFA5; NDUFA6; NDUFA7; NDUFA8; NDUFA9; NDUFA10; NDUFAB1; NDUFB1; NDUFB2; NDUFB3; NDUFB4; NDUFB5; NDUFB6; NDUFB7; NDUFB8; NDUFB9; NDUFB10; NDUFC1; NDUFC2; NDUFS1; NDUFS2; NDUFS3; NDUFV1; NDUFS4; NDUFS5; NDUFS6; NDUFS8; NDUFV2; NDUFV3; NDUFA13; NDUFB11; NDUFA12; NDUFA4L2
Phototransduction	CNGB1; CNGA1; RHO; PDE6A; PDE6G; PDE6B; GNAT1; GNAT2; GNB1; GNGT1; GUCY2F; GUCY2D; SLC24A1; GUCA1A; GUCA1B; GUCA1C; RCVRN; GRK7; GRK1; SAG; RGS9; CALML6; CALML5; CALM1; CALM2; CALM3; CALML3; CALML4
Acute myeloid leukemia	RARA; ZBTB16; KRAS; NRAS; FLT3; SPI1; CEBPA; RUNX1; RUNX1T1; PPARD; MYC; NFKB1; RELA; STAT3; STAT5A; STAT5B; JUP; CCNA2; CCNA1; CCND1; LEF1; TCF7; TCF7L2; TCF7L1; PML; PIM1; HRAS; CHUK; IKBKB; IKBKG; BAD; PIM2; RPS6KB1; RPS6KB2; SOS1; SOS2; GRB2; KIT; MTOR; EIF4EBP1; AKT3; AKT1; AKT2; PIK3CA; PIK3CB; PIK3CD; PIK3R1; PIK3R2; PIK3R3; MAPK1; MAPK3; MAP2K1; MAP2K2; ARAF; RAF1; BRAF; CEBPE; BCL2A1; PER2; CD14;

	ITGAM; FCGR1A; DUSP6; CSF1R; MPO; CSF2; IL3
Chronic myeloid leukemia	MECOM; RUNX1; BCL2L1; PTPN11; ABL1; BCR; TP53; TGFB1; TGFB2; TGFB3; HDAC1; HDAC2; CTBP1; CTBP2; MYC; TGFBR2; TGFBR1; STAT5A; STAT5B; SMAD4; NFKB1; RELA; NFKBIA; CDKN1B; MDM2; CHUK; IKBKB; IKBKG; BAD; CDKN2A; CDKN1A; SOS1; SOS2; GRB2; SHC2; SHC4; SHC3; SHC1; GAB2; CRK; CRKL; CBL; E2F1; E2F2; E2F3; RB1; SMAD3; CDK4; CDK6; CCND1; AKT3; AKT1; AKT2; PIK3CA; PIK3CB; PIK3CD; PIK3R1; PIK3R2; PIK3R3; MAPK1; MAPK3; MAP2K1; MAP2K2; ARAF; RAF1; BRAF; HRAS; KRAS; NRAS; GADD45G; GADD45A; GADD45B; BAX; BAK1; DDB2; POLK
Basal cell carcinoma	KIF7; GLI1; PTCH1; PTCH2; BMP2; BMP4; WNT16; WNT4; WNT1; WNT2; WNT3; WNT5A; WNT6; WNT7A; WNT7B; WNT8A; WNT8B; WNT10B; WNT11; WNT2B; WNT9A; WNT9B; WNT10A; WNT5B; WNT3A; SHH; APC2; APC; SUFU; AXIN1; AXIN2; DVL1; DVL2; DVL3; FZD10; FZD2; FZD5; FZD3; FZD1; FZD4; FZD6; FZD7; FZD8; FZD9; GLI2; GLI3; SMO; HHIP; CTNNB1; GSK3B; LEF1; TCF7; TCF7L2; TCF7L1; TP53; CDKN1A; GADD45G; GADD45A; GADD45B; BAX; BAK1; DDB2; POLK
Bladder cancer	HRAS; MMP1; MMP2; MMP9; TYMP; VEGFA; CXCL8; THBS1; EGFR; CDH1; ERBB2; CDKN2A; CDKN1A; MYC; RASSF1; DAPK1; DAPK3; DAPK2; MDM2; TP53; E2F1; E2F2; E2F3; RB1; FGFR3; CDK4; CCND1; RPS6KA5; EGF; MAPK1; MAPK3; MAP2K1; MAP2K2; ARAF; RAF1; BRAF; KRAS; NRAS; UPK3A; HBEGF; SRC
Prostate cancer	SRD5A2; AR; NFKB1; RELA; NFKBIA; CDKN1B; PTEN; NKX3-1; KLK3; BCL2; CCND1; LEF1; TCF7; TCF7L2; TCF7L1; CREBBP; EP300; MDM2; CDKN1A; FOXO1; CDK2; CTNNB1; E2F1; E2F2; E2F3; HRAS; KRAS; NRAS; TP53; EGFR; ERBB2; FGFR1; FGFR2; IGF1R; INSRR; PDGFRA; PDGFRB; CHUK; IKBKB; IKBKG; BAD; CASP9; CREB3; CREB1; CREB3L4; ATF4; CREB3L2; CREB3L3; CREB3L1; CREB5; CCNE1; CCNE2; EGF; IGF1; INS; PDGFA; PDGFB; PDGFC; TGFA; PDGFD; RB1; PDPK1; SOS1; SOS2; GRB2; GSK3B; HSP90AA1; HSP90AB1; HSP90B1; MTOR; AKT3; AKT1; AKT2; PIK3CA; PIK3CB; PIK3CD; PIK3R1; PIK3R2; PIK3R3; GSTP1; MAPK1; MAPK3; MAP2K1; MAP2K2; ARAF; RAF1; BRAF; TMPRSS2; ERG; ETV5; PLAU; MMP3; MMP9; ZEB1; IL1R2; SPINT1; PLAT
Small cell lung cancer	CCNE1; CCNE2; CDK2; CCND1; ITGB1; ITGA6; ITGA2; ITGA2B; ITGA3; ITGAV; ZBTB17; CDKN1B; RXRA; RXRB; RXRG; RARB; AKT3; AKT1; AKT2; CKS1B; CKS2; CASP9; APAF1; MAX; MYC; PTEN; PIK3CA; PIK3CB; PIK3CD; PIK3R1; PIK3R2; PIK3R3; PTK2; FHIT; CYCS; LAMC3; COL4A1; COL4A2; COL4A3; COL4A4; COL4A5; COL4A6; LAMB4; FN1; LAMA1; LAMA2; LAMA3; LAMA4; LAMA5; LAMB1; LAMB2; LAMB3; LAMC1; LAMC2; BIRC2; BIRC3; XIAP; BIRC7; BCL2; NOS2; PTGS2; CHUK; IKBKB; IKBKG; NFKBIA; BCL2L1; TRAF1; TRAF2; TRAF3; TRAF5; TRAF6; TRAF4; E2F1; E2F2; E2F3; CDK4; CDK6; NFKB1; RELA; RB1; SKP2; CDKN2B; BAX; CASP3; TP53; CDKN1A; GADD45G; GADD45A; GADD45B; BAK1; DDB2; POLK
Asthma	HLA-DMA; HLA-DMB; HLA-DOA; HLA-DOB; HLA-DPA1; HLA-DPB1; HLA-DQA1; HLA-DQA2; HLA-DQB1; HLA-DRA; HLA-DRB1; HLA-DRB3; HLA-DRB4; HLA-DRB5; CD40LG; CD40; IL10; IL9; FCER1A; MS4A2; FCER1G; IL4; IL13;

	RNASE3; PRG2; LOC102723407; EPX; CCL11; IL5; IL3; TNF
Allograft rejection	HLA-A; HLA-B; HLA-C; HLA-DMA; HLA-DMB; HLA-DOA; HLA-DOB; HLA-DPA1; HLA-DPB1; HLA-DQA1; HLA-DQA2; HLA-DQB1; HLA-DRA; HLA-DRB1; HLA-DRB3; HLA-DRB4; HLA-DRB5; HLA-E; HLA-F; HLA-G; IL2; CD40LG; CD40; LOC102723407; FAS; FASLG; GZMB; PRF1; IL4; IL5; CD80; CD86; CD28; IL10; IFNG; TNF; IL12A; IL12B
Fluid shear stress and atherosclerosis	MAPK14; MAPK11; MAPK13; MAPK12; VCAM1; ICAM1; FOS; JUN; AKT3; AKT1; AKT2; MAPK8; MAPK9; MAPK10; PIK3CA; PIK3CB; PIK3CD; PIK3R1; PIK3R2; PIK3R3; CCL2; KEAP1; NFE2L2; MAPK7; MEF2A; MEF2C; KLF2; NQO1; MAP2K7; MAP2K4; MAP2K6; NOX1; CYBA; NCF1; NCF2; RAC1; RAC2; RAC3; BMP4; BMPR1A; BMPR1B; BMPR2; ACVR1; ACVR2A; ACVR2B; PRKCZ; MAP2K5; CDH5; PECAM1; KDR; HMOX1; GSTO2; GSTA5; GSTA1; GSTA2; GSTA3; GSTA4; GSTM1; GSTM2; GSTM3; GSTM4; GSTM5; GSTP1; GSTT1; GSTT2; MGST1; MGST2; MGST3; GSTT2B; GSTO1; TXN2; TXN; SQSTM1; NOS3; NPPC; ASS1; THBD; PLAT; CTNNB1; MMP2; MMP9; CTSL; SELE; TNF; IL1A; IL1B; IFNG; PDGFA; PDGFB; EDN1; VEGFA; ITGA2B; ITGAV; ITGB3; SRC; PTK2; ACTB; ACTG1; RHOA; TP53; SUMO4; SUMO3; SUMO2; SUMO1; PIAS4; BCL2; PRKAA1; PRKAA2; MAP3K5; TNFRSF1A; IL1R1; IL1R2; MAP3K7; CHUK; IKBKB; IKBKG; NFKB1; RELA; MIR10A; CAV1; CAV2; CAV3; CALML6; CALML5; CALM1; CALM2; CALM3; CALML3; CALML4; HSP90AA1; HSP90AB1; HSP90B1; GPC1; SDC1; SDC2; SDC4; TRPV4; DUSP1; ARHGEF2
Arrhythmogenic right ventricular cardiomyopathy	SGCD; CACNG3; CACNG2; CACNG5; CACNG4; CACNA2D3; CACNG8; CACNG7; CACNG6; CACNA1C; CACNA1D; CACNA1F; CACNA1S; CACNA2D1; CACNB1; CACNB2; CACNB3; CACNB4; CACNG1; CACNA2D2; CACNA2D4; RYR2; ATP2A1; ATP2A2; ATP2A3; SLC8A2; SLC8A1; SLC8A3; SGCG; SGCB; SGCA; DAG1; LAMA1; LAMA2; EMD; LMNA; DMD; DSC2; DSG2; JUP; PKP2; DSP; ITGA11; ITGA6; ITGA1; ITGA2; ITGA2B; ITGA3; ITGA4; ITGA5; ITGA7; ITGA9; ITGAV; ITGA10; ITGA8; ITGB1; ITGB3; ITGB4; ITGB5; ITGB6; ITGB7; ITGB8; ACTB; ACTG1; DES; CDH2; CTNNA1; CTNNA2; CTNNA3; ACTN2; ACTN3; LEF1; TCF7; TCF7L2; TCF7L1; CTNNB1; GJA1
Vibrio cholerae infection	ARF1; TCIRG1; ATP6V1G3; ATP6V0E2; ATP6V0A2; ATP6V0D2; ATP6V1C2; ATP6V0A4; ATP6V1D; ATP6V1H; ATP6V1A; ATP6V1B1; ATP6V1B2; ATP6V0C; ATP6V1C1; ATP6V1E1; ATP6V0B; ATP6V1G2; ATP6V0A1; ATP6AP1; ATP6V0E1; ATP6V1E2; ATP6V0D1; ATP6V1F; ATP6V1G1; ERO1A; CFTR; TJP2; TJP1; GNAS; KCNQ1; PRKCA; SLC12A2; PLCG1; PLCG2; PRKACA; PRKACB; PRKACG; SEC61B; SEC61G; SEC61A1; SEC61A2; ACTB; ACTG1; PDIA4; ADCY3; ADCY9; KDELR1; KDELR2; KDELR3
Bacterial invasion of epithelial cells	SEPTIN9; SEPTIN12; SEPTIN1; SEPTIN6; SEPTIN8; SEPTIN2; SEPTIN11; SEPTIN3; CTNNB1; CTNNA1; CTNNA2; CTNNA3; RAC1; CDH1; ARHGAP10; CDC42; SRC; CTTN; HCLS1; ITGA5; ITGB1; ARPC5; ARPC4; ARPC3; ARPC1B; ARPC2; ARPC1A; ARPC5L; ACTB; ACTG1; DOCK1; PXN; PTK2; CRK; CRKL; CD2AP; PIK3CA; PIK3CB; PIK3CD; PIK3R1; PIK3R2; PIK3R3; CBL; MET; DNM1; DNM2; DNM3; BCAR1; CLTA; CLTB; CLTC; CLTCL1; WASF2; WASF1; WASL; GAB1; SHC2; SHC4; SHC3; SHC1; FN1; CAV1; CAV2; CAV3; ILK; RHOG; ARHGEF26; MAD2L2; VCL;

	RHOA; ELMO2; ELMO3; ELMO1
Malaria	ICAM1; HGF; SDC1; SDC2; LRP1; CD81; MET; ACKR1; GYPA; GYPB; GYPC; VCAM1; CD36; PECAM1; HBA1; HBA2; HBB; TLR9; TLR2; TLR4; MYD88; IL10; IFNG; IL6; CCL2; TNF; IL12A; IL18; CR1; CR1L; ITGAL; ITGB2; CD40LG; CD40; COMP; THBS1; THBS2; THBS3; THBS4; SELE; TGFB1; TGFB2; TGFB3; IL1B; CSF3; CXCL8; KLRC4-KLRK1; KLRK1; KLRB1; SELP
Leishmaniasis	ITGA4; ITGB1; FCGR1A; FCGR2A; FCGR3A; FCGR3B; FCGR2C; C3; PRKCB; NCF1; CR1; CR1L; ITGAM; ITGB2; IFNG; JAK1; JAK2; STAT1; HLA-DMA; HLA-DMB; HLA-DOA; HLA-DOB; HLA-DPA1; HLA-DPB1; HLA-DQA1; HLA-DQA2; HLA-DQB1; HLA-DRA; HLA-DRB1; HLA-DRB3; HLA-DRB4; HLA-DRB5; TLR2; TLR4; MYD88; NFKBIA; NFKBIB; NFKB1; RELA; IL1A; IL4; IL12A; IL12B; NOS2; IL10; TGFB1; TGFB2; TGFB3; TNF; IL1B; PTPN6; EEF1A1; EEF1A2; MAPK1; MAPK3; ELK1; FOS; CYBB; MARCKSL1; MAPK14; MAPK11; MAPK13; MAPK12; IRAK1; IRAK4; TRAF6; TAB1; TAB2; MAP3K7; IFNGR1; IFNGR2; JUN; CYBA; NCF2; NCF4; PTGS2; LOC102723407
African trypanosomiasis	TLR9; MYD88; IL12A; IL12B; IL18; IFNG; TNF; APOL1; SELE; VCAM1; LAMA4; IL1B; LOC102723407; IL10; IL6; ICAM1; THOP1; HPR; APOA1; HBA1; HBA2; HBB; IDO2; IDO1; FAS; FASLG; F2RL1; GNAQ; PLCB1; PLCB2; PLCB3; PLCB4; PRKCA; PRKCB; PRKCG; KNG1; NPPA
IL-17 signaling pathway	IKBKB; MAP3K7; CHUK; TRAF6; IL6; TAB3; TAB2; NFKBIA; IKBKG; NFKB1; RELA; FOS; FOSB; JUN; JUND; FOSL1; IL25; IL17RA; IL17RB; IL17A; IL17F; IL17C; IL17RE; IL17B; TRAF3IP2; TRAF3; IL17RC; HSP90AA1; HSP90AB1; HSP90B1; TRAF5; TRAF2; ELAVL1; SRSF1; IKBKE; MAPK14; MAPK15; MAPK1; MAPK3; MAPK4; MAPK6; MAPK7; MAPK8; MAPK11; MAPK9; MAPK10; MAPK13; MAPK12; CEBPB; TRADD; FADD; CASP3; CASP8; GSK3B; IL17D; CXCL1; CXCL2; CXCL3; CCL2; CCL7; CCL20; DEFB4B; DEFB4A; MUC5AC; MMP9; MMP13; IL4; IL5; IL13; CCL17; TBK1; TNFAIP3; ANAPC5; USP25; CXCL8; CSF2; CCL11; MMP1; MMP3; LCN2; PTGS2; TNF; CXCL6; CXCL5; CXCL10; CSF3; MUC5B; S100A7A; S100A7; S100A8; S100A9; TRAF4; IL1B; IFNG
Adipocytokine signaling pathway	NFKB1; RELA; NFKBIA; NFKBIB; NFKBIE; PTPN11; SOCS3; CPT1C; CPT1A; CPT1B; G6PC; G6PC2; G6PC3; PCK1; PCK2; ACSL1; ACSL3; ACSL4; ACSBG1; ACSL6; ACSL5; ACSBG2; LEP; POMC; PRKAG2; PRKAG3; PRKAA1; PRKAA2; PRKAB1; PRKAB2; PRKAG1; ADIPOQ; ACACB; PPARA; ADIPOR1; ADIPOR2; PRKCQ; LEPR; CAMKK2; STK11; NPY; RXRA; RXRB; RXRG; SLC2A1; SLC2A4; CHUK; IKBKB; IKBKG; TNFRSF1B; AGRP; STAT3; MAPK8; MAPK9; MAPK10; AKT3; AKT1; AKT2; MTOR; TRAF2; IRS1; IRS4; IRS2; TNFRSF1A; CD36; PPARGC1A; TRADD; JAK2; TNF
Various types of N-glycan biosynthesis	MAN1A2; MAN1A1; MAN1C1; ALG11; ALG9; ALG3; ALG12; ALG2; ALG1; ALG14; ALG13; DAD1; DDOST; STT3B; STT3A; RPN1; RPN2; TUSC3; MGAT1; MGAT2; B4GALNT3; B4GALNT4; FUT8; CHST8; CHST9; B4GALT1; B4GALT3; B4GALT2; ST3GAL3; MGAT4B; MGAT4A; MGAT4D; MGAT4C; MAN2A2; MAN2A1; HEXD; MAN1B1; HEXA; HEXB
Aminoacyl-tRNA biosynthesis	IARS1; IARS2; NARS1; NARS2; HARS2; HARS1; FARSB; FARS2; FARSA; RARS2; RARS1; QARS1; CARS2; CARS1; GARS1; DARS1; DARS2; SARS2; SARS1;

	MARS1; MARS2; VARS2; VARS1; AARS1; AARS2; KARS1; LARS2; LARS1; TARS3; TARS1; TARS2; WARS2; WARS1; MTFMT; YARS2; YARS1; EPRS1; PARS2; EARS2; GATC; GATB; QRSL1; PSTK; SEPSECS; TRNE; TRNQ; TRNA; TRND; TRNS1; TRNS2; TRNT; TRNN; TRNG; TRNC; TRNM; TRNV; TRNL1; TRNL2; TRNI; TRNK; TRNR; TRNP; TRNH; TRNF; TRNY; TRNW
EGFR tyrosine kinase inhibitor resistance	ERBB3; ERBB2; EGFR; GSK3B; EIF4EBP1; RPS6KB1; RPS6KB2; MTOR; BAD; GAB1; TGFA; SOS1; SOS2; GRB2; SHC2; SHC4; SHC3; SHC1; PRKCA; PRKCB; PRKCG; PLCG1; PLCG2; PIK3CA; PIK3CB; PIK3CD; PIK3R1; PIK3R2; PIK3R3; AKT3; AKT1; AKT2; EGF; MAPK1; MAPK3; MAP2K1; MAP2K2; ARAF; RAF1; BRAF; HRAS; KRAS; NRAS; PTEN; NF1; MET; IGF1R; PDGFRA; PDGFRB; KDR; HGF; FGFR3; FGFR2; STAT3; JAK1; JAK2; BCL2; BAX; BCL2L1; BCL2L11; IGF1; VEGFA; PDGFA; PDGFB; PDGFC; PDGFD; FGF2; SRC; GAS6; AXL; IL6; IL6R; RPS6; EIF4E; EIF4E1B; EIF4E2; FOXO3; NRG1; NRG2
Endocrine resistance	ESR1; ESR2; GPER1; SRC; MMP2; MMP9; HBEGF; EGFR; CARM1; MED1; NCOA3; PRKACA; PRKACB; PRKACG; HRAS; KRAS; NRAS; ARAF; RAF1; BRAF; MAP2K1; MAP2K2; MAPK1; MAPK3; SHC2; SHC4; SHC3; SHC1; GRB2; SOS1; SOS2; GNAS; ADCY1; ADCY2; ADCY3; ADCY5; ADCY6; ADCY7; ADCY8; ADCY9; ADCY4; PIK3CA; PIK3CB; PIK3CD; PIK3R1; PIK3R2; PIK3R3; AKT3; AKT1; AKT2; MAPK14; MAPK11; MAPK13; MAPK12; MAPK8; MAPK9; MAPK10; JAG1; JAG2; NOTCH1; NOTCH2; NOTCH3; NOTCH4; MDM2; TP53; CDKN2A; CDKN2C; CDKN1A; CDKN1B; CCND1; CDK4; RB1; E2F1; E2F2; E2F3; MTOR; RPS6KB1; RPS6KB2; IGF1; IGF1R; ERBB2; JUN; FOS; BCL2; BIK; BAD; BAX; SP1; NCOR1; PTK2; LOC107987478; LOC107987479; CYP2D7; CYP2D6; ABCB11
Antifolate resistance	FOLR1; FOLR2; FOLR3; IZUMO1R; SLC19A1; ABCC1; ABCC2; ABCC3; ABCC4; ABCC5; GART; FPGS; SLC46A1; GGH; TYMS; DHFR; DHFR2; ATIC; ABCG2; CHUK; IKBKB; IKBKG; NFKB1; RELA; IL6; IL1B; TNF; SHMT1; SHMT2; ALOX12; MTHFR
Platinum drug resistance	ATM; TP53; BCL2; CASP9; CASP3; BAD; BIRC2; BIRC3; XIAP; BIRC5; APAF1; CYCS; BID; CASP8; FADD; FAS; FASLG; BAX; BAK1; PMAIP1; BBC3; BCL2L1; SLC31A1; ATP7A; ATP7B; ABCC2; GSTO2; GSTA5; GSTA1; GSTA2; GSTA3; GSTA4; GSTM1; GSTM2; GSTM3; GSTM4; GSTM5; GSTP1; GSTT1; GSTT2; MGST1; MGST2; MGST3; GSTT2B; GSTO1; MLH1; XPA; ERCC1; TOP2A; TOP2B; BRCA1; MSH2; MSH6; MSH3; ERBB2; PIK3CA; PIK3CB; PIK3CD; PIK3R1; PIK3R2; PIK3R3; PDPK1; AKT3; AKT1; AKT2; MAPK1; MAPK3; CDKN2A; MDM2; CDKN1A; POLH; REV3L; MAP3K5
ABC transporters	ABCB11; ABCG8; ABCG5; ABCG2; ABCG4; ABCG1; ABCD4; ABCD3; ABCD2; ABCD1; ABCC10; CFTR; ABCC4; ABCC12; ABCC11; ABCC9; ABCC8; ABCC6; ABCC5; ABCC3; ABCC2; ABCC1; ABCB7; ABCB6; ABCB5; ABCB4; ABCB1; ABCB10; ABCB9; ABCB8; TAP2; TAP1; ABCA10; ABCA9; ABCA8; ABCA6; ABCA5; ABCA13; ABCA12; ABCA7; ABCA4; ABCA3; ABCA2; ABCA1; DEFB1
Ribosome biogenesis in eukaryotes	RAN; XPO1; NMD3; SNORD3E; SNORD3B-1; SNORD3A; SNORD3B-2; SNORD3C; RNR1; RNA5-8SN5; RNA5-8SN1; RNA5-8SN4; RNA5-8SN2; RNA5-8SN3; RNR2; RNA5S1; RNA5S2; RNA5S3; RNA5S4; RNA5S5; RNA5S6; RNA5S7; RNA5S8; RNA5S9; RNA5S10; RNA5S11; RNA5S12; RNA5S13; RNA5S14; RNA5S15;

	RNA5S16; RNA5S17; TCOF1; SNU13; NOP58; NOP56; FBL; FBLL1; NOP10; NHP2; GAR1; DKC1; FCF1; DROSHA; NAT10; UTP14A; UTP14C; BMS1; RCL1; POP7; RPP30; RPP38; POP4; RPP40; POP1; RPP25L; POP5; RPP25; XRN2; XRN1; GTPBP4; GNL3; GNL2; GNL3L; MDN1; NVL; LOC101929601; LOC101929627; REXO2; REXO1; REXO5; NXF1; NXF5; NXF3; NXF2; NXF2B; NXT1; NXT2; MPHOSPH10; LOC643802; IMP4; IMP3; WDR75; UTP15; UTP4; HEATR1; WDR43; UTP18; TBL3; UTP6; PWP2; WDR36; WDR3; CSNK2A1; CSNK2A2; CSNK2A3; CSNK2B; NOL6; LSG1; EFL1; RRP7A; RIOK1; SPATA5; RIOK2; AK6; RMRP; EIF6; EMG1; SBDS; RBM28; NOB1
Ribosome	FAU; RPL13; RPL28; RPS14; RPS23; RPS18; RPS29; RPS13; RPS11; RPS15; RPS6; RPS8; RPS17; RPS19; RPS24; RPS27L; RPS27; RPS27A; RPS7; RPS10-NUDT3; RPS10; RPS12; RPS21; RPS25; RPS26; RPS28; RPS4Y2; RPS4X; RPS4Y1; RPS3A; RNR2; RNA5S1; RNA5S2; RNA5S3; RNA5S4; RNA5S5; RNA5S6; RNA5S7; RNA5S8; RNA5S9; RNA5S10; RNA5S11; RNA5S12; RNA5S13; RNA5S14; RNA5S15; RNA5S16; RNA5S17; MRPL10; MRPL11; RPL3; RPL3L; RPL11; RPL9; RPLP0; RPL12; RPL13A; MRPL13; RNA5-8SN5; RNA5-8SN1; RNA5-8SN4; RNA5-8SN2; RNA5-8SN3; MRPL2; MRPL3; RPL10A; RPL8; MRPL14; RPL23; RPL27A; MRPL15; RPL5; MRPL18; RPL17-C18orf32; RPL17; MRPL22; RPL23A; MRPL23; RPL26L1; RPL26; MRPL24; RPL35; MRPL30; RPL7; RPL15; RPL18; RPL19; RPL21; RSL24D1; RPL24; RPL30; RPL39; UBA52; RPL41; RPL36A-HNRNPH2; RPL36AL; RPL36A; RPL31; RPL32; RPL34; RPL35A; RPL37; RPL37A; RPL6; RPL18A; RPL22L1; RPL22; RPL27; RPL29; RPL36; RPL38; RPL4; RPL7A; RPL10L; RPL10; RPL14; MRPL4; MRPL9; MRPL12; RPLP1; RPLP2; MRPL16; MRPL17; MRPL19; MRPL20; MRPL21; MRPL27; MRPL28; MRPL32; MRPL33; MRPL34; MRPL35; MRPL36; RNR1; MRPS5; MRPS7; MRPS9; MRPS10; RPS2; RPS5; RPS15A; RPS16; RPS20; RPSA; RPS3; MRPS11; MRPS12; MRPS14; MRPS15; MRPS17; MRPS6; MRPS16; MRPS18C; MRPS18A; MRPS21; RPS9; MRPS2; MRPL1
RNA transport	XPOT; RAN; XPO1; NCBP2; NCBP2L; NCBP1; PHAX; CLNS1A; GEMIN2; SMN1; SMN2; DDX20; GEMIN5; GEMIN4; GEMIN7; GEMIN8; STRAP; TGS1; GEMIN6; KPNB1; SNUPN; RBM8A; MAGOH; MAGOHB; EIF4A3; CASC3; ACIN1; RNPS1; SAP18; SRRM1; NXF1; NXF5; NXF3; NXF2; NXF2B; PNN; ALYREF; NXT1; NXT2; NUP58; NUP205; NUP188; NUP62; TPR; NUP153; NUP93; NUP54; NUP50; NUP155; SENP2; NUP98; NUP37; SEC13; NUP160; NUP107; NUP85; SEH1L; RAE1; NUP43; NUP133; NUP210; NUP210L; POM121C; POM121L2; POM121; NDC1; NUP214; NUP88; RANBP2; UBE2I; RANGAP1; SUMO4; SUMO3; SUMO2; SUMO1; NUP42; AAAS; PRMT5; XPO5; EEF1A1; EEF1A2; NMD3; UPF1; DDX39B; PYM1; EIF1; EIF1B; EIF1AX; EIF1AY; EIF3E; EIF3CL; EIF3A; EIF3B; EIF3C; EIF3D; EIF3F; EIF3G; EIF3H; EIF3I; EIF3J; TRNT1; POP7; RPP30; RPP38; POP4; RPP40; POP1; RPP14; RPP25L; POP5; RPP25; RPP21; ELAC1; ELAC2; THOC1; THOC2; THOC3; THOC5; THOC6; THOC7; PABPC4L; PABPC5; PABPC1; PABPC1L2A; PABPC3; PABPC1L2B; PABPC1L; PABPC4; EIF2S1; EIF2S3; EIF2S2; PAIP1; EIF4A1; EIF4A2; EIF4E; EIF4E1B; EIF4E2; EIF4G1; EIF4G2; EIF4G3; EIF5; UPF2; UPF3B; UPF3A; EIF4B; EIF4EBP1; EIF4EBP2; EIF4EBP3; TACC3; EIF5B; EIF2B1; EIF2B4; EIF2B3; EIF2B2; EIF2B5; NUP35; RNVU1-4; RNVU1-20; RNVU1-1; RNVU1-18; RNVU1-7;

	RNU1-3; RNU1-2; RNU1-1; RNU1-4; RNU2-1; RNU4-2; RNU4-1; RNU5A-1; RNU6-9; RNU6-1; SNORD3E; SNORD3B-1; SNORD3A; SNORD3B-2; SNORD3C; CYFIP1; CYFIP2; FMR1; FXR1; FXR2; DDX19B; DDX19A; GLE1; TARDBP; FUS
RNA degradation	ENO1; ENO2; ENO3; ENO4; PNPT1; EXOSC4; EXOSC7; EXOSC1; EXOSC2; TTC37; WDR61; DIS3; SKIV2L; EXOSC9; EXOSC6; EXOSC8; EXOSC5; EXOSC3; C1D; EXOSC10; MPHOSPH6; TENT4A; TENT4B; ZCCHC7; MTREX; CNOT1; CNOT2; CNOT3; CNOT4; CNOT6L; CNOT6; CNOT7; CNOT8; CNOT9; CNOT10; DCPS; DCP1B; DCP1A; DCP2; LSM1; LSM2; LSM3; LSM4; LSM5; LSM6; LSM7; LSM8; XRN1; XRN2; DDX6; EDC3; EDC4; PATL1; PNLDC1; PARN; HSPA9; HSPD1; DHX36; TOB1; TOB2; BTG3; BTG4; BTG1; BTG2; PABPC4L; PABPC5; PABPC1; PABPC1L2A; PABPC3; PABPC1L2B; PABPC1L; PABPC4; PAN2; PAN3; NUDT16; DIS3L; PFKL; PFKM; PFKP
Basal transcription factors	TBPL2; TBP; TBPL1; TAF1L; TAF1; TAF2; TAF3; TAF4; TAF4B; TAF5L; TAF5; TAF6L; TAF6; TAF7L; TAF7; TAF8; TAF9B; TAF9; TAF10; TAF11; GTF2B; GTF2A1L; GTF2A1; GTF2A2; GTF2I; GTF2IRD1; GTF2F1; GTF2F2; ERCC2; GTF2E1; GTF2E2; GTF2H1; GTF2H2; GTF2H2C; GTF2H2C_2; GTF2H3; GTF2H4; ERCC3; TAF12; TAF13; TAF15; GTF2H5; CDK7; MNAT1; CCNH
DNA replication	LIG1; FEN1; DNA2; RNASEH2A; RNASEH2B; RFC2; RFC4; RNASEH2C; RFC3; RFC5; PCNA; RPA3; MCM5; POLD4; POLD3; POLE4; POLE3; SSBP1; RNASEH1; MCM2; MCM3; MCM4; POLA1; POLA2; PRIM1; PRIM2; POLD1; POLD2; POLE; POLE2; MCM6; MCM7; RPA1; RPA4; RPA2; RFC1
Protein export	OXA1L; SRP14; SRP72; SRP68; SRP54; SRP19; SRPRA; SRP9; SEC62; SEC63; SPCS1; SPCS2; SEC61A1; SEC61A2; SPCS3; SEC11A; SEC11C; IMMP1L; IMMP2L; SRPRB; SEC61B; SEC61G; HSPA5
PPAR signaling pathway	LOC100509620; LOC112267859; AQP7; GK; GK2; PCK1; PCK2; PLIN2; PLIN5; PLIN1; PLIN4; CD36; SLC27A5; SLC27A4; SLC27A2; SLC27A6; SLC27A1; FABP4; FABP1; FABP2; FABP3; FABP5; FABP6; FABP7; PPARA; RXRA; RXRB; RXRG; PPARD; PPARG; SCP2; ACAA1; ACADM; ACADL; CYP4A11; CYP4A22; EHHADH; CPT2; CPT1C; CPT1A; CPT1B; ACSL1; ACSL3; ACSL4; ACSBG1; ACSL6; ACSL5; ACSBG2; OLR1; LPL; CYP7A1; HMGCS1; HMGCS2; SORBS1; MMP1; ANGPTL4; ACOX1; ACOX2; ACOX3; ILK; PDPK1; APOA2; PLTP; ADIPOQ; SCD; SCD5; FADS2; APOC3; CYP8B1; NR1H3; CYP27A1; APOA1; UBC; DBI; APOA5; ME3; ME1; UCP1
Mismatch repair	MSH2; MLH3; MLH1; LIG1; PCNA; POLD3; POLD1; POLD2; POLD4; RPA4; RPA1; RPA2; RPA3; MSH6; EXO1; MSH3; PMS2; RFC1; RFC2; RFC3; RFC4; RFC5; SSBP1
Homologous recombination	TOP3A; TOP3B; BLM; SEM1; RAD52; MRE11; RAD50; XRCC3; RAD51C; XRCC2; RAD51D; RAD51B; MUS81; NBN; RPA4; RPA1; RPA2; RPA3; RAD51; EME1; BRCA2; RAD54B; RAD54L; POLD3; POLD1; POLD2; POLD4; SSBP1; SYCP3; BRCA1; RBBP8; PALB2; UIMC1; ABRAXAS1; BRIP1; BARD1; TOPBP1; ATM; BABAM2; BABAM1; BRCC3
Non-homologous end-joining	LIG4; POLL; DNTT; POLM; DCLRE1C; MRE11; RAD50; PRKDC; XRCC5; XRCC4; XRCC6; FEN1; NHEJ1
Fanconi anemia pathway	FANCM; FANCA; FANCB; FANCC; FANCD2; FANCI; FANCE; FANCF; FANCG; FANCL; PALB2; FAAP100; BLM; RMI1; RMI2; TOP3A; TOP3B; ATRIP; ATR; RPA4;

	RPA1; RPA2; RPA3; TELO2; BRCA2; MLH1; BRIP1; BRCA1; RAD51C; FAN1; USP1; WDR48; UBE2T; REV1; CENPS-CORT; CENPX; CENPS; POLH; POLK; REV3L; POLI; PMS2; MUS81; EME1; EME2; ERCC4; ERCC1; SLX1A; SLX1B; SLX4; RAD51; FAAP24; HES1; POLN
MAPK signaling pathway	RASGRF1; RASGRF2; DUSP10; DUSP1; DUSP2; DUSP4; DUSP5; DUSP6; DUSP7; DUSP8; DUSP9; DUSP16; DUSP3; PTPN7; PTPRR; PTPN5; PPM1B; FASLG; MAP4K4; PLA2G4B; PLA2G4E; PLA2G4F; PLA2G4D; PLA2G4A; PLA2G4C; JMJD7-PLA2G4B; STMN1; MAPT; NFKB1; NFKB2; RELA; RELB; CHUK; IKBKB; IKBKG; MAP3K14; MAPK1; MAPK3; LAMTOR3; MAP2K2; MAP2K1; RAF1; BRAF; RASA1; NF1; PRKCA; PRKCB; PRKCG; RAP1A; RAP1B; PRKACA; PRKACB; PRKACG; RAPGEF2; RASA2; RRAS2; MRAS; HRAS; KRAS; NRAS; RRAS; RASGRP1; RASGRP2; RASGRP4; RASGRP3; SOS1; SOS2; GNA12; GNG12; GRB2; CSF1R; EGFR; EPHA2; ERBB2; ERBB3; ERBB4; FGFR1; FGFR3; FGFR2; FGFR4; FLT1; FLT3; FLT4; IGF1R; INSR; KDR; KIT; MET; NGFR; NTRK1; NTRK2; PDGFRA; PDGFRB; TEK; CACNG3; CACNG2; CACNG5; CACNG4; CACNA2D3; CACNG8; CACNG7; CACNG6; CACNA1A; CACNA1B; CACNA1C; CACNA1D; CACNA1E; CACNA1F; CACNA1S; CACNA2D1; CACNB1; CACNB2; CACNB3; CACNB4; CACNG1; CACNA1I; CACNA1H; CACNA1G; CACNA2D2; CACNA2D4; NR4A1; MAPK7; MAP2K5; NLK; CDC25B; RPS6KA4; RPS6KA5; MAPKAPK3; MAPKAPK2; ATF4; MEF2C; MAX; DDIT3; ELK4; TP53; ELK1; ATF2; MAPK14; MAPK11; MAPK13; MAPK12; PPP5C; MAP2K6; MAP2K3; PPM1A; AKT3; AKT1; AKT2; TAOK3; TAOK1; TAOK2; MAP3K4; HSPB1; MAP3K7; MAP3K5; MAP3K6; MAP3K20; MAP3K12; MAP3K13; MAP4K2; STK3; STK4; ECSIT; TAB2; TAB1; GADD45G; GADD45A; GADD45B; TRAF6; DAXX; TRAF2; CASP3; CD14; TGFBR1; TGFBR2; FAS; IL1R1; IL1RAP; TNFRSF1A; TGFB1; TGFB2; TGFB3; IL1A; IL1B; TNF; MAPKAPK5; RAC1; RAC2; RAC3; CDC42; PAK1; PAK2; MAP3K2; MAP3K3; MAPK8IP2; MAPK8IP1; MAP3K11; MAP2K7; MAPK8; MAPK9; MAPK10; MAP2K4; CRK; CRKL; ARRB1; ARRB2; MECOM; HSPA1A; HSPA1B; HSPA1L; HSPA2; HSPA6; HSPA8; JUND; FOS; JUN; NFATC3; NFATC1; PPP3CA; PPP3CB; PPP3CC; PPP3R1; PPP3R2; MAPK8IP3; FLNA; FLNB; FLNC; MAP3K1; MAP3K8; MAP4K1; MAP4K3; SRF; MYC; RPS6KA6; RPS6KA1; RPS6KA2; RPS6KA3; MKNK2; MKNK1; CSF1; EFNA1; EFNA2; EFNA3; EFNA4; EFNA5; EGF; EREG; FGF1; FGF2; FGF3; FGF4; FGF5; FGF6; FGF7; FGF8; FGF9; FGF10; VEGFD; FLT3LG; FGF20; FGF21; FGF22; ANGPT1; ANGPT2; HGF; IGF1; IGF2; INS; AREG; KITLG; NGF; NTF3; NTF4; ANGPT4; PDGFA; PDGFB; PGF; PDGFC; BDNF; TGFA; VEGFA; VEGFB; VEGFC; PDGFD; FGF23; FGF18; FGF17; FGF16; FGF19; TRADD; MYD88; IRAK1; IRAK4; ARAF
ErbB signaling pathway	GRB2; ELK1; ERBB4; ERBB3; STAT5A; STAT5B; ERBB2; EGFR; GSK3B; EIF4EBP1; RPS6KB1; RPS6KB2; CDKN1A; CDKN1B; MTOR; PAK4; BUB1B-PAK6; PAK1; PAK2; PAK3; PAK6; PAK5; NCK1; NCK2; BAD; GAB1; TGFA; MAP2K7; MAP2K4; CBL; CBLB; NRG3; NRG2; MAPK8; MAPK9; MAPK10; CRK; CRKL; PTK2; SOS1; SOS2; SHC2; SHC4; SHC3; SHC1; PRKCA; PRKCB; PRKCG; CAMK2A; CAMK2B; CAMK2D; CAMK2G; PLCG1; PLCG2; HBEGF; NRG1; EREG; BTC; JUN; ABL1; ABL2; PIK3CA; PIK3CB; PIK3CD; PIK3R1; PIK3R2; PIK3R3;

	AKT3; AKT1; AKT2; SRC; NRG4; MYC; AREG; EGF; MAPK1; MAPK3; MAP2K1; MAP2K2; ARAF; RAF1; BRAF
Ras signaling pathway	MAPK1; MAPK3; MAP2K1; MAP2K2; RAF1; RRAS2; MRAS; HRAS; KRAS; NRAS; RRAS; GRB2; SOS1; SOS2; AKT3; AKT1; AKT2; PIK3CA; PIK3CB; PIK3CD; PIK3R1; PIK3R2; PIK3R3; SHC2; SHC4; SHC3; SHC1; CSF1; EFNA1; EFNA2; EFNA3; EFNA4; EFNA5; EGF; FGF1; FGF2; FGF3; FGF4; FGF5; FGF6; FGF7; FGF8; FGF9; FGF10; VEGFD; FLT3LG; FGF20; FGF21; FGF22; ANGPT1; ANGPT2; HGF; IGF1; IGF2; INS; KITLG; NGF; NTF3; NTF4; ANGPT4; PDGFA; PDGFB; PGF; PDGFC; BDNF; TGFA; VEGFA; VEGFB; VEGFC; PDGFD; FGF23; FGF18; FGF17; FGF16; FGF19; CSF1R; EGFR; EPHA2; FGFR1; FGFR3; FGFR2; FGFR4; FLT1; FLT3; FLT4; IGF1R; INSR; KDR; KIT; MET; NGFR; NTRK1; NTRK2; PDGFRA; PDGFRB; TEK; GAB1; GAB2; PTPN11; TIAM1; RAC1; RAC2; RAC3; RALGDS; RGL1; RGL2; RALA; RALB; PLD1; PLD2; PLCE1; PRKCA; PRKCB; PRKCG; ABL1; ABL2; RIN1; BAD; BCL2L1; CHUK; IKBKB; IKBKG; NFKB1; RELA; FOXO4; FASLG; MAPK8; MAPK9; MAPK10; RALBP1; CDC42; PLA2G4B; PLAAT3; PLA2G4E; PLA2G4F; PLA2G2D; PLA2G4D; PLA2G2E; PLA2G2C; PLA2G3; PLA1A; PLA2G1B; PLA2G2A; PLA2G4A; PLA2G5; PLA2G2F; PLA2G12A; PLA2G6; PLA2G10; PLA2G12B; PLA2G4C; JMJD7-PLA2G4B; ELK1; ETS1; ETS2; RAB5A; RAB5B; RAB5C; RASGRP1; RASGRP2; RASGRP4; RASGRP3; RASGRF1; RASGRF2; RASA4B; RASA4; RASA3; RASA1; RASA2; RASAL3; RASAL1; SYNGAP1; RASAL2; NF1; RHOA; AFDN; RASSF5; RASSF1; STK4; RAPGEF5; RAP1A; RAP1B; ARF6; GRIN1; GRIN2A; GRIN2B; CALML6; CALML5; CALM1; CALM2; CALM3; CALML3; CALML4; GNB5; GNB1; GNB2; GNB3; GNG3; GNG4; GNG5; GNG7; GNG10; GNG11; GNGT1; GNGT2; GNG13; GNG2; GNG12; GNB4; GNG8; HTR7; PLCG1; PLCG2; ZAP70; LAT; PAK4; BUB1B-PAK6; PAK1; PAK2; PAK3; PAK6; PAK5; EXOC2; TBK1; REL; BRAP; KSR2; KSR1; PRKACA; PRKACB; PRKACG; SHOC2
Rap1 signaling pathway	MAPK1; MAPK3; MAP2K1; MAP2K2; RAF1; RAP1A; RAP1B; APBB1IP; CSF1; EFNA1; EFNA2; EFNA3; EFNA4; EFNA5; EGF; FGF1; FGF2; FGF3; FGF4; FGF5; FGF6; FGF7; FGF8; FGF9; FGF10; VEGFD; FGF20; FGF21; FGF22; ANGPT1; ANGPT2; HGF; IGF1; INS; KITLG; NGF; ANGPT4; PDGFA; PDGFB; PGF; PDGFC; VEGFA; VEGFB; VEGFC; PDGFD; FGF23; FGF18; FGF17; FGF16; FGF19; CSF1R; EGFR; EPHA2; FGFR1; FGFR3; FGFR2; FGFR4; FLT1; FLT4; IGF1R; INSR; KDR; KIT; MET; NGFR; PDGFRA; PDGFRB; TEK; RALGDS; RAC1; RAC2; RAC3; RASSF5; MAP2K3; MAP2K6; MAPK14; MAPK11; MAPK13; MAPK12; EVL; ENAH; VASP; PFN3; PFN4; PFN1; PFN2; ACTB; ACTG1; CDH1; DOCK4; RAPGEF3; RAPGEF4; SIPA1L1; SIPA1; RAP1GAP; RALA; RALB; AFDN; BRAF; ITGAL; ITGAM; ITGB1; ITGB2; ADORA2A; ADORA2B; LPAR1; F2R; LPAR3; FPR1; LPAR4; P2RY1; LPAR5; F2RL3; LPAR2; GNAS; GNAQ; PLCB1; PLCB2; PLCB3; PLCB4; CRK; CRKL; RAPGEF1; CTNNB1; MAGI3; MAGI1; MAGI2; RAPGEF6; RAPGEF2; VAV3; VAV1; VAV2; TIAM1; PARD3; ARAP3; RHOA; PRKD3; PRKD2; PRKD1; KRIT1; SRC; FARP2; CDC42; MRAS; RAPGEF5; RASGRP3; RASGRP2; ADCY1; ADCY2; ADCY3; ADCY5; ADCY6; ADCY7; ADCY8; ADCY9; ADCY4; CNR1; DRD2; GNAI1; GNAI2; GNAI3; GNAO1; PIK3CA; PIK3CB; PIK3CD; PIK3R1;

	PIK3R2; PIK3R3; AKT3; AKT1; AKT2; SIPA1L2; SIPA1L3; HRAS; KRAS; NRAS; RRAS; CTNND1; GRIN1; GRIN2A; GRIN2B; CALML6; CALML5; CALM1; CALM2; CALM3; CALML3; CALML4; TLN1; TLN2; ITGA2B; ITGB3; LCP2; FYB1; SKAP1; PRKCA; PRKCB; PRKCG; LAT; PLCG1; ID1; THBS1; PLCE1; RGS14; BCAR1; PRKCI; PRKCZ
cGMP-PKG signaling pathway	PPP1R12A; PPP1CA; PPP1CB; PPP1CC; MYL9; NPR1; PRKG1; PRKG2; GUCY1A2; GUCY1A1; GUCY1B1; ITPR1; ITPR2; ITPR3; KCNMB2; KCNU1; KCNMB3; KCNMB4; KCNMA1; KCNMB1; NPR2; IRAG1; PDE5A; CNGB1; CNGA1; RGS2; ADRA1D; ADRA1B; ADRA1A; ADRA2A; ADRA2B; ADRA2C; AGTR1; EDNRA; EDNRB; PLCB1; PLCB2; PLCB3; PLCB4; CACNA1C; CACNA1D; CACNA1F; CACNA1S; RHOA; ROCK1; ROCK2; GNA11; GNAQ; TRPC6; PPP3CA; PPP3CB; PPP3CC; PPP3R1; PPP3R2; NFATC1; NFATC2; NFATC3; NFATC4; MEF2B; MEF2A; BORCS8-MEF2B; MEF2C; MEF2D; GATA4; SRF; ATP2A1; ATP2A2; ATP2A3; PLN; KCNJ8; RAF1; MAP2K1; MAP2K2; MAPK1; MAPK3; BAD; CREB3; CREB1; ATF2; ATF6B; CREB3L4; ATF4; CREB3L2; CREB3L3; CREB3L1; CREB5; VASP; SLC8A2; SLC8A1; SLC8A3; ATP1B4; ATP1A1; ATP1A2; ATP1A3; ATP1A4; ATP1B1; ATP1B2; ATP1B3; FXYD2; ATP2B1; ATP2B2; ATP2B3; ATP2B4; GTF2I; GTF2IRD1; OPRD1; PIK3R6; PIK3R5; PIK3CG; AKT3; AKT1; AKT2; NOS3; MYH6; MYH7; NPPB; ADCY1; ADCY2; ADCY3; ADCY5; ADCY6; ADCY7; ADCY8; ADCY9; ADCY4; PDE2A; PDE3A; PDE3B; PRKCE; PPIF; SLC25A4; SLC25A5; SLC25A6; VDAC1; VDAC2; VDAC3; SLC25A31; MYLK4; MYLK; MYLK2; MYLK3; CALML6; CALML5; CALM1; CALM2; CALM3; CALML3; CALML4; IRS1; IRS4; IRS2; INSR; ADRB1; ADRB2; ADRB3; GNAI1; GNAI2; GNAI3; BDKRB2; ADORA1; ADORA3; INS; GNA13; GNA12; KNG1; NPPA; NPPC
cAMP signaling pathway	CACNA1C; CACNA1D; CACNA1F; CACNA1S; RYR2; ATP2A1; ATP2A2; ATP2A3; TNNI3; ATP1B4; ATP1A1; ATP1A2; ATP1A3; ATP1A4; ATP1B1; ATP1B2; ATP1B3; FXYD2; SLC9A1; CHRM1; CHRM2; ADORA1; ADRB2; DRD2; EDNRA; GABBR1; GHSR; HCAR1; FFAR2; HTR1A; HTR1B; HTR1D; HTR1E; HTR1F; HCAR2; NPY1R; OXTR; SUCNR1; PTGER3; SSTR1; SSTR2; SSTR5; HCAR3; GABBR2; GNAI1; GNAI2; GNAI3; GNAS; ADCY1; ADCY2; ADCY3; ADCY5; ADCY6; ADCY7; ADCY8; ADCY9; ADCY4; PRKACA; PRKACB; PRKACG; PIK3CA; PIK3CB; PIK3CD; PIK3R1; PIK3R2; PIK3R3; AKT3; AKT1; AKT2; MAPK1; MAPK3; PLN; CFTR; CALML6; CALML5; CALM1; CALM2; CALM3; CALML3; CALML4; RAPGEF3; RAPGEF4; ATP2B1; ATP2B2; ATP2B3; ATP2B4; RAP1A; RAP1B; RAF1; MAP2K1; MAP2K2; ARAP3; BRAF; PLCE1; MAPK8; MAPK9; MAPK10; CREB3; CREB1; CREB3L4; CREB3L2; CREB3L3; CREB3L1; CREB5; CNGB1; CNGA1; CNGA2; CNGA3; CNGA4; CNGB3; RHOA; ROCK1; ROCK2; PPP1R12A; CAMK4; CAMK2A; CAMK2B; CAMK2D; CAMK2G; ADCYAP1R1; ADORA2A; GPR119; ADRB1; DRD1; DRD5; FSHR; GIPR; GLP1R; HTR4; HTR6; LHCGR; MC2R; NPR1; PTGER2; TSHR; VIPR2; AFDN; PDE10A; PDE3A; PDE3B; PDE4A; PDE4B; PDE4C; PDE4D; BAD; GLI3; NFKB1; RELA; PPARA; NFATC1; CREBBP; EP300; SOX9; ABCC4; ADCY10; RRAS2; RRAS; PLD1; PLD2; LIPE; NPY; GHRL; GRIA1; GRIA2; GRIA3; GRIA4; GRIN3A; GRIN3B; GRIN1; GRIN2A; GRIN2B; GRIN2C; GRIN2D; ORAI1; FXYD1; HCN4; HCN2; PPP1R1B; PPP1CA; PPP1CB; PPP1CC; NFKBIA;

	AMH; BDNF; FOS; JUN; GLI1; PTCH1; HHIP; F2R; ACOX1; ACOX3; MYL9; TIAM1; VAV3; VAV1; VAV2; RAC1; RAC2; RAC3; PAK1; CGA; FSHB; OXT; POMC; GIP; NPPA; EDN1; EDN2; EDN3; SST; GCG; ADCYAP1; VIP; TSHB
Cytokine-cytokine receptor interaction	IL20RB; IL4; BMPR2; ACVR2B; ACVR1B; ACVR2A; IL10RB; IL22RA1; IL2RG; IL2RB; CSF2RB; LIFR; IL6ST; IL18RAP; IL18R1; IL1R2; IL1RAP; IL1R1; IL17RA; BMPR1A; BMPR1B; ACVR1; AMHR2; TGFBR1; TGFBR2; IL18; IL1B; IL1A; IL25; IL17B; IL17A; GDF5; BMP7; BMP2; AMH; INHBE; INHBC; INHBB; INHBA; TGFB3; TGFB2; TGFB1; RELT; TNFRSF19; EDA2R; EDAR; TNFRSF13C; TNFRSF13B; TNFRSF17; TNFRSF18; TNFRSF4; TNFRSF9; TNFRSF8; CD27; CD40; FAS; TNFRSF6B; TNFRSF14; LTBR; EDA; TNFSF13B; TNFSF13; TNFSF18; TNFSF4; TNFSF9; TNFSF8; CD70; CD40LG; FASLG; TNFSF14; LTB; LTA; IFNLR1; IL20RA; IL10RA; IFNGR2; IFNGR1; IFNAR2; IFNAR1; IL26; IFNL1; IFNL2; IFNL3; IL22; IL24; IL20; IL19; IL10; IFNG; IFNE; IFNK; IFNW1; IFNB1; IFNA1; IFNA2; IFNA4; IFNA5; IFNA6; IFNA7; IFNA8; IFNA10; IFNA13; IFNA14; IFNA16; IFNA17; IFNA21; MPL; PRLR; GHR; EPOR; CRLF2; IL7R; IL21R; IL15RA; IL9R; IL4R; IL2RA; IL5RA; IL3RA; CSF2RA; THPO; PRL; CSH1; CSH2; GH1; GH2; EPO; TSLP; IL21; IL15; IL9; IL7; IL2; IL5; IL3; CSF2; IL12RB1; IL23R; IL12RB2; IL13RA1; CSF3R; CNTFR; OSMR; IL11RA; IL6R; IL23A; IL12A; IL12B; IL13; CSF3; CTF1; CLCF1; CNTF; LIF; OSM; IL11; IL6; CX3CR1; XCR1; CXCR6; CXCR5; CXCR4; CXCR3; CXCR1; CXCR2; CX3CL1; XCL2; XCL1; CXCL14; PF4; PF4V1; CXCL16; CXCL13; CXCL12; CXCL11; CXCL10; CXCL9; CXCL8; CXCL6; CXCL5; PPBP; TNFRSF1A; TNFRSF1B; NGFR; TNFRSF21; TNFRSF12A; TNFRSF11A; TNFSF12; TNFRSF10C; TNFRSF11B; TNFRSF10B; TNFRSF10A; TNFSF10; TNFRSF10D; TNFSF15; TNF; CXCL1; CXCL2; CXCL3; TNFSF11; ACKR3; IL17RB; IL17RC; IL17RE; IL17F; IL17C; IL17D; ACKR4; CCR5; CCL4L1; CCL4; CCL4L2; CCL3L3; CCL3; CCL3L1; CCR7; CCL21; CCL19; CCR3; CCL26; CCL24; CCL11; CCL16; CCL14; CCR1; CCR2; CCR10; CCL7; CCL27; CCL28; CCR8; CCR9; CCL1; CCL25; CCL2; CCR4; CCL22; CCL17; CCL5; CCL8; CCL13; CCL15; CCL23; CCL18; CCR6; CCL20; CXCL17; IL27; IL27RA; IL31; IL31RA; IL1RN; IL36RN; IL36A; IL37; IL36B; IL36G; IL1F10; IL33; IL1RL2; IL1RL1; IL16; IL32; IL34; CSF1; CD4; CSF1R; TNFRSF25; NGF; ACVR1C; INHA; GDF1; GDF3; NODAL; BMP3; GDF10; GDF11; MSTN; GDF9; ACVRL1; GDF15; GDF2; BMP10; BMP4; GDF6; GDF7; BMP5; BMP6; BMP8A; BMP8B; BMP15; LEP; LEPR; IL13RA2
Viral protein interaction with cytokine and cytokine receptor	CCL3L3; CCL3; CCL3L1; CCL5; CCL7; CCL8; CCL13; CCL14; CCL15; CCL16; CCL23; CCR1; CCL27; CCL2; CCR2; CCL26; CCL28; CCL11; CCL24; CCR3; CCL17; CCL22; CCR4; CCL4L1; CCL4; CCL4L2; CCR5; CCL20; CCR6; CCL1; CCR8; CCL25; CCR9; ACKR4; CCR10; CCL18; CCL19; CCL21; CCR7; CX3CL1; CX3CR1; CXCL1; CXCL2; CXCL3; CXCL8; CXCL6; CXCL5; CXCR1; PPBP; CXCR2; CXCL13; CXCL10; CXCL9; PF4; PF4V1; CXCL11; CXCR3; CXCR5; CXCL12; ACKR3; CXCR4; CXCL14; XCL1; XCL2; XCR1; IL10RB; IL10RA; IL10; IL6ST; IL6R; IL6; IL2RG; IL2RB; IL2RA; IL2; IL18RAP; IL18R1; IL37; IL18; IL20RB; IL20RA; IL24; IL19; IL20; IL22RA1; IL34; CSF1; CSF1R; TNFRSF14; LTBR; TNFSF14; LTA; TNFRSF1A; TNFRSF1B; TNF; TNFRSF10D; TNFRSF10C; TNFRSF10B; TNFRSF10A; TNFSF10

Chemokine signaling pathway	CXCR6; CCR9; CCR1; CCR3; CCR4; CCR5; CCR6; CCR7; CCR8; CX3CR1; CCR10; XCR1; CXCR3; CXCR1; CXCR2; CXCR5; CCR2; CXCR4; CCL26; CXCL13; CCL27; CXCL1; CXCL2; CXCL3; CXCL8; CXCL10; CCL4L1; CCL3L3; CXCL9; PF4; PF4V1; PPBP; CCL28; CXCL16; CCL1; CCL2; CCL3; CCL3L1; CCL4; CCL5; CCL7; CCL8; CCL11; CCL13; CCL14; CCL15; CCL16; CCL17; CCL18; CCL19; CCL20; CCL21; CCL22; CCL23; CCL24; CCL25; CXCL6; CXCL11; CXCL5; XCL1; CX3CL1; CXCL12; XCL2; CXCL14; CCL4L2; JAK2; JAK3; PTK2; FGR; HCK; LYN; SRC; PXN; RHOA; PIK3CA; PIK3CB; PIK3CD; PIK3R1; PIK3R2; PIK3R3; PLCB1; PLCB2; PLCB3; PLCB4; PLCG1; PLCG2; SHC2; SHC4; SHC3; SHC1; GNAI1; GNAI2; GNAI3; GNB5; GNB1; GNB2; GNB3; GNG3; GNG4; GNG5; GNG7; GNG10; GNG11; GNGT1; GNGT2; GNG13; GNG2; GNG12; GNB4; GNG8; GRB2; BCAR1; ELMO1; CRK; CRKL; GRK7; GRK2; GRK3; GRK4; GRK5; GRK6; GRK1; ARRB1; ARRB2; ROCK1; ROCK2; VAV3; VAV1; VAV2; ITK; CDC42; ADCY1; ADCY2; ADCY3; ADCY5; ADCY6; ADCY7; ADCY8; ADCY9; ADCY4; RASGRP2; RAP1A; RAP1B; WAS; PRKCB; PRKCD; PARD3; PRKCZ; TIAM1; NCF1; PRKACA; PRKACB; PRKACG; PTK2B; PAK1; STAT1; STAT2; STAT3; STAT5B; MAPK1; MAPK3; AKT3; AKT1; AKT2; HRAS; KRAS; NRAS; MAP2K1; RAF1; BRAF; DOCK2; RAC1; RAC2; RAC3; NFKB1; RELA; CHUK; IKBKB; IKBKG; NFKBIA; NFKBIB; PREX1; SOS1; SOS2; FOXO3; GSK3A; GSK3B; BAD; PIK3R6; PIK3R5; PIK3CG; GNAQ
NF-kappa B signaling pathway	BCL2L1; BCL2; BIRC2; BIRC3; NFKBIA; IRAK1; IRAK4; MYD88; TRAF2; TRAF5; RIPK1; TRADD; IL1R1; TNFRSF1A; IL1B; TNF; TNFSF14; LTBR; TRAF3; MAP3K14; CHUK; IKBKG; IKBKB; NFKB2; RELB; NFKB1; RELA; MAP3K7; TNFSF13B; TNFRSF13C; CD40LG; CD40; LOC102723407; PLCG1; PRKCB; PRKCQ; CARD10; CARD14; CARD11; BCL10; MALT1; TRAF6; TNFAIP3; TRAF1; CXCL8; CCL13; CXCL12; TNFRSF11A; TNFSF11; TLR4; TAB1; TAB2; TAB3; DDX58; TIRAP; TICAM2; TICAM1; ZAP70; LAT; LY96; CD14; LBP; CSNK2A1; CSNK2A2; CSNK2B; CSNK2A3; PLAU; BCL2A1; PLCG2; SYK; LYN; BTK; LCK; BLNK; ATM; PARP1; PIAS4; UBE2I; CFLAR; XIAP; GADD45G; GADD45A; GADD45B; TRIM25; PIDD1; PTGS2; CCL4L1; CCL4; CCL4L2; VCAM1; CXCL1; CXCL2; CXCL3; CCL21; CCL19; ICAM1; LTA; LTB; ERC1; EDA; EDAR; EDARADD; EDA2R; CYLD
HIF-1 signaling pathway	CUL2; RBX1; ELOB; ELOC; VEGFA; EGLN2; EGLN3; EGLN1; VHL; ARNT; CREBBP; EP300; HIF1A; SLC2A1; EGF; IGF1; INS; EGFR; ERBB2; IGF1R; INSR; PIK3CA; PIK3CB; PIK3CD; PIK3R1; PIK3R2; PIK3R3; AKT3; AKT1; AKT2; MTOR; CYBB; PLCG1; PLCG2; PRKCA; PRKCB; PRKCG; LDHAL6A; LDHA; LDHB; LDHC; LDHAL6B; PDK1; PDHA1; PDHA2; PDHB; CAMK2A; CAMK2B; CAMK2D; CAMK2G; HK1; HK2; HK3; HKDC1; PFKL; PFKM; PFKP; GAPDH; ALDOA; ALDOB; ALDOC; ENO1; ENO2; ENO3; ENO4; PGK1; PGK2; PFKFB3; EPO; TF; TFRC; FLT1; SERPINE1; ANGPT1; ANGPT2; ANGPT4; TEK; TIMP1; EDN1; NOS2; NOS3; HMOX1; NPPA; LTBR; TLR4; NFKB1; RELA; IFNG; IFNGR1; IFNGR2; IL6; IL6R; STAT3; EIF4EBP1; EIF4E; EIF4E1B; EIF4E2; RPS6KB1; RPS6KB2; RPS6; MAPK1; MAPK3; MAP2K1; MAP2K2; MKNK2; MKNK1; BCL2
FoxO signaling pathway	CHUK; IKBKB; MAPK8; MAPK9; MAPK10; MAPK1; MAPK3; PRKAG2; PRKAG3; PRKAA1; PRKAA2; PRKAB1; PRKAB2; PRKAG1; INS; FOXO6; FOXO1; FOXO3;

	FOXO4; SOS1; SOS2; HRAS; KRAS; NRAS; MAP2K1; MAP2K2; INSR; ARAF; RAF1; BRAF; PCK1; PCK2; G6PC; G6PC2; G6PC3; AKT3; AKT1; AKT2; PIK3CA; PIK3CB; PIK3CD; PIK3R1; PIK3R2; PIK3R3; PDPK1; IRS1; IRS4; IRS2; EGFR; GRB2; STK4; EGF; PTEN; SKP2; MDM2; CREBBP; EP300; SIRT1; IGF1; IGF1R; CDK2; CCNB3; CCNB1; CCNB2; CCND1; CCND2; CDKN2B; CDKN2D; CDKN1A; CDKN1B; RBL2; PLK4; PLK2; PLK3; PLK1; GADD45G; GADD45A; GADD45B; FASLG; BCL2L11; TNFSF10; BCL6; CAT; SOD2; ATM; IL7R; KLF2; S1PR1; S1PR4; RAG1; RAG2; GRM1; HOMER3; HOMER2; HOMER1; AGAP2; TPTEP2-CSNK1E; CSNK1E; BNIP3; GABARAP; GABARAPL2; GABARAPL1; ATG12; C8orf44-SGK3; SGK2; SGK3; SGK1; TGFBR1; TGFBR2; SMAD3; SMAD4; FOXG1; STAT3; IL6; IL10; NLK; MAPK14; MAPK11; MAPK13; MAPK12; SETD7; PRMT1; USP7; FBXO32; FBXO25; SLC2A4; STK11; CCNG2
Phosphatidylinositol signaling system	PTEN; IPPK; PLCD3; PLCB1; PLCE1; PLCB2; PLCB3; PLCB4; PLCD1; PLCG1; PLCG2; PLCD4; PLCZ1; CALML6; CALML5; CALM1; CALM2; CALM3; CALML3; CALML4; ITPR1; ITPR2; ITPR3; CDIPT; PIK3CA; PIK3CB; PIK3CD; PIK3R1; PIK3R2; PIK3R3; PIP5K1C; PIP5K1A; PIP5K1B; PIKFYVE; PI4KA; PI4KB; PI4K2B; PI4K2A; PIK3C2A; PIK3C2B; PIK3C2G; CDS1; CDS2; DGKK; DGKA; DGKB; DGKG; DGKH; DGKQ; DGKZ; DGKE; DGKD; DGKI; INPP4A; INPP4B; INPP5B; OCRL; INPP5E; SYNJ1; SYNJ2; PIP4K2A; PIP4K2C; PIP4K2B; IPMK; MTM1; IMPA1; IMPA2; BPNT2; INPP1; INPP5A; ITPK1; ITPKA; ITPKB; ITPKC; INPP5D; INPPL1; MTMR8; MTMR14; MTMR1; MTMR3; MTMR2; MTMR6; MTMR7; MTMR4; PIP4P2; PIP4P1; IP6K3; IP6K2; IP6K1; PPIP5K2; PPIP5K1; PIK3C3; PRKCA; PRKCB; PRKCG
Phospholipase D signaling pathway	MAPK1; MAPK3; MAP2K1; MAP2K2; RAF1; RRAS2; MRAS; HRAS; KRAS; NRAS; RRAS; GRB2; SOS1; SOS2; AKT3; AKT1; AKT2; PIK3CA; PIK3CB; PIK3CD; PIK3R1; PIK3R2; PIK3R3; SHC2; SHC4; SHC3; SHC1; KITLG; EGFR; INSR; KIT; PDGFRA; PDGFRB; GAB1; GAB2; PTPN11; RALGDS; RALA; RALB; RHOA; LPAR6; AGTR1; LPAR1; F2R; LPAR3; LPAR4; GRM1; GRM2; GRM3; GRM4; GRM5; GRM6; GRM7; GRM8; CXCR1; CXCR2; AVPR1A; AVPR1B; AVPR2; LPAR5; PTGFR; LPAR2; PLCG1; PLCG2; RAPGEF3; RAPGEF4; PRKCA; PLD1; PLD2; PLCB1; PLCB2; PLCB3; PLCB4; TSC1; TSC2; RHEB; MTOR; PIP5K1C; PIP5K1A; PIP5K1B; GNA13; PIK3R6; PIK3R5; PIK3CG; CYTH4; CYTH3; CYTH2; CYTH1; ARF1; GNAS; ADCY1; ADCY2; ADCY3; ADCY5; ADCY6; ADCY7; ADCY8; ADCY9; ADCY4; PTK2B; GNA12; PLA2G4B; PLA2G4E; PLA2G4F; PLA2G4D; PLA2G4A; PLA2G4C; JMJD7-PLA2G4B; PLPP1; PLPP2; PLPP3; DGKK; DGKA; DGKB; DGKG; DGKH; DGKQ; DGKZ; DGKE; DGKD; DGKI; SPHK2; SPHK1; PDGFA; PDGFB; PDGFC; PDGFD; INS; EGF; DNM1; DNM2; DNM3; CXCL8; LOC102723407; FYN; FCER1A; MS4A2; FCER1G; SYK; AVP; ARF6; AGPAT1; AGPAT2; AGPAT5; AGPAT3; AGPAT4; F2; AGT
Neuroactive ligand-receptor interaction	CTSG; F2; GZMA; PLG; PRSS1; PRSS2; PRSS3; MAS1; MCHR1; MCHR2; CGA; TSHB; LHB; FSHB; TACR3; TACR2; KISS1R; PRL; PRLR; LEPR; THRA; THRB; LEP; CSH1; CSH2; GH1; GH2; GHR; NR3C1; TSPO; TRPV1; GLRA1; GLRA2; GLRB; GLRA3; GRIN3A; GRIN3B; GRIA1; GRIA2; GRIA3; GRIA4; GRID1; GRID2; GRIK1; GRIK2; GRIK3; GRIK4; GRIK5; GRIN1; GRIN2A; GRIN2B; GRIN2C; GRIN2D;

	P2RX2; P2RX1; P2RX3; P2RX4; P2RX5; P2RX7; P2RX6; GABRR3; GABRA1; GABRA2; GABRA3; GABRA4; GABRA5; GABRA6; GABRB1; GABRB2; GABRB3; GABRD; GABRE; GABRG1; GABRG2; GABRG3; GABRP; GABRR1; GABRR2; GABRQ; PRLHR; NPBWR1; NPBWR2; MLNR; GPR156; GABBR1; GABBR2; GRM1; GRM2; GRM3; GRM4; GRM5; GRM6; GRM7; GRM8; VIPR1; VIPR2; SCTR; ADCYAP1R1; PTH1R; PTH2R; GHRHR; GLP1R; GLP2R; GCGR; GIPR; CRHR1; CRHR2; CALCRL; CALCR; CYSLTR1; CYSLTR2; RXFP2; RXFP4; RXFP3; RXFP1; LTB4R; LTB4R2; S1PR1; S1PR3; S1PR5; S1PR4; S1PR2; LPAR1; LPAR3; LPAR4; LPAR2; MTNR1A; MTNR1B; GPR50; TRHR; GNRHR; PTAFR; CNR1; CNR2; LPAR6; P2RY10; GPR35; P2RY8; P2RY1; P2RY2; P2RY4; P2RY6; P2RY11; P2RY13; P2RY14; ADORA1; ADORA2A; ADORA2B; ADORA3; TBXA2R; PTGIR; PTGFR; PTGER1; PTGER2; PTGER3; PTGER4; PTGDR; TSHR; LHCGR; FSHR; NMUR1; NMUR2; UTS2R; NPFFR2; NPFFR1; HCRTR1; HCRTR2; F2R; F2RL1; F2RL2; PARD3; F2RL3; GALR1; GALR3; GALR2; OXTR; AVPR1A; AVPR1B; AVPR2; TACR1; SSTR1; SSTR2; SSTR3; SSTR4; SSTR5; OPRD1; OPRK1; OPRL1; OPRM1; NTSR2; NTSR1; NPY4R2; GPR83; NPY1R; NPY2R; NPY5R; NPY4R; MC1R; MC2R; MC3R; MC4R; MC5R; GHSR; EDNRA; EDNRB; CCKAR; CCKBR; APLNR; FPR1; FPR2; FPR3; C5AR1; C3AR1; BDKRB1; BDKRB2; GRPR; NMBR; BRS3; AGTR1; AGTR2; TAAR9; TAAR1; TAAR6; TAAR8; TAAR5; TAAR2; HTR1A; HTR1B; HTR1D; HTR1E; HTR1F; HTR2A; HTR2B; HTR2C; HTR4; HTR5A; HTR6; HTR7; HRH3; HRH1; HRH2; HRH4; DRD1; DRD2; DRD3; DRD4; DRD5; ADRA1D; ADRA1B; ADRA1A; ADRA2A; ADRA2B; ADRA2C; ADRB1; ADRB2; ADRB3; CHRM1; CHRM2; CHRM3; CHRM4; CHRM5; CHRNA1; CHRNA2; CHRNA3; CHRNA4; CHRNA5; CHRNA7; CHRNB1; CHRNB2; CHRNB3; CHRNB4; CHRND; CHRNE; CHRNG; CHRNA9; CHRNA10; CHRNA6; AGT; APLN; GRP; NMB; KNG1; C3; C5; CCK; EDN1; EDN2; EDN3; GAL; GHRL; KISS1; POMC; MLN; NMU; NPFF; NPY; PPY; PYY; NPB; NPW; NTS; HCRT; OXT; CORT; SST; TAC4; TAC1; TAC3; UTS2; UTS2B; AVP; PRLH; PMCH; GNRH1; GNRH2; INSL5; RLN3; INSL3; RLN1; RLN2; TRH; ADM; IAPP; CALCA; CALCB; UCN3; CRH; UCN; UCN2; GIP; GCG; GHRH; PTH2; PTH; ADCYAP1; SCT; VIP; PDYN; PENK
Cell cycle	CDKN2A; FZR1; MCM2; MCM3; MCM4; MCM5; MCM6; MCM7; ORC6; ORC3; ORC1; ORC2; ORC4; ORC5; ANAPC10; CDC26; ANAPC13; ANAPC2; ANAPC4; ANAPC5; ANAPC7; ANAPC11; ANAPC1; CDC23; CDC16; CDC27; SKP1; CUL1; RBX1; CDK1; BUB1B; PCNA; PLK1; ATM; ATR; DBF4; MAD2L2; MAD2L1; BUB1; BUB3; CDC14B; CDC14A; YWHAQ; YWHAB; YWHAE; YWHAG; YWHAH; YWHAZ; SFN; CHEK1; CHEK2; CDKN1A; PRKDC; MDM2; CREBBP; EP300; SKP2; PKMYT1; WEE2; WEE1; PTTG2; PTTG1; ESPL1; SMC1B; SMC1A; RB1; GADD45G; GADD45A; GADD45B; TP53; CDKN1B; CDKN1C; CDKN2B; TGFB1; TGFB2; TGFB3; SMAD4; SMAD2; SMAD3; CDC7; CDC20; CDC25B; CDC25C; CDC45; CDC6; CDC25A; GSK3B; CCNB3; CCNB1; CCNB2; CCNA2; CCNA1; CDK7; CDK2; CDK4; CDK6; CCNH; CCNE1; CCNE2; CCND1; CCND2; CCND3; MAD1L1; TTK; CDKN2C; CDKN2D; SMC3; STAG1; STAG2; RAD21; RBL1; E2F4; E2F5; MYC; ZBTB17; ABL1; TFDP1; TFDP2; E2F1; E2F2; E2F3; HDAC1; HDAC2; RBL2
Oocyte meiosis	MOS; CDK1; MAD2L2; MAD2L1; MAD1L1; CCNB2; PKMYT1; RPS6KA6;

	RPS6KA1; RPS6KA2; RPS6KA3; MAPK1; MAPK3; MAP2K1; ANAPC10; CDC26; ANAPC13; ANAPC2; ANAPC4; ANAPC5; ANAPC7; ANAPC11; ANAPC1; CDC23; CDC16; CDC27; CDC20; PTTG2; PTTG1; CCNB1; FBXO5; SKP1; CUL1; RBX1; CCNE1; CCNE2; FBXO43; CDC25C; CDK2; FBXW11; BTRC; CAMK2A; CAMK2B; CAMK2D; CAMK2G; PLK1; AURKA; PLCZ1; ITPR1; ITPR2; ITPR3; CALML6; CALML5; CALM1; CALM2; CALM3; CALML3; CALML4; PPP3CA; PPP3CB; PPP3CC; PPP3R1; PPP3R2; CPEB2; CPEB3; CPEB1; CPEB4; PRKACA; PRKACB; PRKACG; ADCY1; ADCY2; ADCY3; ADCY5; ADCY6; ADCY7; ADCY8; ADCY9; ADCY4; YWHAQ; YWHAB; YWHAE; YWHAG; YWHAH; YWHAZ; PPP1CA; PPP1CB; PPP1CC; MAPK14; MAPK11; MAPK13; MAPK12; ESPL1; SMC1B; SMC1A; REC8; STAG3; SGO1; PPP2R5A; PPP2R5B; PPP2R5C; PPP2R5D; PPP2R5E; PPP2CA; PPP2CB; PPP2R1A; PPP2R1B; SMC3; PGR; AR; IGF1R; INS; IGF1; SPDYE2B; SPDYE18; SPDYE11; SPDYE16; SPDYE17; SPDYE15; SPDYA; SPDYE1; SPDYC; SPDYE4; SPDYE3; SPDYE2; SPDYE5; SPDYE6; SLK; BUB1
p53 signaling pathway	SIAH1; IGFBP3; GADD45G; GADD45A; GADD45B; MDM4; CHEK1; ATR; CHEK2; ATM; RPRM; SFN; CDK2; CCNE1; CCNE2; TSC2; PPM1D; TP73; CCNG1; CCNG2; COP1; RRM2B; RRM2; DDB2; STEAP3; PTEN; SERPINB5; CD82; THBS1; IGF1; SERPINE1; ADGRB1; CDKN2A; CDKN1A; ZMAT3; BID; EI24; TP53I3; FAS; GTSE1; BAX; PIDD1; TNFRSF10B; TNFRSF10A; CASP8; CDK1; APAF1; CYCS; CASP9; MDM2; TP53; RCHY1; CDK4; CDK6; CCND1; CCND2; CCND3; PERP; SHISA5; SESN3; SESN1; SESN2; CCNB1; CCNB2; CASP3; TP53AIP1; BBC3; PMAIP1; GORAB; SIVA1; BCL2L1; BCL2; AIFM2
Ubiquitin mediated proteolysis	UBA2; SAE1; ELOC; ELOB; RBX1; FBXW11; FBXO2; FBXO4; FBXW7; SKP2; BTRC; RHOBTB2; KLHL9; KLHL13; KEAP1; RHOBTB1; ERCC8; DDB2; DET1; COP1; SOCS1; SOCS3; VHL; HERC4; HERC3; UBE2W; UBE2F; UBA3; CDC26; ANAPC10; CDC23; CDC16; ANAPC7; ANAPC5; ANAPC4; CDC27; ANAPC1; FZR1; CDC20; ANAPC2; ANAPC11; FBXW8; CUL3; CUL2; SKP1; CUL1; CUL7; CUL5; RNF7; DDB1; CUL4B; CUL4A; TRIM37; TRIM32; MID1; FANCL; BRCA1; MGRN1; AIRE; NHLRC1; SYVN1; PIAS3; PIAS4; PIAS1; PIAS2; BIRC2; BIRC3; XIAP; BIRC7; RCHY1; MAP3K1; TRAF6; PML; SIAH1; PRKN; CBLC; CBL; CBLB; MDM2; UBOX5; PRPF19; UBE3A; UBE2U; UBE2D4; UBE2D1; UBE2D2; UBE2D3; UBE2NL; UBE2N; WWP1; SMURF1; SMURF2; UBE3C; UBE2J1; UBE3B; UBE2S; UBE2C; UBE2A; ITCH; UBE2K; UBE2J2; UBE2L6; UBE2L3; UBE2G2; UBE2G1; UBA6; UBA1; UBA7; PPIL2; HERC2; UBE2R2; CDC34; STUB1; HERC1; WWP2; UBE4B; UBR5; TRIP12; UBE4A; HUWE1; UBE2Z; BIRC6; NEDD4L; NEDD4; UBE2QL1; UBE2Q1; UBE2Q2; UBE2O; UBE2M; UBE2I; UBE2H; UBE2B; ANAPC13; UBE2E3; UBE2E1; UBE2E2
Sulfur relay system	MOCS3; URM1; NFS1; CTU2; CTU1; MPST; TST; MOCS2
Mitophagy - animal	PINK1; MFN1; MFN2; USP30; USP15; AMBRA1; USP8; PRKN; OPTN; TBK1; SQSTM1; CALCOCO2; NBR1; GABARAP; GABARAPL2; GABARAPL1; SRC; ULK1; FUNDC1; CSNK2A1; CSNK2A2; CSNK2B; CSNK2A3; PGAM5; BCL2L1; BNIP3; BNIP3L; RHOT1; RHOT2; BECN2; BECN1; TOMM7; ATG5; ATG9B; ATG9A; TFEB; MITF; TFE3; TAX1BP1; TBC1D15; TBC1D17; RAB7B; RAB7A; FIS1; BCL2L13; EIF2AK3; ATF4; HIF1A; E2F1; RELA; TP53; RRAS2; MRAS; HRAS;

	KRAS; NRAS; RRAS; SP1; MAPK8; MAPK9; MAPK10; JUN; FOXO3; CITED2; RPS27A; UBA52; UBB; UBC
Autophagy - animal	IGF1R; INS; IRS1; IRS4; IRS2; PIK3CA; PIK3CB; PIK3CD; PIK3R1; PIK3R2; PIK3R3; PTEN; PDPK1; AKT3; AKT1; AKT2; RRAS2; MRAS; HRAS; KRAS; NRAS; RRAS; RAF1; MAP2K1; MAP2K2; MAPK1; MAPK3; HIF1A; DDIT4; BNIP3; PRKCD; TSC2; TSC1; RHEB; RPTOR; DEPTOR; MTOR; AKT1S1; MLST8; RPS6KB1; RPS6KB2; PPP2CA; PPP2CB; IGBP1; ULK1; ULK2; ATG13; RB1CC1; ATG101; ATG2A; ATG2B; WIPI2; WIPI1; RRAGB; RRAGA; RRAGD; RRAGC; STK11; PRKAA1; PRKAA2; CAMKK2; MAP3K7; ERN1; MAPK8; MAPK9; MAPK10; PRKACA; PRKACB; PRKACG; TP53INP2; TRAF6; AMBRA1; ITPR1; BCL2; BCL2L1; ATG9B; ATG9A; SUPT20H; STX17; BECN2; BECN1; PIK3R4; NRBF2; PIK3C3; ATG14; UVRAG; SH3GLB1; RUBCN; ZFYVE1; MTMR3; MTMR4; MTMR14; BAD; HMGB1; PRAP1; EIF2AK3; EIF2AK4; DAPK1; DAPK3; DAPK2; EIF2S1; ATG12; ATG5; RAB33B; ATG7; ATG10; ATG16L1; ATG16L2; CFLAR; ATG3; PRKCQ; GABARAP; GABARAPL2; GABARAPL1; ATG4A; ATG4B; ATG4C; ATG4D; LAMP1; LAMP2; RAB7B; RAB7A; VAMP8; SNAP29; CTSD; CTSL; CTSB; VMP1; C9orf72; RAB1A; WDR41; SMCR8; RAB39B; RAB8A; SQSTM1; TBK1; TANK
Protein processing in endoplasmic reticulum	ATF4; DDIT3; MAPK8; MAPK9; MAPK10; TRAF2; MAP3K5; MAP2K7; EIF2S1; CANX; CALR; PPP1R15A; PDIA3; CASP12; CAPN1; CAPN2; SEC61B; SEC61G; SEC61A1; SEC61A2; VCP; DAD1; DDOST; STT3B; STT3A; RPN1; RPN2; TUSC3; MMP24-AS1-EDEM2; EDEM2; EDEM3; EDEM1; SEC24B; SEC23B; SEC23A; SEC24A; SEC24C; SEC24D; LMAN2; LMAN1; UFD1; NPLOC4; ERO1A; ERO1B; MAN1A2; MAN1B1; MAN1A1; MAN1C1; GANAB; PRKCSH; MOGS; UGGT2; UGGT1; MARCHF6; SYVN1; UBE2G1; UBE2G2; HSPA5; SEC63; SEC62; BAK1; BAX; ATF6B; ATF6; ERN1; EIF2AK1; EIF2AK4; EIF2AK2; EIF2AK3; MBTPS1; MBTPS2; XBP1; WFS1; RAD23A; RAD23B; UBQLN2; UBQLN1; UBQLN3; UBQLN4; NGLY1; UBE2J2; UBE2J1; UBE4B; YOD1; ATXN3; ATXN3L; PLAA; HSPA1A; HSPA1B; HSPA1L; HSPA2; HSPA6; HSPA8; SEL1L; SEL1L2; DERL2; DERL1; DERL3; OS9; HERPUD1; ERLEC1; SELENOS; RBX1; FBXO2; FBXO6; UBE2D4; UBE2D1; UBE2D2; UBE2D3; DNAJA2; DNAJC5G; DNAJB2; DNAJA1; DNAJB1; DNAJB12; DNAJC5; DNAJC5B; STUB1; PDIA6; ERP29; P4HB; TXNDC5; PDIA4; DNAJB11; DNAJC10; DNAJC3; DNAJC1; UBXN2A; UBXN4; UBXN1; NSFL1C; UBXN8; UBXN6; SVIP; SKP1; CUL1; RNF5; RNF185; HSP90AA1; HSP90AB1; NFE2L2; SEC31A; SEC31B; SEC13; SAR1B; SAR1A; PREB; HSP90B1; BCL2; LMAN1L; TRAM1L1; TRAM1; PRKN; HSPH1; HSPA4L; HSPBP1; BAG1; BAG2; CRYAA2; CRYAA; CRYAB; HYOU1; SIL1; CKAP4; RRBP1; BCAP31; SSR1; SSR2; SSR3; SSR4; AMFR
Lysosome	TCIRG1; ATP6V0A2; ATP6V0D2; ATP6V0A4; ATP6V1H; ATP6V0C; ATP6V0B; ATP6V0A1; ATP6AP1; ATP6V0D1; CTSC; CTSB; CTSD; CTSE; CTSG; CTSH; CTSK; CTSL; CTSV; CTSO; CTSS; CTSW; CTSZ; CTSA; CTSF; NAPSA; LGMN; TPP1; GLA; GLB1; GAA; GBA; HEXA; HEXB; MANBA; MAN2B1; NAGA; GUSB; NEU1; GALC; NAGLU; IDUA; FUCA1; FUCA2; ARSG; ARSA; ARSB; GNS; IDS; SGSH; GALNS; LIPA; PLA2G15; DNASE2; DNASE2B; ACP2; SMPD1; ASAH1; AGA;

	GNPTAB; GNPTG; GM2A; PSAP; PSAPL1; PPT1; PPT2; LAMP3; LAMP1; LAMP2; CD68; SCARB2; CD63; CTNS; SLC17A5; LAPTM4B; LAPTM5; LAPTM4A; ABCB9; ABCA2; SLC11A2; SLC11A1; NPC2; NPC1; CLN3; CLN5; CD164; ENTPD4; SORT1; MFSD8; HGSNAT; CLTA; CLTB; CLTC; CLTCL1; IGF2R; M6PR; NAGPA; AP1M2; AP1S1; AP1S3; AP1B1; AP1G1; AP1S2; AP1G2; AP1M1; AP3S2; AP3M2; AP3S1; AP3M1; AP3B2; AP3B1; AP3D1; GGA2; GGA3; GGA1; ACP5; AP4B1; AP4S1; AP4E1; AP4M1; HYAL4; HYAL1; SPAM1; HYAL3; HYAL2; SUMF1; MCOLN1; LITAF
Endocytosis	STAM2; STAM; HGS; STAMBP; USP8; DNM1; DNM2; DNM3; RNF41; WWP1; NEDD4L; CBLC; MDM2; NEDD4; SMURF1; SMURF2; TRAF6; ITCH; CBL; CBLB; LDLR; CLTA; CLTB; CLTC; CLTCL1; AP2M1; AP2S1; AP2A1; AP2A2; AP2B1; MVB12B; MVB12A; VPS37A; VPS37D; VPS37C; VPS37B; VPS28; HSPA1A; HSPA1B; HSPA1L; HSPA2; HSPA6; HSPA8; DNAJC6; VPS36; TSG101; VPS25; SNF8; CHMP6; RNF103-CHMP3; CHMP3; CHMP4B; CHMP4A; CHMP4C; CHMP1A; CHMP1B; CHMP2B; CHMP2A; PDCD6IP; VPS4A; VPS4B; VTA1; CHMP5; CDC42; PRKCI; PRKCZ; PARD6A; PARD6G; PARD6B; PARD3; ARF6; HRAS; SRC; RAB11A; RAB11B; TFRC; EPN2; EPN1; EPN3; EPS15; EPS15L1; RAB5A; RAB5B; RAB5C; LDLRAP1; DAB2; CCR5; CXCR1; CXCR2; CXCR4; EGFR; FGFR3; FGFR2; FGFR4; IGF1R; PDGFRA; SH3KBP1; SH3GLB1; SH3GLB2; SH3GL1; SH3GL2; SH3GL3; GRK7; GRK2; GRK3; GRK4; GRK5; GRK6; GRK1; ARRB1; ARRB2; RAB11FIP2; RAB11FIP5; RAB11FIP1; PLD1; PLD2; PIP5KL1; PIP5K1C; PIP5K1A; PIP5K1B; EEA1; RABEP1; ARFGEF2; ARFGEF1; IQSEC2; PSD3; PSD4; CYTH4; IQSEC3; PSD; PSD2; GBF1; CYTH3; CYTH2; CYTH1; IQSEC1; ACAP3; ARAP2; ARAP1; AGAP2; AGAP1; AGAP3; AGAP4; AGAP11; ACAP2; ARFGAP3; GIT1; AGAP6; ASAP1; ASAP3; ARFGAP1; SMAP1; AGAP9; ARAP3; SMAP2; AGAP5; ARFGAP2; ASAP2; ACAP1; GIT2; EHD3; EHD1; RAB4A; EHD4; EHD2; RBSN; RUFY1; VPS45; HLA-A; HLA-B; HLA-C; HLA-E; HLA-F; HLA-G; RAB7A; TGFBR1; TGFBR2; ZFYVE9; ZFYVE16; SMAD2; SMAD3; PML; CAV1; CAV2; CAV3; IL2RA; IL2RB; IL2RG; RHOA; FOLR1; FOLR2; FOLR3; IZUMO1R; CHMP7; ARF1; ARF3; ARF4; ARF5; ZFYVE27; KIF5A; KIF5B; KIF5C; WASHC1; WASHC2C; WASHC2A; WASHC3; WASHC4; WASHC5; CAPZA1; CAPZA2; CAPZA3; SPART; CAPZB; RAB35; RAB10; SNX4; VPS26B; VPS26A; RAB31; RAB22A; VPS29; VPS35; RAB8A; RAB11FIP4; RAB11FIP3; AMPH; BIN1; SPG21; ARPC5; ARPC4; ARPC3; ARPC1B; ARPC2; ARPC1A; ARPC5L; SNX12; SNX3; SNX1; SNX2; SNX32; SNX5; SNX6; IGF2R; WASL; WIPF2; WIPF3; WIPF1; IST1
Peroxisome	PEX14; PEX2; PEX1; PEX6; PEX26; PEX13; PEX10; PEX12; PEX5L; PEX5; PEX7; PEX19; PEX11B; PEX11A; PEX11G; PEX16; PEX3; ABCD3; GNPAT; AGPS; PHYH; ACAA1; ACOX1; ACOX2; ACOX3; HSD17B4; CAT; SCP2; PIPOX; AGXT; AMACR; MVK; ECH1; PAOX; PMVK; CROT; BAAT; HMGCL; HMGCLL1; CRAT; DAO; DDO; DHRS4; DHRS4L1; MLYCD; HACL1; EHHADH; PECR; ECI2; NUDT12; ACOT8; NUDT19; DECR2; ABCD1; ABCD2; ABCD4; ACSL1; ACSL3; ACSL4; ACSL6; ACSL5; SLC27A2; IDH1; IDH2; HAO2; HAO1; XDH; FAR2; FAR1; PRDX5; SOD1; SOD2; GSTK1; EPHX2; NOS2; PRDX1; PXMP2; MPV17L; MPV17; MPV17L2; PXMP4; SLC25A17; NUDT7

AMPK signaling pathway	PRKAG2; PRKAG3; PRKAA1; PRKAA2; PRKAB1; PRKAB2; PRKAG1; INS; INSR; AKT3; AKT1; AKT2; PIK3CA; PIK3CB; PIK3CD; PIK3R1; PIK3R2; PIK3R3; PDPK1; IRS1; IRS4; IRS2; IGF1; IGF1R; FOXO1; FOXO3; SLC2A4; STK11; SIRT1; PPARGC1A; ADIPOR1; ADIPOR2; CAMKK2; ACACA; CPT1C; CPT1A; CPT1B; PFKFB1; PFKFB2; PFKFB3; PFKFB4; PFKL; PFKM; PFKP; FBP1; FBP2; TSC2; MTOR; RPTOR; AKT1S1; ADIPOQ; GYS1; GYS2; HNF4A; PCK1; PCK2; G6PC; G6PC2; G6PC3; SREBF1; FASN; SCD; SCD5; EEF2K; EEF2; RPS6KB1; RPS6KB2; EIF4EBP1; PPARG; ACACB; CRTC2; TBC1D1; STRADB; STRADA; CAB39; CAB39L; PPP2R3B; PPP2R3C; PPP2CA; PPP2CB; PPP2R1A; PPP2R1B; PPP2R2A; PPP2R2B; PPP2R2C; PPP2R3A; PPP2R5A; PPP2R5B; PPP2R5C; PPP2R5D; PPP2R5E; PPP2R2D; ELAVL1; CCND1; CCNA2; CCNA1; LIPE; HMGCR; ADRA1A; LEPR; LEP; CD36; CFTR; RAB10; RAB8A; RAB14; RAB2A; RAB11B; RHEB; TSC1; MLYCD; MAP3K7; CREB3; CREB1; CREB3L4; CREB3L2; CREB3L3; CREB3L1; CREB5; ULK1
Apoptosis	BCL2L1; DFFA; ATM; TP53; ENDOG; AIFM1; BCL2; BIRC2; BIRC3; XIAP; BIRC5; DFFB; CASP9; CASP7; CASP3; BAD; NFKB1; RELA; CASP6; NFKBIA; APAF1; CYCS; BID; CASP8; CASP10; CAPN1; CAPN2; AKT3; AKT1; AKT2; MAP3K14; CHUK; IKBKB; IKBKG; CFLAR; PIK3CA; PIK3CB; PIK3CD; PIK3R1; PIK3R2; PIK3R3; TRAF2; RIPK1; TRADD; FADD; CSF2RB; IL3RA; NTRK1; TNFRSF1A; TNFRSF10B; TNFRSF10A; FAS; IL3; NGF; TNF; TNFSF10; FASLG; SPTA1; SPTAN1; LMNA; LMNB1; LMNB2; PARP2; PARP3; PARP1; PARP4; DIABLO; SEPTIN4; HTRA2; BAX; BAK1; PMAIP1; BBC3; TP53AIP1; ERN1; CASP12; PTPN13; GADD45G; GADD45A; GADD45B; BCL2A1; TRAF1; DAXX; MAP3K5; MAPK8; MAPK9; MAPK10; DAB2IP; JUN; FOS; BCL2L11; CTSC; CTSB; CTSD; CTSH; CTSK; CTSL; CTSV; CTSO; CTSS; CTSW; CTSZ; CTSF; DDIT3; EIF2AK3; EIF2S1; ATF4; ITPR1; ITPR2; ITPR3; PDPK1; CASP2; PIDD1; GZMB; PRF1; ACTB; ACTG1; TUBA1B; TUBA3E; TUBA3D; TUBA8; TUBA4A; TUBA3C; TUBA1A; TUBAL3; TUBA1C; MCL1; HRK; HRAS; KRAS; NRAS; RAF1; MAP2K1; MAP2K2; MAPK1; MAPK3
Longevity regulating pathway	FOXO1; FOXO3; RPS6KB1; RPS6KB2; MTOR; RPTOR; AKT1S1; AKT3; AKT1; AKT2; PIK3CA; PIK3CB; PIK3CD; PIK3R1; PIK3R2; PIK3R3; IGF1; IGF1R; IRS1; IRS4; IRS2; HRAS; KRAS; NRAS; PRKACA; PRKACB; PRKACG; ADCY1; ADCY2; ADCY3; ADCY5; ADCY6; ADCY7; ADCY8; ADCY9; ADCY4; PRKAG2; PRKAG3; PRKAA1; PRKAA2; PRKAB1; PRKAB2; PRKAG1; INS; INSR; CREB3; CREB1; ATF2; ATF6B; CREB3L4; ATF4; CREB3L2; CREB3L3; CREB3L1; CREB5; SOD2; TSC2; TSC1; RHEB; EIF4EBP1; EIF4E; EIF4E1B; EIF4E2; STK11; TP53; SESN3; SESN1; SESN2; ADIPOQ; ADIPOR1; ADIPOR2; BAX; SIRT1; PPARGC1A; ULK1; KL; CAMKK2; CAMK4; NFKB1; RELA; PPARG; CAT; ATG5; APPL1; RB1CC1; ATG13; ATG101
Longevity regulating pathway - multiple species	FOXO1; FOXO3; RPS6KB1; RPS6KB2; MTOR; RPTOR; AKT1S1; AKT3; AKT1; AKT2; PIK3CA; PIK3CB; PIK3CD; PIK3R1; PIK3R2; PIK3R3; IGF1; IGF1R; IRS1; IRS4; IRS2; HRAS; KRAS; NRAS; INS; INSR; PRKACA; PRKACB; PRKACG; EIF4EBP2; FOXA2; ADCY1; ADCY2; ADCY3; ADCY5; ADCY6; ADCY7; ADCY8; ADCY9; ADCY4; PRKAG2; PRKAG3; PRKAA1; PRKAA2; PRKAB1; PRKAB2;

	PRKAG1; SOD2; SOD1; CRYAB; HSPA1A; HSPA1B; HSPA1L; HSPA2; HSPA6; HSPA8; CLPB; CAT; ATG5; SIRT1
Apoptosis - multiple species	BIRC7; CASP7; APAF1; BOK; CYCS; CASP9; CASP3; BIRC2; BIRC3; XIAP; BIRC5; DIABLO; HTRA2; SEPTIN4; BAX; BAK1; BCL2; BCL2L1; BIRC6; BECN2; BECN1; FADD; CASP8; TNFRSF1A; MAPK8; MAPK9; MAPK10; NGFR
Ferroptosis	SLC7A11; TP53; GPX4; VDAC2; VDAC3; GCLC; GCLM; SLC3A2; TFRC; TF; CP; STEAP3; GSS; PCBP1; PCBP2; SLC40A1; FTH1; FTL; ATG7; ATG5; SLC39A14; SLC39A8; SLC11A2; MAP1LC3C; MAP1LC3B2; MAP1LC3B; MAP1LC3A; NCOA4; SAT2; SAT1; PRNP; HMOX1; CYBB; ACSL1; ACSL3; ACSL4; ACSL6; ACSL5; LPCAT3; ALOX15; FTMT
Necroptosis	TNF; TNFRSF1A; TRADD; TRAF2; TRAF5; MLKL; TLR4; IFNAR1; IFNAR2; IFNGR1; IFNGR2; IFNA1; IFNA2; IFNA4; IFNA5; IFNA6; IFNA7; IFNA8; IFNA10; IFNA13; IFNA14; IFNA16; IFNA17; IFNA21; IFNB1; IFNG; JAK1; JAK2; JAK3; TYK2; TNFRSF10B; TNFRSF10A; TNFSF10; FAS; FASLG; BIRC2; BIRC3; XIAP; CASP8; CFLAR; CYLD; TRPM7; TLR3; RIPK1; FADD; TNFAIP3; FAF1; EIF2AK2; RIPK3; TICAM1; RBCK1; RNF31; SHARPIN; TICAM2; IRF9; STAT1; STAT2; STAT3; STAT4; STAT5A; STAT5B; STAT6; IL33; IL1A; HMGB1; HSP90AA1; HSP90AB1; ZBP1; SPATA2L; SPATA2; USP21; PYGB; PYGL; PYGM; GLUL; GLUD1; GLUD2; SLC25A4; SLC25A5; SLC25A6; SLC25A31; SMPD1; PGAM5; DNM1L; NLRP3; IL1B; PYCARD; CASP1; MAPK8; MAPK9; MAPK10; FTH1; FTL; CAPN1; CAPN2; BID; H2AC1; H2AC8; H2AC7; H2AX; H2AZ1; H2AC21; H2AB2; H2AB1; MACROH2A2; H2AJ; H2AC19; H2AC13; H2AC15; H2AC14; H2AC16; H2AC6; H2AC4; H2AC17; H2AC18; H2AC20; H2AB3; H2AC12; H2AC11; H2AW; H2AZ2; MACROH2A1; AIFM1; BAX; PARP1; BCL2; PPIA; PLA2G4B; PLA2G4E; PLA2G4F; PLA2G4D; PLA2G4A; PLA2G4C; JMJD7-PLA2G4B; CAMK2A; CAMK2B; CAMK2D; CAMK2G; CYBB; RNF103-CHMP3; CHMP4B; CHMP2B; VPS4A; CHMP2A; CHMP4A; CHMP1A; CHMP5; CHMP3; CHMP1B; CHMP6; CHMP7; CHMP4C; VPS4B; SQSTM1; ALOX15; VDAC1; VDAC2; VDAC3; PPID
Cellular senescence	RASSF5; PPP1CA; PPP1CB; PPP1CC; MYC; HIPK3; HIPK4; HIPK1; HIPK2; CDKN2A; MDM2; TP53; PIK3CA; PIK3CB; PIK3CD; PIK3R1; PIK3R2; PIK3R3; AKT3; AKT1; AKT2; TSC1; TSC2; RHEB; MTOR; FOXO3; SIRT1; RRAS2; MRAS; HRAS; KRAS; NRAS; RRAS; MAP2K1; MAP2K2; ETS1; RAF1; CDK4; CDK6; CCND1; CCND2; CCND3; MAPK1; MAPK3; CDKN1A; CDK2; CCNE1; CCNE2; RB1; RBL1; RBL2; E2F1; E2F2; E2F3; E2F4; E2F5; MAPK14; MAPK11; MAPK13; MAPK12; MAP2K3; MAP2K6; CCNA2; CCNA1; MYBL2; FOXM1; LIN54; LIN9; LIN37; RBBP4; LIN52; ATM; CHEK2; MRE11; RAD50; NBN; GADD45G; GADD45A; GADD45B; ATR; CDC25A; CHEK1; RAD9B; RAD9A; RAD1; HUS1; CDK1; CCNB3; CCNB1; CCNB2; NFKB1; RELA; TRAF3IP2; GATA4; SQSTM1; MAPKAPK2; ZFP36L1; ZFP36L2; EIF4EBP1; IL1A; CACNA1D; CAPN1; CAPN2; CALML6; CALML5; CALM1; CALM2; CALM3; CALML3; CALML4; PPP3CA; PPP3CB; PPP3CC; PPP3R1; PPP3R2; NFATC1; NFATC2; NFATC3; NFATC4; ITPR1; ITPR2; ITPR3; MCU; TRPV4; TRPM7; IL6; CXCL8; TGFB1; TGFB2; TGFB3; SMAD2; SMAD3; FOXO1; FBXW11; BTRC; TGFBR1; TGFBR2; IGFBP3; SERPINE1; KIR2DL4; HLA-A; HLA-B; HLA-C; HLA-E; HLA-F; HLA-G; PTEN; CDKN2B;

	SLC25A4; SLC25A5; SLC25A6; SLC25A31; VDAC1; VDAC2; VDAC3; PPID
Cardiac muscle contraction	CACNG3; CACNG2; CACNG5; CACNG4; CACNA2D3; CACNG8; CACNG7; CACNG6; CACNA1C; CACNA1D; CACNA1F; CACNA1S; CACNA2D1; CACNB1; CACNB2; CACNB3; CACNB4; CACNG1; CACNA2D2; CACNA2D4; RYR2; SLC8A2; SLC8A1; SLC8A3; ATP2A1; ATP2A2; ATP2A3; TNNC1; TNNI3; TNNT2; ACTC1; TPM1; TPM2; TPM3; TPM4; MYH6; MYH7; MYL2; MYL3; MYL4; SLC9A6; SLC9A7; UQCR11; COX6B2; COX4I1; COX5B; COX6A1; COX6A2; COX6B1; COX6C; COX7A1; COX7A2; COX7B; COX7C; COX8A; CYC1; COX7B2; UQCRQ; UQCR10; COX8C; UQCRHL; COX1; COX2; COX3; CYTB; UQCRB; UQCRC1; UQCRC2; UQCRFS1; UQCRH; COX4I2; COX7A2L; COX5A; ATP1B4; ATP1A1; ATP1A2; ATP1A3; ATP1A4; ATP1B1; ATP1B2; ATP1B3; FXYD2; SLC9A1; TRDN; ASPH; CASQ2; HRC
Vascular smooth muscle contraction	MYLK4; MYLK; MYLK2; MYLK3; MYL9; MYL6B; MYL6; PPP1R12A; PPP1R12B; PPP1R12C; PPP1CA; PPP1CB; PPP1CC; ROCK1; ROCK2; PRKCA; PRKCB; PRKCD; PRKCE; PRKCG; PRKCH; PRKCQ; RHOA; ARHGEF12; ARHGEF1; ARHGEF11; ACTA2; ACTG2; ADRA1D; ADRA1B; ADRA1A; GNA11; GNAQ; GNA13; GNA12; PLCB1; PLCB2; PLCB3; PLCB4; ITPR1; ITPR2; ITPR3; CALML6; CALML5; CALM1; CALM2; CALM3; CALML3; CALML4; CACNA1C; CACNA1D; CACNA1F; CACNA1S; PPP1R14A; KCNMB2; KCNU1; KCNMB3; KCNMB4; KCNMA1; KCNMB1; CYP4A11; CYP4A22; PLA2G4B; PLA2G4E; PLA2G4F; PLA2G2D; PLA2G4D; PLA2G2E; PLA2G2C; PLA2G3; PLA2G1B; PLA2G2A; PLA2G4A; PLA2G5; PLA2G2F; PLA2G12A; PLA2G6; PLA2G10; PLA2G12B; PLA2G4C; JMJD7-PLA2G4B; CALD1; ARAF; RAF1; BRAF; MAP2K1; MAP2K2; MAPK1; MAPK3; ADORA2A; ADORA2B; NPR1; ADCY1; ADCY2; ADCY3; ADCY5; ADCY6; ADCY7; ADCY8; ADCY9; ADCY4; PRKACA; PRKACB; PRKACG; PRKG1; GUCY1A2; GUCY1A1; GUCY1B1; GNAS; NPR2; AGTR1; EDNRA; AVPR1A; AVPR1B; PTGIR; CALCRL; RAMP2; RAMP1; RAMP3; MYH9; MYH10; MYH11; MYH14; IRAG1; AGT; AVP; NPPA; NPPB; NPPC; EDN1; EDN2; EDN3; CALCA; CALCB; ADM
Wnt signaling pathway	SMAD3; NFATC1; NFATC2; NFATC3; NFATC4; PRKCA; PRKCB; PRKCG; PPP3CA; PPP3CB; PPP3CC; PPP3R1; PPP3R2; CAMK2A; CAMK2B; CAMK2D; CAMK2G; PLCB1; PLCB2; PLCB3; PLCB4; FZD10; FZD2; FZD5; FZD3; FZD1; FZD4; FZD6; FZD7; FZD8; FZD9; WNT5A; WNT5B; MAPK8; MAPK9; MAPK10; RAC1; RAC2; RAC3; ROCK2; RHOA; DAAM1; DAAM2; DVL1; DVL2; DVL3; PRICKLE1; PRICKLE2; PRICKLE4; PRICKLE3; GPC4; VANGL2; VANGL1; WNT11; MMP7; PPARD; CCND1; CCND2; CCND3; FOSL1; JUN; MYC; CTBP1; CTBP2; SOX17; SMAD4; LEF1; TCF7; TCF7L2; TCF7L1; CREBBP; EP300; RUVBL1; CHD8; CTNNBIP1; NLK; MAP3K7; CSNK1A1L; CSNK1A1; RBX1; CUL1; SKP1; FBXW11; BTRC; TBL1X; TBL1XR1; TBL1Y; CACYBP; APC2; APC; SIAH1; NKD1; NKD2; TP53; AXIN1; AXIN2; CTNNB1; PRKACA; PRKACB; PRKACG; PSEN1; GSK3B; FRAT1; FRAT2; SENP2; CSNK2A1; CSNK2A2; CSNK2B; CSNK2A3; CXXC4; TPTEP2-CSNK1E; CSNK1E; DKK1; DKK4; DKK2; WIF1; CER1; LRP6; LRP5; SFRP1; SFRP2; SFRP4; SFRP5; PORCN; WNT16; WNT4; WNT1; WNT2; WNT3; WNT6; WNT7A; WNT7B; WNT8A; WNT8B; WNT10B; WNT2B; WNT9A; WNT9B;

	WNT10A; WNT3A; BAMBI; SOST; SERPINF1; INVS; NOTUM; CCN4; ROR1; ROR2; RYK; RNF43; ZNRF3; LGR4; LGR6; LGR5; RSPO1; RSPO2; RSPO4; RSPO3; CBY1; CTNND2
Axon guidance	PARD6A; PARD6G; PARD6B; PARD3; GNAI1; GNAI2; GNAI3; CXCR4; MAPK1; MAPK3; RAC1; RAC2; RAC3; DCC; NFATC2; NFATC3; NFATC4; PPP3CA; PPP3CB; PPP3CC; PPP3R1; PPP3R2; NTNG1; NTN4; SLIT3; SLIT2; SLIT1; PTK2; CDC42; ROCK1; ROCK2; PAK4; BUB1B-PAK6; PAK1; PAK2; PAK3; PAK6; PAK5; RHOA; ITGB1; RASA1; NCK1; ROBO3; ROBO1; NGEF; ABL1; UNC5D; UNC5B; UNC5C; UNC5A; EPHA2; EPHA1; EPHA3; EPHA4; EPHA5; EPHA7; EPHA8; EPHA6; LRRC4C; ROBO2; NTN3; EFNB1; EFNB2; EFNB3; EPHB1; EPHB2; EPHB3; EPHB4; EPHB6; SEMA7A; EFNA1; EFNA2; EFNA3; EFNA4; EFNA5; FYN; NTN1; RGS3; ARHGEF12; MET; PLXNC1; PLXNB2; PLXNB1; PLXNB3; PLXNA1; PLXNA2; PLXNA3; PLXNA4; SEMA3A; SEMA3C; SEMA3D; SEMA3G; SEMA3F; SEMA3B; SEMA3E; SEMA6C; SEMA6B; SEMA6A; SEMA6D; SEMA4F; SEMA4D; SEMA4B; SEMA4C; SEMA4G; SEMA4A; SEMA5B; SEMA5A; SRGAP2; SRGAP1; SRGAP3; NRP1; L1CAM; CXCL12; NCK2; LRRC4; NTNG2; SRC; HRAS; KRAS; NRAS; PTCH1; SMO; PRKCA; SHH; ILK; BMP7; BMPR1B; BMPR2; GDF7; LIMK1; FZD3; CAMK2A; CAMK2B; CAMK2D; CAMK2G; WNT4; RYK; TRPC1; TRPC3; TRPC4; TRPC5; TRPC6; WNT5A; WNT5B; PIK3CA; PIK3CB; PIK3CD; PIK3R1; PIK3R2; PIK3R3; GSK3B; PDK1; DPYSL2; RHOD; DPYSL5; RND1; LIMK2; CDK5; FES; CFL1; CFL2; RRAS; RAF1; PRKCZ; SSH1; SSH3; SSH2; ENAH; PLCG1; PLCG2; BOC; PTPN11; ABLIM3; ABLIM1; ABLIM2; NEO1; RGMA
VEGF signaling pathway	CDC42; KDR; PLCG1; PLCG2; SPHK2; SPHK1; PTGS2; PLA2G4B; PLA2G4E; PLA2G4F; PLA2G4D; PLA2G4A; PLA2G4C; JMJD7-PLA2G4B; HSPB1; MAPKAPK3; MAPKAPK2; PPP3CA; PPP3CB; PPP3CC; PPP3R1; PPP3R2; AKT3; AKT1; AKT2; PIK3CA; PIK3CB; PIK3CD; PIK3R1; PIK3R2; PIK3R3; NFATC2; PRKCA; PRKCB; PRKCG; PTK2; MAPK14; MAPK11; MAPK13; MAPK12; PXN; SH2D2A; SRC; SHC2; VEGFA; MAPK1; MAPK3; MAP2K1; MAP2K2; RAF1; HRAS; KRAS; NRAS; NOS3; CASP9; BAD
Apelin signaling pathway	APLNR; PRKAG2; PRKAG3; PRKAA1; PRKAA2; PRKAB1; PRKAB2; PRKAG1; SPHK2; SPHK1; SERPINE1; GNB5; GNAI1; GNAI2; GNAI3; GNB1; GNB2; GNB3; GNG3; GNG4; GNG5; GNG7; GNG10; GNG11; GNGT1; GNGT2; GNG13; GNG2; GNG12; GNB4; GNG8; PIK3R6; PIK3R5; PIK3CG; AKT3; AKT1; AKT2; MAP2K1; MAP2K2; MAPK1; MAPK3; RPS6KB1; RPS6KB2; PLCB1; PLCB2; PLCB3; PLCB4; ITPR1; ITPR2; ITPR3; RYR1; RYR2; RYR3; MTOR; RRAS2; MRAS; HRAS; KRAS; NRAS; RRAS; RAF1; KLF2; TGFBR1; CCN2; PPARGC1A; TFAM; NRF1; MYLK4; MYLK; MYLK2; MYLK3; MYL2; MYL3; MYL4; PRKCE; SLC8A2; SLC8A1; SLC8A3; CCND1; ADCY1; ADCY2; ADCY3; ADCY5; ADCY6; ADCY7; ADCY8; ADCY9; ADCY4; ADCY10; PRKACA; PRKACB; PRKACG; UCP1; PDE3B; LIPE; NOS1; NOS2; NOS3; MEF2B; MEF2A; BORCS8-MEF2B; MEF2C; MEF2D; HDAC5; HDAC4; GNA13; PIK3R4; PIK3C3; GABARAP; GABARAPL2; GABARAPL1; BECN2; BECN1; EGR1; SPP1; NOTCH3; JAG1; PLAT; SMAD4; SMAD2; SMAD3; ACTA2; CDH1; RPS6; PLIN1; SLC9A1; GNAQ; CAMK4; CALML6; CALML5; CALM1; CALM2; CALM3; CALML3; CALML4; APLN; AGTR1

Osteoclast differentiation	CSF1R; TRAF2; TRAF6; TNFRSF11A; TNFSF11; CSF1; TNFRSF11B; CYLD; GAB2; MAP3K14; CHUK; IKBKG; IKBKB; NFKBIA; NFKB1; RELA; MAP3K7; TAB1; TAB2; MAP2K6; MAP2K1; MAPK8; MAPK9; MAPK10; MAPK14; MAPK11; MAPK13; MAPK12; NFATC1; IFNB1; IFNAR1; IFNAR2; JAK1; TYK2; STAT1; STAT2; IRF9; FYN; LCK; PIK3CA; PIK3CB; PIK3CD; PIK3R1; PIK3R2; PIK3R3; AKT3; AKT1; AKT2; IL1A; IL1B; IL1R1; IFNG; IFNGR1; IFNGR2; FOS; FOSB; FOSL2; JUN; JUNB; JUND; FOSL1; FCGR1A; FCGR2A; FCGR2B; FCGR3A; FCGR3B; FCGR2C; TYROBP; BTK; TEC; SYK; BLNK; LCP2; PLCG2; PPP3CA; PPP3CB; PPP3CC; PPP3R1; PPP3R2; CAMK4; CREB1; CTSK; ACP5; CALCR; ITGB3; SPI1; MITF; TNF; TNFRSF1A; NFATC2; SOCS1; SOCS3; FHL2; PPARG; TGFB1; TGFB2; TGFBR1; TGFBR2; NFKB2; RELB; SQSTM1; GRB2; MAPK1; MAPK3; MAP2K7; RAC1; CYBA; NOX1; NCF2; NCF4; NCF1; LOC102725035; LILRB2; LILRB1; LILRB5; LILRB4; LILRA1; LILRB3; LILRA3; LILRA2; OSCAR; LILRA4; LILRA5; LILRA6
Hippo signaling pathway	WWC1; NF2; FRMD6; FRMD1; SAV1; STK3; YAP1; WWTR1; LATS2; LATS1; CRB1; CRB2; CTNNA1; CTNNA2; CTNNA3; RASSF1; CCN2; BIRC5; AREG; PPP1CA; PPP1CB; PPP1CC; TP53BP2; YWHAQ; YWHAB; YWHAE; YWHAG; YWHAH; YWHAZ; MOB1A; MOB1B; AMOT; MPP5; PATJ; CDH1; LLGL2; LLGL1; SCRIB; DLG1; DLG2; DLG3; DLG4; DLG5; TEAD1; TEAD4; TEAD3; TEAD2; WTIP; AJUBA; LIMD1; ACTB; ACTG1; PARD3; PARD6A; PARD6G; PARD6B; PRKCI; PRKCZ; TP73; SMAD2; SMAD3; TPTEP2-CSNK1E; CSNK1D; CSNK1E; FBXW11; BTRC; ITGB2; TGFB1; TGFB2; TGFB3; TGFBR1; TGFBR2; SMAD4; SMAD7; SERPINE1; WNT16; WNT4; WNT1; WNT2; WNT3; WNT5A; WNT6; WNT7A; WNT7B; WNT8A; WNT8B; WNT10B; WNT11; WNT2B; WNT9A; WNT9B; WNT10A; WNT5B; WNT3A; FZD10; FZD2; FZD5; FZD3; FZD1; FZD4; FZD6; FZD7; FZD8; FZD9; DVL1; DVL2; DVL3; GSK3B; CTNNB1; APC2; APC; AXIN1; AXIN2; LEF1; TCF7; TCF7L2; TCF7L1; NKD1; NKD2; MYC; CCND1; CCND2; CCND3; SOX2; SNAI2; BIRC2; BIRC3; GDF7; AMH; BMP8A; GDF6; BMP2; BMP4; BMP5; BMP6; BMP7; BMP8B; GDF5; BMPR1A; BMPR1B; BMPR2; SMAD1; ID1; ID2; BBC3; PPP2CA; PPP2CB; PPP2R1A; PPP2R1B; PPP2R2A; PPP2R2B; PPP2R2C; PPP2R2D; RASSF6; GLI2; AFP; FGF1
Hippo signaling pathway - multiple species	WWC1; NF2; FRMD6; FRMD1; SAV1; STK3; YAP1; WWTR1; LATS2; LATS1; MOB1A; MOB1B; TEAD1; TEAD4; TEAD3; TEAD2; PAK1; DCHS2; DCHS1; FAT4; TPTEP2-CSNK1E; CSNK1E; RASSF4; RASSF2; RASSF6; RASSF1; WTIP; AJUBA; LIMD1
Focal adhesion	ITGB1; ITGB3; ITGB4; ITGB5; ITGB6; ITGB7; ITGB8; ITGA11; ITGA6; ITGA1; ITGA2; ITGA2B; ITGA3; ITGA4; ITGA5; ITGA7; ITGA9; ITGAV; ITGA10; ITGA8; FLNA; FLNB; FLNC; CDC42; MYLK4; MYLK; MYLK2; MYLK3; MYL12B; MYL9; MYL12A; MYLPF; MYL2; MYL5; MYL7; MYL10; DIAPH1; PPP1R12A; PPP1R12B; PPP1R12C; PPP1CA; PPP1CB; PPP1CC; ROCK1; ROCK2; AKT3; AKT1; AKT2; PDPK1; PARVB; PARVA; PARVG; ILK; VCL; VASP; PTEN; PIK3CA; PIK3CB; PIK3CD; PIK3R1; PIK3R2; PIK3R3; TLN1; TLN2; ZYX; PIP5K1C; PIP5K1A; PIP5K1B; ACTN4; ACTN1; PTK2; CAPN2; PXN; ARHGAP35; ARHGAP5; PRKCA; PRKCB; PRKCG; SRC; FYN; RHOA; RASGRF1; LAMC3; CHAD; COL1A1;

	COL1A2; COL2A1; COL4A1; COL4A2; COL4A3; COL4A4; COL4A5; COL4A6; COL6A1; COL6A2; COL6A3; COL9A1; COL9A2; COL9A3; COMP; COL6A6; LAMB4; FN1; COL6A5; LAMA1; TNC; IBSP; LAMA2; LAMA3; LAMA4; LAMA5; LAMB1; LAMB2; LAMB3; LAMC1; LAMC2; RELN; TNN; SPP1; THBS1; THBS2; THBS3; THBS4; TNR; TNXB; VTN; VWF; CAV1; CAV2; CAV3; BIRC2; BIRC3; XIAP; BAD; BCL2; ELK1; MAPK1; MAPK3; CCND1; CCND2; CCND3; MAP2K1; BRAF; RAF1; HRAS; SOS1; SOS2; GRB2; EGFR; ERBB2; FLT1; FLT4; IGF1R; KDR; MET; PDGFRA; PDGFRB; EGF; VEGFD; HGF; IGF1; PDGFA; PDGFB; PGF; PDGFC; VEGFA; VEGFB; VEGFC; PDGFD; SHC2; SHC4; SHC3; SHC1; BCAR1; CRK; CRKL; RAPGEF1; RAP1A; RAP1B; DOCK1; MAPK8; MAPK9; MAPK10; JUN; PAK4; BUB1B-PAK6; PAK1; PAK2; PAK3; PAK6; PAK5; RAC1; RAC2; RAC3; VAV3; VAV1; VAV2; CTNNB1; GSK3B
ECM-receptor interaction	LAMC3; LAMB4; LAMA1; LAMA2; LAMA3; LAMA4; LAMA5; LAMB1; LAMB2; LAMB3; LAMC1; LAMC2; COL1A1; COL1A2; COL2A1; COL4A1; COL4A2; COL4A3; COL4A4; COL4A5; COL4A6; COL6A1; COL6A2; COL6A3; COL9A1; COL9A2; COL9A3; COL6A6; COL6A5; DAG1; TNC; TNN; TNR; TNXB; GP6; GP9; GP1BB; GP1BA; GP5; VWF; IBSP; VTN; SPP1; FN1; COMP; THBS1; THBS2; THBS3; THBS4; ITGB3; ITGB1; ITGA6; ITGAV; ITGA4; HMMR; CD47; CD36; SV2C; SV2B; SV2A; SDC1; SDC4; HSPG2; ITGB4; ITGB8; ITGB6; ITGB5; ITGA2B; ITGB7; ITGA11; ITGA10; ITGA9; ITGA8; ITGA7; ITGA5; ITGA3; ITGA1; NPNT; CHAD; RELN; CD44; ITGA2; AGRN; FREM1; FREM2; FRAS1; DSPP; DMP1
Cell adhesion molecules	ALCAM; CD6; SPN; SIGLEC1; PTPRC; CD22; CD8A; CD8B; CD8B2; ITGB1; ITGA8; ITGB2; ITGAL; CD40; NEO1; CDH15; CDH4; CDH3; ITGA6; NECTIN2; CDH1; VCAN; MAG; MPZ; MPZL1; NECTIN3; NFASC; CNTNAP2; CNTNAP1; PTPRM; CNTN2; CNTN1; NRCAM; NLGN4Y; NLGN1; NLGN3; NLGN4X; NLGN2; NRXN3; NRXN1; NRXN2; ITGB8; ITGAV; SDC1; SDC2; SDC4; SDC3; PTPRF; NEGR1; CADM3; CADM1; CDH2; NCAM1; NCAM2; L1CAM; NECTIN1; PVR; CD226; SELE; GLG1; SELP; SELPLG; CD34; SELL; MADCAM1; VCAM1; ITGB7; ITGA9; ITGA4; CD99; CD99L2; PECAM1; CD58; CD2; ICAM3; ICAM1; ICAM2; JAM2; JAM3; ITGAM; F11R; CD40LG; CDH5; ESAM; PDCD1LG2; CD274; PDCD1; CD4; HLA-DMA; HLA-DMB; HLA-DOA; HLA-DOB; HLA-DPA1; HLA-DPB1; HLA-DQA1; HLA-DQA2; HLA-DQB1; HLA-DRA; HLA-DRB1; HLA-DRB3; HLA-DRB4; HLA-DRB5; CD86; CD80; CD28; HLA-A; HLA-B; HLA-C; HLA-E; HLA-F; HLA-G; OCLN; CLDN24; CLDN34; CLDN16; CLDN4; CLDN3; CLDN7; CLDN23; CLDN19; CLDN14; CLDN15; CLDN17; CLDN20; CLDN11; CLDN18; CLDN22; CLDN25; CLDN5; CLDN10; CLDN8; CLDN6; CLDN2; CLDN1; CLDN9; ICOS; CD276; LOC102723996; ICOSLG; CTLA4; VTCN1; TIGIT; NTNG1; NTNG2; LRRC4C; LRRC4; LRRC4B; VSIR; IGSF11
Adherens junction	SMAD4; CDH1; CTNNA1; CTNNA2; CTNNA3; CTNND1; LEF1; TCF7; TCF7L2; TCF7L1; NLK; MAP3K7; CREBBP; EP300; SMAD3; YES1; FYN; FER; SNAI1; SNAI2; MAPK1; MAPK3; TGFBR1; TGFBR2; FGFR1; EGFR; ERBB2; MET; INSR; IGF1R; CSNK2A1; CSNK2A2; CSNK2B; CSNK2A3; PTPRJ; PTPN6; PTPN1; PTPRF; PTPRB; PTPRM; ACP1; RHOA; WASF2; WASF3; WASF1; BAIAP2; WAS; WASL; RAC1; RAC2; RAC3; CDC42; ACTB; ACTG1; TJP1; VCL; SORBS1; ACTN4; ACTN1;

	SSX2IP; LMO7; AFDN; CTNNB1; IQGAP1; SRC; NECTIN3; NECTIN1; NECTIN2; NECTIN4; PARD3; FARP2
Tight junction	PARD6A; PARD6G; PARD6B; PATJ; CDC42; STK11; CGN; TIAM1; ARHGAP17; OCLN; TJP1; ARHGEF18; RHOA; ROCK1; ROCK2; MYL2; EPB41L4B; PARD3; PRKCI; PRKCZ; RAC1; IGSF5; MAGI1; F11R; RAPGEF6; RAP1A; PPP2CA; PPP2CB; PPP2R1A; PPP2R1B; PPP2R2A; PPP2R2B; PPP2R2C; PPP2R2D; ACTN4; ACTN1; ACTB; ACTG1; SYNPO; MICALL2; RAB13; PRKACA; PRKACB; PRKACG; VASP; SRC; MAP2K7; MAP3K5; MAPK8; MAPK9; MAPK10; CACNA1D; YBX3; SLC9A3R1; MSN; RDX; EZR; LLGL2; LLGL1; MPDZ; MPP4; MARVELD3; MARVELD2; MAP3K1; CLDN24; CLDN34; CLDN16; CLDN4; CLDN3; CLDN7; CLDN23; CLDN19; CLDN14; CLDN15; CLDN17; CLDN20; CLDN11; CLDN18; CLDN22; CLDN25; CLDN5; CLDN10; CLDN8; CLDN6; CLDN2; CLDN1; CLDN9; PRKAG2; PRKAG3; PRKAA1; PRKAA2; PRKAB1; PRKAB2; PRKAG1; TJP2; AFDN; RAPGEF2; RAP2C; TUBA1B; TUBA3E; TUBA3D; TUBA8; TUBA4A; TUBA3C; TUBA1A; TUBAL3; TUBA1C; WHAMM; WAS; ACTR3; ACTR2; ACTR3B; ACTR3C; NF2; CDK4; ERBB2; PCNA; RUNX1; ARHGEF2; JAM2; JAM3; AMOT; AMOTL1; AMOTL2; JUN; CD1A; CD1B; CD1C; CD1D; CD1E; HSPA4; GATA4; CCND1; SYMPK; CFTR; BVES; CTTN; HCLS1; RAB8A; RAB8B; ITGB1; CRB3; MPP5; SCRIB; DLG1; TJP3; MYL12B; MYL9; MYL12A; MYL6B; MYH9; MYH10; MYH11; MYL6; MYH14; PRKCE; CGNL1; TJAP1; DLG3; DLG2
Signaling pathways regulating pluripotency of stem cells	WNT16; WNT4; WNT1; WNT2; WNT3; WNT5A; WNT6; WNT7A; WNT7B; WNT8A; WNT8B; WNT10B; WNT11; WNT2B; WNT9A; WNT9B; WNT10A; WNT5B; WNT3A; APC2; APC; AXIN1; AXIN2; DVL1; DVL2; DVL3; FZD10; FZD2; FZD5; FZD3; FZD1; FZD4; FZD6; FZD7; FZD8; FZD9; CTNNB1; GSK3B; TCF7; LIF; IL6ST; LIFR; JAK1; JAK2; JAK3; STAT3; KLF4; PIK3CA; PIK3CB; PIK3CD; PIK3R1; PIK3R2; PIK3R3; AKT3; AKT1; AKT2; TBX3; GRB2; INHBA; INHBB; INHBC; INHBE; ACVR1C; ACVR1B; ACVR2A; ACVR2B; SMAD2; SMAD3; BMP4; BMPR1A; BMPR1B; BMPR2; ACVR1; MAPK1; MAPK3; NANOGP8; NANOG; POU5F1; POU5F1B; SOX2; HRAS; KRAS; NRAS; RAF1; MAP2K1; MAP2K2; FGF2; FGFR1; FGFR3; FGFR2; FGFR4; IGF1; IGF1R; SMAD1; SMAD5; SMAD9; MAPK14; MAPK11; MAPK13; MAPK12; SMAD4; MYC; ID1; ID2; ID3; ID4; HESX1; ZIC3; ESRRB; TCF3; LEFTY1; LEFTY2; SKIL; SMARCAD1; KAT6A; SETDB1; JARID2; REST; RIF1; PAX6; MEIS1; HOXA1; HOXB1; HOXD1; LHX5; OTX1; NEUROG1; HAND1; DLX5; MYF5; ONECUT1; ISL1; ZFHX3; ESX1; DUSP9; COMMD3-BMI1; PCGF3; BMI1; PCGF2; PCGF6; PCGF5; PCGF1; NODAL
Complement and coagulation cascades	F12; F11; F9; F10; F7; FGA; FGB; FGG; F13A1; F13B; TFPI; F2R; SERPIND1; SERPINC1; VWF; SERPINA5; SERPINE1; SERPINB2; PLG; KNG1; KLKB1; PLAU; PLAT; BDKRB1; BDKRB2; MBL2; MASP2; MASP1; C1QA; C1QB; C1QC; C1R; C1S; C3; SERPING1; CFH; C5; C6; C7; C8A; C8B; C8G; C9; CD59; CD46; CD55; C4BPA; C4BPB; PROS1; PROC; THBD; SERPINA1; F2; A2M; SERPINF2; C4A; C4B; C2; CFI; F5; F8; CPB2; CLU; VTN; CR1; CR1L; PLAUR; F2RL2; F2RL3; PROCR; F3; CR2; ITGAM; ITGB2; ITGAX; VSIG4; C5AR1; C3AR1; CFB; CFD; CFHR3; CFHR1; CFHR2; CFHR5; CFHR4
Platelet activation	ITPR1; ITPR2; ITPR3; F2R; F2RL3; P2RY1; TBXA2R; PLCB1; PLCB2; PLCB3;

	PLCB4; GP6; ITGA2; ITGB1; GP5; PLCG2; RASGRP1; RASGRP2; RAP1A; RAP1B; APBB1IP; TLN1; TLN2; ITGA2B; ITGB3; ORAI1; PIK3CA; PIK3CB; PIK3CD; PIK3R1; PIK3R2; PIK3R3; AKT3; AKT1; AKT2; NOS3; GUCY1A2; GUCY1A1; GUCY1B1; PRKG1; PRKG2; MAPK14; MAPK11; MAPK13; MAPK12; MAPK1; MAPK3; GNAQ; GNA13; ARHGEF12; ARHGEF1; RHOA; ROCK1; ROCK2; PPP1R12A; PPP1CA; PPP1CB; PPP1CC; MYL12B; MYL12A; MYLK4; MYLK; MYLK2; MYLK3; P2RY12; GNAI1; GNAI2; GNAI3; ADCY1; ADCY2; ADCY3; ADCY5; ADCY6; ADCY7; ADCY8; ADCY9; ADCY4; PIK3R6; PIK3R5; PIK3CG; PTGIR; GNAS; PLA2G4B; PLA2G4E; PLA2G4F; PLA2G4D; PLA2G4A; PLA2G4C; JMJD7-PLA2G4B; STIM1; VWF; PTGS1; TBXAS1; PRKACA; PRKACB; PRKACG; VASP; ACTB; ACTG1; COL1A1; COL1A2; COL3A1; FGA; FGB; FGG; FERMT3; P2RX1; GP1BA; GP1BB; GP9; FCER1G; LYN; FYN; LCP2; BTK; SYK; PRKCI; PRKCZ; SNAP23; VAMP8; SRC; FCGR2A; ARHGAP35; F2
NOD-like receptor signaling pathway	PYCARD; CASP1; NOD1; NOD2; RIPK2; CHUK; IKBKB; MAP3K7; TAB1; TAB2; TAB3; NLRP1; NLRP3; IL1B; IL18; CARD8; NLRC4; SUGT1; HSP90AA1; HSP90AB1; MAPK8; MAPK9; MAPK10; MAPK1; MAPK3; MAPK14; MAPK11; MAPK13; MAPK12; PYDC2; MEFV; PSTPIP1; NAIP; AIM2; TP53BP1; P2RX7; TRPV2; TRPM7; TRPM2; PANX1; NFKB1; RELA; JUN; IKBKG; TRAF3; TBK1; IKBKE; IRF3; IRF7; IRAK4; NLRP12; GPRC6A; CASR; ITPR1; ITPR2; ITPR3; PLCB1; PLCB2; PLCB3; PLCB4; JAK1; TYK2; STAT1; STAT2; CASP4; GSDMD; TLR4; MYD88; IFNAR1; IFNAR2; ANTXR2; ANTXR1; GBP5; GBP2; RHOA; IFNA1; IFNA2; IFNA4; IFNA5; IFNA6; IFNA7; IFNA8; IFNA10; IFNA13; IFNA14; IFNA16; IFNA17; IFNA21; IFNB1; MAVS; VDAC1; VDAC2; VDAC3; MCU; ERBIN; TICAM1; NEK7; GBP4; GBP1; GBP3; GBP7; BRCC3; CASP12; TRAF6; NFKBIA; NFKBIB; PYDC5; PYDC1; TXNIP; BIRC2; BIRC3; XIAP; TXN2; TXN; RBCK1; RNF31; SHARPIN; ATG16L1; GABARAP; GABARAPL2; GABARAPL1; ATG12; ATG5; TANK; CYBB; CYBA; NAMPT; IFI16; STING1; NLRP6; NLRP7; TNFAIP3; CARD6; TRIP6; TRAF2; TRAF5; NLRX1; CARD9; IRF9; CARD16; CARD17; CARD18; PRKCD; CASP5; CASP8; FADD; BCL2; BCL2L1; MFN1; MFN2; DHX33; RIPK3; RIPK1; DNM1L; CTSB; OAS1; OAS2; OAS3; RNASEL; IL6; TNF; CXCL8; CXCL1; CXCL2; CXCL3; CCL2; CCL5; CAMP; DEFB4B; DEFA1; DEFA3; DEFA4; DEFA5; DEFA6; DEFB4A; DEFB103A; DEFB103B; DEFA1B; PKN1; PKN2; YWHAE
RIG-I-like receptor signaling pathway	DDX58; IFIH1; TRIM25; RNF125; MAVS; NFKB1; RELA; OTUD5; TRAF3; IRF3; IKBKE; IRF7; TANK; AZI2; TBKBP1; FADD; CASP8; CASP10; IFNA1; IFNA2; IFNA4; IFNA5; IFNA6; IFNA7; IFNA8; IFNA10; IFNA13; IFNA14; IFNA16; IFNA17; IFNA21; IFNB1; TRAF6; CHUK; IKBKB; IKBKG; NFKBIA; NFKBIB; TBK1; DHX58; ISG15; ATG5; ATG12; NLRX1; STING1; CYLD; PIN1; TRADD; RIPK1; SIKE1; TKFC; DDX3X; MAP3K1; MAPK8; MAPK9; MAPK10; MAPK14; MAPK11; MAPK13; MAPK12; TRAF2; MAP3K7; IFNW1; IFNE; IFNK; CXCL8; TNF; IL12A; IL12B; CXCL10
Cytosolic DNA-sensing pathway	CASP1; PYCARD; AIM2; IL1B; IL18; ZBP1; TBK1; IKBKE; IRF3; IFNB1; IRF7; IFNA1; IFNA2; IFNA4; IFNA5; IFNA6; IFNA7; IFNA8; IFNA10; IFNA13; IFNA14; IFNA16; IFNA17; IFNA21; CXCL10; MAVS; STING1; NFKB1; RELA; RIPK1; RIPK3; ADAR; IL6; CHUK; IKBKB; IKBKG; DDX58; IL33; NFKBIA; NFKBIB; POLR3F;

	POLR3G; POLR3C; POLR3A; POLR3H; POLR1D; POLR3K; POLR2E; POLR2F; POLR2H; POLR2K; POLR2L; POLR3B; POLR3E; POLR3D; POLR3GL; POLR1C; CCL4L1; CCL4; CCL4L2; CCL5; TREX1; CGAS
C-type lectin receptor signaling pathway	CLEC7A; CLEC6A; CLEC4E; CLEC4D; FCER1G; CLEC4M; CD209; LSP1; KSR1; PLK3; SRC; NFKB1; RAF1; PAK1; RRAS2; MRAS; HRAS; KRAS; NRAS; RRAS; RELA; SYK; IL23A; PTPN11; IKBKG; CHUK; IKBKB; IL12A; IL12B; NLRP3; CASP1; PYCARD; IL1B; CASP8; IL6; ARHGEF12; RHOA; CYLD; IKBKE; STAT1; STAT2; IRF9; IL17D; BCL3; MAPKAPK2; IL10; NFKBIA; CARD9; BCL10; MALT1; PRKCD; PLCG2; MAPK1; MAPK3; NFATC1; NFATC2; NFATC3; NFATC4; IL2; PPP3CA; PPP3CB; PPP3CC; PPP3R1; PPP3R2; ITPR1; ITPR2; ITPR3; MAP3K14; TNF; CBLB; CLEC1B; PIK3CA; PIK3CB; PIK3CD; PIK3R1; PIK3R2; PIK3R3; AKT3; AKT1; AKT2; MDM2; IRF1; NFKB2; RELB; CALML6; CALML5; CALM1; CALM2; CALM3; CALML3; CALML4; PTGS2; CCL22; CCL17; EGR2; EGR3; MAPK14; MAPK11; MAPK13; MAPK12; MAPK8; MAPK9; MAPK10; JUN
Hematopoietic cell lineage	LOC102723407; ITGA6; ITGA1; ITGA2; ITGA3; ITGA4; ITGA5; GP1BA; GP1BB; GP5; GP9; ITGA2B; CD14; CD9; ITGB3; IL11RA; CD59; CD55; CR1; CR1L; CD44; GYPA; CD36; KIT; EPOR; IL5RA; CSF3R; IL1R1; IL1R2; IL9R; ITGAM; ANPEP; IL6R; CSF1R; FCGR1A; IL4R; CD33; CSF2RA; IL3RA; FCER2; CD37; CR2; MS4A1; CD24; CD22; CD19; MME; CD3D; CD3E; CD3G; CD8A; CD8B; CD8B2; CD4; CD1A; CD1B; CD1C; CD1D; CD1E; CD5; CD2; CD7; CD34; CD38; TFRC; IL7R; IL2RA; DNTT; FLT3; HLA-DMA; HLA-DMB; HLA-DOA; HLA-DOB; HLA-DPA1; HLA-DPB1; HLA-DQA1; HLA-DQA2; HLA-DQB1; HLA-DRA; HLA-DRB1; HLA-DRB3; HLA-DRB4; HLA-DRB5; THPO; EPO; TNF; CSF1; CSF3; FLT3LG; IL11; IL6; CSF2; IL4; IL1A; IL1B; IL5; IL3; IL7; KITLG
Natural killer cell mediated cytotoxicity	LOC102723407; NFATC1; NFATC2; PPP3CA; PPP3CB; PPP3CC; PPP3R1; PPP3R2; FYN; TNFSF10; IFNGR1; IFNGR2; PLCG1; PLCG2; LAT; PRF1; HLA-E; IFNG; CSF2; TNF; PRKCA; PRKCB; PRKCG; SH3BP2; PTK2B; LCP2; SYK; ZAP70; LCK; SH2D1B; SH2D1A; HCST; CD247; FCER1G; TYROBP; PTPN11; PTPN6; IFNA1; IFNA2; IFNA4; IFNA5; IFNA6; IFNA7; IFNA8; IFNA10; IFNA13; IFNA14; IFNA16; IFNA17; IFNA21; IFNB1; BID; CASP3; GZMB; FAS; TNFRSF10B; TNFRSF10A; CD48; ULBP3; ULBP2; ULBP1; MICA; MICB; ICAM1; ICAM2; HLA-C; HLA-B; HLA-G; HLA-A; PIK3CA; PIK3CB; PIK3CD; PIK3R1; PIK3R2; PIK3R3; MAPK1; MAPK3; MAP2K1; MAP2K2; ARAF; RAF1; BRAF; HRAS; KRAS; NRAS; SOS1; SOS2; GRB2; SHC2; SHC4; SHC3; SHC1; PAK1; RAC1; RAC2; RAC3; VAV3; VAV1; VAV2; FASLG; KLRD1; IFNAR1; IFNAR2; CD244; KLRC4-KLRK1; KLRK1; NCR3; NCR1; FCGR3A; FCGR3B; NCR2; KLRC2; KLRC3; KIR2DS2; KIR2DS1; KIR2DS3; KIR2DS4; KIR2DS5; ITGB2; ITGAL; KLRC1; KIR2DL1; KIR2DL2; KIR2DL3; KIR2DL5A; KIR3DL3; KIR3DL1; KIR3DL2; RAET1E; RAET1L; RAET1G
T cell receptor signaling pathway	CDK4; TNF; CSF2; IFNG; IL10; IL5; IL4; IL2; NFKBIA; NFKBIB; NFKBIE; NFKB1; RELA; IKBKB; IKBKG; CHUK; MAP3K14; MAP3K8; AKT3; AKT1; AKT2; MALT1; BCL10; CARD11; PRKCQ; PDPK1; GRB2; PIK3CA; PIK3CB; PIK3CD; PIK3R1; PIK3R2; PIK3R3; CD28; ICOS; CD40LG; SOS1; SOS2; RASGRP1; HRAS; KRAS; NRAS; FOS; JUN; NFATC1; NFATC2; NFATC3; PPP3CA; PPP3CB; PPP3CC; PPP3R1; PPP3R2; ITK; TEC; PLCG1; LAT; GRAP2; VAV3; VAV1; VAV2; RHOA; CDC42;

	PAK4; BUB1B-PAK6; PAK1; PAK2; PAK3; PAK6; PAK5; NCK1; NCK2; LCP2; ZAP70; CD3E; CD3D; CD247; FYN; CD3G; CD4; CD8A; CD8B; CD8B2; CBLB; LCK; PTPRC; PTPN6; CTLA4; PDCD1; MAP2K1; MAP2K2; RAF1; MAPK1; MAPK3; MAP3K7; MAP2K7; MAPK8; MAPK9; MAPK10; DLG1; MAPK14; MAPK11; MAPK13; MAPK12; GSK3B
B cell receptor signaling pathway	NFKBIA; NFKBIB; NFKBIE; NFKB1; RELA; IKBKB; IKBKG; CHUK; GSK3B; MALT1; BCL10; CARD11; AKT3; AKT1; AKT2; PRKCB; VAV3; VAV1; VAV2; PIK3CA; PIK3CB; PIK3CD; PIK3R1; PIK3R2; PIK3R3; CR2; CD19; CD81; IFITM1; INPP5D; INPPL1; FCGR2B; FOS; JUN; HRAS; KRAS; NRAS; RASGRP3; NFATC1; NFATC2; NFATC3; PPP3CA; PPP3CB; PPP3CC; PPP3R1; PPP3R2; RAC1; RAC2; RAC3; PLCG2; BLNK; BTK; LYN; CD79B; CD79A; LOC102723407; SYK; PTPN6; CD72; CD22; LOC102725035; LILRB2; LILRB1; LILRB5; LILRB4; LILRA1; LILRB3; LILRA3; LILRA2; LILRA4; LILRA5; LILRA6; MAP2K1; MAP2K2; MAPK1; MAPK3; RAF1; SOS1; SOS2; GRB2; PIK3AP1; DAPP1
Fc epsilon RI signaling pathway	LOC102723407; FCER1G; MS4A2; FCER1A; FYN; TNF; IL13; IL4; AKT3; AKT1; AKT2; CSF2; MAPK14; MAPK11; MAPK13; MAPK12; MAP2K7; MAP2K4; MAP2K3; MAP2K6; PDPK1; PIK3CA; PIK3CB; PIK3CD; PIK3R1; PIK3R2; PIK3R3; PRKCA; GAB2; LCP2; BTK; SYK; LYN; LAT; PLCG1; PLCG2; INPP5D; MAPK1; MAPK3; MAP2K1; MAP2K2; RAF1; HRAS; KRAS; NRAS; SOS1; SOS2; GRB2; PLA2G4B; PLA2G4E; PLA2G4F; PLA2G4D; PLA2G4A; PLA2G4C; JMJD7-PLA2G4B; MAPK8; MAPK9; MAPK10; RAC1; RAC2; RAC3; VAV3; VAV1; VAV2; IL5; IL3; ALOX5; ALOX5AP
Fc gamma R-mediated phagocytosis	FCGR3A; FCGR3B; FCGR2B; FCGR2A; FCGR1A; HCK; LYN; SYK; PTPRC; LOC102723407; PIK3CA; PIK3CB; PIK3CD; PIK3R1; PIK3R2; PIK3R3; INPP5D; INPPL1; LAT; PLCG1; PLCG2; PRKCD; PRKCE; RAF1; MAP2K1; MAPK1; MAPK3; PLA2G4B; PLA2G4E; PLA2G4F; PLA2G4D; PLA2G4A; PLA2G4C; JMJD7-PLA2G4B; AKT3; AKT1; AKT2; RPS6KB1; RPS6KB2; VAV3; VAV1; VAV2; RAC1; RAC2; WASF2; WASF3; WASF1; ARPC5; ARPC4; ARPC3; ARPC1B; ARPC2; ARPC1A; ARPC5L; CDC42; WAS; DOCK1; CRK; CRKL; PLD1; PLD2; PAK1; VASP; LIMK1; LIMK2; CFL1; CFL2; ASAP1; ASAP3; ASAP2; PLA2G6; MARCKS; MARCKSL1; MYO10; GSN; SCIN; PIP5K1C; PIP5K1A; PIP5K1B; PLPP1; PLPP2; PLPP3; GAB2; ARF6; DNM2; NCF1; AMPH; BIN1; SPHK2; SPHK1
TNF signaling pathway	TNF; TNFRSF1A; TRADD; TRAF2; TRAF5; RIPK1; BIRC2; BIRC3; MAP3K7; MAP2K7; MAP2K4; MAP2K3; MAP2K6; MAPK14; MAPK11; MAPK13; MAPK12; MAPK8; MAPK9; MAPK10; IKBKG; IKBKB; NFKBIA; NFKB1; RELA; MAP3K8; MAP2K1; MAPK1; MAPK3; RPS6KA4; RPS6KA5; CREB3; CREB1; ATF2; ATF6B; CREB3L4; ATF4; CREB3L2; CREB3L3; CREB3L1; CREB5; CEBPB; FOS; JUN; FADD; CASP8; CASP7; CASP3; CCL2; CCL5; CCL20; CXCL1; CXCL2; CXCL3; CXCL6; CXCL5; CXCL10; CX3CL1; CSF1; CSF2; FAS; IL18R1; JAG1; IL1B; IL6; IL15; LIF; LTA; BCL3; SOCS3; TNFAIP3; TRAF1; JUNB; MMP3; MMP9; MMP14; EDN1; VEGFD; VEGFC; NOD2; ICAM1; SELE; VCAM1; PTGS2; TNFRSF1B; PIK3CA; PIK3CB; PIK3CD; PIK3R1; PIK3R2; PIK3R3; AKT3; AKT1; AKT2; MAP3K14; CHUK; TRAF3; RIPK3; BAG4; CASP10; MLKL; PGAM5; DNM1L; TAB1; TAB2; TAB3; MAP3K5; ITCH; CFLAR; DAB2IP; IRF1; IFNB1

Leukocyte transendothelial migration	CLDN24; CLDN34; OCLN; CLDN16; CLDN4; CLDN3; CLDN7; CLDN23; CLDN19; CLDN14; CLDN15; CLDN17; CLDN20; CLDN11; CLDN18; CLDN22; CLDN25; CLDN5; CLDN10; CLDN8; CLDN6; CLDN2; CLDN1; CLDN9; ESAM; MMP2; MMP9; PTPN11; CDH5; CD99; CD99L2; PECAM1; ITGB2; CTNND1; BCAR1; AFDN; SIPA1; F11R; ITGAL; ITGB1; ITGA4; JAM2; ITGAM; PIK3CA; PIK3CB; PIK3CD; PIK3R1; PIK3R2; PIK3R3; CTNNB1; ACTB; ACTG1; MYL12B; MYL9; MYL12A; MYLPF; MYL2; MYL5; MYL7; MYL10; ROCK1; ROCK2; PRKCA; PRKCB; PRKCG; PTK2; RHOA; JAM3; MAPK14; MAPK11; MAPK13; MAPK12; ARHGAP35; ARHGAP5; CTNNA1; CTNNA2; CTNNA3; CYBB; CYBA; NCF2; PXN; NCF1; RAC1; NCF4; RAP1A; RAP1B; VAV3; VAV1; VAV2; RAC2; MSN; EZR; RAPGEF3; RAPGEF4; ITK; TXK; RASSF5; PTK2B; CXCL12; CXCR4; GNAI1; GNAI2; GNAI3; THY1; VCAM1; ICAM1; PLCG1; PLCG2; CDC42; RHOH; VCL; VASP; ACTN4; ACTN1
Intestinal immune network for IgA production	TGFB1; IL5; IL6; TNFSF13; TNFSF13B; CD40LG; CD40; TNFRSF13C; TNFRSF13B; TNFRSF17; CD80; CD86; CD28; HLA-DMA; HLA-DMB; HLA-DOA; HLA-DOB; HLA-DPA1; HLA-DPB1; HLA-DQA1; HLA-DQA2; HLA-DQB1; HLA-DRA; HLA-DRB1; HLA-DRB3; HLA-DRB4; HLA-DRB5; CCR9; IL10; CCR10; CCL28; IL15; ITGA4; ITGB7; MADCAM1; CXCL12; IL4; AICDA; LOC102723407; CCL25; IL2; LTBR; MAP3K14; ICOS; LOC102723996; ICOSLG; CXCR4; IL15RA; PIGR
Circadian rhythm	ARNTL; NPAS2; CLOCK; RORA; RORB; RORC; NR1D1; PER1; PER3; PER2; CRY1; CRY2; TPTEP2-CSNK1E; CSNK1D; CSNK1E; SKP1; CUL1; RBX1; FBXW11; BTRC; FBXL3; PRKAG2; PRKAG3; PRKAA1; PRKAA2; PRKAB1; PRKAB2; PRKAG1; CREB1
Circadian entrainment	GRIN1; GRIN2A; GRIN2B; GRIN2C; GRIN2D; CALML6; CALML5; CALM1; CALM2; CALM3; CALML3; CALML4; CAMK2A; CAMK2B; CAMK2D; CAMK2G; NOS1; GUCY1A2; GUCY1A1; GUCY1B1; PRKG1; PRKG2; MTNR1B; PLCB1; PLCB2; PLCB3; PLCB4; PRKCA; PRKCB; PRKCG; GNB5; GNAQ; GNB1; GNB2; GNB3; GNG3; GNG4; GNG5; GNG7; GNG10; GNG11; GNGT1; GNGT2; GNG13; GNG2; GNG12; GNB4; GNG8; CACNA1I; CACNA1H; CACNA1G; ITPR1; ITPR3; RYR1; RYR2; RYR3; GRIA1; GRIA2; GRIA3; GRIA4; MAPK1; MAPK3; ADCYAP1R1; ADCY1; ADCY2; ADCY3; ADCY5; ADCY6; ADCY7; ADCY8; ADCY9; ADCY4; ADCY10; PRKACA; PRKACB; PRKACG; GNAS; RASD1; CACNA1C; CACNA1D; NOS1AP; GNAI1; GNAI2; GNAI3; GNAO1; RPS6KA5; CREB1; MTNR1A; KCNJ3; KCNJ5; KCNJ6; KCNJ9; PER1; PER3; PER2; FOS; ADCYAP1
Thermogenesis	CREB3; CREB1; CREB3L4; CREB3L2; CREB3L3; CREB3L1; CREB5; PRKACA; PRKACB; PRKACG; MAPK14; MAPK11; MAPK13; MAPK12; GNAS; ADRB3; ATF2; FGF21; UCP1; PPARGC1A; ADCY1; ADCY2; ADCY3; ADCY5; ADCY6; ADCY7; ADCY8; ADCY9; ADCY4; ADCY10; PPARG; SIRT6; NPR1; PRKG1; PRKG2; KDM3B; KDM3A; ACTL6B; ARID1B; ACTB; SMARCA2; SMARCA4; SMARCB1; SMARCC1; SMARCC2; SMARCD1; SMARCD2; SMARCD3; SMARCE1; ACTG1; DPF3; DPF1; ARID1A; ACTL6A; LIPE; PLIN1; CPT1C; CPT1A; CPT1B; ZNF516; KDM1A; RPS6KA6; RPS6KA1; RPS6KA2; RPS6KA3; TSC1; TSC2; RHEB; MTOR; MLST8; RPTOR; AKT1S1; RPS6KB1; RPS6KB2; RPS6; FGFR1; KLB;

	FRS2; GRB2; SOS1; SOS2; HRAS; KRAS; NRAS; PRDM16; CPT2; ACSL1; ACSL3; ACSL4; ACSL6; ACSL5; CNR1; PNPLA2; MGLL; MAP2K3; MAP3K5; PRKAG2; PRKAG3; PRKAA1; PRKAA2; PRKAB1; PRKAB2; PRKAG1; GCG; BMP8A; BMP8B; SLC25A29; SLC25A20; NDUFC2-KCTD14; COX17; UQCR11; COX20; COX6B2; NDUFA11; COX4I1; COX5B; COX6A1; COX6A2; COX6B1; COX6C; COX7A1; COX7A2; COX7B; COX7C; COX8A; COX10; COX11; COX15; NDUFAF6; CYC1; COX7B2; NDUFAF3; UQCRQ; COX18; COA3; NDUFAF4; UQCR10; COX8C; NDUFS7; COA6; UQCRHL; COX1; COX2; COX3; CYTB; ND1; ND2; ND3; ND4; ND4L; ND5; ND6; NDUFA1; NDUFA2; NDUFA3; NDUFA4; NDUFA5; NDUFA6; NDUFA7; NDUFA8; NDUFA9; NDUFA10; NDUFAB1; NDUFB1; NDUFB2; NDUFB3; NDUFB4; NDUFB5; NDUFB6; NDUFB7; NDUFB8; NDUFB9; NDUFB10; NDUFC1; NDUFC2; NDUFS1; NDUFS2; NDUFS3; NDUFV1; NDUFS4; NDUFS5; NDUFS6; NDUFS8; NDUFV2; NDUFV3; COA5; NDUFA13; NDUFAF1; COX16; COA4; NDUFB11; NDUFAF7; COA1; NDUFA12; NDUFA4L2; SDHA; SDHB; SDHC; SDHD; COA7; UQCRB; UQCRC1; UQCRC2; UQCRFS1; UQCRH; NDUFAF5; COX4I2; COX14; COX19; COX7A2L; NDUFAF2; COX5A; ATP5PD; ATP5MG; ATP6; ATP8; ATP5F1A; ATP5F1B; ATP5F1C; ATP5F1D; ATP5F1E; ATP5PB; ATP5MC1; ATP5MC2; ATP5MC3; ATP5ME; ATP5PF; ATP5PO; ATP5MF; NPPA; NPPB
Long-term potentiation	PRKCA; PRKCB; PRKCG; CAMK4; CREBBP; EP300; CAMK2A; CAMK2B; CAMK2D; CAMK2G; PPP3CA; PPP3CB; PPP3CC; PPP3R1; PPP3R2; CALML6; CALML5; CALM1; CALM2; CALM3; CALML3; CALML4; RPS6KA6; RPS6KA1; RPS6KA2; RPS6KA3; ATF4; GRIN1; GRIN2A; GRIN2B; GRIN2C; GRIN2D; PRKACA; PRKACB; PRKACG; CACNA1C; PPP1CA; PPP1CB; PPP1CC; ADCY1; ADCY8; GRIA1; GRIA2; RAPGEF3; MAPK1; MAPK3; MAP2K1; MAP2K2; ARAF; RAF1; BRAF; HRAS; KRAS; NRAS; RAP1A; RAP1B; PPP1R1A; GNAQ; GRM1; GRM5; PLCB1; PLCB2; PLCB3; PLCB4
Synaptic vesicle cycle	CACNA1A; CACNA1B; STX1B; STX2; STX1A; STX3; RIMS1; CPLX2; CPLX1; CPLX4; CPLX3; RAB3A; VAMP2; SNAP25; NAPA; NSF; SYT1; CLTA; CLTB; CLTC; CLTCL1; AP2M1; AP2S1; AP2A1; AP2A2; AP2B1; DNM1; DNM2; DNM3; STXBP1; UNC13B; UNC13A; UNC13C; SLC32A1; SLC17A8; SLC17A7; SLC17A6; SLC18A1; SLC18A2; SLC18A3; TCIRG1; ATP6V1G3; ATP6V0E2; ATP6V0A2; ATP6V0D2; ATP6V1C2; ATP6V0A4; ATP6V1D; ATP6V1H; ATP6V1A; ATP6V1B1; ATP6V1B2; ATP6V0C; ATP6V1C1; ATP6V1E1; ATP6V0B; ATP6V1G2; ATP6V0A1; ATP6V0E1; ATP6V1E2; ATP6V0D1; ATP6V1F; ATP6V1G1; SLC1A1; SLC1A2; SLC1A3; SLC1A6; SLC1A7; SLC6A1; SLC6A2; SLC6A3; SLC6A4; SLC6A7; SLC6A9; SLC6A11; SLC6A12; SLC6A13; SLC6A5
Neurotrophin signaling pathway	NTRK1; NTRK2; NTRK3; BDNF; NTF4; NTF3; PIK3CA; PIK3CB; PIK3CD; PIK3R1; PIK3R2; PIK3R3; PLCG1; PLCG2; SHC2; SHC4; SHC3; SHC1; GRB2; PRKCD; NGF; NGFR; AKT3; AKT1; AKT2; HRAS; KRAS; NRAS; MAP2K1; MAP2K2; RAF1; BRAF; SOS1; SOS2; MAPK1; MAPK3; RPS6KA6; RPS6KA1; RPS6KA2; RPS6KA3; CRK; CRKL; RAPGEF1; RAP1A; RAP1B; PTPN11; GAB1; BAD; GSK3B; FOXO3; NFKBIA; NFKBIB; NFKBIE; NFKB1; RELA; ATF4; FRS2; PDPK1; FASLG; SORT1; KIDINS220; RPS6KA5; CAMK4; CAMK2A; CAMK2B; CAMK2D; CAMK2G; MATK; MAPK8; MAPK9; MAPK10; RHOA; TP53; MAP3K1; JUN; MAP3K5; IRS1;

	MAPKAPK2; MAPK14; MAPK11; MAPK13; MAPK12; MAP2K5; MAPK7; MAP3K3; ARHGDIA; ARHGDIB; ARHGDIG; SH2B2; SH2B3; SH2B1; ABL1; MAP2K7; CDC42; RAC1; TP73; TRAF6; ZNF274; MAGED1; PRDM4; BEX3; RIPK2; IRAK3; IRAK1; IRAK2; IRAK4; YWHAE; IKBKB; BCL2; CALML6; CALML5; CALM1; CALM2; CALM3; CALML3; CALML4; PSEN1; PSEN2; BAX
Glutamatergic synapse	GRM7; GRM8; GRIK1; GRIK2; GRIK3; GRIK4; GRIK5; GRIA1; GRIA2; GRIA3; GRIA4; GRIN3A; GRIN3B; GRIN1; GRIN2A; GRIN2B; GRIN2C; GRIN2D; GRM1; GRM5; GRM2; GRM3; GRM4; GRM6; SLC1A1; SLC1A6; CACNA1C; CACNA1D; SLC1A2; SLC1A3; ADCY1; ADCY2; ADCY3; ADCY5; ADCY6; ADCY7; ADCY8; ADCY9; ADCY4; SLC38A3; SLC38A2; SLC38A1; SLC17A8; SLC17A7; SLC17A6; GNAS; PRKACA; PRKACB; PRKACG; GNAQ; PLCB1; PLCB2; PLCB3; PLCB4; ITPR1; ITPR2; ITPR3; PRKCA; PRKCB; PRKCG; TRPC1; GNB5; GNAI1; GNAI2; GNAI3; GNAO1; GNB1; GNB2; GNB3; GNG3; GNG4; GNG5; GNG7; GNG10; GNG11; GNGT1; GNGT2; GNG13; GNG2; GNG12; GNB4; GNG8; PPP3CA; PPP3CB; PPP3CC; PPP3R1; PPP3R2; GLS2; GLS; GLUL; SLC1A7; GRK2; GRK3; CACNA1A; KCNJ3; PLA2G4B; PLA2G4E; PLA2G4F; PLA2G4D; PLA2G4A; PLA2G4C; JMJD7-PLA2G4B; PLD1; PLD2; MAPK1; MAPK3; HOMER3; HOMER2; HOMER1; DLG4; DLGAP1; SHANK2; SHANK1; SHANK3
Cholinergic synapse	CHRNA3; CHRNA4; CHRNA7; CHRNB2; CHRNB4; CHRNA6; CHRM1; CHRM2; CHRM4; ACHE; SLC18A3; ADCY1; ADCY2; ADCY3; ADCY5; ADCY6; ADCY7; ADCY8; ADCY9; ADCY4; PRKACA; PRKACB; PRKACG; GNA11; GNAQ; PLCB1; PLCB2; PLCB3; PLCB4; ITPR1; ITPR2; ITPR3; PRKCA; PRKCB; PRKCG; CHAT; SLC5A7; CACNA1A; CACNA1B; GNB5; GNAI1; GNAI2; GNAI3; GNAO1; GNB1; GNB2; GNB3; GNG3; GNG4; GNG5; GNG7; GNG10; GNG11; GNGT1; GNGT2; GNG13; GNG2; GNG12; GNB4; GNG8; CHRM5; CHRM3; KCNJ18; KCNJ2; KCNJ4; KCNJ12; KCNJ14; KCNQ1; KCNQ2; KCNQ3; KCNQ5; KCNQ4; MAPK1; MAPK3; CREB3; CREB1; CREB3L4; ATF4; CREB3L2; CREB3L3; CREB3L1; CREB5; CAMK4; CAMK2A; CAMK2B; CAMK2D; CAMK2G; PIK3CA; PIK3CB; PIK3CD; PIK3R1; PIK3R2; PIK3R3; AKT3; AKT1; AKT2; BCL2; CACNA1C; CACNA1D; CACNA1F; CACNA1S; PIK3R6; PIK3R5; PIK3CG; HRAS; KRAS; NRAS; MAP2K1; FOS; JAK2; FYN; KCNJ3; KCNJ6
GABAergic synapse	GABBR1; GABBR2; SLC6A11; SLC6A13; SLC32A1; ADCY1; ADCY2; ADCY3; ADCY5; ADCY6; ADCY7; ADCY8; ADCY9; ADCY4; GAD1; GAD2; SLC6A1; SLC6A12; CACNA1A; CACNA1B; GNB5; GNAI1; GNAI2; GNAI3; GNAO1; GNB1; GNB2; GNB3; GNG3; GNG4; GNG5; GNG7; GNG10; GNG11; GNGT1; GNGT2; GNG13; GNG2; GNG12; GNB4; GNG8; GABRA1; GABRA2; GABRA3; GABRA4; GABRA5; GABRA6; GABRB1; GABRB2; GABRB3; GABRD; GABRE; GABRG1; GABRG2; GABRG3; GABRP; GABRQ; ABAT; GLUL; KCNJ6; GLS2; GLS; PRKACA; PRKACB; PRKACG; GPHN; GABARAP; GABARAPL2; GABARAPL1; NSF; TRAK2; PLCL1; HAP1; CACNA1C; CACNA1D; CACNA1F; CACNA1S; GABRR3; GABRR1; GABRR2; SLC12A5; PRKCA; PRKCB; PRKCG; SRC; SLC38A3; SLC38A5
Long-term depression	PLCB1; PLCB2; PLCB3; PLCB4; GNA11; GNAQ; GRIA1; GRIA2; GRIA3; RYR1; PLA2G4B; PLA2G4E; PLA2G4F; PLA2G4D; PLA2G4A; PLA2G4C; JMJD7-

	PLA2G4B; GNA13; GNA12; GNAI1; GNAI2; GNAI3; GNAO1; GNAS; GNAZ; NOS1; GRM1; PRKCA; PRKCB; PRKCG; IGF1R; CRHR1; CRH; IGF1; GUCY1A2; GUCY1A1; GUCY1B1; GRID2; PRKG1; PRKG2; CACNA1A; PPP1R17; MAPK1; MAPK3; MAP2K1; MAP2K2; ARAF; RAF1; BRAF; HRAS; KRAS; NRAS; PPP2CA; PPP2CB; PPP2R1A; PPP2R1B; LYN
Olfactory transduction	GNAL; GNB1; GNG7; GNG13; ADCY3; LOC102723532; OR4N4C; OR5I1; LOC107987545; LOC112268384; OR52E2; OR52J3; OR51L1; OR51A7; OR51S1; OR51F2; OR52R1; OR4C46; OR4X2; OR4B1; OR52M1; OR52K2; OR5P2; OR5P3; OR8I2; OR2D3; OR2D2; OR52W1; OR56A4; OR56A1; OR2AP1; OR10P1; OR10AD1; OR10A7; OR4K14; OR4L1; OR11H6; OR4D2; OR7D4; OR7G1; OR1M1; OR1I1; OR10H4; OR2M5; OR2M3; OR2T12; OR14C36; OR2T34; OR2T10; OR2T4; OR2T11; OR10J5; OR2AJ1; OR2B11; OR10T2; OR6P1; OR10X1; OR10Z1; OR6K6; OR6N1; OR9A4; OR2Y1; OR9A2; OR2A14; OR6B1; OR2F2; OR13C5; OR13C8; OR13C3; OR13C4; OR13F1; OR1L8; OR1N2; OR1N1; OR52B4; OR52I2; OR51E1; OR10A5; OR2AG1; OR6B3; OR1Q1; OR7D2; OR56B4; OR8U1; OR4C16; OR4C11; OR4S2; OR4C6; OR5D14; OR5L1; OR5D18; OR5AS1; OR8K5; OR5T2; OR8H1; OR8K3; OR8J1; OR8U3; OR5M3; OR5M8; OR5M11; OR5AR1; OR8B12; OR8G5; OR10G8; OR10G9; OR10S1; OR6T1; OR4D5; OR6Q1; OR9I1; OR9Q1; OR9Q2; OR1S2; OR1S1; OR10Q1; OR5B17; OR5B21; OR5A2; OR5A1; OR4D6; OR4D11; OR52A1; OR6C74; OR6C3; OR2T6; OR1L4; OR52B2; OR4C3; OR4S1; OR51F1; OR1C1; OR1A2; OR2F1; OR2B6; OR1J4; OR2M4; OR2L2; OR2K2; OR7A17; OR5L2; OR5K1; OR5H1; OR10J1; OR8G2P; OR8B8; OR8G1; OR10A3; OR10D3; OR12D2; OR12D1; OR11A1; OR10H3; OR10G3; OR10G2; OR10H2; OR10H1; OR8B2; OR7E24; OR7C2; OR7A5; OR7C1; OR4F4; OR4F3; OR4E2; OR4E1; OR4D1; OR2W1; OR2V1; OR2T1; OR2J2; OR2H1; OR1L3; OR1L1; OR1J2; OR51B5; OR10AG1; OR5J2; OR4C13; OR4C12; OR52Z1; OR51V1; OR8D1; OR8D2; OR8B4; OR9G4; OR10A4; OR6C6; OR4K3; OR4N4; OR2Z1; OR10H5; OR2L13; OR14A16; OR2V2; OR13C9; OR13D1; OR8D4; OR5F1; OR5AP2; OR52L1; OR2AG2; OR52B6; OR2AT4; OR10A2; OR6C2; OR6C4; OR8S1; OR6S1; OR6F1; OR14K1; OR2W3; OR2T8; OR2T3; OR10R2; OR2T29; OR6V1; OR2A12; OR2A1; OR1J1; OR1B1; OR13H1; OR56B1; OR14A2; OR6B2; OR52K1; OR52I1; OR51D1; OR52A5; OR51B6; OR51M1; OR51Q1; OR51I1; OR51I2; OR52D1; OR52H1; OR52N4; OR52N5; OR52N2; OR52E6; OR52E8; OR52E4; OR52E5; OR56A3; OR56A5; OR10A6; OR4X1; OR5D13; OR5D16; OR5W2; OR8H2; OR8H3; OR5T3; OR5T1; OR8K1; OR5M9; OR5M10; OR5M1; OR9G1; OR5AK2; OR5B2; OR5B12; OR5AN1; OR4D10; OR4D9; OR10V1; OR6X1; OR6M1; OR10G4; OR10G7; OR8B3; OR8A1; OR6C1; OR6C75; OR6C76; OR6C70; OR4N2; OR4K2; OR4Q2; OR4K13; OR4K17; OR4N5; OR11G2; OR11H7; OR11H4; OR5AU1; OR4M2; OR4F6; OR4F15; OR7G2; OR7G3; OR7A10; OR10K2; OR10K1; OR6Y1; OR6K3; OR10J4; OR11L1; OR2L8; OR2AK2; OR2L3; OR2M2; OR2T33; OR2M7; OR2G6; OR10AC1; OR2A25; OR13J1; OR13C2; OR1L6; OR5C1; OR1K1; OR2A5; OR2A7; OR51H1; OR51T1; OR51A4; OR51A2; OR2T2; OR2T5; OR14I1; OR5K2; OR2A42; OR2T27; OR2T35; OR4A47; OR4C45; OR5H14; OR5H15; OR5K3; OR5K4; OR6C65; OR6C68; OR11H12; OR4F21; OR5B3; OR9K2; OR4Q3; OR4M1; OR10J3; OR13G1; OR1F12; OR2B3; OR2J1; OR2J3; OR14J1; OR10C1; OR2A2; OR1D2; OR1F1; OR2C1;

	OR3A1; OR3A2; OR8U8; OR8U9; OR9G9; OR2S2; OR1D4; OR4F29; OR13A1; OR5H6; OR52E1; OR5H2; OR4K5; OR2H2; OR51G1; OR11H2; OR51B4; OR51B2; OR4C5; OR51J1; OR52N1; OR5AL1; OR10G6; OR4F5; OR2A4; OR4K1; OR6J1; OR5AC2; OR11H1; OR4F17; OR4K15; OR8J3; OR51G2; OR51E2; OR4P4; OR4C15; OR4A5; OR4A16; OR4A15; OR10W1; OR2AE1; OR4F16; OR6N2; OR6K2; OR2T7; OR2L5; OR2G3; OR2G2; OR2C3; OR5V1; OR2B2; OR12D3; OR1A1; OR1D5; OR1E1; OR1E2; OR1E3; OR1G1; OR3A3; OR6A2; CNGB1; CNGA2; CNGA4; ANO2; GUCY2D; CNGA3; PRKG1; PRKG2; CAMK2A; CAMK2B; CAMK2D; CAMK2G; CALML6; CALML5; CALM1; CALM2; CALM3; CALML3; CALML4; PDE1A; PDE1C; PDE1B; PRKACA; PRKACB; PRKACG; ARRB1; ARRB2; GRK2; GRK3; SLC24A4; SLC8A2; SLC8A1; SLC8A3; PDE2A; NCALD; RGS2
Taste transduction	TRPM5; TAS1R2; PLCB1; PLCB2; PLCB3; PLCB4; ITPR3; SCN2A; SCN3A; SCN9A; PDE1A; PDE1C; PDE1B; CACNA1A; CACNA1C; PKD1L3; HCN4; CALHM1; SCNN1A; SCNN1B; SCNN1G; ENTPD2; TAS2R39; TAS2R40; TAS2R41; TAS2R43; TAS2R31; TAS2R45; TAS2R46; TAS2R30; TAS2R19; TAS2R20; TAS2R50; TAS2R60; TAS2R42; TAS2R3; TAS2R4; TAS2R16; TAS2R1; TAS2R9; TAS2R8; TAS2R7; TAS2R13; TAS2R10; TAS2R14; TAS2R5; TAS2R38; TAS1R1; GRM1; GRM4; TAS1R3; P2RY4; KCNK5; P2RX2; ASIC2; PKD2L1; P2RY1; GABRA1; GABRA2; GABRA3; GABRA4; GABRA5; GABRA6; GABBR1; GABBR2; HTR1A; HTR1B; HTR1D; HTR1E; HTR1F; P2RX3; CHRM3; HTR3C; HTR3D; HTR3E; HTR3A; HTR3B; ADCY6; ADCY8; ADCY4; PRKACA; PRKACB; PRKACG; GNB3; GNAT3; GNG13
Inflammatory mediator regulation of TRP channels	P2RY2; BDKRB1; BDKRB2; HTR2A; HTR2B; HTR2C; HRH1; PTGER2; PTGER4; TRPV1; ASIC2; ASIC1; ASIC5; ASIC4; ASIC3; IL1R1; IL1RAP; NTRK1; IL1B; NGF; GNAQ; PLA2G4B; PLA2G4E; PLA2G4F; PLA2G4D; PLA2G4A; PLA2G6; PLA2G4C; JMJD7-PLA2G4B; PLCB1; PLCB2; PLCB3; PLCB4; ALOX12; GNAS; ADCY1; ADCY2; ADCY3; ADCY5; ADCY6; ADCY7; ADCY8; ADCY9; ADCY4; PRKCE; PRKACA; PRKACB; PRKACG; CAMK2A; CAMK2B; CAMK2D; CAMK2G; MAPK14; MAPK8; MAPK11; MAPK9; MAPK10; MAPK13; MAPK12; PLCG1; PLCG2; PIK3CA; PIK3CB; PIK3CD; PIK3R1; PIK3R2; PIK3R3; PRKCD; SRC; ITPR1; ITPR2; ITPR3; TRPA1; CYP2J2; CYP4A11; CYP4A22; TRPV4; TRPM8; PPP1CA; PPP1CB; PPP1CC; TRPV2; TRPV3; IGF1; F2RL1; PRKCA; PRKCB; PRKCG; PRKCH; PRKCQ; CALML6; CALML5; CALM1; CALM2; CALM3; CALML3; CALML4; MAP2K3; MAP2K6; KNG1
Regulation of actin cytoskeleton	F2; TMSB4X; TMSB4Y; ARPC5; ARPC4; ARPC3; ARPC1B; ARPC2; ARPC1A; ARPC5L; PFN3; PFN4; PFN1; PFN2; WASF2; WASF1; BRK1; APC2; APC; WASL; SPATA13; ARHGEF4; ABI2; NCKAP1; NCKAP1L; CYFIP1; CYFIP2; BAIAP2; SSH1; SSH3; SSH2; CFL1; CFL2; LIMK1; LIMK2; GSN; SCIN; VCL; PIKFYVE; PIP5K1C; PIP4K2A; PIP4K2C; PIP5K1A; PIP5K1B; PIP4K2B; DIAPH1; DIAPH2; ACTN4; ACTN1; MSN; RDX; EZR; PXN; SLC9A1; PPP1R12A; PPP1R12B; PPP1R12C; PPP1CA; PPP1CB; PPP1CC; MYL12B; MYL9; MYL12A; MYLPF; MYL2; MYL5; MYL7; MYL10; MYLK4; MYLK; MYLK2; MYLK3; GIT1; ENAH; DIAPH3; ROCK1; ROCK2; PAK4; BUB1B-PAK6; PAK1; PAK2; PAK3; PAK6; PAK5; ARHGEF7; ARHGEF6; CDC42; RAC1; RAC2; RAC3; RHOA; ARHGEF12; ARHGEF1; IQGAP2;

	IQGAP3; IQGAP1; ARHGAP35; VAV3; TIAM1; VAV1; VAV2; MAPK1; MAPK3; MAP2K1; MAP2K2; ARAF; MOS; RAF1; BRAF; PIK3CA; PIK3CB; PIK3CD; PIK3R1; PIK3R2; PIK3R3; DOCK1; FGD1; FGD3; GNG12; CRK; CRKL; BCAR1; PTK2; CHRM1; CHRM2; CHRM3; CHRM4; CHRM5; BDKRB1; BDKRB2; SRC; ITGA11; ITGA6; ITGA1; ITGA2; ITGA2B; ITGA3; ITGA4; ITGA5; ITGA7; ITGA9; ITGAD; ITGAE; ITGAL; ITGAM; ITGAV; ITGAX; ITGB1; ITGB2; ITGB3; ITGB4; ITGB5; ITGB6; ITGB7; ITGB8; ITGA10; ITGA8; FN1; RRAS2; MRAS; HRAS; KRAS; NRAS; RRAS; GNA13; GNA12; SOS1; SOS2; EGFR; FGFR1; FGFR3; FGFR2; FGFR4; INSRR; PDGFRA; PDGFRB; EGF; FGF1; FGF2; FGF3; FGF4; FGF5; FGF6; FGF7; FGF8; FGF9; FGF10; FGF20; FGF21; FGF22; PDGFA; PDGFB; PDGFC; PDGFD; FGF23; FGF18; FGF17; FGF16; FGF19; INS; F2R; ACTB; ACTG1; MYH9; MYH10; MYH11; MYH14; CXCR4; LPAR1; LPAR4; LPAR5; LPAR2; CXCL12; KNG1
Insulin signaling pathway	TSC1; INPP5A; INPPL1; IKBKB; MAPK8; MAPK9; MAPK10; PTPRF; PTPN1; SOCS4; SOCS1; SOCS2; SOCS3; ELK1; MKNK2; MKNK1; MAPK1; MAPK3; FASN; PRKAG2; PRKAG3; PRKAA1; PRKAA2; PRKAB1; PRKAB2; PRKAG1; INS; LIPE; PPP1CA; PPP1CB; PPP1CC; PPP1R3A; PPP1R3C; PPP1R3D; PPP1R3B; PPP1R3F; PPP1R3E; PKLR; GYS1; GYS2; CALML6; CALML5; PHKA1; PHKA2; PHKB; PHKG1; PHKG2; CALM1; CALM2; CALM3; CALML3; CALML4; PRKACA; PRKACB; PRKACG; PRKAR1A; PRKAR1B; PRKAR2A; PRKAR2B; ACACA; ACACB; EIF4EBP1; RPS6; PYGB; PYGL; PYGM; FOXO1; RPTOR; SOS1; SOS2; HRAS; KRAS; NRAS; EIF4E; EIF4E1B; EIF4E2; MAP2K1; MAP2K2; SHC2; SHC4; SHC3; SHC1; TRIP10; INSR; RPS6KB1; RPS6KB2; GRB2; BAD; TSC2; ARAF; RAF1; BRAF; PCK1; PCK2; G6PC; G6PC2; G6PC3; MTOR; PDE3B; FBP1; FBP2; EXOC7; PPARGC1A; SORBS1; RHEB; AKT3; AKT1; AKT2; PIK3CA; PIK3CB; PIK3CD; PIK3R1; PIK3R2; PIK3R3; RHOQ; GSK3B; SREBF1; RAPGEF1; GCK; HK1; HK2; HK3; HKDC1; CRK; CRKL; PRKCI; PRKCZ; SH2B2; SLC2A4; PDPK1; CBL; CBLB; IRS1; IRS4; IRS2
Insulin secretion	SLC2A1; SLC2A2; KCNJ11; CACNA1C; CACNA1D; CACNA1F; CACNA1S; CHRM3; GLP1R; PLCB1; PLCB2; PLCB3; PLCB4; ADCY1; ADCY2; ADCY3; ADCY5; ADCY6; ADCY7; ADCY8; ADCY9; ADCY4; PRKACA; PRKACB; PRKACG; GNAS; GNA11; GNAQ; GCG; GCK; INS; PRKCA; PRKCB; PRKCG; RYR2; CREB3; CREB1; ATF2; ATF6B; CREB3L4; ATF4; CREB3L2; CREB3L3; CREB3L1; CREB5; CAMK2A; CAMK2B; CAMK2D; CAMK2G; FFAR1; TRPM4; ATP1B4; ATP1A1; ATP1A2; ATP1A3; ATP1A4; ATP1B1; ATP1B2; ATP1B3; FXYD2; RAPGEF4; ABCC8; RIMS2; STX1A; SNAP25; VAMP2; CCKAR; GIP; GPR119; ADCYAP1; ADCYAP1R1; ITPR3; PDX1; PCLO; RAB3A; KCNMB2; KCNU1; KCNMB3; KCNMB4; KCNMA1; KCNMB1; KCNN1; KCNN2; KCNN3; KCNN4; CCK
GnRH signaling pathway	PRKCA; PRKCB; PRKCD; ADCY1; ADCY2; ADCY3; ADCY5; ADCY6; ADCY7; ADCY8; ADCY9; ADCY4; CGA; LHB; PLCB1; PLCB2; PLCB3; PLCB4; CDC42; ATF4; CAMK2A; CAMK2B; CAMK2D; CAMK2G; MAP2K3; MAP2K6; MAPK14; MAPK11; MAPK13; MAPK12; MAP3K2; MAP3K1; MAP3K3; MAP3K4; MAPK7; PLA2G4B; PLA2G4E; PLA2G4F; PLA2G4D; PLA2G4A; PLA2G4C; JMJD7-PLA2G4B; ITPR1; ITPR2; ITPR3; CALML6; CALML5; CALM1; CALM2; CALM3;

	CALML3; CALML4; GNA11; GNAQ; MMP14; GNRHR; PLD1; PLD2; GNAS; GNRH1; GNRH2; SRC; MMP2; PRKACA; PRKACB; PRKACG; CACNA1C; CACNA1D; CACNA1F; CACNA1S; ELK1; MAPK1; MAPK3; FSHB; MAP2K1; MAP2K2; RAF1; HRAS; KRAS; NRAS; SOS1; SOS2; GRB2; EGFR; HBEGF; PTK2B; MAP2K7; MAP2K4; MAPK8; MAPK9; MAPK10; JUN; EGR1
Ovarian steroidogenesis	HSD3B1; HSD3B2; HSD17B2; AKR1C3; LHCGR; CGA; LHB; STAR; CYP11A1; CYP17A1; GNAS; ADCY1; ADCY2; ADCY3; ADCY5; ADCY6; ADCY7; ADCY8; ADCY9; ADCY4; FSHB; FSHR; CYP19A1; CYP1B1; CYP1A1; HSD17B1; HSD17B7; PRKACA; PRKACB; PRKACG; ACOT2; ACOT4; ACOT1; INS; INSR; IGF1; IGF1R; LDLR; ALOX5; CYP2J2; PTGS2; PLA2G4B; PLA2G4E; PLA2G4F; PLA2G4D; PLA2G4A; PLA2G4C; JMJD7-PLA2G4B; BMP15; BMP6; SCARB1
Estrogen signaling pathway	ESR1; ESR2; GPER1; SRC; MMP2; MMP9; HBEGF; EGFR; FOS; JUN; SP1; PRKACA; PRKACB; PRKACG; HRAS; KRAS; NRAS; RAF1; MAP2K1; MAP2K2; MAPK1; MAPK3; CREB3; CREB1; ATF2; ATF6B; CREB3L4; ATF4; CREB3L2; CREB3L3; CREB3L1; CREB5; GNAQ; PLCB1; PLCB2; PLCB3; PLCB4; PRKCD; ADCY1; ADCY2; ADCY3; ADCY5; ADCY6; ADCY7; ADCY8; ADCY9; ADCY4; KCNJ3; KCNJ5; KCNJ6; KCNJ9; GABBR1; GABBR2; PIK3CA; PIK3CB; PIK3CD; PIK3R1; PIK3R2; PIK3R3; AKT3; AKT1; AKT2; NOS3; SHC2; SHC4; SHC3; SHC1; GRB2; SOS1; SOS2; HSP90AA1; HSP90AB1; HSP90B1; FKBP4; FKBP5; HSPA1A; HSPA1B; HSPA1L; HSPA2; HSPA6; HSPA8; GNAS; GNAI1; GNAI2; GNAI3; GNAO1; ITPR1; ITPR2; ITPR3; CALML6; CALML5; CALM1; CALM2; CALM3; CALML3; CALML4; OPRM1; GRM1; NCOA2; NCOA3; NCOA1; BCL2; EBAG9; LOC100653049; KRT40; KRT25; KRT28; KRT24; KRT23; KRT27; KRT26; KRT9; KRT10; KRT12; KRT13; KRT14; KRT15; KRT16; KRT17; KRT18; KRT19; KRT31; KRT32; KRT33A; KRT33B; KRT34; KRT35; KRT39; KRT20; KRT38; KRT37; KRT36; CTSD; TFF1; PGR; TGFA; RARA; POMC
Thyroid hormone synthesis	PRKACA; PRKACB; PRKACG; CGA; TSHB; TSHR; GNAQ; PLCB1; PLCB2; PLCB3; PLCB4; GNAS; ADCY1; ADCY2; ADCY3; ADCY5; ADCY6; ADCY7; ADCY8; ADCY9; ADCY4; TG; SLC5A5; ATP1B4; ATP1A1; ATP1A2; ATP1A3; ATP1A4; ATP1B1; ATP1B2; ATP1B3; FXYD2; SLC26A4; DUOX2; DUOX1; TPO; IYD; PRKCA; PRKCB; PRKCG; TTF1; TTF2; PAX8; CREB3; CREB1; ATF2; ATF6B; CREB3L4; ATF4; CREB3L2; CREB3L3; CREB3L1; CREB5; DUOXA2; ITPR1; ITPR2; ITPR3; HSPA5; HSP90B1; CANX; PDIA4; LRP2; GPX6; GPX1; GPX2; GPX3; GPX5; GPX7; GPX8; ASGR1; ASGR2; GSR; ALB; TTR; SERPINA7
Oxytocin signaling pathway	GNAQ; MYLK4; MYLK; MYLK2; MYLK3; ITPR1; ITPR2; ITPR3; CALML6; CALML5; CALM1; CALM2; CALM3; CALML3; CALML4; ADCY1; ADCY2; ADCY3; ADCY5; ADCY6; ADCY7; ADCY8; ADCY9; ADCY4; PRKACA; PRKACB; PRKACG; GNAS; OXTR; OXT; PLCB1; PLCB2; PLCB3; PLCB4; PRKCA; PRKCB; PRKCG; HRAS; KRAS; NRAS; RAF1; MAP2K1; MAP2K2; MAPK1; MAPK3; PLA2G4B; PLA2G4E; PLA2G4F; PLA2G4D; PLA2G4A; PLA2G4C; JMJD7-PLA2G4B; EEF2K; EEF2; CAMK1D; CAMK1G; CAMK4; CAMK2A; CAMK2B; CAMK2D; CAMK2G; CAMK1; NOS3; GUCY1A2; GUCY1A1; GUCY1B1; MYL9; MYL6B; MYL6; ACTB; ACTG1; RHOA; ROCK1; ROCK2; PPP1R12A; PPP1R12B; PPP1R12C; PPP1CA; PPP1CB; PPP1CC; PTGS2; CACNG3; CACNG2; CACNG5;

	CACNG4; CACNA2D3; CACNG8; CACNG7; CACNG6; CACNA1C; CACNA1D; CACNA1F; CACNA1S; CACNA2D1; CACNB1; CACNB2; CACNB3; CACNB4; CACNG1; CACNA2D2; CACNA2D4; NPR1; NPR2; CAMKK2; PRKAG2; PRKAG3; PRKAA1; PRKAA2; PRKAB1; PRKAB2; PRKAG1; MAP2K5; MAPK7; PPP3CA; PPP3CB; PPP3CC; PPP3R1; PPP3R2; NFATC1; NFATC2; NFATC3; NFATC4; RGS2; RCAN1; JUN; FOS; MEF2C; CCND1; ELK1; RYR1; RYR2; RYR3; CD38; TRPM2; GNAI1; GNAI2; GNAI3; GNAO1; PIK3R6; PIK3R5; PIK3CG; SRC; EGFR; CDKN1A; KCNJ3; KCNJ5; KCNJ6; KCNJ9; KCNJ18; KCNJ2; KCNJ4; KCNJ12; KCNJ14; NPPA
Glucagon signaling pathway	SLC2A1; SLC2A2; PDHA1; PDHA2; PDHB; PKM; GCK; PFKL; PFKM; PFKP; PGAM4; PGAM1; PGAM2; GCG; GCGR; GNAS; ADCY2; PRKACA; PRKACB; PRKACG; GYS1; GYS2; PYGB; PYGL; PYGM; GNAQ; PLCB1; PLCB2; PLCB3; PLCB4; CREB3; CREB1; ATF2; CREB3L4; ATF4; CREB3L2; CREB3L3; CREB3L1; CREB5; PPP4R3C; PPP4C; PPP4R3A; PPP4R3B; CRTC2; SIK2; CREBBP; EP300; PPARGC1A; PCK1; PCK2; G6PC; G6PC2; G6PC3; FOXO1; PRMT1; SIK1B; SIK1; AKT3; AKT1; AKT2; PDE3B; CPT1C; CPT1A; CPT1B; PHKA1; PHKA2; PHKB; PHKG1; PHKG2; CALML6; CALML5; CALM1; CALM2; CALM3; CALML3; CALML4; PPP3CA; PPP3CB; PPP3CC; PPP3R1; PPP3R2; FBP1; FBP2; PFKFB1; ACACA; ACACB; PRKAG2; PRKAG3; PRKAA1; PRKAA2; PRKAB1; PRKAB2; PRKAG1; CAMK2A; CAMK2B; CAMK2D; CAMK2G; ITPR1; ITPR2; ITPR3; LDHAL6A; LDHA; LDHB; LDHC; LDHAL6B; SIRT1; PPARA
Regulation of lipolysis in adipocytes	GNAS; ADCY1; ADCY2; ADCY3; ADCY5; ADCY6; ADCY7; ADCY8; ADCY9; ADCY4; PRKACA; PRKACB; PRKACG; ADRB1; ADRB2; ADRB3; LIPE; NPR1; PRKG1; PRKG2; PLIN1; LOC100509620; LOC112267859; AQP7; MGLL; PNPLA2; ABHD5; PTGER3; GNAI1; GNAI2; GNAI3; INS; INSR; PDE3B; PIK3CA; PIK3CB; PIK3CD; PIK3R1; PIK3R2; PIK3R3; IRS1; IRS4; IRS2; PLAAT3; PTGS1; PTGS2; ADORA1; TSHR; FABP4; CGA; TSHB; NPY1R; NPY; AKT3; AKT1; AKT2; NPPA
Renin secretion	GNAS; ADCY5; ADCY6; PRKACA; PRKACB; PRKACG; ADRB1; ADRB2; ADRB3; NPR1; GNAI1; GNAI2; GNAI3; ADORA1; ORAI1; AGTR1; GNAQ; PLCB1; PLCB2; PLCB3; PLCB4; ITPR1; ITPR2; ITPR3; GUCY1A2; GUCY1A1; GUCY1B1; PRKG2; PDE3A; PDE3B; CREB1; REN; CTSB; AGT; ACE; PTGER2; PTGER4; EDNRA; KCNMA1; PDE1A; PDE1C; PDE1B; CLCA1; CLCA4; CLCA2; CACNA1C; CACNA1D; CACNA1F; CACNA1S; KCNJ2; CALML6; CALML5; CALM1; CALM2; CALM3; CALML3; CALML4; PPP3CA; PPP3CB; PPP3CC; PPP3R1; PPP3R2; AQP1; ADCYAP1R1; ADCYAP1; NPPA
Relaxin signaling pathway	AKT3; AKT1; AKT2; RXFP1; NFKBIA; NFKB1; RELA; NOS3; MAPK1; MAPK3; PIK3CA; PIK3CB; PIK3CD; PIK3R1; PIK3R2; PIK3R3; GNB5; GNB1; GNB2; GNB3; GNG3; GNG4; GNG5; GNG7; GNG10; GNG11; GNGT1; GNGT2; GNG13; GNG2; GNG12; GNB4; GNG8; CREB3; CREB1; ATF2; ATF6B; CREB3L4; ATF4; CREB3L2; CREB3L3; CREB3L1; CREB5; PRKCZ; ADCY5; PRKACA; PRKACB; PRKACG; RLN1; RLN2; EDN1; EDNRB; GNAS; GNAO1; GNAI1; GNAI2; GNAI3; VEGFD; VEGFA; VEGFB; VEGFC; NOS2; MMP2; MMP9; NOS1; TGFB1; SMAD2; ACTA2; COL1A1; COL1A2; COL3A1; COL4A1; COL4A2; COL4A3; COL4A4; COL4A5; COL4A6; MMP1; MMP13; RXFP2; INSL3; ADCY1; ADCY2; ADCY3; ADCY6; ADCY7; ADCY8; ADCY9; ADCY4; RXFP3; RLN3; SRC; HRAS; KRAS; NRAS;

	RAF1; MAP2K1; MAP2K2; PLCB1; PLCB2; PLCB3; PLCB4; PRKCA; SHC2; SHC4; SHC3; SHC1; SOS1; SOS2; FOS; JUN; RXFP4; GNA15; INSL5; TGFBR1; TGFBR2; MAP2K7; MAP2K4; MAPK14; MAPK11; MAPK13; MAPK12; MAPK8; MAPK9; MAPK10; EGFR; GRB2
Cortisol synthesis and secretion	ITPR1; ITPR2; ITPR3; PLCB1; PLCB2; PLCB3; PLCB4; CACNA1C; CACNA1D; CACNA1F; CACNA1S; GNA11; GNAQ; CACNA1I; CACNA1H; CACNA1G; CREB3; CREB1; ATF2; ATF6B; CREB3L4; ATF4; CREB3L2; CREB3L3; CREB3L1; CREB5; ADCY1; ADCY2; ADCY3; ADCY5; ADCY6; ADCY7; ADCY8; ADCY9; ADCY4; PDE8A; PDE8B; AGTR1; MC2R; GNAS; STAR; HSD3B1; HSD3B2; PRKACA; PRKACB; PRKACG; ORAI1; LDLR; SCARB1; CYP11A1; CYP21A2; MRAP; CYP11B1; KCNK2; NCEH1; CYP17A1; KCNA4; KCNK3; POMC; NR5A1; NR4A1; SP1; PBX1; NR0B1; AGT
Parathyroid hormone synthesis	secretion and action
GnRH secretion	GNA11; GNAQ; PLCB1; PLCB2; PLCB3; PLCB4; ITPR1; ITPR2; ITPR3; PRKCA; PRKCB; PRKCG; PIK3CA; PIK3CB; PIK3CD; PIK3R1; PIK3R2; PIK3R3; AKT3; AKT1; AKT2; CACNA1I; CACNA1H; CACNA1G; HRAS; KRAS; NRAS; MAP2K1; MAP2K2; MAPK1; MAPK3; KCNJ3; KCNJ5; KCNJ6; KCNJ9; KCNJ11; KISS1R; ARRB1; ARRB2; KISS1; TRPC1; TRPC4; TRPC5; GNRH1; GNRH2; GABBR1; GABBR2; HCN1; HCN3; HCN2; CGA; SPP1; LHB; GPER1; ESR2; KCNN1; KCNN2; KCNN3; KCNN4; CACNA1C; CACNA1D; CACNA1F; CACNA1S; RAF1
Type II diabetes mellitus	PKLR; PKM; PIK3CA; PIK3CB; PIK3CD; PIK3R1; PIK3R2; PIK3R3; SLC2A4; IRS1; INSR; INS; SOCS4; SOCS1; SOCS2; SOCS3; MAPK1; MAPK3; ADIPOQ; IKBKB; PRKCD; PRKCE; PDX1; IRS4; IRS2; MAPK8; MAPK9; MAPK10; PRKCZ; TNF; CACNA1A; CACNA1B; CACNA1C; CACNA1D; CACNA1E; CACNA1G; GCK; HK1; HK2; HK3; HKDC1; MTOR; MAFA; SLC2A2; KCNJ11; ABCC8
Insulin resistance	MAPK8; MAPK9; MAPK10; TNF; TNFRSF1A; SLC27A5; SLC27A4; SLC27A3; SLC27A2; SLC27A6; SLC27A1; IKBKB; MTOR; RPS6KB1; RPS6KB2; INSR; IRS1; PIK3CA; PIK3CB; PIK3CD; PIK3R1; PIK3R2; PIK3R3; AKT3; AKT1; AKT2; GSK3B; PRKCB; PRKCD; PRKCQ; CD36; SLC2A4; TBC1D4; INS; PTPA; IL6; STAT3; SOCS3; CPT1B; NFKBIA; NFKB1; RELA; PPP1CA; PPP1CB; PPP1CC; PPP1R3A; PPP1R3C; PPP1R3D; PPP1R3B; PPP1R3E; GYS1; GYS2; NOS3; PTPN1; PTPN11; PTPRF; PRKCZ; RPS6KA6; RPS6KA1; RPS6KA2; RPS6KA3; IRS2; PRKCE; ACACB; MLXIP; MLXIPL; MLX; PPARGC1B; SREBF1; SLC2A2; FOXO1; PCK1; PCK2; G6PC; G6PC2; G6PC3; PPARGC1A; PPARA; TRIB3; NR1H3; NR1H2; PYGB; PYGL; PYGM; SLC2A1; GFPT1; GFPT2; PDPK1; PTEN; OGA; OGT; CRTC2; CREB3; CREB1; CREB3L4; CREB3L2; CREB3L3; CREB3L1; CREB5; PRKAG2; PRKAG3; PRKAA1; PRKAA2; PRKAB1; PRKAB2; PRKAG1; CPT1A; AGT
Non-alcoholic fatty liver disease	INS; IRS1; IRS2; AKT3; AKT1; AKT2; SREBF1; MLXIP; MLXIPL; MLX; PKLR; TNF; NFKB1; RELA; IL6; SOCS3; PIK3CA; PIK3CB; PIK3CD; PIK3R1; PIK3R2; PIK3R3; GSK3A; GSK3B; PPARA; ADIPOQ; PRKAG2; PRKAG3; PRKAA1; PRKAA2; PRKAB1; PRKAB2; PRKAG1; LEP; ADIPOR1; ADIPOR2; CASP8; BID; CYCS; CASP3; CASP7; BAX; FAS; EIF2AK3; EIF2S1; BCL2L11; ATF4; DDIT3; CYP2E1; TGFB1; CXCL8; FASLG; ERN1; TRAF2; MAPK8; MAPK9; MAPK10; FOS; JUN; IKBKB; IL1A; IL1B; MAP3K5; CDC42; RAC1; MAP3K11; ITCH; COX6B2; COX4I1;

	COX5B; COX6A1; COX6A2; COX6B1; COX6C; COX7A1; COX7A2; COX7B; COX7C; COX8A; COX7B2; COX8C; COX1; COX2; COX3; COX4I2; COX7A2L; COX5A; NDUFC2-KCTD14; NDUFA11; NDUFS7; NDUFA1; NDUFA2; NDUFA3; NDUFA4; NDUFA5; NDUFA6; NDUFA7; NDUFA8; NDUFA9; NDUFA10; NDUFAB1; NDUFB1; NDUFB2; NDUFB3; NDUFB4; NDUFB5; NDUFB6; NDUFB7; NDUFB8; NDUFB9; NDUFB10; NDUFC1; NDUFC2; NDUFS1; NDUFS2; NDUFS3; NDUFV1; NDUFS4; NDUFS5; NDUFS6; NDUFS8; NDUFV2; NDUFV3; NDUFA13; NDUFB11; NDUFA12; NDUFA4L2; SDHA; SDHB; SDHC; SDHD; UQCR11; CYC1; UQCRQ; UQCR10; UQCRHL; CYTB; UQCRB; UQCRC1; UQCRC2; UQCRFS1; UQCRH; XBP1; LEPR; NR1H3; RXRA; INSR; CEBPA; IL6R; TNFRSF1A
AGE-RAGE signaling pathway in diabetic complications	CYBB; NOX1; NOX4; AGER; NFKB1; RELA; PRKCB; MAPK8; MAPK9; MAPK10; EGR1; HRAS; KRAS; NRAS; MAPK14; MAPK11; MAPK13; MAPK12; MAPK1; MAPK3; TGFB2; TGFB3; TGFBR1; TGFBR2; SMAD2; SMAD3; SMAD4; EDN1; F3; THBD; IL1A; IL1B; IL6; TNF; VCAM1; ICAM1; CXCL8; JUN; JAK2; STAT5A; STAT5B; CCND1; CDK4; STAT1; STAT3; BAX; BCL2; CASP3; AKT3; AKT1; AKT2; DIAPH1; COL1A1; COL1A2; COL3A1; COL4A1; COL4A2; COL4A3; COL4A4; COL4A5; COL4A6; PIK3CA; PIK3CB; PIK3CD; PIK3R1; PIK3R2; PIK3R3; NOS3; FOXO1; RAC1; CDC42; PIM1; NFATC1; VEGFD; VEGFA; VEGFB; VEGFC; CCL2; SERPINE1; MMP2; SELE; PRKCA; PRKCD; PRKCE; PRKCZ; PLCD3; PLCB1; PLCE1; PLCB2; PLCB3; PLCB4; PLCD1; PLCG1; PLCG2; PLCD4; TGFB1; AGTR1; CDKN1B; FN1; AGT
Cushing syndrome	ITPR1; ITPR2; ITPR3; PLCB1; PLCB2; PLCB3; PLCB4; CACNA1C; CACNA1D; CACNA1F; CACNA1S; GNA11; GNAQ; CACNA1I; CACNA1H; CACNA1G; CREB3; CREB1; ATF2; ATF6B; CREB3L4; ATF4; CREB3L2; CREB3L3; CREB3L1; CREB5; ADCY1; ADCY2; ADCY3; ADCY5; ADCY6; ADCY7; ADCY8; ADCY9; ADCY4; PDE8A; PDE8B; AGTR1; MC2R; GNAS; STAR; HSD3B1; HSD3B2; PRKACA; PRKACB; PRKACG; ORAI1; LDLR; SCARB1; CYP11A1; CYP21A2; MRAP; CYP11B1; KCNK2; NCEH1; CYP17A1; KCNA4; KCNK3; POMC; NR5A1; NR4A1; SP1; PBX1; APC2; APC; AXIN1; AXIN2; DVL1; DVL2; DVL3; FZD10; FZD2; FZD5; FZD3; FZD1; FZD4; FZD6; FZD7; FZD8; FZD9; CTNNB1; GSK3B; LEF1; TCF7; TCF7L2; TCF7L1; EGFR; MAPK1; MAPK3; USP8; CRH; CRHR1; CRHR2; CAMK2A; CAMK2B; CAMK2D; CAMK2G; GNAI1; GNAI2; GNAI3; RASD1; CCND1; CDKN1A; CDK4; CDK6; RB1; E2F1; E2F2; E2F3; CDK2; CCNE1; CCNE2; CDKN1B; CDKN2B; CDKN2A; AHR; ARNT; AIPL1; AIP; CDKN2C; MEN1; WDR5; KMT2A; WDR5B; RBBP5; KMT2D; ASH2L; ARMC5; FH; PDE11A; RAP1A; RAP1B; BRAF; MAP2K1; MAP2K2; WNT16; WNT4; WNT1; WNT2; WNT3; WNT5A; WNT6; WNT7A; WNT7B; WNT8A; WNT8B; WNT10B; WNT11; WNT2B; WNT9A; WNT9B; WNT10A; WNT5B; WNT3A; AGT
Type I diabetes mellitus	ICA1; CD28; IL12A; IL12B; CPE; CD80; CD86; PTPRN; PTPRN2; GAD1; GAD2; HSPD1; IL2; INS; FASLG; GZMB; HLA-DMA; HLA-DMB; HLA-DOA; HLA-DOB; HLA-DPA1; HLA-DPB1; HLA-DQA1; HLA-DQA2; HLA-DQB1; HLA-DRA; HLA-DRB1; HLA-DRB3; HLA-DRB4; HLA-DRB5; HLA-A; HLA-B; HLA-C; HLA-E; HLA-F; HLA-G; FAS; IFNG; PRF1; IL1A; IL1B; TNF; LTA
Maturity onset diabetes of the young	HNF1B; HES1; BHLHA15; ONECUT1; GCK; PDX1; FOXA3; NEUROD1; MAFA;

	HNF4G; NKX2-2; INS; HNF1A; IAPP; PAX4; PAX6; SLC2A2; NKX6-1; PKLR; NR5A2; NEUROG3; HNF4A; HHEX; MNX1; FOXA2; RFX6
Aldosterone-regulated sodium reabsorption	SCNN1A; SCNN1B; SCNN1G; ATP1B4; ATP1A1; ATP1A2; ATP1A3; ATP1A4; ATP1B1; ATP1B2; ATP1B3; FXYD2; SGK1; NR3C2; KRAS; NEDD4L; FXYD4; HSD11B2; INSR; IRS1; PIK3CA; PIK3CB; PIK3CD; PIK3R1; PIK3R2; PIK3R3; PDPK1; INS; IGF1; KCNJ1; SFN; MAPK1; MAPK3; PRKCA; PRKCB; PRKCG; SLC9A3R2
Endocrine and other factor-regulated calcium reabsorption	TRPV5; PTH1R; VDR; SLC8A2; SLC8A1; SLC8A3; ATP2B1; ATP2B2; ATP2B3; ATP2B4; CALB1; BDKRB2; CLTA; CLTB; CLTC; CLTCL1; AP2M1; AP2S1; AP2A1; AP2A2; AP2B1; RAB11A; DNM1; DNM2; DNM3; KL; PRKCA; PRKCB; PRKCG; GNAQ; PLCB1; PLCB2; PLCB3; PLCB4; KLK1; KLK2; ESR1; PRKACA; PRKACB; PRKACG; ADCY6; ADCY9; GNAS; ATP1B4; ATP1A1; ATP1A2; ATP1A3; ATP1A4; ATP1B1; ATP1B2; ATP1B3; FXYD2; PTH
Vasopressin-regulated water reabsorption	AVP; AVPR2; GNAS; ADCY3; ADCY6; ADCY9; PRKACA; PRKACB; PRKACG; CREB3; CREB1; CREB3L4; CREB3L2; CREB3L3; CREB3L1; CREB5; AQP2; AQP4; AQP3; ARHGDIA; ARHGDIB; ARHGDIG; RAB11A; RAB11B; RAB5A; RAB5B; RAB5C; STX4; VAMP2; NSF; DCTN2; DCTN6; DCTN1; DCTN4; DCTN5
Proximal tubule bicarbonate reclamation	SLC9A3; SLC4A4; CA4; CA2; GLS2; GLS; GLUD1; GLUD2; ATP1B4; ATP1A1; ATP1A2; ATP1A3; ATP1A4; ATP1B1; ATP1B2; ATP1B3; FXYD2; AQP1; SLC38A3; SLC25A10; MDH1; PCK1; PCK2
Collecting duct acid secretion	TCIRG1; ATP6V1G3; ATP6V0E2; ATP6V0A2; ATP6V0D2; ATP6V1C2; ATP6V0A4; ATP6V1D; ATP6V1A; ATP6V1B1; ATP6V1B2; ATP6V0C; ATP6V1C1; ATP6V1E1; ATP6V1G2; ATP6V0A1; ATP6V0E1; ATP6V1E2; ATP6V0D1; ATP6V1F; ATP6V1G1; SLC4A1; ATP4A; ATP4B; CA2; SLC12A7; CLCNKB
Salivary secretion	ADRB1; ADRB2; ADRB3; ADRA1D; ADRA1B; ADRA1A; CHRM3; BEST2; SLC12A2; GNAS; ADCY1; ADCY2; ADCY3; ADCY5; ADCY6; ADCY7; ADCY8; ADCY9; ADCY4; PRKACA; PRKACB; PRKACG; VAMP2; GNAQ; PLCB1; PLCB2; PLCB3; PLCB4; ITPR1; ITPR2; ITPR3; ATP2B1; ATP2B2; ATP2B3; ATP2B4; PRKCA; PRKCB; PRKCG; AQP5; RYR3; CALML6; CALML5; CALM1; CALM2; CALM3; CALML3; CALML4; NOS1; GUCY1A2; GUCY1A1; GUCY1B1; PRKG1; PRKG2; BST1; CD38; KCNN4; TRPV6; MUC5B; MUC7; KCNMA1; PRH1; PRH2; PRB1; PRB2; AMY1A; AMY1B; AMY1C; AMY2A; AMY2B; CST1; CST2; CST3; CST4; CST5; HTN1; HTN3; DMBT1; STATH; ATP1B4; ATP1A1; ATP1A2; ATP1A3; ATP1A4; ATP1B1; ATP1B2; ATP1B3; FXYD2; SLC4A2; SLC9A1; LYZ; LYZL1; LPO; CAMP
Gastric acid secretion	CCKBR; CHRM3; ADCY1; ADCY2; ADCY3; ADCY5; ADCY6; ADCY7; ADCY8; ADCY9; ADCY4; PRKACA; PRKACB; PRKACG; GNAQ; PLCB1; PLCB2; PLCB3; PLCB4; ITPR1; ITPR2; ITPR3; HRH2; PRKCA; PRKCB; PRKCG; ATP4A; ATP4B; GAST; GNAS; CFTR; KCNK2; KCNK10; ATP1B4; ATP1A1; ATP1A2; ATP1A3; ATP1A4; ATP1B1; ATP1B2; ATP1B3; SLC9A4; SLC9A1; SLC26A7; SLC4A2; KCNQ1; KCNE2; EZR; CA2; GNAI1; GNAI2; GNAI3; SSTR2; SST; KCNJ1; KCNJ2; KCNJ10; KCNJ15; KCNJ16; ACTB; ACTG1; CALML6; CALML5; CALM1; CALM2; CALM3; CALML3; CALML4; MYLK4; MYLK; MYLK2; MYLK3
Pancreatic secretion	CCKAR; CHRM3; SLC12A2; GNAQ; PLCB1; PLCB2; PLCB3; PLCB4; ITPR1; ITPR2; ITPR3; ATP2B1; ATP2B2; ATP2B3; ATP2B4; PRKCA; PRKCB; PRKCG; KCNQ1;

	KCNMA1; ATP1B4; ATP1A1; ATP1A2; ATP1A3; ATP1A4; ATP1B1; ATP1B2; ATP1B3; FXYD2; SLC9A1; SLC26A3; CFTR; SCTR; SLC4A2; CLCA1; CLCA4; CLCA2; CA2; RYR2; TPCN2; AMY1A; AMY1B; AMY1C; AMY2A; AMY2B; SLC4A4; PRSS1; PRSS2; PRSS3; CPA1; CPA2; CPA3; CPB1; CPB2; CELA3A; CELA3B; CELA2B; CELA2A; PNLIPRP1; PNLIPRP2; CEL; PLA2G2D; PLA2G2E; PLA2G2C; PLA2G3; PLA2G1B; PLA2G2A; PLA2G5; PLA2G2F; PLA2G12A; PLA2G10; PLA2G12B; PNLIP; ATP2A1; ATP2A2; ATP2A3; TRPC1; CTRB1; CTRB2; BST1; CD38; GNAS; ADCY1; ADCY2; ADCY3; ADCY5; ADCY6; ADCY7; ADCY8; ADCY9; ADCY4; CTRL; RAB8A; RAB27B; RAB11A; RAB3D; RAP1A; RAP1B; RHOA; RAC1; SCT; CCK
Protein digestion and absorption	SLC1A1; SLC7A9; SLC7A8; SLC38A2; SLC7A7; SLC15A1; SLC6A19; SLC1A5; PRSS1; PRSS2; PRSS3; SLC3A1; ATP1B4; ATP1A1; ATP1A2; ATP1A3; ATP1A4; ATP1B1; ATP1B2; ATP1B3; FXYD2; SLC3A2; CTRB1; CTRB2; CTRL; CELA3A; CELA3B; CELA2B; CELA2A; CPA1; CPA2; CPA3; CPB1; CPB2; PGA5; PGA3; PGA4; SLC9A3; KCNE3; KCNJ13; KCNN4; KCNQ1; KCNK5; SLC36A4; SLC36A2; SLC36A1; SLC36A3; SLC8A2; SLC8A1; SLC8A3; DPP4; MEP1A; MEP1B; MME; PRCP; ACE2; XPNPEP2; ELN; COL1A1; COL1A2; COL2A1; COL3A1; COL4A1; COL4A2; COL4A3; COL4A4; COL4A5; COL4A6; COL5A1; COL5A2; COL6A1; COL6A2; COL6A3; COL7A1; COL8A1; COL8A2; COL9A1; COL9A2; COL9A3; COL10A1; COL11A1; COL11A2; COL12A1; COL13A1; COL15A1; COL16A1; COL17A1; COL19A1; COL6A6; COL26A1; COL22A1; COL24A1; COL6A5; COL28A1; COL5A3; COL20A1; COL14A1; COL18A1; COL21A1; COL25A1; COL27A1; COL23A1; SLC16A10
Bile secretion	AQP9; SLC9A1; SLC4A2; ATP1B4; ATP1A1; ATP1A2; ATP1A3; ATP1A4; ATP1B1; ATP1B2; ATP1B3; FXYD2; CFTR; SCTR; CA2; SLC4A4; GNAS; ADCY1; ADCY2; ADCY3; ADCY5; ADCY6; ADCY7; ADCY8; ADCY9; ADCY4; PRKACA; PRKACB; PRKACG; SLCO1B1; SLCO1B3; SLCO1B7; SLCO1A2; EPHX1; SLC10A1; ABCB11; ABCC2; ABCC4; ABCC3; CYP7A1; NR1H4; ABCG5; ABCG8; SLC22A7; SLC22A8; SLC22A1; SLC10A2; ABCB4; ABCB1; SLC5A1; SLC2A1; AQP8; NR0B2; CYP3A4; UGT2B11; UGT2A1; UGT2B28; UGT1A10; UGT1A8; UGT1A7; UGT1A6; UGT1A5; UGT1A9; UGT1A4; UGT1A1; UGT1A3; UGT2A2; UGT2B4; UGT2B7; UGT2B10; UGT2B15; UGT2B17; UGT2A3; SULT2A1; SLC27A5; BAAT; SLC9A3; SLC51A; SLC51B; AQP4; AQP1; ABCG2; SLC4A5; KCNN2; RXRA; SCARB1; LDLR; HMGCR; NCEH1; SCT
Vitamin digestion and absorption	APOB; APOA4; APOA1; SCARB1; CUBN; CBLIF; LMBRD1; ABCC1; TCN2; MMACHC; RBP2; LRAT; SLC5A6; SLC23A1; SLC19A1; SLC19A2; SLC19A3; BTD; FOLH1; SLC52A3; AWAT2; SLC46A1; PNLIP; PLB1
Mineral absorption	S100G; ATP2B1; ATP2B2; ATP2B3; ATP2B4; SLC8A2; SLC8A1; SLC8A3; VDR; TRPV6; SLC46A1; CYBRD1; SLC11A2; SLC40A1; FTH1; FTL; HMOX1; HMOX2; HEPH; TF; SLC39A4; SLC31A1; SLC30A1; ATP7A; ATP7B; MT1A; MT1B; MT1E; MT1F; MT1G; MT1H; MT1M; MT1X; MT2A; MT1HL1; STEAP2; STEAP1; ATOX1; SLC26A6; SLC9A3; ATP1B4; ATP1A1; ATP1A2; ATP1A3; ATP1A4; ATP1B1; ATP1B2; ATP1B3; FXYD2; SLC26A3; SLC5A1; SLC6A19; TRPM6; TRPM7; SLC34A2; SLC34A3; SLC34A1; SLC26A9; CLCN2

Cholesterol metabolism	APOA1; APOB; APOC1; APOC2; APOC3; APOE; LPA; APOA2; APOA4; ANGPTL3; ANGPTL4; ANGPTL8; APOH; LPL; ABCA1; NCEH1; SOAT1; SOAT2; LDLR; CD36; LCAT; PLTP; CETP; LIPG; LIPC; SCARB1; SORT1; PCSK9; LDLRAP1; MYLIP; LIPA; STARD3; NPC1; VAPB; VAPA; OSBPL5; NPC2; STAR; VDAC1; VDAC2; VDAC3; TSPO; CYP27A1; CYP7A1; ABCG5; ABCG8; ABCB11; LRP1; LRP2; LRPAP1
Alzheimer disease	PSENEN; PSEN1; PSEN2; NCSTN; APH1A; APH1B; BACE1; BACE2; ADAM10; ADAM17; TNF; APP; CASP9; CASP12; CASP3; CYCS; APAF1; NDUFC2-KCTD14; NDUFA11; NDUFS7; ND1; ND2; ND3; ND4; ND4L; ND5; ND6; NDUFA1; NDUFA2; NDUFA3; NDUFA4; NDUFA5; NDUFA6; NDUFA7; NDUFA8; NDUFA9; NDUFA10; NDUFAB1; NDUFB1; NDUFB2; NDUFB3; NDUFB4; NDUFB5; NDUFB6; NDUFB7; NDUFB8; NDUFB9; NDUFB10; NDUFC1; NDUFC2; NDUFS1; NDUFS2; NDUFS3; NDUFV1; NDUFS4; NDUFS5; NDUFS6; NDUFS8; NDUFV2; NDUFV3; NDUFA13; NDUFB11; NDUFA12; NDUFA4L2; SDHA; SDHB; SDHC; SDHD; UQCR11; CYC1; UQCRQ; UQCR10; UQCRHL; CYTB; UQCRB; UQCRC1; UQCRC2; UQCRFS1; UQCRH; ATP5PD; ATP6; ATP8; ATP5F1A; ATP5F1B; ATP5F1C; ATP5F1D; ATP5F1E; ATP5PB; ATP5MC1; ATP5MC2; ATP5MC3; ATP5PF; ATP5PO; COX6B2; COX4I1; COX5B; COX6A1; COX6A2; COX6B1; COX6C; COX7A1; COX7A2; COX7B; COX7C; COX8A; COX7B2; COX8C; COX1; COX2; COX3; COX4I2; COX7A2L; COX5A; RYR3; ITPR1; ITPR2; ITPR3; ATP2A1; ATP2A2; ATP2A3; CHRM1; CHRM3; CHRM5; GRM5; GNAQ; PLCB1; PLCB2; PLCB3; PLCB4; GRIN1; GRIN2A; GRIN2B; GRIN2C; GRIN2D; CACNA1C; CACNA1D; CACNA1F; CACNA1S; HSD17B10; CAPN1; CAPN2; CDK5R1; CDK5; MAPT; FAS; TNFRSF1A; FADD; CASP8; BID; MAPK1; MAPK3; NOS1; EIF2AK3; ERN1; ATF6; APOE; CALML6; CALML5; CALM1; CALM2; CALM3; CALML3; CALML4; PPP3CA; PPP3CB; PPP3CC; PPP3R1; PPP3R2; BAD; IDE; MME; APBB1; LRP1; LPL; GAPDH; NAE1; CASP7; GSK3B; SNCA; IL1B; RTN3; RTN4; TRAF2; MAP3K5; MAPK8; MAPK9; MAPK10; MTOR; ULK1; ULK2; ATG13; RB1CC1; ATG101; ATG2A; ATG2B; WIPI2; WIPI1; AMBRA1; BECN2; BECN1; PIK3R4; PIK3C3; ATG14; MAP2K7; EIF2S1; ATF4; KLC3; KIF5A; KIF5B; KIF5C; KLC1; KLC2; KLC4; TUBA1B; TUBA3E; TUBA3D; TUBA8; TUBA4A; TUBA3C; TUBA1A; TUBAL3; TUBA1C; TUBB3; TUBB4A; TUBB4B; TUBB; TUBB8; TUBB2B; TUBB2A; TUBB1; TUBB6; NRBF2; CSNK1A1L; CSNK1A1; AXIN1; AXIN2; APC2; APC; CTNNB1; FRAT1; FRAT2; DVL1; DVL2; DVL3; CSNK2A1; CSNK2A2; CSNK2B; CSNK2A3; TPTEP2-CSNK1E; CSNK1E; LRP6; LRP5; FZD10; FZD2; FZD5; FZD3; FZD1; FZD4; FZD6; FZD7; FZD8; FZD9; WNT16; WNT4; WNT1; WNT2; WNT3; WNT5A; WNT6; WNT7A; WNT7B; WNT8A; WNT8B; WNT10B; WNT11; WNT2B; WNT9A; WNT9B; WNT10A; WNT5B; WNT3A; DKK1; DKK4; DKK2; AGER; HRAS; KRAS; NRAS; CYBB; NOX1; NOX4; NFKB1; RELA; IL6; IL1A; NOS2; PTGS2; CSF1; INS; INSR; AKT3; AKT1; AKT2; PIK3CA; PIK3CB; PIK3CD; PIK3R1; PIK3R2; PIK3R3; IRS1; IRS4; IRS2; EIF2AK2; GPR83; CHUK; IKBKB; ARAF; RAF1; BRAF; MAP2K1; MAP2K2; MCU; PPIF; SLC25A4; SLC25A5; SLC25A6; VDAC1; VDAC2; VDAC3; SLC25A31; PPID; CHRNA7; XBP1; DDIT3; PSMA8; PSMA1; PSMA2; PSMA3; PSMA4; PSMA5; PSMA6; PSMA7; PSMB1; PSMB2; PSMB3; PSMB4; PSMB5;

	PSMB6; PSMB7; PSMD14; ADRM1; PSMC1; PSMC2; PSMC3; PSMC4; PSMC5; PSMC6; PSMD1; PSMD2; PSMD3; PSMD4; PSMD7; PSMD8; PSMD9; PSMD11; PSMD12; PSMD13; SEM1; PSMD6
Parkinson disease	LRRK2; UBA1; UBA7; RPS27A; UBA52; UBB; UBC; UBE2L3; UBE2L6; PRKN; SEPTIN5; SNCAIP; GPR37; PARK7; PINK1; SNCA; UCHL1; CYCS; NDUFC2-KCTD14; NDUFA11; NDUFS7; ND1; ND2; ND3; ND4; ND4L; ND5; ND6; NDUFA1; NDUFA2; NDUFA3; NDUFA4; NDUFA5; NDUFA6; NDUFA7; NDUFA8; NDUFA9; NDUFA10; NDUFAB1; NDUFB1; NDUFB2; NDUFB3; NDUFB4; NDUFB5; NDUFB6; NDUFB7; NDUFB8; NDUFB9; NDUFB10; NDUFC1; NDUFC2; NDUFS1; NDUFS2; NDUFS3; NDUFV1; NDUFS4; NDUFS5; NDUFS6; NDUFS8; NDUFV2; NDUFV3; NDUFA13; NDUFB11; NDUFA12; NDUFA4L2; SDHA; SDHB; SDHC; SDHD; UQCR11; CYC1; UQCRQ; UQCR10; UQCRHL; CYTB; UQCRB; UQCRC1; UQCRC2; UQCRFS1; UQCRH; COX6B2; COX4I1; COX5B; COX6A1; COX6A2; COX6B1; COX6C; COX7A1; COX7A2; COX7B; COX7C; COX8A; COX7B2; COX8C; COX1; COX2; COX3; COX4I2; COX7A2L; COX5A; ATP5PD; ATP6; ATP8; ATP5F1A; ATP5F1B; ATP5F1C; ATP5F1D; ATP5F1E; ATP5PB; ATP5MC1; ATP5MC2; ATP5MC3; ATP5PF; ATP5PO; HTRA2; PPIF; SLC25A4; SLC25A5; SLC25A6; VDAC1; VDAC2; VDAC3; SLC25A31; UBE2J2; UBE2J1; UBE2G1; UBE2G2; TH; SLC6A3; CASP9; CASP3; APAF1; SLC18A1; SLC18A2; ADORA2A; DRD2; DRD1; GNAL; GNAS; GNAI1; GNAI2; GNAI3; ADCY5; PRKACA; PRKACB; PRKACG; EIF2AK3; ERN1; ATF6; EIF2S1; ATF4; XBP1; DDIT3; HSPA5; PSMA8; PSMA1; PSMA2; PSMA3; PSMA4; PSMA5; PSMA6; PSMA7; PSMB1; PSMB2; PSMB3; PSMB4; PSMB5; PSMB6; PSMB7; PSMD14; ADRM1; PSMC1; PSMC2; PSMC3; PSMC4; PSMC5; PSMC6; PSMD1; PSMD2; PSMD3; PSMD4; PSMD7; PSMD8; PSMD9; PSMD11; PSMD12; PSMD13; SEM1; PSMD6; ITPR1; ITPR2; ITPR3; PLCG1; CALML6; CALML5; CALM1; CALM2; CALM3; CALML3; CALML4; CAMK2A; CAMK2B; CAMK2D; CAMK2G; MAPT; RYR3; KLC3; KIF5A; KIF5B; KIF5C; KLC1; KLC2; KLC4; TUBA1B; TUBA3E; TUBA3D; TUBA8; TUBA4A; TUBA3C; TUBA1A; TUBAL3; TUBA1C; TUBB3; TUBB4A; TUBB4B; TUBB; TUBB8; TUBB2B; TUBB2A; TUBB1; TUBB6; BAX; BCL2L1; TRAP1; TP53; DAXX; MAP3K5; MAPK8; MAPK9; MAPK10; NFE2L2; DUSP1; TXN2; TXN; MAOA; MAOB; MCU; MFN1; MFN2
Amyotrophic lateral sclerosis	SOD1; SLC1A2; GRIA1; GRIA2; GRIN1; GRIN2A; GRIN2B; GRIN2C; GRIN2D; TOMM40; TOMM40L; CYCS; TNF; CASP1; CASP3; BAX; BAD; BCL2; BCL2L1; APAF1; CASP9; BID; TNFRSF1A; TNFRSF1B; DAXX; MAP3K5; MAP2K3; MAP2K6; MAPK14; MAPK11; MAPK13; MAPK12; NEFL; NEFM; NEFH; PRPH; GPX6; GPX1; GPX2; GPX3; GPX5; GPX7; GPX8; CAT; NOS1; CCS; ALS2; DERL1; RAC1; RAB5A; PPP3CA; PPP3CB; PPP3CC; PPP3R1; PPP3R2; TP53; PINK1; NDUFC2-KCTD14; NDUFA11; NDUFS7; ND1; ND2; ND3; ND4; ND4L; ND5; ND6; NDUFA1; NDUFA2; NDUFA3; NDUFA4; NDUFA5; NDUFA6; NDUFA7; NDUFA8; NDUFA9; NDUFA10; NDUFAB1; NDUFB1; NDUFB2; NDUFB3; NDUFB4; NDUFB5; NDUFB6; NDUFB7; NDUFB8; NDUFB9; NDUFB10; NDUFC1; NDUFC2; NDUFS1; NDUFS2; NDUFS3; NDUFV1; NDUFS4; NDUFS5; NDUFS6; NDUFS8; NDUFV2; NDUFV3; NDUFA13; NDUFB11; NDUFA12; NDUFA4L2; SDHA; SDHB;

	SDHC; SDHD; UQCR11; CYC1; UQCRQ; UQCR10; UQCRHL; CYTB; UQCRB; UQCRC1; UQCRC2; UQCRFS1; UQCRH; COX6B2; COX4I1; COX5B; COX6A1; COX6A2; COX6B1; COX6C; COX7A1; COX7A2; COX7B; COX7C; COX8A; COX7B2; COX8C; COX1; COX2; COX3; COX4I2; COX7A2L; COX5A; ATP5PD; ATP6; ATP8; ATP5F1A; ATP5F1B; ATP5F1C; ATP5F1D; ATP5F1E; ATP5PB; ATP5MC1; ATP5MC2; ATP5MC3; ATP5PF; ATP5PO; EIF2AK3; ERN1; ATF6; EIF2S1; ATF4; XBP1; DDIT3; HSPA5; KLC3; KIF5A; KIF5B; KIF5C; KLC1; KLC2; KLC4; TUBA1B; TUBA3E; TUBA3D; TUBA8; TUBA4A; TUBA3C; TUBA1A; TUBAL3; TUBA1C; TUBB3; TUBB4A; TUBB4B; TUBB; TUBB8; TUBB2B; TUBB2A; TUBB1; TUBB6; PRKN; TRAF2; MTOR; ULK1; ULK2; ATG13; RB1CC1; ATG101; ATG2A; ATG2B; WIPI2; WIPI1; AMBRA1; BECN2; BECN1; PIK3R4; NRBF2; PIK3C3; ATG14; PSMA8; PSMA1; PSMA2; PSMA3; PSMA4; PSMA5; PSMA6; PSMA7; PSMB1; PSMB2; PSMB3; PSMB4; PSMB5; PSMB6; PSMB7; PSMD14; ADRM1; PSMC1; PSMC2; PSMC3; PSMC4; PSMC5; PSMC6; PSMD1; PSMD2; PSMD3; PSMD4; PSMD7; PSMD8; PSMD9; PSMD11; PSMD12; PSMD13; SEM1; PSMD6; C9orf72; RAB1A; WDR41; SMCR8; RAB39B; RAB8A; SQSTM1; TBK1; TANK; OPTN; TARDBP; HAP1; ACTR1B; ACTR1A; DCTN2; DCTN6; DCTN3; DCTN1; DCTN4; ACTR10; DCTN5; DNAL4; DNAH14; DNAH2; DNAH5; DNAH6; DNAH8; DNAH9; DNAH10; DNAH12; DNAH1; DNAI1; DNAH3; DNAH7; DNAI2; DNALI1; DNAL1; DNAH17; DNAH11; GABARAP; GABARAPL2; GABARAPL1; VDAC1; HDAC6; UBQLN2; UBQLN1; UBQLN3; UBQLN4; VAPB; VCP; HNRNPA1L2; HNRNPA3; HNRNPA1; HNRNPA2B1; FUS; ATXN2L; ATXN2; CHCHD10; ITPR3; SIGMAR1; GLE1; POM121C; NUP50; NUP35; NUP205; NUP210; NUP160; NUP188; NUP62; NUP43; NUP88; NUP98; NUP54; NDC1; NUP133; NUP107; RANBP2; SEC13; TPR; NUP37; NUP85; NUP214; SEH1L; RAE1; NUP210L; POM121L2; NUP155; NUP93; NUP58; POM121; NUP153; MATR3; SETX; ANG; FIG4; CHMP2B; PFN3; PFN4; PFN1; PFN2; ACTB; ACTG1; SPG11; NRG3; NRG4; NRG1; NRG2; ERBB4; ANXA7; ANXA11; MCU; NOS2; CASP12; NCBP1; ALYREF; SRSF3; SRSF7; NXF1; NXF5; NXF3; NXF2; NXF2B
Huntington disease	NDUFC2-KCTD14; NDUFA11; NDUFS7; ND1; ND2; ND3; ND4; ND4L; ND5; ND6; NDUFA1; NDUFA2; NDUFA3; NDUFA4; NDUFA5; NDUFA6; NDUFA7; NDUFA8; NDUFA9; NDUFA10; NDUFAB1; NDUFB1; NDUFB2; NDUFB3; NDUFB4; NDUFB5; NDUFB6; NDUFB7; NDUFB8; NDUFB9; NDUFB10; NDUFC1; NDUFC2; NDUFS1; NDUFS2; NDUFS3; NDUFV1; NDUFS4; NDUFS5; NDUFS6; NDUFS8; NDUFV2; NDUFV3; NDUFA13; NDUFB11; NDUFA12; NDUFA4L2; SDHA; SDHB; SDHC; SDHD; UQCR11; CYC1; UQCRQ; UQCR10; UQCRHL; CYTB; UQCRB; UQCRC1; UQCRC2; UQCRFS1; UQCRH; COX6B2; COX4I1; COX5B; COX6A1; COX6A2; COX6B1; COX6C; COX7A1; COX7A2; COX7B; COX7C; COX8A; COX7B2; COX8C; COX1; COX2; COX3; COX4I2; COX7A2L; COX5A; ATP5PD; ATP6; ATP8; ATP5F1A; ATP5F1B; ATP5F1C; ATP5F1D; ATP5F1E; ATP5PB; ATP5MC1; ATP5MC2; ATP5MC3; ATP5PF; ATP5PO; HTT; REST; RCOR1; SIN3A; HDAC1; HDAC2; BDNF; HAP1; CREBBP; EP300; POLR2J2; POLR2A; POLR2B; POLR2C; POLR2D; POLR2E; POLR2F; POLR2G; POLR2H; POLR2I; POLR2J; POLR2K; POLR2L; POLR2J3; GRIN1; GRIN2B; DLG4; GNAQ; GRM5; PLCB1;

	PLCB2; PLCB3; PLCB4; ITPR1; PPIF; SLC25A4; SLC25A5; SLC25A6; VDAC1; VDAC2; VDAC3; SLC25A31; CYCS; TAF4; TAF4B; TFAM; NRF1; SOD2; GPX6; GPX1; GPX2; GPX3; GPX5; GPX7; GPX8; UCP1; SOD1; TBPL2; TBP; TBPL1; TP53; CLTA; CLTB; CLTC; CLTCL1; AP2M1; AP2S1; AP2A1; AP2A2; AP2B1; HIP1; IFT57; CASP8; CASP3; ACTR1B; ACTR1A; DCTN2; DCTN6; DCTN3; DCTN1; DCTN4; ACTR10; DCTN5; DNAL4; DNAH14; DNAH2; DNAH5; DNAH6; DNAH8; DNAH9; DNAH10; DNAH12; DNAH1; DNAI1; DNAH3; DNAH7; DNAI2; DNALI1; DNAL1; DNAH17; DNAH11; TGM2; APAF1; CASP9; CREB3; CREB1; CREB3L4; CREB3L2; CREB3L3; CREB3L1; CREB5; PPARGC1A; PPARG; BAX; BBC3; SP1; ERN1; TRAF2; MAP3K5; MAPK8; MAPK9; MAPK10; MTOR; ULK1; ULK2; ATG13; RB1CC1; ATG101; ATG2A; ATG2B; WIPI2; WIPI1; AMBRA1; BECN2; BECN1; PIK3R4; NRBF2; PIK3C3; ATG14; MAP2K7; MAP3K10; KLC3; KIF5A; KIF5B; KIF5C; KLC1; KLC2; KLC4; GRIA1; GRIA2; GRIA3; GRIA4; KCNJ10; SLC1A2; SLC1A3; CACNA1B; STX1A; TUBA1B; TUBA3E; TUBA3D; TUBA8; TUBA4A; TUBA3C; TUBA1A; TUBAL3; TUBA1C; TUBB3; TUBB4A; TUBB4B; TUBB; TUBB8; TUBB2B; TUBB2A; TUBB1; TUBB6; PSMA8; PSMA1; PSMA2; PSMA3; PSMA4; PSMA5; PSMA6; PSMA7; PSMB1; PSMB2; PSMB3; PSMB4; PSMB5; PSMB6; PSMB7
Spinocerebellar ataxia	PLCB1; PLCB2; PLCB3; PLCB4; GNAQ; PRKCA; PRKCB; PRKCG; GRIA1; GRIA2; GRIA3; GRM1; RYR1; CACNA1A; ITPR1; ITPR2; ITPR3; FGF14; ATXN1L; ATXN1; KAT5; RORA; TRPC3; ATXN2L; ATXN3; ATXN2; ATXN3L; SPTBN2; ERN1; TRAF2; MAP3K5; MAPK8; MAPK9; MAPK10; MTOR; ULK1; ULK2; ATG13; RB1CC1; ATG101; ATG2A; ATG2B; WIPI2; WIPI1; AMBRA1; BECN2; BECN1; PIK3R4; NRBF2; PIK3C3; ATG14; TWNK; ATXN8OS; ATXN10; BEAN1; AFG3L2; OMA1; OPA1; KCNC3; CIC; ATP2A1; ATP2A2; ATP2A3; PUM2; PUM1; KCND3; GRIN3A; GRIN3B; GRIN1; GRIN2A; GRIN2B; GRIN2C; GRIN2D; PDYN; NOP56; VLDLR; DAB1; PIK3CA; PIK3CB; PIK3CD; PIK3R1; PIK3R2; PIK3R3; AKT3; AKT1; AKT2; RELN; SLC1A6; TBPL2; TBP; TBPL1; RBPJL; RBPJ; SP1; GTF2B; NFYA; XBP1; MYOD1; PSMA8; PSMA1; PSMA2; PSMA3; PSMA4; PSMA5; PSMA6; PSMA7; PSMB1; PSMB2; PSMB3; PSMB4; PSMB5; PSMB6; PSMB7; PSMD14; ADRM1; PSMC1; PSMC2; PSMC3; PSMC4; PSMC5; PSMC6; PSMD1; PSMD2; PSMD3; PSMD4; PSMD7; PSMD8; PSMD9; PSMD11; PSMD12; PSMD13; SEM1; PSMD6; CYCS; MCU
Prion disease	PRNP; NCAM1; NCAM2; LAMC1; STIP1; PRKACA; PRKACB; PRKACG; SOD1; BAX; C1QA; C1QB; C1QC; FYN; MAPK1; MAPK3; EGR1; CCL5; IL1A; IL1B; IL6; NOTCH1; CASP12; HSPA1A; HSPA1B; HSPA1L; HSPA2; HSPA6; HSPA8; C5; C6; C7; C8A; C8B; C8G; C9; HSPA5; CASP9; CASP3; CYCS; APAF1; NDUFC2-KCTD14; NDUFA11; NDUFS7; ND1; ND2; ND3; ND4; ND4L; ND5; ND6; NDUFA1; NDUFA2; NDUFA3; NDUFA4; NDUFA5; NDUFA6; NDUFA7; NDUFA8; NDUFA9; NDUFA10; NDUFAB1; NDUFB1; NDUFB2; NDUFB3; NDUFB4; NDUFB5; NDUFB6; NDUFB7; NDUFB8; NDUFB9; NDUFB10; NDUFC1; NDUFC2; NDUFS1; NDUFS2; NDUFS3; NDUFV1; NDUFS4; NDUFS5; NDUFS6; NDUFS8; NDUFV2; NDUFV3; NDUFA13; NDUFB11; NDUFA12; NDUFA4L2; SDHA; SDHB; SDHC; SDHD; UQCR11; CYC1; UQCRQ; UQCR10; UQCRHL; CYTB; UQCRB; UQCRC1; UQCRC2; UQCRFS1;

	UQCRH; ATP5PD; ATP6; ATP8; ATP5F1A; ATP5F1B; ATP5F1C; ATP5F1D; ATP5F1E; ATP5PB; ATP5MC1; ATP5MC2; ATP5MC3; ATP5PF; ATP5PO; COX6B2; COX4I1; COX5B; COX6A1; COX6A2; COX6B1; COX6C; COX7A1; COX7A2; COX7B; COX7C; COX8A; COX7B2; COX8C; COX1; COX2; COX3; COX4I2; COX7A2L; COX5A; RYR1; RYR2; RYR3; ITPR1; ITPR2; ITPR3; GRIN3A; GRIN3B; GRIN1; GRIN2A; GRIN2B; GRIN2C; GRIN2D; EIF2AK3; BAD; EIF2S1; ATF4; MCU; PPIF; SLC25A4; SLC25A5; SLC25A6; VDAC1; VDAC2; VDAC3; SLC25A31; DDIT3; PSMA8; PSMA1; PSMA2; PSMA3; PSMA4; PSMA5; PSMA6; PSMA7; PSMB1; PSMB2; PSMB3; PSMB4; PSMB5; PSMB6; PSMB7; PSMD14; ADRM1; PSMC1; PSMC2; PSMC3; PSMC4; PSMC5; PSMC6; PSMD1; PSMD2; PSMD3; PSMD4; PSMD7; PSMD8; PSMD9; PSMD11; PSMD12; PSMD13; SEM1; PSMD6; KLC3; KIF5A; KIF5B; KIF5C; KLC1; KLC2; KLC4; TUBA1B; TUBA3E; TUBA3D; TUBA8; TUBA4A; TUBA3C; TUBA1A; TUBAL3; TUBA1C; TUBB3; TUBB4A; TUBB4B; TUBB; TUBB8; TUBB2B; TUBB2A; TUBB1; TUBB6; GSK3B; PPP3CA; PPP3CB; PPP3CC; PPP3R1; PPP3R2; CACNA1B; CACNA1C; CACNA1D; CACNA1F; CACNA1S; CSNK2A1; CSNK2A2; CSNK2B; CSNK2A3; TNF; CAV1; CAV2; CAV3; PIK3CA; PIK3CB; PIK3CD; PIK3R1; PIK3R2; PIK3R3; PRKCD; CREB3; CREB1; ATF2; ATF6B; CREB3L4; CREB3L2; CREB3L3; CREB3L1; CREB5; CYBB; CYBA; NCF1; NCF2; RAC1; RAC2; NCF4; MAPK14; MAPK11; MAPK13; MAPK12
Cocaine addiction	SLC18A1; SLC18A2; GRM2; GRM3; DRD2; DRD1; SLC6A3; MAOA; MAOB; TH; DDC; GNAS; ADCY5; PRKACA; PRKACB; PRKACG; GNAI1; GNAI2; GNAI3; GPSM1; RGS9; JUN; FOSB; CDK5; NFKB1; RELA; CREB3; CREB1; ATF2; ATF6B; CREB3L4; ATF4; CREB3L2; CREB3L3; CREB3L1; CREB5; PDYN; BDNF; GRIA2; PPP1R1B; GRIN3A; GRIN3B; GRIN1; GRIN2A; GRIN2B; GRIN2C; GRIN2D; DLG4; CDK5R1
Amphetamine addiction	SLC18A1; SLC18A2; CACNA1C; CACNA1D; GRIN3A; GRIN3B; GRIN1; GRIN2A; GRIN2B; GRIN2C; GRIN2D; GRIA1; GRIA2; GRIA3; GRIA4; DRD1; SLC6A3; MAOA; MAOB; TH; DDC; CALML6; CALML5; CALM1; CALM2; CALM3; CALML3; CALML4; CAMK4; CAMK2A; CAMK2B; CAMK2D; CAMK2G; PPP1R1B; PRKACA; PRKACB; PRKACG; GNAS; ADCY5; CREB3; CREB1; ATF2; ATF6B; CREB3L4; ATF4; CREB3L2; CREB3L3; CREB3L1; CREB5; PPP1CA; PPP1CB; PPP1CC; PPP3CA; PPP3CB; PPP3CC; PPP3R1; PPP3R2; STX1A; PRKCA; PRKCB; PRKCG; PDYN; ARC; FOS; FOSB; HDAC1; HDAC2; SIRT1; JUN
Morphine addiction	OPRM1; ADCY1; ADCY2; ADCY3; ADCY5; ADCY6; ADCY7; ADCY8; ADCY9; ADCY4; PRKACA; PRKACB; PRKACG; GNB5; GNAI1; GNAI2; GNAI3; GNAO1; GNB1; GNB2; GNB3; GNG3; GNG4; GNG5; GNG7; GNG10; GNG11; GNGT1; GNGT2; GNG13; GNG2; GNG12; GNB4; GNG8; PDE10A; PDE7B; PDE11A; PDE1A; PDE1C; PDE2A; PDE3A; PDE3B; PDE4A; PDE4B; PDE4C; PDE4D; PDE7A; PDE8A; PDE1B; PDE8B; ADORA1; GABRR3; GABRA1; GABRA2; GABRA3; GABRA4; GABRA5; GABRA6; GABRB1; GABRB2; GABRB3; GABRD; GABRE; GABRG1; GABRG2; GABRG3; GABRP; GABRR1; GABRR2; GABRQ; KCNJ3; KCNJ5; KCNJ6; KCNJ9; CACNA1A; CACNA1B; GNAS; PRKCA; PRKCB; PRKCG; SLC32A1; DRD1; GABBR1; GABBR2; GRK2; GRK3; GRK4; GRK5; GRK6; ARRB1; ARRB2
Nicotine addiction	CHRNA6; CACNA1A; CACNA1B; CHRNA4; CHRNB2; CHRNA7; GABRR3;

	GABRA1; GABRA2; GABRA3; GABRA4; GABRA5; GABRA6; GABRB1; GABRB2; GABRB3; GABRD; GABRE; GABRG1; GABRG2; GABRG3; GABRP; GABRR1; GABRR2; GABRQ; GRIN3A; GRIN3B; GRIN1; GRIN2A; GRIN2B; GRIN2C; GRIN2D; GRIA1; GRIA2; GRIA3; GRIA4; SLC32A1; SLC17A8; SLC17A7; SLC17A6
Alcoholism	SLC18A1; SLC18A2; DRD1; SLC6A3; GRIN3A; GRIN3B; GRIN1; GRIN2A; GRIN2B; GRIN2C; GRIN2D; MAOA; MAOB; TH; DDC; PPP1CA; PPP1CB; PPP1CC; GNAS; PPP1R1B; ADCY5; PRKACA; PRKACB; PRKACG; CREB3; CREB1; ATF2; ATF6B; CREB3L4; ATF4; CREB3L2; CREB3L3; CREB3L1; CREB5; CAMK4; HDAC6; HDAC5; HDAC1; HDAC2; HDAC7; HDAC8; HDAC11; HDAC10; HDAC3; HDAC9; HDAC4; CRH; NPY; BDNF; CALML6; CALML5; CALM1; CALM2; CALM3; CALML3; CALML4; CAMKK2; CAMKK1; DRD2; GNB5; GNAI1; GNAI2; GNAI3; GNAO1; GNB1; GNB2; GNB3; GNG3; GNG4; GNG5; GNG7; GNG10; GNG11; GNGT1; GNGT2; GNG13; GNG2; GNG12; GNB4; GNG8; ADORA2A; ADORA2B; SLC29A1; SLC29A2; SLC29A3; HRAS; KRAS; NRAS; ARAF; RAF1; BRAF; MAP2K1; MAPK1; MAPK3; FOSB; HAT1; H2AC1; H2AC8; H2AC7; H2AX; H2AZ1; H2AC21; H2AB2; H2AB1; MACROH2A2; H2AJ; H2AC19; H2AC13; H2AC15; H2AC14; H2AC16; H2AC6; H2AC4; H2AC17; H2AC18; H2AC20; H2AB3; H2AC12; H2AC11; H2AW; H2AZ2; MACROH2A1; LOC102724334; H2BE1; H2BU1; H2BW1; H2BC1; H2BW2; H2BC5; H2BC3; H2BC18; H2BS1; H2BC8; H2BC13; H2BC15; H2BC14; H2BC7; H2BC6; H2BC9; H2BC10; H2BC4; H2BC17; H2BC21; H2BC12; H2BC11; H4-16; H4C15; H4C9; H4C1; H4C4; H4C6; H4C12; H4C11; H4C3; H4C8; H4C2; H4C5; H4C13; H4C7; H4C14; H3C14; H3-3A; H3-3B; H3C15; H3-5; H3C13; H3-4; H3C1; H3C4; H3C3; H3C6; H3C11; H3C8; H3C12; H3C10; H3C2; H3C7; NTRK2; SHC2; SHC4; SHC3; SHC1; GRB2; SOS1; SOS2; PDYN; PKIA
Epithelial cell signaling in Helicobacter pylori infection	MAPK14; MAPK11; MAPK13; MAPK12; JUN; MAPK8; MAPK9; MAPK10; MAP2K4; CHUK; IKBKG; IKBKB; NFKBIA; TCIRG1; ATP6V1G3; ATP6V0E2; ATP6V0A2; ATP6V0D2; ATP6V1C2; ATP6V0A4; ATP6V1D; ATP6V1H; ATP6V1A; ATP6V1B1; ATP6V1B2; ATP6V0C; ATP6V1C1; ATP6V1E1; ATP6V0B; ATP6V1G2; ATP6V0A1; ATP6AP1; ATP6V0E1; ATP6V1E2; ATP6V0D1; ATP6V1F; ATP6V1G1; CASP3; PAK1; NFKB1; RELA; CCL5; CXCL1; CXCL2; CXCL3; CXCL8; CDC42; RAC1; MAP3K14; MET; IGSF5; F11R; JAM2; JAM3; PLCG1; PLCG2; ADAM17; HBEGF; ADAM10; TJP1; CXCR1; CXCR2; CSK; EGFR; LYN; SRC; GIT1; PTPRZ1; PTPN11; NOD1
Pathogenic Escherichia coli infection	TUBA1B; TUBA3E; TUBA3D; TUBA8; TUBA4A; TUBA3C; TUBA1A; TUBAL3; TUBA1C; CTTN; HCLS1; ROCK1; ROCK2; OCLN; CDC42; ABL1; FYN; WASL; CLDN24; CLDN34; CLDN16; CLDN4; CLDN3; CLDN7; CLDN23; CLDN19; CLDN14; CLDN15; CLDN17; CLDN20; CLDN11; CLDN18; CLDN22; CLDN25; CLDN5; CLDN10; CLDN8; CLDN6; CLDN2; CLDN1; CLDN9; ITGB1; NCK1; NCK2; ARHGEF2; RHOA; NCL; TUBB3; TUBB4A; TUBB4B; TUBB; TUBB8; TUBB2B; TUBB2A; TUBB1; TUBB6; ARPC5; ARPC4; ARPC3; ARPC1B; ARPC2; ARPC1A; ARPC5L; ACTB; ACTG1; BAIAP2L1; BAIAP2; WIPF2; WIPF3; WIPF1; LPAR1; F2R; LPAR4; LPAR5; LPAR2; GNA13; GNA12; ARHGEF12; ARHGEF1; ARHGEF11; RAC1; SLC9A3R1; WASF2; NLRP4; PYCARD; CASP1; IL18; IL1B; NLRP3; NAIP; TNFRSF1A; TNF; EZR; RELA; NFKBIA; NFKBIB; IKBKB; TRAF2; RIPK1; TRADD;

	IRAK1; IRAK4; MYD88; IL1R1; TRAF6; TLR4; GAPDH; RPS3; TAB1; TAB2; TAB3; MAP3K7; MAPK8; MAPK9; MAPK10; MAPK14; MAPK11; MAPK13; MAPK12; MAPK1; MAPK3; FOS; JUN; PTPN6; PTPN11; NFKB1; CYTH4; CYTH3; CYTH2; CYTH1; ARF6; ARF1; WASF3; WASF1; FCGR2A; LOC102723407; SRC; TLR5; TIRAP; ABI1; NCKAP1; NCKAP1L; CYFIP1; CYFIP2; TJP1; MYO1H; MYO1B; MYO1F; MYH9; MYH10; MYH11; MYO1A; MYO1C; MYO1D; MYO1E; MYO5A; MYO5B; MYO6; MYO10; MYO5C; MYO1G; MYH14; FADD; TNFRSF10B; TNFRSF10A; FAS; TNFSF10; FASLG; CASP9; CASP7; CASP3; CASP8; BAX; BAK1; TMBIM6; CASP4; ABCF2; CYCS; IL6; CXCL8; BRK1; CHUK; IKBKG; F2; SEC24B; SEC24A; SEC24C; SEC24D; TMED10; RAB1A; PAK1; PAK2; PAK3
Shigellosis	TNFRSF1A; TNF; NFKBIA; NFKBIB; CHUK; IKBKB; TRAF2; TRAF5; RIPK1; TRADD; IL1R1; IL1B; TRAF6; TLR4; TAB1; TAB2; TAB3; MAP3K7; MAPK14; MAPK11; MAPK13; MAPK12; MAPK1; MAPK3; H3C14; H3-3A; H3-3B; H3C15; H3-5; H3C13; H3-4; H3C1; H3C4; H3C3; H3C6; H3C11; H3C8; H3C12; H3C10; H3C2; H3C7; NFKB1; TLR5; CXCL8; IKBKG; NOD1; RIPK2; ARHGEF2; PRKCD; PRKCE; PRKCQ; BCL10; MALT1; UBE2V1; UBE2V2; UBE2NL; UBE2N; TNIP1; U2AF1L5; U2AF1L4; U2AF1; CCL5; CSF2; CD14; SKP1; CUL1; RBX1; FBXW11; BTRC; UBE2D4; UBE2D1; UBE2D2; UBE2D3; CBX3; RPS6KA5; MAPK8; MAPK9; MAPK10; JUN; TIFA; RBCK1; RNF31; SHARPIN; NAIP; NLRC4; PYCARD; CASP1; IL18; NLRP3; GLMN; CGAS; STING1; TBK1; IRF3; IFNB1; ARF1; MYD88; RELA; ATG14; BECN2; BECN1; PIK3R4; PIK3C3; RRAGB; RRAGA; RRAGD; RRAGC; MTOR; RPTOR; AKT1S1; ATG16L1; ATG5; ATG12; GABARAP; GABARAPL2; GABARAPL1; CALCOCO2; SQSTM1; C3; RPS27A; UBA52; UBB; UBC; SEPTIN9; SEPTIN12; SEPTIN6; SEPTIN8; SEPTIN2; SEPTIN11; SEPTIN3; SEPTIN7; WIPI2; WIPI1; TECPR1; FNBP1; FNBP1L; RAC1; DOCK1; CRK; CRKL; CTTN; HCLS1; SRC; ELMO2; ELMO1; CDC42; WASF2; WASF1; ARPC5; ARPC4; ARPC3; ARPC1B; ARPC2; ARPC1A; ARPC5L; ROCK1; ROCK2; DIAPH1; RHOA; ACTB; ACTG1; CYTH4; CYTH3; CYTH2; CYTH1; ARF6; VCL; ITGA5; ITGB1; CD44; PTK2; TLN1; TLN2; PXN; ILK; ACTN4; ACTN1; BCAR1; PIK3CA; PIK3CB; PIK3CD; PIK3R1; PIK3R2; PIK3R3; CAST; ITPR1; ITPR2; ITPR3; PLCD3; PLCB1; PLCE1; PLCB2; PLCB3; PLCB4; PLCD1; PLCG1; PLCG2; PLCD4; PLCZ1; MDM2; AKT3; AKT1; AKT2; FOXO6; FOXO1; FOXO3; FOXO4; GSK3A; GSK3B; RPS6KB1; RPS6KB2; CAPNS1; CAPN1; CAPN2; TP53; ATM; BCL2; BCL2L1; EGFR; CASP4; HK1; HK2; HK3; HKDC1; VDAC1; CYCS; BNIP3; PPID; BAX; MYL12B; MYL9; MYL12A; MYLPF; MYL2; MYL5; MYL7; MYL10; WASL; PFN3; PFN4; PFN1; PFN2
Salmonella infection	MYD88; MAPK1; MAPK3; IKBKB; MAP3K7; MAPK14; MAPK11; MAPK13; MAPK12; IRAK1; IRAK4; CHUK; CXCL8; TIRAP; MAPK8; MAPK9; MAPK10; IL1B; TRAF6; IL6; TAB1; TAB2; TAB3; NFKBIA; TNF; TLR9; IKBKG; MAP2K7; MAP2K4; NFKB1; MAP2K3; MAP2K6; MAP2K1; MAP2K2; FOS; JUN; RELA; NLRC4; PYCARD; CASP1; IL18; NLRP3; NAIP; CASP4; CASP5; GSDMD; NOD1; RIPK2; HSP90AA1; HSP90AB1; HSP90B1; RPS3; SKP1; FBXO22; KPNA1; KPNA3; KPNA4; CSE1L; HRAS; RAF1; PKN1; CTNNB1; LEF1; TCF7; TCF7L2; TCF7L1; MYC; RAC1; AKT3; AKT1; AKT2; CDC42; RHOA; RHOJ; RHOG; ARHGEF26; WASF3; ABI1; BRK1; NCKAP1; NCKAP1L; CYFIP1; CYFIP2; ACTB; ACTG1;

	ARPC5; ARPC4; ARPC3; ARPC1B; ARPC2; ARPC1A; ARPC5L; CYTH4; CYTH3; CYTH2; CYTH1; ARF6; ARF1; PAK1; PAK3; MYO6; PIK3C2A; PIK3C2B; PIK3C2G; PIK3CA; PIK3CB; PIK3CD; PIK3CG; SNX18; SNX33; SNX9; DNM2; WASL; ROCK2; FHOD1; EXOC7; EXOC2; EXOC4; EXOC5; RALA; AHNAK2; AHNAK; RHOB; RHOH; RRAS; TRADD; TRAF2; RIPK1; BIRC2; BIRC3; CASP8; CASP7; TNFSF10; BAK1; BAX; CASP3; FADD; RIPK3; MLKL; BCL2; CYCS; TXN2; TXN; GAPDH; PODXL; PTPRC; FLNA; FLNB; FLNC; PFN3; PFN4; PFN1; PFN2; MYL12B; MYL9; MYL12A; MYLPF; MYL2; MYL5; MYL7; MYL10; TLR2; LY96; TLR5; TLR6; TLR4; CD14; ANXA2; S100A10; TNFRSF1A; TNFRSF10B; TNFRSF10A; ELMO2; ELMO1; RAB7B; RAB7A; RILP; FYCO1; DYNLL2; DYNC1H1; DYNC1I1; DYNC1I2; DYNC1LI2; DYNC1LI1; DYNC2LI1; DYNLT3; DYNLT1; DYNC2H1; DYNLRB2; DYNLRB1; DYNLL1; PLEKHM2; ACTR1B; ACTR1A; DCTN2; DCTN6; DCTN3; DCTN1; DCTN4; ACTR10; DCTN5; KLC3; KIF5A; KIF5B; KIF5C; KLC1; KLC2; KLC4; TUBA1B; TUBA3E; TUBA3D; TUBA8; TUBA4A; TUBA3C; TUBA1A; TUBAL3; TUBA1C; TUBB3; TUBB4A; TUBB4B; TUBB; TUBB8; TUBB2B; TUBB2A; TUBB1; TUBB6; PLEKHM1; VPS39; VPS41; VPS11; VPS18; VPS16; VPS33A; RAB5A; RAB5B; RAB5C; PIK3C3; STX10; GCC2; RAB9B; RAB9A; M6PR; ACBD3; ARL8A; ARL8B
Pertussis	ITGAM; ITGB1; ITGB2; CALML6; CALML5; CALM1; CALM2; CALM3; CALML3; CALML4; TLR4; LY96; NFKB1; RELA; MYD88; TIRAP; IRF3; NOD1; IL1A; IL1B; NOS2; IL6; IL23A; SFTPA1; SFTPA2; GNAI1; GNAI2; GNAI3; CXCL8; CXCL6; CXCL5; ITGA5; RHOA; CFL1; CFL2; CASP3; CASP7; C1R; C1S; C1QA; C1QB; C1QC; C4BPA; C4BPB; SERPING1; C4A; C4B; C2; C3; C5; MAPK14; MAPK11; MAPK13; MAPK12; MAPK1; MAPK3; FOS; JUN; MAPK8; MAPK9; MAPK10; TNF; IRAK1; TRAF6; TICAM2; TICAM1; CD14; IL12A; IL12B; IL10; IRF8; IRF1; CASP1; NLRP3; PYCARD; IRAK4
Legionellosis	NLRC4; PYCARD; CASP1; IL1B; IL18; NAIP; CASP7; NFKB1; RELA; NFKBIA; BNIP3; BCL2L13; ARF1; RAB1A; RAB1B; CLK1; CLK4; SEC22B; SAR1B; SAR1A; VCP; CYCS; CASP9; CASP3; CASP8; APAF1; ITGAM; ITGB2; CR1; CR1L; C3; HSPD1; NFKB2; EEF1G; EEF1A1; EEF1A2; HSF1; HBS1L; HSPA1A; HSPA1B; HSPA1L; HSPA2; HSPA6; HSPA8; TLR5; TLR2; TLR4; CD14; MYD88; TNF; IL6; CXCL8; IL12A; IL12B; CXCL1; CXCL2; CXCL3
Yersinia infection	ITGB1; SRC; WASL; ACTR3; ACTR2; ACTR3B; ACTR3C; FN1; BCAR1; CRK; CRKL; DOCK1; PIP5K1C; PIP5K1A; PIP5K1B; ACTB; ACTG1; PTK2B; PTK2; WIPF2; WIPF3; WIPF1; ARF6; PXN; GIT2; ARHGEF7; WAS; RHOA; ROCK1; ROCK2; BAIAP2; WASF2; NLRC4; MEFV; PYCARD; CASP1; IL18; IL1B; LIMK1; PKN1; PKN2; RPS6KA6; RPS6KA1; RPS6KA2; RPS6KA3; NLRP3; ARHGEF12; ARHGEF28; ARHGEF1; ITGA4; ITGA5; FYB1; SKAP2; LCK; ZAP70; LCP2; LAT; PLCG1; NFATC1; NFATC2; NFATC3; IL2; IL10; VAV3; VAV1; VAV2; FOS; JUN; RAC1; IKBKB; MAP3K7; MAPK14; MAPK11; MAPK13; MAPK12; CHUK; MAPK8; MAPK9; MAPK10; TRAF2; TRAF6; TAB1; TAB2; NFKBIA; IKBKG; MAP2K7; MAP2K4; NFKB1; RELA; MAP2K3; MAP2K6; MYD88; IRAK1; TLR4; IRAK4; MAPK1; MAPK3; MAP2K1; MAP2K2; CXCL8; TNF; IFNB1; IL6; PIK3CA; PIK3CB; PIK3CD; PIK3R1; PIK3R2; PIK3R3; AKT3; AKT1; AKT2; GSK3B; CCL2; TICAM1;

	TBK1; IRF3; RHOG; ELMO2; ELMO1; CDC42; FCGR2A; GNAQ; LOC102723407; RAC2; RAC3
Chagas disease	TLR2; TLR4; IFNG; CALR; C3; C1QA; C1QB; C1QC; TLR9; MYD88; IRAK1; IRAK4; TRAF6; MAP2K4; MAPK8; MAPK9; MAPK10; MAPK14; MAPK11; MAPK13; MAPK12; MAPK1; MAPK3; FOS; JUN; TNF; IL12A; IL12B; TICAM1; IFNB1; NFKBIA; NFKB1; RELA; CHUK; IKBKB; IKBKG; IL1B; IFNGR1; IFNGR2; NOS2; TNFRSF1A; TGFBR1; TGFBR2; SMAD2; SERPINE1; GNAI1; GNAI2; GNAI3; GNAO1; BDKRB2; GNA11; GNA15; GNAQ; GNA14; PLCB1; PLCB2; PLCB3; PLCB4; GNAL; GNAS; ADCY1; PIK3CA; PIK3CB; PIK3CD; PIK3R1; PIK3R2; PIK3R3; AKT3; AKT1; AKT2; IL10; TGFB1; TGFB2; TGFB3; PPP2CA; PPP2CB; PPP2R1A; PPP2R1B; PPP2R2A; PPP2R2B; PPP2R2C; PPP2R2D; FASLG; FAS; FADD; CASP8; CFLAR; CCL2; CXCL8; IL6; CCL3L3; CCL3; CCL3L1; CD3D; CD3E; CD3G; CD247; CCL5; IL2; TLR6; KNG1; ACE
Toxoplasmosis	MYD88; IRAK1; IRAK4; TRAF6; MAP3K7; TAB1; TAB2; CHUK; IKBKB; IKBKG; NFKBIA; NFKBIB; NFKB1; RELA; MAPK1; MAPK3; MAPK8; MAPK9; MAPK10; MAPK14; MAPK11; MAPK13; MAPK12; MAP2K3; MAP2K6; GNAI1; GNAI2; GNAI3; GNAO1; IL12A; IL12B; TNF; BCL2; BCL2L1; BIRC2; BIRC3; XIAP; BIRC7; NOS2; JAK1; JAK2; STAT1; CIITA; IRGM; HLA-DMA; HLA-DMB; HLA-DOA; HLA-DOB; HLA-DPA1; HLA-DPB1; HLA-DQA1; HLA-DQA2; HLA-DQB1; HLA-DRA; HLA-DRB1; HLA-DRB3; HLA-DRB4; HLA-DRB5; ALOX5; SOCS1; CYCS; STAT3; CASP9; CASP3; CASP8; PIK3R6; PIK3R5; PIK3CG; PDPK1; AKT3; AKT1; AKT2; ITGA6; ITGB1; LAMC3; LAMB4; LAMA1; LAMA2; LAMA3; LAMA4; LAMA5; LAMB1; LAMB2; LAMB3; LAMC1; LAMC2; LY96; TLR4; HSPA1A; HSPA1B; HSPA1L; HSPA2; HSPA6; HSPA8; TLR2; IFNG; IFNGR1; IFNGR2; CCR5; PPIF; CD40LG; CD40; BAD; TNFRSF1A; LDLR; IL10; TGFB1; TGFB2; TGFB3; IL10RA; IL10RB; TYK2
Amoebiasis	IL1B; MUC2; FN1; LAMC3; LAMB4; LAMA1; LAMA2; LAMA3; LAMA4; LAMA5; LAMB1; LAMB2; LAMB3; LAMC1; LAMC2; NFKB1; RELA; CXCL8; IL6; CASP3; CXCL1; CXCL2; CXCL3; CSF2; IL1R1; IL1R2; TNF; NOS2; TLR2; TLR4; LOC102723407; IL12A; IL12B; RAB7B; RAB5A; RAB5B; RAB5C; RAB7A; HSPB1; CD1A; CD1B; CD1C; CD1D; CD1E; CD14; PIK3CA; PIK3CB; PIK3CD; PIK3R1; PIK3R2; PIK3R3; IFNG; ITGAM; ITGB2; ARG1; ARG2; PRDX1; C8A; C8B; C8G; C9; GNAL; GNAS; GNA11; GNA15; GNAQ; GNA14; PRKCA; PRKCB; PRKCG; ADCY1; PLCB1; PLCB2; PLCB3; PLCB4; PRKACA; PRKACB; PRKACG; IL10; TGFB1; TGFB2; TGFB3; COL1A1; COL1A2; COL4A1; COL4A2; COL4A3; COL4A4; COL4A5; COL4A6; VCL; ACTN4; ACTN1; COL3A1; PTK2; CTSG; SERPINB6; SERPINB9; SERPINB10; SERPINB13; SERPINB3; SERPINB4
Staphylococcus aureus infection	CFD; C3; CFB; C5; C1S; C1R; C1QA; C1QB; C1QC; C4A; C4B; C2; MBL2; MASP2; MASP1; C5AR1; FPR1; PLG; LOC102723407; CFI; FGG; FCAR; FCGR1A; FCGR2A; FCGR2B; FCGR3A; FCGR3B; FCGR2C; DSG1; HLA-DMA; HLA-DMB; HLA-DOA; HLA-DOB; HLA-DPA1; HLA-DPB1; HLA-DQA1; HLA-DQA2; HLA-DQB1; HLA-DRA; HLA-DRB1; HLA-DRB3; HLA-DRB4; HLA-DRB5; CFH; C3AR1; LOC100653049; KRT40; KRT25; KRT28; KRT24; KRT23; KRT27; KRT26; KRT9; KRT10; KRT12; KRT13; KRT14; KRT15; KRT16; KRT17; KRT18; KRT19; KRT31;

	KRT32; KRT33A; KRT33B; KRT34; KRT35; KRT39; KRT20; KRT38; KRT37; KRT36; ICAM1; ITGB2; SELP; SELPLG; ITGAL; ITGAM; PTAFR; IL10; FPR2; FPR3; DEFB4B; DEFB1; DEFB4A; DEFB103A; DEFB103B; DEFA1; DEFA3; DEFA4; DEFA5; DEFA6; DEFA1B; CAMP
Tuberculosis	PLA2R1; MRC1; MRC2; CLEC4M; CD209; RAF1; IL10; RAB5A; RAB5B; RAB5C; PIK3C3; TCIRG1; ATP6V0A2; ATP6V0D2; ATP6V0A4; ATP6V1H; ATP6V0C; ATP6V0B; ATP6V0A1; ATP6AP1; ATP6V0D1; EEA1; LAMP1; LAMP2; RAB7A; CTSD; CALML6; CALML5; CALM1; CALM2; CALM3; CALML3; CALML4; CAMK2A; CAMK2B; CAMK2D; CAMK2G; SPHK2; SPHK1; FCGR1A; FCGR2A; FCGR2B; FCGR3A; FCGR3B; FCGR2C; CORO1A; PPP3CA; PPP3CB; PPP3CC; PPP3R1; PPP3R2; ITGB2; ITGAM; CREBBP; EP300; STAT1; JAK1; JAK2; IFNGR2; IFNG; RFX5; RFXAP; RFXANK; CREB1; NFYA; NFYB; NFYC; TLR2; CEBPB; CEBPG; CIITA; MAPK1; MAPK3; NFKB1; RELA; TRAF6; CD14; MYD88; IRAK4; TLR4; TLR6; TLR1; CASP3; TNF; TNFRSF1A; TRADD; FADD; AKT3; AKT1; AKT2; CASP10; CASP8; BID; CYCS; APAF1; BAD; CASP9; BCL2; BAX; CD74; HLA-DMA; HLA-DMB; HLA-DOA; HLA-DOB; HLA-DPA1; HLA-DPB1; HLA-DQA1; HLA-DQA2; HLA-DQB1; HLA-DRA; HLA-DRB1; HLA-DRB3; HLA-DRB4; HLA-DRB5; LOC102723407; CTSS; IL10RA; IL12A; IL12B; IL1A; IL1B; TIRAP; TLR9; IFNA1; IFNA2; IFNA4; IFNA5; IFNA6; IFNA7; IFNA8; IFNA10; IFNA13; IFNA14; IFNA16; IFNA17; IFNA21; IL6; ARHGEF12; RHOA; LSP1; KSR1; PLK3; ITGAX; CR1; CR1L; LBP; VDR; CYP27B1; CAMP; IL18; IL23A; NOD2; RIPK2; CLEC7A; SYK; SRC; CARD9; MALT1; BCL10; CLEC4E; FCER1G; IFNGR1; HSPD1; HSPA9; MAPK8; MAPK9; MAPK10; MAPK14; MAPK11; MAPK13; MAPK12; TGFB1; TGFB2; TGFB3; IRAK1; IRAK2; IL10RB; C3; NOS2; IFNB1
Hepatitis C	DDX58; MAVS; TRAF3; TBK1; IKBKE; IRF3; IRF7; NFKB1; RELA; TLR3; TICAM1; NFKBIA; CHUK; IKBKB; IKBKG; TRAF6; RIPK1; IFNA1; IFNA2; IFNA4; IFNA5; IFNA6; IFNA7; IFNA8; IFNA10; IFNA13; IFNA14; IFNA16; IFNA17; IFNA21; IFNB1; IFNAR1; IFNAR2; JAK1; TYK2; STAT1; STAT2; IRF9; SCARB1; CD81; CLDN24; CLDN34; CLDN16; CLDN4; CLDN3; CLDN7; CLDN23; CLDN19; CLDN14; CLDN15; CLDN17; CLDN20; CLDN11; CLDN18; CLDN22; CLDN25; CLDN5; CLDN10; CLDN8; CLDN6; CLDN2; CLDN1; CLDN9; OCLN; SOCS3; PPP2CA; PPP2CB; PPP2R1A; PPP2R1B; PPP2R2A; PPP2R2B; PPP2R2C; PPP2R2D; PIAS1; OAS1; OAS2; OAS3; RNASEL; EIF2AK1; EIF2AK4; EIF2AK2; EIF2AK3; EIF2S1; MAPK1; MAPK3; IFIT1; IFIT1B; EIF3E; EGFR; GRB2; SOS1; SOS2; HRAS; KRAS; NRAS; ARAF; RAF1; BRAF; PIK3CA; PIK3CB; PIK3CD; PIK3R1; PIK3R2; PIK3R3; AKT3; AKT1; AKT2; GSK3B; EGF; TP53; CDKN1A; TNF; TNFRSF1A; TRADD; TRAF2; PSME3; RXRA; NR1H3; LDLR; BAD; PPARA; IFNG; CXCL10; MX1; MX2; RSAD2; STAT3; MAP2K1; MAP2K2; YWHAQ; YWHAB; YWHAE; YWHAG; YWHAH; YWHAZ; CTNNB1; CDK2; CDK4; CDK6; RB1; E2F1; E2F2; E2F3; FASLG; FAS; FADD; CASP8; CASP3; BID; BAX; CYCS; CASP9; BAK1; APAF1; CFLAR; CCND1; MYC
Hepatitis B	VDAC3; CYCS; CASP9; APAF1; CASP12; CASP3; BAX; BIRC5; PRKCA; PRKCB; PRKCG; ARAF; RAF1; BRAF; MAP2K1; MAP2K2; MAPK1; MAPK3; FOS; JUN; MMP9; MYC; ELK1; STAT3; CREB3; CREB1; ATF2; ATF6B; CREB3L4; ATF4;

	CREB3L2; CREB3L3; CREB3L1; CREB5; CXCL8; PCNA; JAK1; JAK2; JAK3; TYK2; PTK2B; SRC; GRB2; HRAS; KRAS; NRAS; MAP3K1; MAPK8; MAPK9; MAPK10; NFKB1; RELA; NFKBIA; SMAD4; ATP6AP1; NFATC1; NFATC2; NFATC3; NFATC4; TNF; PIK3CA; PIK3CB; PIK3CD; PIK3R1; PIK3R2; PIK3R3; AKT3; AKT1; AKT2; BAD; CHUK; IKBKB; IKBKG; BCL2; DDB1; FAS; FASLG; FADD; CASP8; CASP10; MAP2K4; YWHAQ; YWHAB; YWHAZ; TGFB1; TGFB2; TGFB3; TGFBR1; SMAD3; CDKN1A; CREBBP; EP300; EGR2; EGR3; HSPG2; SLC10A1; TBK1; IKBKE; IRF3; IRF7; TLR3; TICAM1; DDX58; MAVS; IFIH1; IFNAR1; IFNA1; IFNA2; IFNA4; IFNA5; IFNA6; IFNA7; IFNA8; IFNA10; IFNA13; IFNA14; IFNA16; IFNA17; IFNA21; STAT1; STAT2; IFNB1; IL6; CCNE1; CCNE2; CDK2; TP53; CCNA2; CCNA1; MYD88; DDB2; MAP3K7; IRAK1; TIRAP; TRAF6; TAB1; TAB2; TLR2; TLR4; IRAK4; MAPK14; MAPK11; MAPK13; MAPK12; MAP2K7; MAP2K3; MAP2K6; BID; RB1; DDX3X; TRAF3; TICAM2; STAT4; STAT5A; STAT5B; STAT6; E2F1; E2F2; E2F3; TGFBR2; SOS1; SOS2
Measles	TLR7; TLR9; MYD88; IRAK1; IRAK4; IRF7; IFNA1; IFNA2; IFNA4; IFNA5; IFNA6; IFNA7; IFNA8; IFNA10; IFNA13; IFNA14; IFNA16; IFNA17; IFNA21; TLR2; TLR4; DDX58; NFKBIA; NFKBIB; NFKB1; RELA; IFIH1; MAVS; IRF3; IFNB1; IFNAR1; IFNAR2; JAK1; TYK2; STAT1; STAT2; IRF9; OAS1; OAS2; OAS3; MSN; CLEC4M; CD209; CD46; SLAMF1; IL2RA; IL2RB; IL2RG; IL2; CD3D; CD3E; CD3G; CD28; PIK3CA; PIK3CB; PIK3CD; PIK3R1; PIK3R2; PIK3R3; AKT3; AKT1; AKT2; HSPA1A; HSPA1B; HSPA1L; HSPA2; HSPA6; HSPA8; EIF3H; JAK3; STAT3; STAT5A; STAT5B; IL1A; IL1B; IL6; IL12A; IL12B; MX1; MX2; RACK1; CBLB; EIF2AK1; EIF2AK4; EIF2AK2; EIF2AK3; EIF2S1; CHUK; IKBKB; IKBKG; FAS; FASLG; TRAF6; TNFAIP3; FCGR2B; CSNK2A1; CSNK2A2; CSNK2B; CSNK2A3; RCHY1; TP53; TP73; BBC3; RAB9B; RAB9A; CCND1; CCND2; CCND3; CCNE1; CCNE2; CDK2; CDK4; CDK6; CDKN1B; GSK3B; ADAR; MAP3K7; TAB2; IKBKE; TBK1; MAPK8; MAPK9; MAPK10; FOS; JUN; FADD; CASP8; TRADD; BAD; BAX; BAK1; CYCS; CASP3; CASP9; BID; BCL2; BCL2L1; APAF1; TRAF3
Human cytomegalovirus infection	EIF4EBP1; RPS6KB1; RPS6KB2; MTOR; RHEB; TSC2; TSC1; AKT3; AKT1; AKT2; PIK3CA; PIK3CB; PIK3CD; PIK3R1; PIK3R2; PIK3R3; EGFR; FASLG; CHUK; IKBKB; IKBKG; NFKB1; RELA; GRB2; SOS1; SOS2; HRAS; KRAS; NRAS; RAF1; MAP2K1; MAP2K2; MAPK1; MAPK3; ITGAV; ITGB3; TNF; TNFRSF1A; FADD; CASP8; FAS; PDGFRA; HLA-A; HLA-B; HLA-C; HLA-E; HLA-F; HLA-G; PTGS2; TRADD; NFKBIA; BAX; BAK1; CYCS; CASP3; STAT3; GNB5; GNB1; GNB2; GNB3; GNG3; GNG4; GNG5; GNG7; GNG10; GNG11; GNGT1; GNGT2; GNG13; GNG2; GNG12; GNB4; GNG8; CASP9; BID; IL6; CXCL8; VEGFA; NFATC1; NFATC2; NFATC3; NFATC4; PPP3CA; PPP3CB; PPP3CC; PPP3R1; PPP3R2; ITPR1; ITPR2; ITPR3; CALML6; CALML5; CALM1; CALM2; CALM3; CALML3; CALML4; PLCB1; PLCB2; PLCB3; PLCB4; GNA11; GNAQ; GNA13; GNA12; RHOA; ROCK1; ROCK2; TAP1; TAP2; TAPBP; CCL2; CCL3L3; CCL3; CCL3L1; CCL4L1; CCL4; CCL4L2; CCL5; CX3CL1; GNAI1; GNAI2; GNAI3; GNAO1; ADCY1; ADCY2; ADCY3; ADCY5; ADCY6; ADCY7; ADCY8; ADCY9; ADCY4; PRKACA; PRKACB; PRKACG; IL6R; JAK1; CCND1; SRC; RAC1; RAC2; RAC3; PTGER1; PTGER2; PTGER3; PTGER4; CTNNB1; GSK3B; MYC; IL1R1; IL1B; CCR1; CCR3; CCR5;

	CXCL12; CXCR4; PTK2; CRK; CRKL; BCAR1; PXN; PRKCA; PRKCB; PRKCG; PTK2B; IL10RA; IL10RB; CXCR2; MAPK14; MAPK11; MAPK13; MAPK12; CREB3; CREB1; ATF2; ATF6B; CREB3L4; ATF4; CREB3L2; CREB3L3; CREB3L1; CREB5; GNAS; MAP2K6; CDKN2A; CDKN1A; MDM2; TP53; E2F1; E2F2; E2F3; RB1; CDK4; CDK6; ELK1; SP1; RIPK1; TRAF5; TRAF2; AKAP13; ARHGEF12; ARHGEF1; ARHGEF11; PDIA3; CALR; B2M; IFNB1; IFNA1; IFNA2; IFNA4; IFNA5; IFNA6; IFNA7; IFNA8; IFNA10; IFNA13; IFNA14; IFNA16; IFNA17; IFNA21; TBK1; IRF3; CGAS; STING1
Influenza A	TPSD1; TPSB2; TPSAB1; PLG; PRSS1; PRSS2; PRSS3; DDX58; IFIH1; MAVS; TRIM25; TBK1; IKBKE; TLR3; TICAM1; CHUK; IKBKB; IKBKG; NFKB1; RELA; NFKBIA; NFKBIB; CREBBP; EP300; IRF3; IRF7; PIK3CA; PIK3CB; PIK3CD; PIK3R1; PIK3R2; PIK3R3; AKT3; AKT1; AKT2; TLR7; MYD88; IRAK4; NLRP3; PYCARD; CASP1; IL1B; IL18; IL33; IFNAR1; IFNAR2; IFNB1; JAK1; TYK2; STAT1; STAT2; IRF9; MX1; MX2; OAS1; OAS2; OAS3; ADAR; PML; CIITA; HLA-DMA; HLA-DMB; HLA-DOA; HLA-DOB; HLA-DPA1; HLA-DPB1; HLA-DQA1; HLA-DQA2; HLA-DQB1; HLA-DRA; HLA-DRB1; HLA-DRB3; HLA-DRB4; HLA-DRB5; IFNA1; IFNA2; IFNA4; IFNA5; IFNA6; IFNA7; IFNA8; IFNA10; IFNA13; IFNA14; IFNA16; IFNA17; IFNA21; SOCS3; CCL5; CXCL8; ICAM1; IL1A; IL6; TNF; IL12A; IL12B; EIF2AK2; DNAJC3; DNAJB1; EIF2S1; RNASEL; TNFSF10; TNFRSF10B; TNFRSF10A; SLC25A4; SLC25A5; SLC25A6; SLC25A31; VDAC1; KPNA6; KPNA1; KPNA2; KPNA5; KPNA7; XPO1; PRKCA; RAF1; MAP2K1; MAP2K2; MAPK1; MAPK3; ACTB; ACTG1; FDPS; RSAD2; CPSF4; BCL2L2-PABPN1; PABPN1L; PABPN1; NXF1; NXF5; NXF3; NXF2; NXF2B; NXT1; NXT2; RAE1; HNRNPUL1; NUP98; CCL2; CXCL10; PRKCB; TMPRSS4; TMPRSS2; TMPRSS11D; FASLG; FAS; NLRX1; RAB11A; RAB11B; IFNG; IFNGR1; IFNGR2; JAK2; TLR4; TNFRSF1A; TRAF3; CCND3; CDK4; CDK6; CALCOCO2; FADD; CASP8; TRADD; BAX; BAK1; CYCS; CASP3; CASP9; BID; APAF1
Human papillomavirus infection	EIF4EBP1; RPS6KB1; RPS6KB2; MTOR; RHEB; TSC2; TSC1; AKT3; AKT1; AKT2; PIK3CA; PIK3CB; PIK3CD; PIK3R1; PIK3R2; PIK3R3; EGFR; GSK3B; FASLG; BAD; MDM2; TP53; CCND1; CHUK; IKBKB; IKBKG; NFKB1; RELA; EGF; GRB2; SOS1; SOS2; HRAS; KRAS; NRAS; CDKN1A; CDKN1B; CDK2; RAF1; MAP2K1; MAP2K2; MAPK1; MAPK3; ITGA11; ITGA6; ITGA1; ITGA2; ITGA2B; ITGA3; ITGA4; ITGA5; ITGA7; ITGA9; ITGAV; ITGA10; ITGA8; ITGB1; ITGB3; ITGB4; ITGB5; ITGB6; ITGB7; ITGB8; LAMC3; CHAD; COL1A1; COL1A2; COL2A1; COL4A1; COL4A2; COL4A3; COL4A4; COL4A5; COL4A6; COL6A1; COL6A2; COL6A3; COL9A1; COL9A2; COL9A3; COMP; COL6A6; LAMB4; FN1; COL6A5; LAMA1; TNC; IBSP; LAMA2; LAMA3; LAMA4; LAMA5; LAMB1; LAMB2; LAMB3; LAMC1; LAMC2; RELN; TNN; SPP1; THBS1; THBS2; THBS3; THBS4; TNR; TNXB; VTN; VWF; PTK2; CCNE1; CCNE2; TNF; TNFRSF1A; FADD; CASP8; CASP3; FAS; MAGI1; PTEN; PARD3; PARD6A; PARD6G; PARD6B; PRKCI; PRKCZ; CDC42; SCRIB; DLG1; DLG2; DLG3; LLGL2; LLGL1; CRB3; PATJ; MPP5; PXN; VEGFA; NFX1; UBE3A; TERT; RB1; RBL1; RBL2; E2F1; CCNA2; CCNA1; CDK4; CDK6; CCND2; CCND3; CREBBP; EP300; JAG1; NOTCH1; NOTCH2; NOTCH3; NOTCH4; RBPJL; RBPJ; MAML3; MAML2; MAML1; HES1; HES5; HES3; HES2; HES6; HES4; HES7; HEY1;

	HEY2; HEYL; TLR3; TICAM1; TRAF3; IKBKE; TBK1; IRF3; IFNB1; PSEN1; LFNG; MFNG; RFNG; IFNA1; IFNA2; IFNA4; IFNA5; IFNA6; IFNA7; IFNA8; IFNA10; IFNA13; IFNA14; IFNA16; IFNA17; IFNA21; IFNAR1; IFNAR2; TYK2; JAK1; STAT2; STAT1; IRF9; EIF2AK2; OASL; MX1; MX2; ISG15; PDGFRB; BCAP31; HLA-A; HLA-B; HLA-C; HLA-E; HLA-F; HLA-G; TCIRG1; ATP6V1G3; ATP6V0E2; ATP6V0A2; ATP6V0D2; ATP6V1C2; ATP6V0A4; ATP6V1D; ATP6V1H; ATP6V1A; ATP6V1B1; ATP6V1B2; ATP6V0C; ATP6V1C1; ATP6V1E1; ATP6V0B; ATP6V1G2; ATP6V0A1; ATP6AP1; ATP6V0E1; ATP6V1E2; ATP6V0D1; ATP6V1F; ATP6V1G1; PTGS2; PTGER4; GNAS; PRKACA; PRKACB; PRKACG; BAX; CREB3; CREB1; CREB3L4; CREB3L2; CREB3L3; CREB3L1; CREB5; PPP2R3B; PPP2R3C; PPP2CA; PPP2CB; PPP2R1A; PPP2R1B; PPP2R2A; PPP2R2B; PPP2R2C; PPP2R3A; PPP2R5A; PPP2R5B; PPP2R5C; PPP2R5D; PPP2R5E; PPP2R2D; FOXO1; SLC9A3R1; FZD10; FZD2; FZD5; FZD3; FZD1; FZD4; FZD6; FZD7; FZD8; FZD9; DVL1; DVL2; DVL3; CTNNB1; AXIN1; AXIN2; APC2; APC; CSNK1A1L; CSNK1A1; TCF7; TCF7L2; TCF7L1; WNT16; WNT4; WNT1; WNT2; WNT3; WNT5A; WNT6; WNT7A; WNT7B; WNT8A; WNT8B; WNT10B; WNT11; WNT2B; WNT9A; WNT9B; WNT10A; WNT5B; WNT3A; TRADD; UBR4; HDAC1; HDAC2; CHD4; IRF1; TBPL2; TBP; TBPL1; TUBG2; TUBG1; ATM; ATR; BAK1; PKM; TADA3; PSMC1
Human T-cell leukemia virus 1 infection	NRP1; SLC2A1; TLN1; TLN2; ICAM1; ITGAL; ITGB2; NFATC1; NFATC2; NFATC3; NFATC4; PPP3CA; PPP3CB; PPP3CC; PPP3R1; PPP3R2; CANX; CALR; IL2RB; IL2RG; JAK1; JAK3; STAT5A; STAT5B; HLA-A; HLA-B; HLA-C; HLA-E; HLA-F; HLA-G; MYC; TRRAP; KAT5; CCND2; FDPS; HRAS; KRAS; NRAS; SLC25A4; SLC25A5; SLC25A6; TSPO; VDAC1; VDAC2; VDAC3; SLC25A31; XPO1; RAN; RANBP3; ADCY1; ADCY2; ADCY3; ADCY5; ADCY6; ADCY7; ADCY8; ADCY9; ADCY4; PRKACA; PRKACB; PRKACG; CREB3; CREB1; ATF2; ATF6B; CREB3L4; ATF4; CREB3L2; CREB3L3; CREB3L1; CREB5; TGFB1; TGFB2; TGFB3; TGFBR1; TGFBR2; SMAD2; SMAD3; SMAD4; CDKN1A; ANAPC10; CDC26; ANAPC2; ANAPC4; ANAPC5; ANAPC7; ANAPC11; ANAPC1; CDC23; CDC16; CDC27; CDC20; CCNB2; PTTG2; PTTG1; BUB1B; MAD2L1; BUB3; MAD1L1; RANBP1; VAC14; TERT; NFKB1; RELA; NFKBIA; IL2; IL2RA; IL15; IL15RA; IL6; CSF2; LTA; NFKB2; RELB; CHUK; MAP3K14; LTBR; CD40; TNFRSF13C; IKBKB; IKBKG; TNFRSF1A; IL1R1; IL1R2; CD3D; CD3E; CD3G; MAP3K1; MAP3K3; MAP2K4; MAPK8; MAPK9; MAPK10; JUN; GPS2; BCL2L1; XIAP; PIK3CA; PIK3CB; PIK3CD; PIK3R1; PIK3R2; PIK3R3; AKT3; AKT1; AKT2; CDKN2A; CDKN2B; CDK4; CCND1; CCND3; RB1; E2F1; E2F2; E2F3; SRF; ELK1; ELK4; ETS1; ETS2; SPI1; FOS; EGR1; EGR2; FOSL1; TBPL2; TBP; TBPL1; NFYB; HLA-DMA; HLA-DMB; HLA-DOA; HLA-DOB; HLA-DPA1; HLA-DPB1; HLA-DQA1; HLA-DQA2; HLA-DQB1; HLA-DRA; HLA-DRB1; HLA-DRB3; HLA-DRB4; HLA-DRB5; KAT2A; KAT2B; CREBBP; EP300; TCF3; CDKN2C; LCK; POLB; BAX; CRTC2; CRTC1; CRTC3; MSX1; MSX2; ZFP36; CHEK1; CHEK2; ATM; ATR; TNF; MAP2K1; MAP2K2; MAPK1; MAPK3; CD4; B2M; ESPL1; CCNA2; CCNA1; CCNE1; CCNE2; CDK2; TP53; MMP7; PTEN; DLG1
Kaposi sarcoma-associated herpesvirus infection	IFNA1; IFNA2; IFNA4; IFNA5; IFNA6; IFNA7; IFNA8; IFNA10; IFNA13; IFNA14; IFNA16; IFNA17; IFNA21; IFNB1; TRAF3; IRF7; CHUK; IKBKB; IKBKG; MAPK8;

	MAPK9; MAPK10; TBK1; IKBKE; AKT3; AKT1; AKT2; STAT1; NFKBIA; IRF3; TLR3; TICAM1; PIK3CA; PIK3CB; PIK3CD; PIK3R1; PIK3R2; PIK3R3; IFNAR1; IFNAR2; NFKB1; RELA; FAS; CASP8; BAX; BAK1; CYCS; CASP3; TNFRSF1A; IL6ST; CREBBP; EP300; JAK1; TYK2; STAT2; IRF9; C3; HLA-A; HLA-B; HLA-C; HLA-E; HLA-F; HLA-G; CDKN1A; CDK4; CDK6; RB1; TP53; E2F1; E2F2; E2F3; GSK3B; CTNNB1; JAK2; STAT3; GNB5; GNB1; GNB2; GNB3; GNG3; GNG4; GNG5; GNG7; GNG10; GNG11; GNGT1; GNGT2; GNG13; GNG2; GNG12; GNB4; GNG8; HIF1A; EIF2AK2; CCR3; CCR5; CCR1; CCR4; CCR8; CD200R1; CD200R1L; GABARAP; GABARAPL2; GABARAPL1; ATG3; ATG14; PIK3C3; BECN2; BECN1; TRADD; TRAF2; CASP9; BID; FADD; IL6; CXCL8; PTGS2; CXCL1; CXCL2; CXCL3; RCAN1; FGF2; VEGFA; PDGFB; MTOR; SYK; PLCG1; NFATC1; NFATC2; NFATC3; NFATC4; PPP3CA; PPP3CB; PPP3CC; PPP3R1; PPP3R2; ITPR1; ITPR2; ITPR3; HRAS; KRAS; NRAS; RAF1; MAP2K1; MAP2K2; MAPK1; MAPK3; FOS; JUN; RAC1; PLCG2; ANGPT2; SRC; HCK; LYN; CREB1; CCND1; MYC; CLEC2B; ICAM1; CD86; MICA; MICB; IFNGR1; MAPKAPK2; MAPK14; MAPK11; MAPK13; MAPK12; MAP2K6; CSF2; ZFP36; PIK3R6; PIK3R5; PIK3CG; PREX1; CALML6; CALML5; CALM1; CALM2; CALM3; CALML3; CALML4; RPS27A; UBA52; UBB; UBC; MAP2K7; MAP2K4
Herpes simplex virus 1 infection	LTA; TNFSF14; TNFRSF14; NECTIN1; NECTIN2; PILRB; PILRA; TRAF2; TRAF5; NFKB1; RELA; PTPN11; C3; C5; CFP; TLR3; TLR9; TLR2; TICAM1; MYD88; TAB1; TAB2; MAP3K7; IRF3; IRF7; NFKBIA; IFNA1; IFNA2; IFNA4; IFNA5; IFNA6; IFNA7; IFNA8; IFNA10; IFNA13; IFNA14; IFNA16; IFNA17; IFNA21; IL12A; IL12B; TNF; IL1B; IL6; CCL2; CCL5; POU2F3; POU2F1; POU2F2; HCFC2; HCFC1; TRAF6; IKBKE; TBK1; DDX58; MAVS; IFIH1; PML; SP100; DAXX; TP53; EIF2AK1; EIF2AK4; EIF2AK2; EIF2AK3; EIF2S1; PPP1CA; PPP1CB; PPP1CC; OAS1; OAS2; OAS3; RNASEL; ALYREF; NXF1; NXF5; NXF3; NXF2; NXF2B; IFNAR1; IFNAR2; IFNGR1; IFNGR2; IFNB1; JAK1; TYK2; STAT1; STAT2; IRF9; JAK2; IFNG; SRPK1; SRSF8; SRSF1; SRSF2; SRSF3; SRSF4; SRSF5; SRSF6; SRSF7; SRSF9; SOCS3; CD74; HLA-DMA; HLA-DMB; HLA-DOA; HLA-DOB; HLA-DPA1; HLA-DPB1; HLA-DQA1; HLA-DQA2; HLA-DQB1; HLA-DRA; HLA-DRB1; HLA-DRB3; HLA-DRB4; HLA-DRB5; FAS; TNFRSF1A; FASLG; FADD; CASP8; CHUK; IKBKB; IKBKG; BIRC2; BIRC3; IRAK4; IRAK1; TRAF3; CGAS; STING1; HLA-A; HLA-B; HLA-C; HLA-E; HLA-F; HLA-G; TAP1; TAP2; TAPBP; PDIA3; CALR; B2M; TRADD; AKT3; AKT1; AKT2; TSC1; TSC2; RHEB; MTOR; EIF4EBP1; PIK3CA; PIK3CB; PIK3CD; PIK3R1; PIK3R2; PIK3R3; BAD; ITGA5; ITGB3; SRC; SYK; CARD9; ZNF730; ZNF737; ZNF316; ZNF705E; ZNF717; ZNF729; ZNF605; ZNF783; ZNF559-ZNF177; ZNF891; ZNF256; ZNF443; ZNF267; ZNF211; ZNF234; ZNF273; ZNF460; ZNF268; ZNF275; ZNF257; ZIM3; ZNF554; ZNF689; ZNF354B; ZNF641; ZNF816; ZNF543; ZNF813; ZNF441; ZNF440; ZNF573; ZNF57; ZNF792; ZNF684; ZNF786; ZFP92; ZFP28; ZFP90; ZNF597; ZNF785; ZNF688; ZNF480; ZNF534; ZNF578; ZNF418; ZNF417; ZNF548; ZNF560; ZNF563; ZNF420; ZNF565; ZNF582; ZNF583; ZNF599; ZNF558; ZNF98; ZNF714; ZNF555; ZNF569; ZNF570; ZNF595; ZNF425; ZNF746; ZNF782; ZFP1; ZNF519; ZNF836; ZNF610; ZNF600; ZNF320; ZNF550; ZNF846; ZNF791; ZNF564; ZNF709; ZNF433; ZNF114; ZNF567; ZNF383; ZNF780B;

	ZNF676; ZNF100; ZNF540; ZNF679; ZNF596; ZNF169; ZNF431; ZNF721; ZNF675; ZNF778; ZNF627; ZNF585A; ZNF584; ZNF25; ZFP30; ZNF510; ZNF620; ZNF718; ZNF549; ZNF324; ZNF473; ZNF658; ZNF337; ZNF285; ZNF777; ZNF544; ZNF311; ZNF547; ZIK1; ZNF780A; ZNF283; ZNF615; ZNF841; ZNF763; ZFP82; ZNF875; ZNF619; ZNF621; ZNF454; ZNF707; ZNF354C; ZNF181; ZNF546; ZFP69; ZNF680; ZNF517; ZNF850; ZNF404; ZNF284; ZNF677; ZNF860; ZNF879; ZFP57; ZNF81; ZNF530; ZNF713; ZNF429; ZNF233; ZNF699; ZNF829; ZNF568; ZNF773; ZNF790; ZNF808; ZNF888; ZNF761; ZNF470; ZNF749; ZNF324B; ZNF662; ZNF793; ZNF805; ZNF772; ZNF705A; ZNF506; ZNF716; ZNF727; ZNF571; ZNF589; ZNF107; ZNF44; ZNF853; ZNF562; ZSCAN32; ZNF331; ZNF823; ZNF416; ZNF334; ZNF701; ZNF83; ZNF415; ZNF302; ZNF253; ZNF248; ZNF630; ZNF286A; ZNF304; ZNF490; ZNF398; ZNF624; ZNF471; ZNF492; ZFP14; ZNF317; ZNF529; RBAK; ZNF77; ZNF250; ZNF350; ZNF667; ZNF674; ZNF806; ZFP69B; ZNF649; ZNF354A; ZNF736; ZNF705D; ZNF878; ZNF814; ZNF726; ZNF735; ZFP37; ZNF2; ZNF3; ZNF7; ZNF8; ZNF10; ZNF12; ZNF14; ZNF708; ZNF17; ZNF19; ZNF20; ZNF182; ZNF23; ZNF26; ZNF33A; ZNF33B; ZNF37A; ZNF41; ZNF43; ZNF45; ZNF74; ZNF79; ZNF84; ZNF221; ZNF85; ZNF90; ZNF91; ZNF99; ZNF222; ZNF124; ZNF132; ZNF133; ZNF135; ZNF136; ZNF140; ZNF141; ZNF154; ZNF155; ZNF157; ZNF175; ZNF177; ZNF180; ZNF184; ZNF189; ZNF195; ZNF200; ZNF205; ZNF208; ZNF214; ZNF223; ZNF224; ZNF225; ZNF226; ZNF227; ZNF112; ZNF229; ZNF230; ZNF426; ZNF343; ZNF557; ZNF768; ZNF419; ZNF665; ZNF552; ZNF669; ZNF212; ZNF671; ZNF613; ZNF442; ZNF556; ZNF606; ZNF614; ZNF430; ZNF34; ZNF436; ZNF611; ZNF93; ZNF484; ZNF282; ZNF528; ZNF333; ZNF527; ZNF559; ZNF347; ZNF577; ZNF607; ZNF514; ZNF382; ZNF587; ZNF566; ZNF30; ZNF551; ZNF616; ZNF766; ZNF468; ZNF160; ZNF799; ZNF700; ZNF439; ZNF486; ZNF479; ZNF251; ZNF682; ZNF765; ZNF845; ZNF300; ZNF461; ZNF585B; ZNF764; ZNF235; ZNF561; ZNF670; ZNF101; ZNF254; ZNF432; ZNF623; BST2; BAX; BAK1; CYCS; CASP3; CASP9; BID; BCL2; BCL2L1; EIF2B1; EIF2B4; EIF2B3; EIF2B2; EIF2B5; APAF1
Epstein-Barr virus infection	CR2; HLA-DMA; HLA-DMB; HLA-DOA; HLA-DOB; HLA-DPA1; HLA-DPB1; HLA-DQA1; HLA-DQA2; HLA-DQB1; HLA-DRA; HLA-DRB1; HLA-DRB3; HLA-DRB4; HLA-DRB5; RBPJL; RBPJ; SNW1; NCOR2; FCER2; MYC; HDAC1; HDAC2; CDKN1B; SKP2; RB1; USP7; TP53; TRADD; RIPK1; TRAF3; TRAF5; NFKBIA; NFKBIB; NFKBIE; MAP3K14; CHUK; NFKB1; RELA; TRAF6; TAB1; TAB2; NFKB2; RELB; ENTPD8; ENTPD1; ENTPD3; CD40; CD44; ITGAL; CD58; ICAM1; BCL2; TNFAIP3; MAP3K7; MAP2K7; MAP2K4; MAP2K3; MAP2K6; MAPK8; MAPK9; MAPK10; MAPK14; MAPK11; MAPK13; MAPK12; JUN; JAK3; PIK3CA; PIK3CB; PIK3CD; PIK3R1; PIK3R2; PIK3R3; AKT3; AKT1; AKT2; LYN; SYK; LOC102723407; PLCG2; NEDD4; DDX58; TBK1; IRF3; EIF2AK2; VIM; PSMD14; ADRM1; PSMC1; PSMC2; PSMC3; PSMC4; PSMC5; PSMC6; PSMD1; PSMD2; PSMD3; PSMD4; PSMD7; PSMD8; PSMD11; PSMD12; PSMD13; SEM1; PSMD6; HLA-A; HLA-B; HLA-C; HLA-E; HLA-F; HLA-G; TAP1; TAP2; TAPBP; PDIA3; CALR; B2M; SIN3A; SAP30L; SAP30; CIR1; HES1; RUNX3; MDM2; CDKN1A; GADD45G; GADD45A; GADD45B; BAX; BAK1; DDB2; POLK; CDK2; CCNA2; CCNA1; CCNE1; CCNE2; E2F1; E2F2; E2F3; CDK4; CDK6; CCND1; CCND2;

	CCND3; TRAF2; STAT1; STAT3; RAC1; BTK; BLNK; MAVS; IKBKE; IRF7; IFNB1; IFNA1; IFNA2; IFNA4; IFNA5; IFNA6; IFNA7; IFNA8; IFNA10; IFNA13; IFNA14; IFNA16; IFNA17; IFNA21; IFNAR1; IFNAR2; JAK1; TYK2; STAT2; IRF9; TNF; IL6; TLR2; OAS1; OAS2; OAS3; CXCL10; ISG15; MYD88; IRAK1; IRAK4; IKBKB; IKBKG; FADD; CASP3; CASP8; BCL2L11; FAS; CYCS; APAF1; CASP9; BID; CD3D; CD3E; CD3G; CD247; CD19
Human immunodeficiency virus 1 infection	RPS6KB1; RPS6KB2; MTOR; AKT3; AKT1; AKT2; PIK3CA; PIK3CB; PIK3CD; PIK3R1; PIK3R2; PIK3R3; NFKB1; RELA; HRAS; KRAS; NRAS; RAF1; MAP2K1; MAP2K2; MAPK1; MAPK3; FADD; CASP8; FAS; HLA-A; HLA-B; HLA-C; HLA-E; HLA-F; HLA-G; TRADD; CHUK; IKBKB; IKBKG; BAX; BAK1; CYCS; CASP3; TNFRSF1A; GNB5; GNB1; GNB2; GNB3; GNG3; GNG4; GNG5; GNG7; GNG10; GNG11; GNGT1; GNGT2; GNG13; GNG2; GNG12; GNB4; GNG8; CASP9; BID; NFATC1; NFATC2; NFATC3; NFATC4; PPP3CA; PPP3CB; PPP3CC; PPP3R1; PPP3R2; CALML6; CALML5; CALM1; CALM2; CALM3; CALML3; CALML4; GNA11; GNAQ; TAP1; TAP2; TAPBP; GNAI1; GNAI2; GNAI3; GNAO1; TNF; CXCR4; PTK2; CRK; CRKL; PXN; PRKCA; PRKCB; PRKCG; PTK2B; RIPK1; TRAF5; TRAF2; PDIA3; CALR; B2M; ITPR1; ITPR2; ITPR3; IFNB1; IFNA1; IFNA2; IFNA4; IFNA5; IFNA6; IFNA7; IFNA8; IFNA10; IFNA13; IFNA14; IFNA16; IFNA17; IFNA21; TBK1; IRF3; CGAS; STING1; CD4; CCR5; MYD88; MAP3K7; MAPK14; MAPK11; MAPK13; MAPK12; IRAK1; MAPK8; MAPK9; MAPK10; TRAF6; TAB1; TAB2; NFKBIA; TLR2; TLR4; IRAK4; FOS; JUN; FASLG; CDK1; ATM; ATR; CHEK1; CDC25C; CCNB3; CCNB1; CCNB2; BAD; PLCG1; PLCG2; CFL1; CFL2; LIMK1; LIMK2; PAK4; BUB1B-PAK6; PAK1; PAK2; PAK3; PAK6; PAK5; RAC1; RAC2; RAC3; APOBEC3A_B; APOBEC3D; APOBEC3H; APOBEC3A; APOBEC3F; APOBEC3C; APOBEC3G; APOBEC3B; SAMHD1; TRIM5; BST2; MAP2K3; MAP2K6; MAP2K7; CD3D; CD3E; CD3G; CD247; BCL2; BCL2L1; WEE2; WEE1; CUL4B; CUL4A; DDB1; DCAF1; ELOB; CUL5; ELOC; RNF7; AP1M2; AP1S1; AP1S3; AP1B1; AP1G1; AP1S2; AP1G2; AP1M1; TNFRSF1B; FBXW11; SKP1; CUL1; BTRC; RBX1
Colorectal cancer	DCC; CASP3; TGFB1; TGFB2; TGFB3; PIK3CA; PIK3CB; PIK3CD; PIK3R1; PIK3R2; PIK3R3; CASP9; APPL1; APC2; APC; SMAD2; SMAD3; TGFBR1; SMAD4; MLH1; MSH2; KRAS; ARAF; RAF1; BRAF; MAPK8; MAPK9; MAPK10; AXIN1; AXIN2; RALGDS; RHOA; RAC1; RAC2; RAC3; BIRC5; MYC; CCND1; MAP2K1; MAP2K2; MAPK1; MAPK3; JUN; FOS; CYCS; BCL2; TP53; LEF1; TCF7; TCF7L2; TCF7L1; GSK3B; CTNNB1; AKT3; AKT1; AKT2; BAD; MSH6; BAX; MSH3; TGFBR2; CDKN1A; GADD45G; GADD45A; GADD45B; BAK1; DDB2; POLK; PMAIP1; BCL2L11; BBC3; RALA; RALB; HRAS; NRAS; RPS6KB1; RPS6KB2; SOS1; SOS2; GRB2; MTOR; EGFR; EGF; TGFA; EREG; AREG
Renal cell carcinoma	PTPN11; TGFB1; TGFB2; TGFB3; VEGFA; SLC2A1; ARNT; ARNT2; CREBBP; EP300; CUL2; RBX1; ELOB; ELOC; VHL; EGLN2; EGLN3; EGLN1; EPAS1; HIF1A; TGFA; PDGFB; FLCN; FH; RAC1; CRK; CRKL; RAPGEF1; SOS1; SOS2; GRB2; MET; GAB1; PAK4; BUB1B-PAK6; PAK1; PAK2; PAK3; PAK6; PAK5; RAP1A; RAP1B; CDC42; ETS1; AKT3; AKT1; AKT2; PIK3CA; PIK3CB; PIK3CD; PIK3R1; PIK3R2; PIK3R3; JUN; HGF; MAPK1; MAPK3; MAP2K1; MAP2K2; ARAF; RAF1;

	BRAF; HRAS; KRAS; NRAS; BAD; PRCC; TFE3; CDKN1A
Pancreatic cancer	ERBB2; KRAS; PIK3CA; PIK3CB; PIK3CD; PIK3R1; PIK3R2; PIK3R3; ARHGEF6; AKT3; AKT1; AKT2; RAC1; RAC2; RAC3; CHUK; IKBKB; IKBKG; NFKB1; RELA; BAD; BCL2L1; ARAF; RAF1; BRAF; CASP9; MAP2K1; MAPK1; MAPK3; RALGDS; RALA; RALB; RALBP1; PLD1; PLD2; MAPK8; MAPK9; MAPK10; TGFA; EGF; JAK1; STAT3; STAT1; VEGFA; CDKN2A; CDC42; CDK4; CDK6; RB1; E2F1; E2F2; E2F3; TGFB1; TGFB2; TGFB3; TGFBR1; TGFBR2; SMAD2; SMAD3; SMAD4; BRCA2; RAD51; EGFR; CCND1; MTOR; RPS6KB1; RPS6KB2; TP53; CDKN1A; GADD45G; GADD45A; GADD45B; BAX; BAK1; DDB2; POLK
Endometrial cancer	CTNNB1; HRAS; KRAS; NRAS; APC2; APC; AXIN1; AXIN2; GSK3B; CTNNA1; CTNNA2; CTNNA3; FOXO3; BAD; CASP9; ILK; ERBB2; CDH1; LEF1; TCF7; TCF7L2; TCF7L1; PTEN; PDPK1; SOS1; SOS2; GRB2; MLH1; ELK1; EGFR; CCND1; MYC; AKT3; AKT1; AKT2; PIK3CA; PIK3CB; PIK3CD; PIK3R1; PIK3R2; PIK3R3; EGF; MAPK1; MAPK3; MAP2K1; MAP2K2; ARAF; RAF1; BRAF; TP53; CDKN1A; GADD45G; GADD45A; GADD45B; BAX; BAK1; DDB2; POLK
Glioma	EGF; TGFA; PDGFA; PDGFB; IGF1; EGFR; PDGFRA; PDGFRB; IGF1R; CALML6; CALML5; CALM1; CALM2; CALM3; CALML3; CALML4; CAMK1D; CAMK1G; CAMK4; CAMK2A; CAMK2B; CAMK2D; CAMK2G; CAMK1; PRKCA; PRKCB; PRKCG; SHC2; SHC4; SHC3; SHC1; GRB2; SOS1; SOS2; ARAF; RAF1; BRAF; MAP2K1; MAP2K2; MAPK1; MAPK3; PIK3CA; PIK3CB; PIK3CD; PIK3R1; PIK3R2; PIK3R3; AKT3; AKT1; AKT2; PTEN; MTOR; MDM2; CDKN2A; TP53; CCND1; CDK4; CDK6; RB1; E2F1; E2F2; E2F3; PLCG1; PLCG2; HRAS; KRAS; NRAS; CDKN1A; GADD45G; GADD45A; GADD45B; BAX; BAK1; DDB2; POLK
Thyroid cancer	HRAS; KRAS; NRAS; PPARG; BRAF; MAPK1; MAPK3; MAP2K1; MAP2K2; PAX8; RXRA; RXRB; RXRG; CTNNB1; MYC; CDH1; CCND1; RET; CCDC6; NCOA4; TFG; NTRK1; TPM3; TPR; LEF1; TCF7; TCF7L2; TCF7L1; TP53; CDKN1A; GADD45G; GADD45A; GADD45B; BAX; BAK1; DDB2; POLK
Melanoma	BRAF; CDH1; NRAS; CDK4; CCND1; MITF; PTEN; EGFR; FGFR1; IGF1R; MET; PDGFRA; PDGFRB; EGF; FGF1; FGF2; FGF3; FGF4; FGF5; FGF6; FGF7; FGF8; FGF9; FGF10; FGF20; FGF21; FGF22; HGF; IGF1; PDGFA; PDGFB; PDGFC; PDGFD; FGF23; FGF18; FGF17; FGF16; FGF19; BAD; CDKN2A; CDKN1A; MDM2; TP53; E2F1; E2F2; E2F3; RB1; CDK6; AKT3; AKT1; AKT2; PIK3CA; PIK3CB; PIK3CD; PIK3R1; PIK3R2; PIK3R3; MAPK1; MAPK3; MAP2K1; MAP2K2; ARAF; RAF1; HRAS; KRAS; GADD45G; GADD45A; GADD45B; BAX; BAK1; DDB2; POLK
Non-small cell lung cancer	PRKCA; PRKCB; PRKCG; FOXO3; CASP9; BAD; AKT3; AKT1; AKT2; PDPK1; PIK3CA; PIK3CB; PIK3CD; PIK3R1; PIK3R2; PIK3R3; CDK4; CDK6; CCND1; E2F1; E2F2; E2F3; HRAS; KRAS; NRAS; PLCG1; PLCG2; TGFA; EGFR; STK4; ERBB2; EGF; RB1; RARB; SOS1; SOS2; GRB2; RXRA; RXRB; RXRG; RASSF1; RASSF5; CDKN2A; FHIT; MAPK1; MAPK3; MAP2K1; MAP2K2; ARAF; RAF1; BRAF; ALK; EML4; JAK3; STAT3; STAT5A; STAT5B; TP53; CDKN1A; GADD45G; GADD45A; GADD45B; BAX; BAK1; DDB2; POLK; MET; HGF
Breast cancer	ESR1; ESR2; EGFR; NCOA3; NCOA1; HRAS; KRAS; NRAS; ARAF; RAF1; BRAF; MAP2K1; MAP2K2; MAPK1; MAPK3; SHC2; SHC4; SHC3; SHC1; GRB2; SOS1; SOS2; JAG1; JAG2; NOTCH1; NOTCH2; NOTCH3; NOTCH4; MTOR; RPS6KB1;

	RPS6KB2; IGF1; IGF1R; ERBB2; FOS; JUN; SP1; DLL3; DLL1; DLL4; PGR; PIK3CA; PIK3CB; PIK3CD; PIK3R1; PIK3R2; PIK3R3; AKT3; AKT1; AKT2; PTEN; CCND1; MYC; LEF1; TCF7; TCF7L2; TCF7L1; CSNK1A1L; CSNK1A1; AXIN1; AXIN2; APC2; APC; CTNNB1; GSK3B; FRAT1; FRAT2; DVL1; DVL2; DVL3; LRP6; LRP5; FZD10; FZD2; FZD5; FZD3; FZD1; FZD4; FZD6; FZD7; FZD8; FZD9; WNT16; WNT4; WNT1; WNT2; WNT3; WNT5A; WNT6; WNT7A; WNT7B; WNT8A; WNT8B; WNT10B; WNT11; WNT2B; WNT9A; WNT9B; WNT10A; WNT5B; WNT3A; CDK4; CDK6; RB1; E2F1; E2F2; E2F3; KIT; CDKN1A; NFKB2; FGFR1; TNFSF11; EGF; BRCA1; BRCA2; FGF1; FGF2; FGF3; FGF4; FGF5; FGF6; FGF7; FGF8; FGF9; FGF10; FGF20; FGF21; FGF22; FGF23; FGF18; FGF17; FGF16; FGF19; HES1; HES5; HEY1; HEY2; HEYL; FLT4; TP53; GADD45G; GADD45A; GADD45B; BAX; BAK1; DDB2; POLK
Hepatocellular carcinoma	EGFR; HRAS; KRAS; NRAS; ARAF; RAF1; BRAF; MAP2K1; MAP2K2; MAPK1; MAPK3; SHC2; SHC4; SHC3; SHC1; GRB2; SOS1; SOS2; MTOR; RPS6KB1; RPS6KB2; IGF2; IGF1R; PIK3CA; PIK3CB; PIK3CD; PIK3R1; PIK3R2; PIK3R3; AKT3; AKT1; AKT2; PTEN; CCND1; MYC; LEF1; TCF7; TCF7L2; TCF7L1; CSNK1A1L; CSNK1A1; AXIN1; AXIN2; APC2; APC; CTNNB1; GSK3B; FRAT1; FRAT2; DVL1; DVL2; DVL3; LRP6; LRP5; FZD10; FZD2; FZD5; FZD3; FZD1; FZD4; FZD6; FZD7; FZD8; FZD9; WNT16; WNT4; WNT1; WNT2; WNT3; WNT5A; WNT6; WNT7A; WNT7B; WNT8A; WNT8B; WNT10B; WNT11; WNT2B; WNT9A; WNT9B; WNT10A; WNT5B; WNT3A; RB1; E2F1; E2F2; E2F3; TP53; CDKN1A; GADD45G; GADD45A; GADD45B; BAX; BAK1; DDB2; POLK; TGFA; PLCG1; PLCG2; PRKCA; PRKCB; PRKCG; ELK1; TGFB1; TGFB2; TGFB3; SMAD2; SMAD3; TGFBR1; SMAD4; TGFBR2; HGF; MET; ARID1B; ARID1A; SMARCB1; SMARCC1; SMARCC2; SMARCA2; SMARCA4; ARID2; NFE2L2; TERT; KEAP1; HMOX1; NQO1; GSTO2; GSTA5; GSTA1; GSTA2; GSTA3; GSTA4; GSTM1; GSTM2; GSTM3; GSTM4; GSTM5; GSTP1; GSTT1; GSTT2; MGST1; MGST2; MGST3; GSTT2B; GSTO1; TXNRD2; TXNRD3; TXNRD1; TERC; BAD; BCL2L1; CDKN2A; CDK4; CDK6; GAB1; ACTB; ACTG1; PHF10; DPF3; DPF1; ACTL6B; ACTL6A; SMARCD1; SMARCD2; SMARCD3; SMARCE1; PBRM1; BRD7
Gastric cancer	HRAS; KRAS; NRAS; ARAF; RAF1; BRAF; MAP2K1; MAP2K2; MAPK1; MAPK3; SHC2; SHC4; SHC3; SHC1; GRB2; SOS1; SOS2; MTOR; RPS6KB1; RPS6KB2; PIK3CA; PIK3CB; PIK3CD; PIK3R1; PIK3R2; PIK3R3; AKT3; AKT1; AKT2; CCND1; MYC; LEF1; TCF7; TCF7L2; TCF7L1; CSNK1A1L; CSNK1A1; AXIN1; AXIN2; APC2; APC; CTNNB1; GSK3B; FRAT1; FRAT2; DVL1; DVL2; DVL3; LRP6; LRP5; FZD10; FZD2; FZD5; FZD3; FZD1; FZD4; FZD6; FZD7; FZD8; FZD9; WNT16; WNT4; WNT1; WNT2; WNT3; WNT5A; WNT6; WNT7A; WNT7B; WNT8A; WNT8B; WNT10B; WNT11; WNT2B; WNT9A; WNT9B; WNT10A; WNT5B; WNT3A; TP53; CDKN1A; GADD45G; GADD45A; GADD45B; BAX; BAK1; DDB2; POLK; TGFB1; TGFB2; TGFB3; SMAD2; SMAD3; TGFBR1; SMAD4; TGFBR2; HGF; MET; TERT; TERC; GAB1; ERBB2; CDX2; CDH1; CTNNA1; CTNNA2; CTNNA3; JUP; CCNE1; CCNE2; CDK2; CDKN1B; E2F1; E2F2; E2F3; RB1; SHH; BCL2; FGFR2; FGF1; FGF2; FGF3; FGF4; FGF5; FGF6; FGF7; FGF8; FGF9; FGF10; FGF20; FGF21; FGF22; FGF23; FGF18; FGF17; FGF16; FGF19; CDKN2B; RARB; RXRA; RXRB; RXRG;

	MLH1; MUC2; REG4; ABCB1; CDH17; EGFR; EGF
Autoimmune thyroid disease	HLA-DMA; HLA-DMB; HLA-DOA; HLA-DOB; HLA-DPA1; HLA-DPB1; HLA-DQA1; HLA-DQA2; HLA-DQB1; HLA-DRA; HLA-DRB1; HLA-DRB3; HLA-DRB4; HLA-DRB5; CD40LG; CD40; LOC102723407; TPO; TG; TSHR; FAS; FASLG; HLA-A; HLA-B; HLA-C; HLA-E; HLA-F; HLA-G; GZMB; PRF1; CGA; TSHB; CTLA4; IL4; IL5; IL2; IFNA1; IFNA2; IFNA4; IFNA5; IFNA6; IFNA7; IFNA8; IFNA10; IFNA13; IFNA14; IFNA16; IFNA17; IFNA21; CD80; CD86; CD28; IL10
Inflammatory bowel disease	TLR2; NFKB1; RELA; NOD2; TLR5; TNF; IL23A; TGFB1; TGFB2; TGFB3; IL6; HLA-DMA; HLA-DMB; HLA-DOA; HLA-DOB; HLA-DPA1; HLA-DPB1; HLA-DQA1; HLA-DQA2; HLA-DQB1; HLA-DRA; HLA-DRB1; HLA-DRB3; HLA-DRB4; HLA-DRB5; IL12A; IL12B; IFNG; IL12RB1; STAT4; IFNGR1; IFNGR2; STAT1; TBX21; IL4; IL4R; STAT6; GATA3; NFATC1; MAF; IL1A; IL1B; IL13; IL10; IL5; TLR4; IL2; IL23R; IL17F; IL17A; IL21; IL22; SMAD2; SMAD3; STAT3; IL18; JUN; IL12RB2; IL2RG; IL18RAP; IL18R1; RORC; IL21R; RORA; FOXP3
Systemic lupus erythematosus	LOC102723407; FCGR1A; FCGR2A; FCGR3A; FCGR3B; ELANE; CTSG; C5; C4A; C4B; C3; C2; C1S; C1QA; C1QB; C1QC; C1R; C6; C7; C8A; C8B; C8G; C9; TNF; IFNG; IL10; CD80; CD86; CD28; HLA-DMA; HLA-DMB; HLA-DOA; HLA-DOB; HLA-DPA1; HLA-DPB1; HLA-DQA1; HLA-DQA2; HLA-DQB1; HLA-DRA; HLA-DRB1; HLA-DRB3; HLA-DRB4; HLA-DRB5; CD40LG; CD40; H2AC1; H2AC8; H2AC7; H2AX; H2AZ1; H2AC21; H2AB2; H2AB1; MACROH2A2; H2AJ; H2AC19; H2AC13; H2AC15; H2AC14; H2AC16; H2AC6; H2AC4; H2AC17; H2AC18; H2AC20; H2AB3; H2AC12; H2AC11; H2AW; H2AZ2; MACROH2A1; LOC102724334; H2BE1; H2BU1; H2BW1; H2BC1; H2BW2; H2BC5; H2BC3; H2BC18; H2BS1; H2BC8; H2BC13; H2BC15; H2BC14; H2BC7; H2BC6; H2BC9; H2BC10; H2BC4; H2BC17; H2BC21; H2BC12; H2BC11; H3C14; H3-3A; H3-3B; H3C15; H3-5; H3C13; H3-4; H3C1; H3C4; H3C3; H3C6; H3C11; H3C8; H3C12; H3C10; H3C2; H3C7; H4-16; H4C15; H4C9; H4C1; H4C4; H4C6; H4C12; H4C11; H4C3; H4C8; H4C2; H4C5; H4C13; H4C7; H4C14; TRIM21; RO60; SNRPB; SNRPD1; SNRPD3; GRIN2A; ACTN4; ACTN1; SSB; GRIN2B
Rheumatoid arthritis	TNFSF11; TNFRSF11A; IL17A; IL1A; IL1B; TNF; IL6; IL23A; TGFB1; TGFB2; TGFB3; LTB; IL11; CSF1; IFNG; CSF2; MMP1; MMP3; CTSL; IL15; LOC102723407; HLA-DMA; HLA-DMB; HLA-DOA; HLA-DOB; HLA-DPA1; HLA-DPB1; HLA-DQA1; HLA-DQA2; HLA-DQB1; HLA-DRA; HLA-DRB1; HLA-DRB3; HLA-DRB4; HLA-DRB5; TCIRG1; ATP6V1G3; ATP6V0E2; ATP6V0A2; ATP6V0D2; ATP6V1C2; ATP6V0A4; ATP6V1D; ATP6V1H; ATP6V1A; ATP6V1B1; ATP6V1B2; ATP6V0C; ATP6V1C1; ATP6V1E1; ATP6V0B; ATP6V1G2; ATP6V0A1; ATP6AP1; ATP6V0E1; ATP6V1E2; ATP6V0D1; ATP6V1F; ATP6V1G1; CTSK; ACP5; TNFSF13; TNFSF13B; ITGAL; ITGB2; ICAM1; CD80; CD86; CD28; FLT1; IL18; VEGFA; CXCL12; CXCL8; CCL3L3; CCL3; CCL3L1; CXCL1; CXCL2; CXCL3; CXCL6; CXCL5; CCL20; TLR2; TLR4; FOS; JUN; CCL5; TEK; ANGPT1; CCL2; CTLA4; PTH
Graft-versus-host disease	HLA-DMA; HLA-DMB; HLA-DOA; HLA-DOB; HLA-DPA1; HLA-DPB1; HLA-DQA1; HLA-DQA2; HLA-DQB1; HLA-DRA; HLA-DRB1; HLA-DRB3; HLA-DRB4; HLA-DRB5; FAS; FASLG; HLA-A; HLA-B; HLA-C; HLA-E; HLA-F; HLA-G; GZMB; PRF1; CD28; IFNG; TNF; CD80; CD86; IL2; IL6; IL1A; IL1B; KIR3DL3; KIR2DL1;

	KIR2DL2; KIR2DL3; KIR3DL1; KIR3DL2; KLRC1; KLRD1; KIR2DL5A
Primary immunodeficiency	TAP1; CD40; CD40LG; LOC102723407; CD4; CD8A; CD8B; CD8B2; IKBKG; AICDA; UNG; CD79A; IGLL1; BLNK; BTK; ICOS; TNFRSF13C; CD19; TNFRSF13B; ADA; IL7R; IL2RG; JAK3; RAG1; RAG2; CD3D; CD3E; DCLRE1C; PTPRC; ZAP70; LCK; AIRE; TAP2; RFX5; RFXAP; RFXANK; CIITA; ORAI1
Hypertrophic cardiomyopathy	SGCD; CACNG3; CACNG2; CACNG5; CACNG4; CACNA2D3; CACNG8; CACNG7; CACNG6; CACNA1C; CACNA1D; CACNA1F; CACNA1S; CACNA2D1; CACNB1; CACNB2; CACNB3; CACNB4; CACNG1; CACNA2D2; CACNA2D4; RYR2; ATP2A1; ATP2A2; ATP2A3; SLC8A2; SLC8A1; SLC8A3; ITGA11; ITGA6; ITGA1; ITGA2; ITGA2B; ITGA3; ITGA4; ITGA5; ITGA7; ITGA9; ITGAV; ITGA10; ITGA8; ITGB1; ITGB3; ITGB4; ITGB5; ITGB6; ITGB7; ITGB8; SGCG; SGCB; SGCA; DAG1; LAMA1; LAMA2; EMD; LMNA; TNNI3; TNNC1; TNNT2; TPM1; TPM2; TPM3; TPM4; DMD; ACTC1; MYL2; TTN; MYBPC3; DES; PRKAA1; PRKAA2; PRKAB1; PRKAB2; PRKAG2; PRKAG3; PRKAG1; ACE; IGF1; TGFB1; TGFB2; TGFB3; TNF; IL6; MYL3; ACTB; ACTG1; MYH6; MYH7; EDN1; AGT
Dilated cardiomyopathy	SGCD; CACNG3; CACNG2; CACNG5; CACNG4; CACNA2D3; CACNG8; CACNG7; CACNG6; CACNA1C; CACNA1D; CACNA1F; CACNA1S; CACNA2D1; CACNB1; CACNB2; CACNB3; CACNB4; CACNG1; CACNA2D2; CACNA2D4; RYR2; ATP2A1; ATP2A2; ATP2A3; SLC8A2; SLC8A1; SLC8A3; ITGA11; ITGA6; ITGA1; ITGA2; ITGA2B; ITGA3; ITGA4; ITGA5; ITGA7; ITGA9; ITGAV; ITGA10; ITGA8; ITGB1; ITGB3; ITGB4; ITGB5; ITGB6; ITGB7; ITGB8; SGCG; SGCB; SGCA; DAG1; LAMA1; LAMA2; EMD; LMNA; TNNI3; TNNC1; TNNT2; TPM1; TPM2; TPM3; TPM4; DMD; ACTC1; MYL3; TTN; MYBPC3; DES; ADRB1; LOC102723407; GNAS; ADCY1; ADCY2; ADCY3; ADCY5; ADCY6; ADCY7; ADCY8; ADCY9; ADCY4; PRKACA; PRKACB; PRKACG; PLN; MYL2; ACTB; ACTG1; MYH6; MYH7; TNF; IGF1; TGFB1; TGFB2; TGFB3; AGT
Viral myocarditis	SGCD; SGCG; SGCB; SGCA; DAG1; LAMA1; LAMA2; DMD; EIF4G1; EIF4G2; EIF4G3; CXADR; CD55; CCND1; CD40; HLA-DMA; HLA-DMB; HLA-DOA; HLA-DOB; HLA-DPA1; HLA-DPB1; HLA-DQA1; HLA-DQA2; HLA-DQB1; HLA-DRA; HLA-DRB1; HLA-DRB3; HLA-DRB4; HLA-DRB5; CD40LG; HLA-A; HLA-B; HLA-C; HLA-E; HLA-F; HLA-G; CD28; CD80; CD86; CASP8; BID; CYCS; CASP9; CASP3; PRF1; LOC102723407; ITGAL; ITGB2; ICAM1; ACTB; ACTG1; MYH6; MYH7; FYN; ABL1; ABL2; CAV1
